# Supplementary material for: Protein abundance of AKT and ERK pathway components governs cell type‐specific regulation of proliferation
Source: Mol Syst Biol. 2017 Jan 25;13(1):904. doi: 10.15252/msb.20167258 (PMC5293153; doi:10.15252/msb.20167258)
Supplement: Supplementary file 1 — Appendix [file MSB-13-904-s001.pdf]

Protein abundance of AKT and ERK pathway components governs cell type-specific regulation of proliferation

Lorenz Adlung§, Sandip Kar§, Marie-Christine Wagner§, Bin She§, Sajib Chakraborty, Susen Lattermann, Jie Bao, Melanie Boerries, Hauke Busch, Patrick Wuchter, Anthony D. Ho, Jens Timmer, Marcel Schilling, Thomas Höfer, Ursula Klingmüller

Contents

A. Stoichiometric dose-dependency of Epo-induced proliferation in mCFU-E and BaF3-EpoR cells **1**

B. AKT phosphorylation at Thr308 and Ser473 **2**

C. Degree of phosphorylation of AKT **3**

D. Microarray analysis of cell-cycle genes and analysis of cell-cycle progression **4**

E. Impact of mTOR and RSK inhibition on S6 activation and utilization of this information for model calibration **5**

F. Quantitative dynamical modeling and model annotation **5**
F.1. Analytic solution to initial concentrations 6
F.2. Systematic model reduction at the receptor-adapter complex level 6
F.3. Parameter estimation and annotation 7
F.4. Dynamic variables, reactions and ODE systems for BaF3-EpoR, mCFU-E and 32D-EpoR cells 10

G. Analysis of Epo-induced changes and steady-state levels of signaling components **21**

H. Epo depletion by mCFU-E and BaF3-EpoR cells **22**

I. Epo-induced activation of EpoR, GTP-Ras, AKT, ERK and S6 in mCFU-E and BaF3-EpoR cells for model calibration **22**

J. Experimental overexpression of SHIP1 and PTEN in BaF3-EpoR cells and simulation of knockdown **25**

K. Basal mRNA expression of DUSP family members in mCFU-E and BaF3-EpoR cells **26**

L. Comparison of AKT, ERK and S6 activation dynamics in mCFU-E, BaF3-EpoR and 32D-EpoR cells by model simulations **27**

M. Expression of individual cell-cycle genes in mCFU-E, BaF3-EpoR and 32D-EpoR cells in response to Epo stimulation and impact of inhibitor treatment **29**

N. Linear regression to link the integrated pAKT response and the integrated ppERK response with the cell-cycle indicator in mCFU-E, BaF3-EpoR and 32D-EpoR cells **30**

O. Multiple linear regression to link the integrated pS6 response and/or the cell-cycle indicator with proliferation in mCFU-E, BaF3-EpoR and 32D-EpoR cells **31**

P. Dose-responses of Epo-induced proliferation in mCFU-E, BaF3-EpoR and 32D-EpoR cells **32**

Q. Doubling times of mCFU-E, BaF3-EpoR and 32D-EpoR cells **33**

R. Cell-type-specific links of signaling to Epo-induced proliferation in the presence of AKT an/or MEK inhibitors **34**
R.1. Analysis of cell-type-specific differences in inhibitor sensitivity of the Epo-induced signaling responses . . 34
R.2. Analysis of integrated responses of pAKT and ppERK and pS6 in mCFU-E, BaF3-EpoR and 32D-EpoR cells depending on protein abundance of pathway components 35
R.3. Analysis of the cell-type-specific link from either the integrated pS6 response or the cell-cycle indicator to proliferation upon Epo stimulation and inhibitor treatment of mCFU-E, BaF3-EpoR and 32D-EpoR cells . . 36

S. Predicting the effect of inhibitor treatment on Epo-induced proliferation in hCFU-E cells based on protein abundance 39

References 42

List of Figures

S1. : Dose-dependency of Epo-induced proliferation in normalized to EpoR surface abundance 1
S2. : AKT phosphorylation at Thr308 and Ser473 2
S3. : Determination of the degree of phosphorylation of AKT using the protein array technique 3
S4. : Microarray analysis of Epo-regulated mRNA expression in BaF3-EpoR and mCFU-E cells 4
S5. : Impact of mTOR and RSK inhibitor treatment on Epo-induced activation of S6 in mCFU-E and BaF3-EpoR cells 5
S6. : Overview of optimization performance for estimation of parameters to describe experimental data of mCFU-E and BaF3-EpoR cells 7
S7. : Analysis of Epo-induced changes and steady-state levels of signaling components in BaF3-EpoR, mCFU-E and 32D-EpoR cells 21
S8. : Epo depletion by mCFU-E and BaF3-EpoR cells 22
S9. : Dynamics of Epo-induced EpoR phosphorylation in mCFU-E and BaF3-EpoR cells 22
S10. : Epo-induced dynamics of GTP-Ras, pAKT and ppERK in mCFU-E and BaF3-EpoR cells 23
S11. : Overexpression of PTEN and SHIP1 in mCFU-E cells 23
S12. : Model calibration with experimental data of Epo-induced dynamics of pS6 in mCFU-E and BaF3-EpoR cells 24
S13. : Impact of SHIP1 and PTEN overexpression on AKT phosphorylation in BaF3-EpoR cells 25
S14. : Basal mRNA expression of DUSP family members in mCFU-E and BaF3-EpoR cells 26
S15. : Comparison of AKT, ERK and S6 activation dynamics by model simulations 27
S16. : Expression of individual cell-cycle genes in mCFU-E, BaF3-EpoR and 32D-EpoR cells in response to Epo stimulation and inhibitor treatment 29
S17. : Linear regression to link the integrated pAKT and the integrated ppERK response with the cell-cycle indicator 30
S18. : Correlation between experimentally determined and simulated cell-cycle indicator for mCFU-E, BaF3-EpoR and 32D-EpoR cells stimulated with 5 U/ml Epo in the presence of either AKT VIII or U0126 31
S19. : Correlation between the prediction and the experimental validation of Epo-induced proliferation of wild-type and SHIP1- or PTEN-overexpression mCFU-E and BaF3-EpoR cells 33
S20. : Proliferation of mCFU-E, BaF3-EpoR and 32D-EpoR cells in response to a broad range of Epo concentration 33
S21. : Proliferation as a two-step process controlling proliferation dynamics of mCFU-E, BaF3-EpoR and 32D-EpoR cells 34
S22. : Profile likelihoods for AKT and MEK inhibitor parameters in mCFU-E, BaF3-EpoR and 32D-EpoR cells 35
S23. : Dependency of the sum of the integrated response of pAKT and ppERK on the abundance of pathway components versus dependency of the integrated pS6 response on the abundance of pathway components . 35
S24. : Relative contribution of the integrated pAKT response and the integrated ppERK response to the cell-cycle indicator in mCFU-E, BaF3-EpoR and 32D-EpoR cells determined by linear regression analysis and the mathematical model 36
S25. : Correlation between experimentally measured proliferation upon Epo stimulation and single inhibitor treatment in mCFU-E, BaF3-EpoR and 32D-EpoR cells and the cell-cycle indicator or the integrated pS6 response 37
S26. : Predictions and experimental validations of proliferation upon Epo stimulation and co-treatment of inhibitors in mCFU-E, BaF3-EpoR and 32D-EpoR cells 38
S27. : Proposed scheme for cell-type-specific information processing of Epo stimulation 39
S28. : Proliferation of hCFU-E cells in response to 5 U/ml Epo 39
S29. : Expansion and differentiation of $CD34^+$ cells mobilized into the peripheral blood of three independent healthy human stem cell donors 41
S30. : Prediction of proliferation upon Epo stimulation and single or co-treatment with AKT VIII and U0126 in hCFU-E cells based on protein abundance 41
S31. : Comparison of proliferation of $CD34^+$ cells mobilized into the peripheral blood of three independent, healthy human stem cell donors 42
S32. : Comparison of proliferation upon Epo stimulation and single or co-treatment with AKT VIII and U0126 in hCFU-E cells from two independent healthy stem cell donors 42

List of Tables

S1. Overview of estimated parameters used in our mathematical model. 8
S2. Comparison of goodness of the fit for model simulations of mCFU-E, BaF3-EpoR and 32D-EpoR cells to experimental data for 32D-EpoR cells 27
S3. Data used for linear regression analysis to link the integrated pAKT response and the integrated ppERK response with the cell-cycle indicator in mCFU-E, BaF3-EpoR and 32D-EpoR cells 31
S4. Data used for multiple linear regression to link the integrated pS6 response and/or the cell-cycle indicator with proliferation in mCFU-E, BaF3-EpoR and 32D-EpoR cells 32
S5. Multiple linear regression analysis and model selection to link the integrated pS6 response and/or the cell-cycle indicator with the impact of the single inhibitors on proliferation in mCFU-E, BaF3-EpoR and 32D-EpoR cells 32
S6. Contribution of the cell-cycle indicator to Epo-induced proliferation in the presence of single inhibitors for BaF3-EpoR and 32D-EpoR cells 40

A. Stoichiometric dose-dependency of Epo-induced proliferation in mCFU-E and BaF3-EpoR cells

To directly compare the proliferative response induced by the amount of Epo normalized to the number of surface EpoR in BaF3-EpoR and CFU-E cells, we converted the Epo doses to U/EpoR taking into account the volume and the number of cells used per experiment as well as the number of EpoR on the cell surface (**Tab. 1**). The dose corresponding to 1 U/ml Epo in BaF3-EpoR cells that was used for the microarray analysis (see **Fig. S4**) was not saturating the proliferative response. Epo doses corresponding to 2.5 U/ml were applied in mCFU-E cells and Epo doses corresponding to 5 U/ml Epo were applied in BaF3-EpoR cells for activation of EpoR, AKT, ERK and S6 in mCFU-E and BaF3-EpoR cells and were used for model calibration (see **Fig. 2B**). These doses were close to saturating the proliferative response (**Fig. S1**) but still allowed dynamic activation of GTP-Ras, ERK and S6 (see **Fig. S12**).

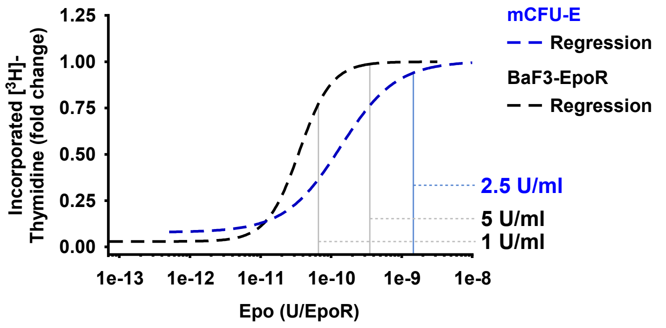

Figure S1: Dose-dependency of Epo-induced proliferation in normalized to EpoR surface abundance. DNA content of mCFU-E and BaF3-EpoR cells in response to different Epo concentrations. [3H]-Thymidine incorporation was measured after 14 h (mCFU-E) or 38 h (BaF3-EpoR). Data was scaled from U/ml to U/EpoR by taking the stimulation volume, the number of cells and the number of EpoR on the respective cell surface into account. Data is represented as mean ± standard deviation, N=3. Lines represent sigmoidal regression from **Fig. 1A**. Indicated Epo doses were used for experiments.

B. AKT phosphorylation at Thr308 and Ser473

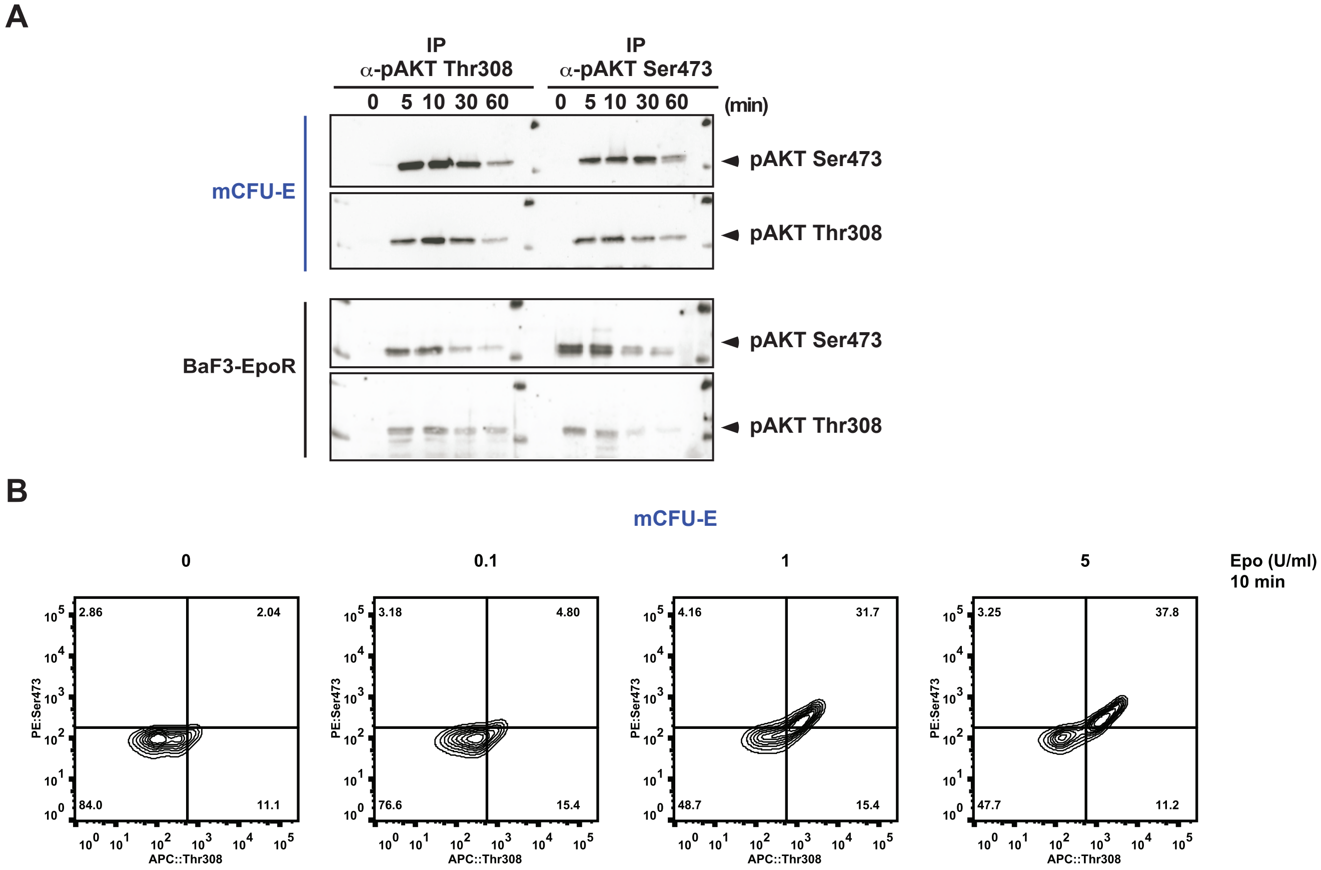

Figure S2: AKT phosphorylation at Thr308 and Ser473. (A) Detection of AKT phosphorylation at Thr308 and Ser473 by quantitative immunoblotting. Cells were deprived from growth factors and each 4×10^7 cells/ml were stimulated with 50 U/ml Epo for indicated time points. Cellular lysates of each 8×10^6 mCFU-E cells or 1.9×10^7 BaF3-EpoR cells were subjected to immunoprecipitation with antibodies against pAKT Thr308 or pAKT Ser473 (both Cell Signaling), respectively. The same antibodies were used for detection of the immunoblots. The dynamics of pAKT Thr308 and pAKT Ser473 was the same independent of whether the pAKT Thr308 or the pAKT Ser473 was utilized for the immunoprecipitation showing synchronous activation of both residues in mCFU-E and BaF3-EpoR cells. (B) Detection of AKT phosphorylation at Thr308 and Ser473 by flow cytometry. mCFU-E cells were deprived from growth factors and were stimulated with the indicated Epo doses for 10 min. Stimulation was terminated by fixation of the cells with 1.6 % paraformaldehyde and permeabilization with 90 % methanol. A PE-conjugated Phospho-Akt (Ser473) antibody and an Alexa Fluor® 647-coupled Phospho-Akt (Thr308) antibody (both Cell Signaling) were applied. Signals were acquired on a FACS CantoII (BD Biosciences). The detected signal on the diagonal shows correlated activation of both phosphorylation sites in mCFU-E cells. Circles of contour plots always indicate 10 % of the cell population. The experiment was performed twice. A representative dataset is shown.

C. Degree of phosphorylation of AKT

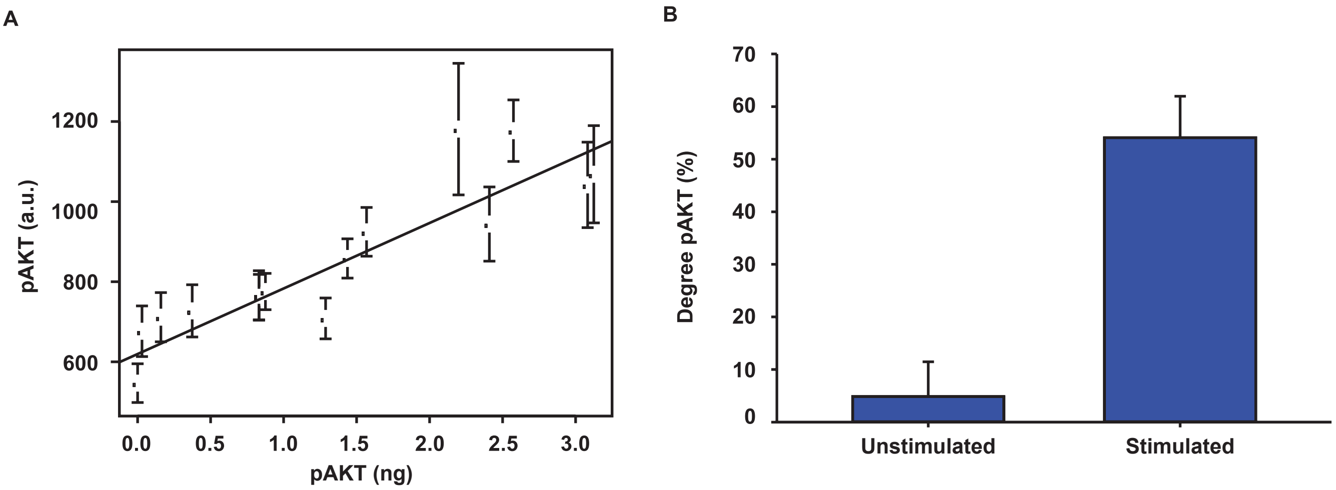

Figure S3: Determination of the degree of phosphorylation of AKT using the protein array technique. Each 4×10^5 growth-factor deprived mCFU-E cells were stimulated with 2.5 U/ml Epo for 10 min while unstimulated cells served as control. For protein array analysis 10 % SDS was added to the cellular lysates. Samples were boiled at 95°C for 3 min, Array Buffer PLUS (Roche) was added and equal volumes of sample and pAKT calibrator were loaded on the blocked array slides to ensure a quantitative analysis. The pAKT calibrator was obtained from Millipore (# 14-276) and the degree of phosphorylation was determined by mass spectrometry. The capturing antibody recognized pAKT (Upstate) The loaded array was incubated over night at 4°C, followed by three times washing for 5 minutes with array buffer at room temperature on the shaker. The detection antibodies against total AKT (Cell Signaling, Santa Cruz) were added for two hours at 4°C, followed by extensive washing steps before adding the secondary fluorescent labeled antibody (Molecular probes) for 30 min at 4°C. The slides were washed with washing buffer and ddH_2O, and the slides were scanned with the LiCor Odyssey scanner. (A) Calibration of the protein array with recombinant pAKT. Calculated absolute amounts of recombinant pAKT were detected by the protein array to show the linear relation between the absolute amounts of pAKT and the measured signal within the detection range. (B) Results for the degree of AKT phosphorylation in percent of total AKT. Results are shown as mean with error bars indicating standard deviation. N=3.

D. Microarray analysis of cell-cycle genes and analysis of cell-cycle progression

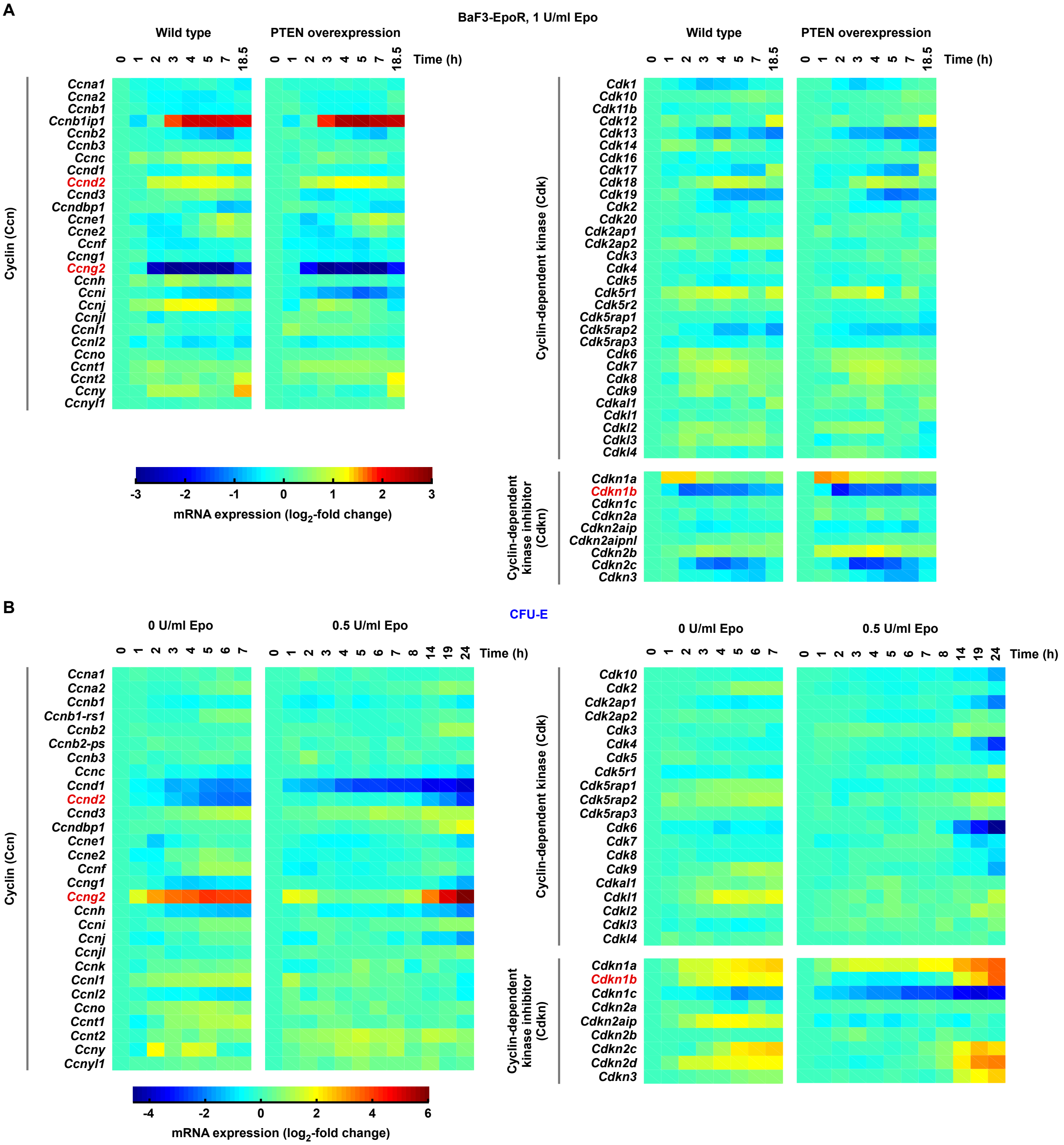

Figure S4: Microarray analysis of Epo-regulated mRNA expression in BaF3-EpoR and mCFU-E cells. Per time point, total RNA from 4×10^6 BaF3-EpoR or 3×10^6 mCFU-E cells was isolated. (A) Wild-type and PTEN-overexpressing BaF3-EpoR cells were stimulated with 1 U/ml Epo for up to 18.5 h. (B) Freshly prepared, growth-factor deprived mCFU-E cells were stimulated with 0.5 U/ml Epo for up to 24 h or left unstimulated for up to 7 h. Data of mCFU-E cells are available elsewhere (Bachmann et al. 2011). mRNA was extracted and expression of cell-cycle regulated genes was analyzed by a microarray (Affymetrix). Genes selected for the cell-cycle indicator are marked in red. The $\log_2$-fold change expression is displayed relative to the respective 0 h time point.

E. Impact of mTOR and RSK inhibition on S6 activation and utilization of this information for model calibration

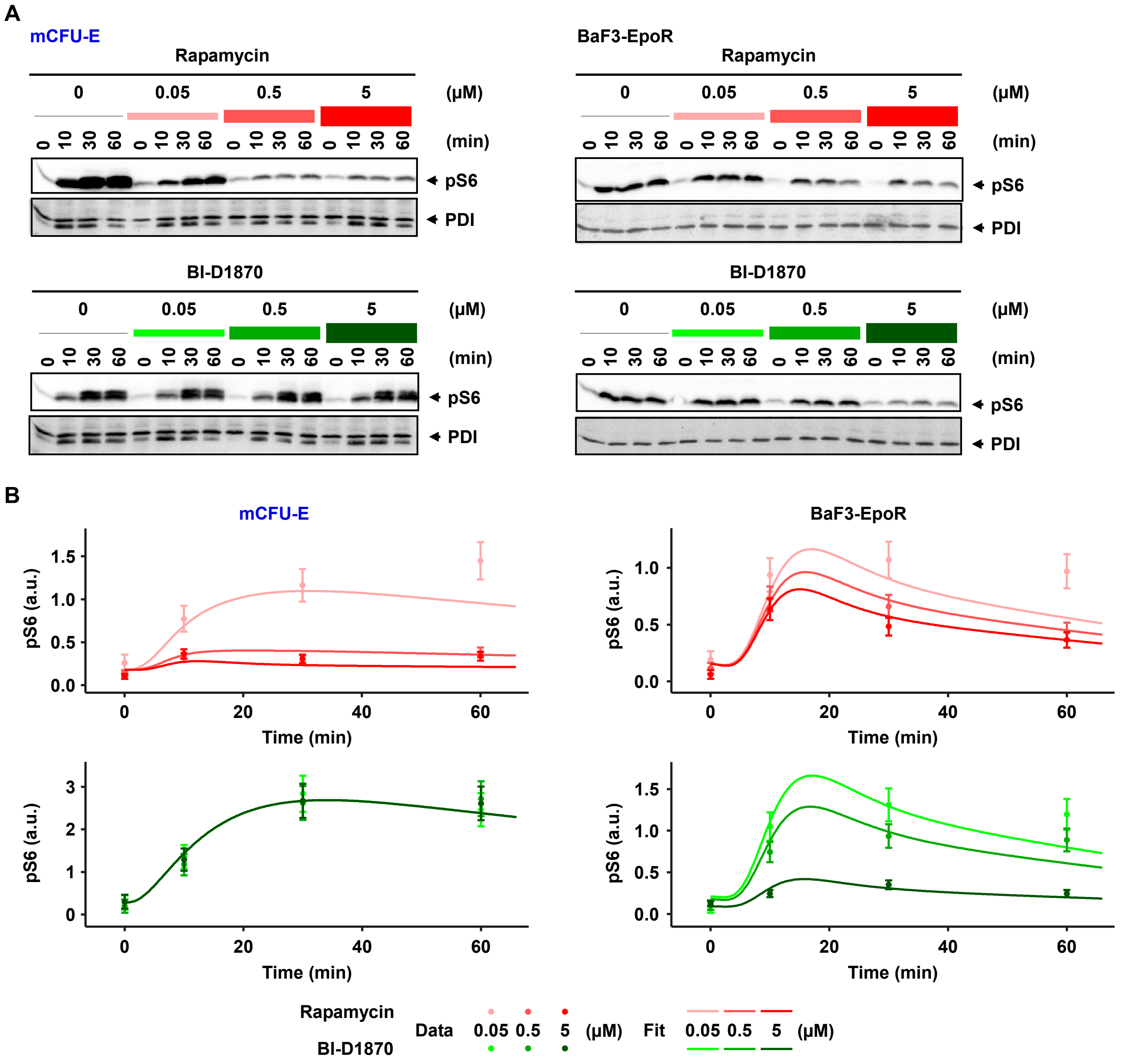

Figure S5: Impact of mTOR and RSK inhibitor treatment on Epo-induced activation of S6 in mCFU-E and BaF3-EpoR cells. (A) Growth-factor deprived mCFU-E and BaF3-EpoR cells were pre-treated for 30 minutes with increasing concentrations of RSK inhibitor (BI-D1870, upper panel) and mTOR inhibitor (Rapamycin, lower panel) and stimulated with 5 U/ml Epo for indicated times. pS6 dynamics were determined by quantitative immunoblotting of 5×10^6 mCFU-E or 1×10^7 BaF3-EpoR cells per condition. PDI served as loading control. (B) Fits of Epo-induced pS6 dynamics by the mathematical model for mCFU-E and BaF3-EpoR cells during RSK inhibitor (BI-D1870) and Rapamycin treatment. Filled circles represent experimental data shown in (A) as mean ± standard deviation given by the error model. Solid lines represent model trajectories

F. Quantitative dynamical modeling and model annotation

The assumption behind our mathematical model was that the change of concentrations of signaling components $\vec{C}$ in response to Epo can be described as a function of a set of cell-type-specific protein abundance $\vec{P}$ and a set of shared kinetic parameters $\vec{K}$:

$$\frac{\mathrm{d}\vec{C}}{\mathrm{d}t} = f\left(\vec{C}, \vec{P}, \vec{K}\right).$$

Following a fundamental concept of enzyme kinetics, we decomposed the enzymatic rate constants (e. g., for phosphatases and kinases) into the product of total enzyme concentration and a biochemical rate constant (also called catalytic efficiency, or turnover, k_{cat}). Whereas the biochemical rate constant is a property of the enzyme and can therefore be assumed as independent of a given cell type, the enzyme concentration is cell-type-specific. This decomposition of the rate constants affords the experimental quantification and subsequent prediction of cell-type-specific signal transduction. In addition to measuring the dynamics of signal transduction (phosphorylation states etc.) we now added a static measurement, namely the protein abundance of signaling components in a given cell type. Protein abundance represents concentrations of total proteins that are in steady state before and after stimulation (see **Appendix G**). They do not change within the time-scale of the experiment and should therefore be characteristic for tissue samples as well. We used these static measurements of protein abundance in BaF3-EpoR and mCFU-E cells to estimate the cell-type-independent biochemical rate constants. Once these were known, we successfully predicted the signaling dynamics in 32D-EpoR cells by simply conducting the static measurement of protein abundance of signaling components and inserting these data into the mathematical model. We assumed cell-type-specific kinetic parameters for the receptor part of the model because mCFU-E and hCFU-E cells possessed endogenous EpoR levels whereas BaF3-EpoR and 32D-EpoR cells were ectopically expressing a retrovirally transduced HA-tagged version of the EpoR. Enzymatic rates downstream of the receptor were assumed to be equal for mCFU-E, hCFU-E, BaF3-EpoR and 32D-EpoR cells. The only difference between the cell types was the cell-type-specific protein abundance of signaling components. Activation of AKT and ERK was linked to the cell-cycle indicator by linear regression analysis (see **Appendix N**). Parameters for the link from integrated pAKT and integrated ppERK to the cell-cycle indicator determined by this linear regression analysis were implemented in the dynamic mathematical model. Activation of S6 and/or cell-cycle indicator was linked to proliferation by multiple linear regression analysis (see **Appendix O**). Parameters for the link from integrated pS6 or the cell-cycle indicator to proliferation determined by this multiple linear regression analysis were implemented in the dynamic mathematical model (**Fig. 6A**). The selected best model of the multiple linear regression analysis revealed that integrated pS6 describes proliferation best for mCFU-E cells whereas the cell-cycle indicator describes proliferation best for BaF3-EpoR and 32D-EpoR cells (**Fig. 6C**).
In the mathematical model, we annotated the AKT inhibitor AKT VIII with Aktin, the MEK inhibitor U0126 with Mekin, the mTOR inhibitor with $\mathrm{Rapamycin}$ and the RSK inhibitor BI-D1870 with RSKin.

F.1. Analytic solution to initial concentrations

Steady states for the initial values of some model species were solved analytically and are given below for mCFU-E cells.

$$\mathrm{init\_GTP\_Ras} = \frac{\mathrm{init\_Rast} \cdot \mathrm{k\_back\_Ras}}{\mathrm{k\_back\_Ras} + \mathrm{kdRas}}$$

$$\mathrm{init\_pRaf} = \frac{\mathrm{init\_Raft} \cdot \mathrm{init\_GTP\_Ras} \cdot \mathrm{kpRaf}}{\mathrm{kpRaf} + \mathrm{lpRaf}}$$

$$\mathrm{init\_pMek} = \frac{\mathrm{init\_pRaf} \cdot \mathrm{init\_Mekt} \cdot \mathrm{kpMek}}{\frac{\mathrm{kpMek} \cdot \mathrm{kppMek} \cdot \mathrm{init\_pRaf}^2}{\mathrm{lppMek}} + \mathrm{kpMek} \cdot \mathrm{init\_pRaf} + \mathrm{lpMek}}$$

$$\mathrm{init\_ppMek} = \frac{\mathrm{init\_pRaf} \cdot \mathrm{init\_pMek} \cdot \mathrm{kppMek}}{\mathrm{lppMek}}$$

$$\mathrm{init\_pErk} = \frac{\mathrm{init\_Erkt} \cdot \mathrm{init\_ppMek} \cdot \mathrm{kpErk}}{\frac{\mathrm{kpErk} \cdot \mathrm{kppErk} \cdot \mathrm{init\_ppMek}^2}{\mathrm{cDUSP3} \cdot \mathrm{lppErk}} + \mathrm{kpErk} \cdot \mathrm{init\_ppMek} + \mathrm{cDUSP3} \cdot \mathrm{lpErk}}$$

$$\mathrm{init\_ppErk} = \frac{\mathrm{init\_pErk} \cdot \mathrm{init\_ppMek} \cdot \mathrm{kppErk}}{\mathrm{cDUSP3} \cdot \mathrm{lppErk}}$$

$$\mathrm{init\_PIP3} = -\frac{\mathrm{init\_PI45P2} \cdot \mathrm{vPIP3\_basal} \cdot (\mathrm{init\_Rp\_PI3K} - \mathrm{init\_PI3Kt} + \mathrm{init\_Rp\_Gab2\_PI3K})}{\mathrm{init\_PTEN} \cdot \mathrm{vPI45P2} + \mathrm{kshbasal} \cdot (\mathrm{init\_SHIP1t} - \mathrm{init\_Rp\_SHIP1})}$$

$$\mathrm{init\_PIP3\_PDK1} = \frac{\mathrm{init\_PIP3} \cdot \mathrm{init\_PDK1t} \cdot \mathrm{kPDK1}}{\mathrm{lPDK1} + \mathrm{init\_PIP3} \cdot \mathrm{kPDK1}}$$

$$\mathrm{init\_PIP3\_Akt} = \frac{\mathrm{dpAkt} \cdot \mathrm{init\_PIP3} \cdot \mathrm{init\_Aktt} \cdot \mathrm{kAkt}}{\mathrm{dpAkt} \cdot \mathrm{lAkt} + \mathrm{dpAkt} \cdot \mathrm{init\_PIP3} \cdot \mathrm{kAkt} + \mathrm{dpAkt} \cdot \mathrm{init\_PIP3\_PDK1} \cdot \mathrm{vpAkt} + \mathrm{init\_PIP3} \cdot \mathrm{init\_PIP3\_PDK1} \cdot \mathrm{kAkt} \cdot \mathrm{vpAkt}}$$

$$\mathrm{init\_pAkt} = \frac{\mathrm{init\_PIP3} \cdot \mathrm{init\_Aktt} \cdot \mathrm{init\_PIP3\_PDK1} \cdot \mathrm{kAkt} \cdot \mathrm{vpAkt}}{\mathrm{dpAkt} \cdot \mathrm{lAkt} + \mathrm{dpAkt} \cdot \mathrm{init\_PIP3} \cdot \mathrm{kAkt} + \mathrm{dpAkt} \cdot \mathrm{init\_PIP3\_PDK1} \cdot \mathrm{vpAkt} + \mathrm{init\_PIP3} \cdot \mathrm{init\_PIP3\_PDK1} \cdot \mathrm{kAkt} \cdot \mathrm{vpAkt}}$$

$$\mathrm{init\_RSKa} = \frac{\mathrm{init\_RSKtc} \cdot \mathrm{init\_ppErk} \cdot \mathrm{karsk}}{(\mathrm{init\_RSKin} \cdot \mathrm{kinrskc} + 1) \cdot (\mathrm{init\_Mekin} \cdot \mathrm{kmekinc} + 1) \cdot (\mathrm{kdphoss6} + \mathrm{init\_TORC1} \cdot \mathrm{kas6t} + \mathrm{init\_RSKa} \cdot \mathrm{kas6r})}$$

$$\mathrm{init\_pS6} = \frac{\mathrm{init\_S6t} \cdot (\mathrm{kbacks6} + \mathrm{init\_TORC1} \cdot \mathrm{kas6t} + \mathrm{init\_RSKa} \cdot \mathrm{kas6r})}{\mathrm{kbacks6} + \mathrm{kdphoss6} + \mathrm{init\_TORC1} \cdot \mathrm{kas6t} + \mathrm{init\_RSKa} \cdot \mathrm{kas6r}}$$

For BaF3-EpoR cells the following parameters are changed: $\mathrm{cDUSP3} \rightarrow \mathrm{bDUSP3}$, $\mathrm{RSKtc} \rightarrow \mathrm{RSKtb}$, $\mathrm{kinrskc} \rightarrow \mathrm{kinrskb}$, $\mathrm{kmekinc} \rightarrow \mathrm{kmekinb}$.

F.2. Systematic model reduction at the receptor-adapter complex level

Many proteins are binding to the EpoR thereby serving as adapters for downstream components of signaling pathways. These adapters include Gab1/2 (i.e. **Gab1** for mCFU-E and hCFU-E cells, or **Gab2** for BaF3-EpoR and 32D-EpoR cells), **PI3K**, and GTP-Ras (**GTR**). During the formation of the receptor complexes, binding was supposed to happen in fast equilibrium. The rate-limiting step was receptor phosphorylation (**EpoR** $\rightarrow$ **Rp**). By assuming conservation of mass, the

following scheme for complex formation was derived with given rates, here exemplarily shown for mCFU-E and hCFU-E cells:

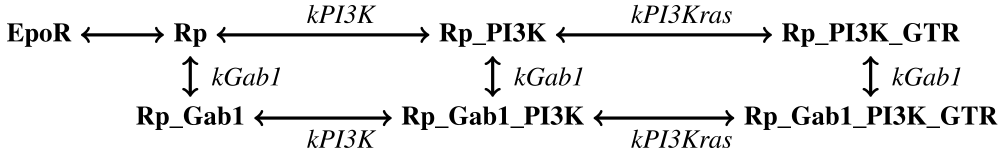

In the respective rate equations, the respective species were substituted as follows:

$$\mathrm{Rp\_Gab1} = \frac{\mathrm{kGab1} \cdot [\mathrm{pEpoR}]}{(\mathrm{kGab1}+1)\cdot(\mathrm{kPI3K}+1)+\mathrm{kPI3K}\cdot\mathrm{kPI3Kras}\cdot(\mathrm{kGab1}+1)}$$

$$\mathrm{Rp\_PI3K} = \frac{\mathrm{kPI3K} \cdot [\mathrm{pEpoR}]}{(\mathrm{kGab1}+1)\cdot(\mathrm{kPI3K}+1)+\mathrm{kPI3K}\cdot\mathrm{kPI3Kras}\cdot(\mathrm{kGab1}+1)}$$

$$\mathrm{Rp\_Gab1\_PI3K} = \frac{\mathrm{kGab1}\cdot\mathrm{kPI3K} \cdot [\mathrm{pEpoR}]}{(\mathrm{kGab1}+1)\cdot(\mathrm{kPI3K}+1)+\mathrm{kPI3K}\cdot\mathrm{kPI3Kras}\cdot(\mathrm{kGab1}+1)}$$

$$\mathrm{Rp\_PI3K\_GTR} = \frac{\mathrm{kPI3K}\cdot\mathrm{kPI3Kras} \cdot [\mathrm{pEpoR}]}{(\mathrm{kGab1}+1)\cdot(\mathrm{kPI3K}+1)+\mathrm{kPI3K}\cdot\mathrm{kPI3Kras}\cdot(\mathrm{kGab1}+1)}$$

$$\mathrm{Rp\_Gab1\_PI3K\_GTR} = \frac{\mathrm{kGab1}\cdot\mathrm{kPI3K}\cdot\mathrm{kPI3Kras} \cdot [\mathrm{pEpoR}]}{(\mathrm{kGab1}+1)\cdot(\mathrm{kPI3K}+1)+\mathrm{kPI3K}\cdot\mathrm{kPI3Kras}\cdot(\mathrm{kGab1}+1)}$$

Parameters of the rate equations were estimated with the mathematical model for mCFU-E and BaF3-EpoR cells. For BaF3-EpoR cells $\mathrm{kGab1}$ was substituted by $\mathrm{kGab2}$ because only Gab2 is present in BaF3-EpoR cells (see **Tab. 1**). Since $\mathrm{kGab1}$ and $\mathrm{kGab2}$ were estimated to be either very low or very high, the Gab1/2 species was removed from the scheme and only **PI3K** and **GTR** were considered as part of the receptor-adapter complex in the reduced model. Species were subsequently substituted by the rate equations.

F.3. Parameter estimation and annotation

Apart from the scaling parameters, 97 parameters were estimated with the mathematical model (**Tab. S1**). The optimization procedure during parameter estimation performed well as more than 90 % of the multi-start runs converged and the difference in the objective function among the 100 best runs was less than 10 (**Fig. S6**)

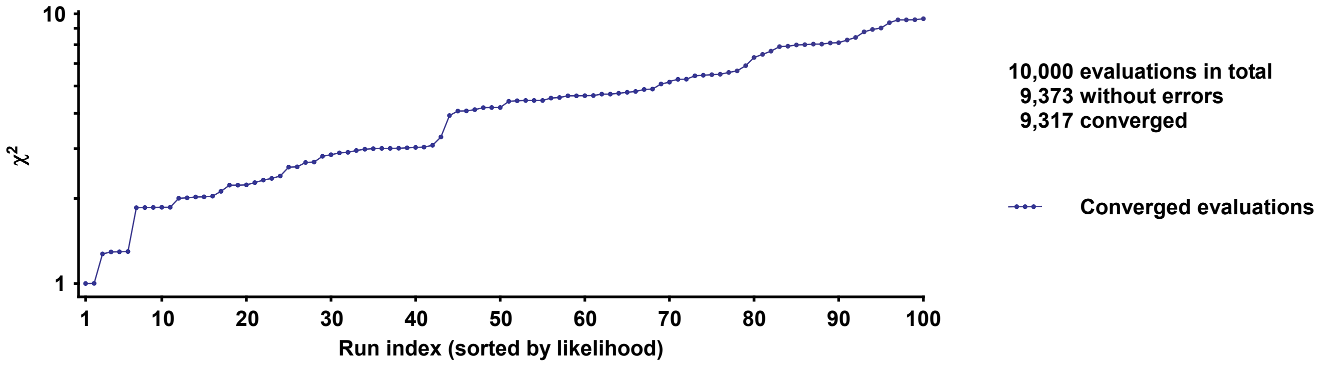

Figure S6: Overview of optimization performance for estimation of parameters to describe experimental data of mCFU-E and BaF3-EpoR cells. Best 100 optimization runs are sorted according to their likelihood. The best fit was found twice and and the best 11 runs were very similar with respect to their χ^2.

Table S1: Overview of estimated parameters used in our mathematical model.

Name of parameter p	Explanation
EpoR_activation	Erythropoietin receptor activation rate in mCFU-E cells
EpoR_activation1	Erythropoietin receptor activation rate in BaF3-EpoR cells
Epo_degradation	Erythropoietin degradation rate
Ji	concentration of pEpoR for half-maximum activation of phosphatase in mCFU-E cells
Ji1	concentration of pEpoR for half-maximum activation of phosphatase in BaF3-EpoR cells
Ki	half-maximal effective concentration of phosphatase inhibiting EpoR phosphorylation in mCFU-E cells
Ki1	half-maximal effective concentration of phosphatase inhibiting EpoR phosphorylation in BaF3-EpoR cells
Phos_act	activation rate of constitutively active phosphatase in mCFU-E cells
Phos_act1	activation rate of constitutively active phosphatase in BaF3-EpoR cells
Phos_deact	deactivation rate of constitutively active phosphatase in mCFU-E cells
Phos_deact1	deactivation rate of constitutively active phosphatase in BaF3-EpoR cells
Phosphatase_activation	activation rate of induced phosphatase in mCFU-E cells
Phosphatase_activation1	activation rate of induced phosphatase in BaF3-EpoR cells
Phosphatase_deactivation	deactivation rate of induced phosphatase in mCFU-E cells
Phosphatase_deactivation1	deactivation rate of induced phosphatase in BaF3-EpoR cells
Sostb	total abundance of Sos in BaF3-EpoR cells
Sostc	total abundance of Sos in mCFU-E cells
bDUSP3	protein abundance of a representative DUSP family member in BaF3-EpoR cells
cDUSP3	protein abundance of a representative DUSP family member in mCFU-E cells
cycleaktinb	regression coefficient for impact of AKT inhibitor on Cell cycle indicator in BaF3-EpoR cells
cycleaktinc	regression coefficient for impact of AKT inhibitor on Cell cycle indicator in mCFU-E cells
cycleaktind	regression coefficient for impact of AKT inhibitor on Cell cycle indicator in 32D-EpoR cells
cyclemekinb	regression coefficient for impact of MEK inhibitor on Cell cycle indicator in BaF3-EpoR cells
cyclemekinc	regression coefficient for impact of MEK inhibitor on Cell cycle indicator in mCFU-E cells
cyclemekind	regression coefficient for impact of MEK inhibitor on Cell cycle indicator in 32D-EpoR cells
dDUSP3	protein abundance of a representative DUSP family member in 32D-EpoR cells (set to bDUSP3)
dpAkt	dephosphorylation rate of pAKT
kAkt	association rate for complex formation between AKT and PIP3
kPDK1	association rate for complex formation between PDK1 and PIP3
kPI3K	association rate of PI3K being recruited to the receptor
kPI3Kras	association rate of the PI3K-GTP-Ras complex being recruited to the receptor
kSHIP1	association rate of SHIP1 being recruited to the receptor
kSos	association rate of Sos being recruited to the receptor
k_back_Ras	receptor independent association rate for GTP-Ras
kaRas	receptor-bound Sos-dependent activation rate of GTP-Ras
kaktinb	AKT inhibitor strength on AKT phosphorylation in BaF3-EpoR cells
kaktinc	AKT inhibitor strength on AKT phosphorylation in mCFU-E cells
kaktind	AKT inhibitor strength on AKT phosphorylation in 32D-EpoR cells
karsk	phosphorylation rate of RSK
kas6r	pRSK-dependent phosphorylation rate of S6
kas6t	TORC1-dependent phosphorylation rate of S6
kbacks6	basal phosphorylation rate of S6
kdRas	dissociation rate of GTP-Ras
kdactm	deactivation rate of mTOR
kdpes	dephosphorylation rate of pSos
kdphoss6	dephosphorylation rate of pS6
kdrsk	dephosphorylation rate of pRSK
kdt1	dissociation rate of active mTOR from TORC1
kdt2	dissociation rate of active mTOR from TORC2
kinrapab	Rapamycin strength on formation of TORC1 in BaF3-EpoR cells
kinrapac	Rapamycin strength on formation of TORC1 in mCFU-E cells
kinrskb	RSK inhibitor strength on RSK phosphorylation in BaF3-EpoR
kinrskc	RSK inhibitor strength on RSK phosphorylation in mCFU-E
kmekinb	MEK inhibitor strength on pERK phosphorylation in BaF3-EpoR cells
kmekinc	MEK inhibitor strength on pERK phosphorylation in mCFU-E cells
kmekind	MEK inhibitor strength on pERK phosphorylation in 32D-EpoR cells
kpErk	phosphorylation rate of ERK
kpMek	phosphorylation rate MEK
kpRaf	phosphorylation rate of Raf
kpes	phosphorylation rate of Sos
kppErk	phosphorylation rate of pERK
kppMek	phosphorylation rate of pMEK
ksatb	Epo concentration occupying half of EpoR in BaF3-EpoR cells
ksatc	Epo concentration occupying half of EpoR in mCFU-E cells
kshbasal	basal degradation rate of PIP3
kst1	association rate for TORC1
kst2	association rate for TORC2
kvpakt	pAKT-dependent activation rate of mTOR
kvppErk	ppERK-dependent activation rate of mTOR
lAkt	dissociation rate of PIP3 from complex with AKT
lPDK1	dissociation rate of PIP3 from complex with PDK1
lSHIP1	dissociation rate of SHIP1 from EpoR

Continued on next page

Table S1 – continued from previous page

Name of parameter p	Explanation
lSos	dissociation rate of Sos from EpoR
lpErk	DUSP-dependent dephosphorylation rate of pERK
lpMek	dephosphorylation rate of pMEK
lpRaf	dephosphorylation rate of pRaf
lppErk	DUSP-dependent dephosphorylation rate of ppERK
lppMek	dephosphorylation rate of ppMEK
main	main phosphorylation rate of PIP3 in mCFU-E cells
main1	main phosphorylation rate of PIP3 in BaF3-EpoR cells
n	Hill coefficient of phosphatase activation in mCFU-E cells
n1	Hill coefficient of phosphatase activation in BaF3-EpoR cells
nb	Hill coefficient of EpoR phosphorylation in BaF3-EpoR cells
nc	Hill coefficient of EpoR phosphorylation in mCFU-E cells
pEpoR_deactivation	dephosphorylation rate of pEpoR in mCFU-E cells
pEpoR_deactivation1	dephosphorylation rate of pEpoR in BaF3-EpoR cells
pbk1	regression coefficient for impact of Cell Cycle indicator on proliferation of BaF3-EpoR cells
pboff	regression coefficient for offset of Cell Cycle indicator on proliferation of BaF3-EpoR cells
pck1	regression coefficient for impact of integrated pS6 on proliferation of mCFU-E cells
pcoff	regression coefficient for offset of integrated pS6 on proliferation of mCFU-E cells
pdk1	regression coefficient for impact of Cell Cycle indicator on proliferation of 32D-EpoR cells
pdoff	regression coefficient for offset of Cell Cycle indicator on proliferation of 32D-EpoR cells
vPI34P2	EpoR-bound SHIP1-dependent dephosphorylation rate of PIP3
vPI45P2	PTEN-dependent dephosphorylation rate of PIP3
vPIP3	adaptor complex-dependent phosphorylation rate of PIP2
vPIP3_basal	basal phosphorylation rate of PIP3
vpAkt	phosphorylation rate of AKT

F.4. Dynamic variables, reactions and ODE systems for BaF3-EpoR, mCFU-E and 32D-EpoR cells

F.4.1. BaF3-EpoR: Dynamic variables

The model contains 44 dynamic variables:

$$[\text{BaF3\_Epo}](t=0) = \text{init\_BaF3\_Epo} \quad \text{conc. [nM]} \tag{1}$$
$$[\text{EpoR\_BaF3}](t=0) = \text{init\_EpoR\_BaF3} \quad \text{conc. [nM]} \tag{2}$$
$$[\text{Phosphataset\_BaF3}](t=0) = \text{init\_Phosphataset\_BaF3} \quad \text{conc. [nM]} \tag{3}$$
$$[\text{SHIP1t\_BaF3}](t=0) = \text{init\_SHIP1t\_BaF3} \quad \text{conc. [nM]} \tag{4}$$
$$[\text{PI3Kt\_BaF3}](t=0) = \text{init\_PI3Kt\_BaF3} \quad \text{conc. [nM]} \tag{5}$$
$$[\text{PI45P2\_BaF3}](t=0) = \text{init\_PI45P2\_BaF3} \quad \text{conc. [nM]} \tag{6}$$
$$[\text{PTEN\_BaF3}](t=0) = \text{init\_PTEN\_BaF3} \quad \text{conc. [nM]} \tag{7}$$
$$[\text{PDK1t\_BaF3}](t=0) = \text{init\_PDK1t\_BaF3} \quad \text{conc. [nM]} \tag{8}$$
$$[\text{Aktt\_BaF3}](t=0) = \text{init\_Aktt\_BaF3} \quad \text{conc. [nM]} \tag{9}$$
$$[\text{Rast\_BaF3}](t=0) = \text{init\_Rast\_BaF3} \quad \text{conc. [nM]} \tag{10}$$

$$[\text{Raft\_BaF3}](t=0) = \text{init\_Raft\_BaF3} \quad \text{conc. [nM]} \tag{11}$$
$$[\text{Mekt\_BaF3}](t=0) = \text{init\_Mekt\_BaF3} \quad \text{conc. [nM]} \tag{12}$$
$$[\text{Erkt\_BaF3}](t=0) = \text{init\_Erkt\_BaF3} \quad \text{conc. [nM]} \tag{13}$$
$$[\text{S6t\_BaF3}](t=0) = \text{init\_S6t\_BaF3} \quad \text{conc. [nM]} \tag{14}$$
$$[\text{mTORt}](t=0) = \text{init\_mTORt} \quad \text{conc. [nM]} \tag{15}$$
$$[\text{Raptorb}](t=0) = \text{init\_Raptorb} \quad \text{conc. [nM]} \tag{16}$$
$$[\text{Rictorb}](t=0) = \text{init\_Rictorb} \quad \text{conc. [nM]} \tag{17}$$
$$[\text{RSKtb}](t=0) = \text{init\_RSKtb} \quad \text{conc. [nM]} \tag{18}$$
$$[\text{pEpoR}](t=0) = \text{init\_pEpoR} \quad \text{conc. [nM]} \tag{19}$$
$$[\text{phos2}](t=0) = \text{init\_phos2} \quad \text{conc. [nM]} \tag{20}$$

$$[\text{actPhosphatase}](t=0) = \text{init\_actPhosphatase} \quad \text{conc. [nM]} \tag{21}$$
$$[\text{GTP\_Ras}](t=0) = \text{init\_GTP\_Ras} \quad \text{conc. [nM]} \tag{22}$$
$$[\text{Rp\_SHIP1}](t=0) = \text{init\_Rp\_SHIP1} \quad \text{conc. [nM]} \tag{23}$$
$$[\text{PIP3}](t=0) = \text{init\_PIP3} \quad \text{conc. [nM]} \tag{24}$$
$$[\text{PIP3\_PDK1}](t=0) = \text{init\_PIP3\_PDK1} \quad \text{conc. [nM]} \tag{25}$$
$$[\text{PIP3\_Akt}](t=0) = \text{init\_PIP3\_Akt} \quad \text{conc. [nM]} \tag{26}$$
$$[\text{pAkt}](t=0) = \text{init\_pAkt} \quad \text{conc. [nM]} \tag{27}$$
$$[\text{pSos}](t=0) = \text{init\_pSos} \quad \text{conc. [nM]} \tag{28}$$
$$[\text{Rp\_Sos}](t=0) = \text{init\_Rp\_Sos} \quad \text{conc. [nM]} \tag{29}$$
$$[\text{ppErk}](t=0) = \text{init\_ppErk} \quad \text{conc. [nM]} \tag{30}$$

$$[\text{pRaf}](t=0) = \text{init\_pRaf} \quad \text{conc. [nM]} \tag{31}$$
$$[\text{pMek}](t=0) = \text{init\_pMek} \quad \text{conc. [nM]} \tag{32}$$
$$[\text{ppMek}](t=0) = \text{init\_ppMek} \quad \text{conc. [nM]} \tag{33}$$
$$[\text{pErk}](t=0) = \text{init\_pErk} \quad \text{conc. [nM]} \tag{34}$$
$$[\text{mTORa}](t=0) = \text{init\_mTORa} \quad \text{conc. [nM]} \tag{35}$$
$$[\text{TORC1}](t=0) = \text{init\_TORC1} \quad \text{conc. [nM]} \tag{36}$$
$$[\text{TORC2}](t=0) = \text{init\_TORC2} \quad \text{conc. [nM]} \tag{37}$$
$$[\text{RSKa}](t=0) = \text{init\_RSKa} \quad \text{conc. [nM]} \tag{38}$$
$$[\text{pS6}](t=0) = \text{init\_pS6} \quad \text{conc. [au]} \tag{39}$$
$$[\text{Intperk}](t=0) = \text{init\_Intperk} \quad \text{conc. [nM]} \tag{40}$$

$$[\text{Intpakt}](t=0) = \text{init\_Intpakt} \quad \text{conc. [nM]} \tag{41}$$
$$[\text{Proliferation}](t=0) = \text{init\_Proliferation} \quad \text{conc. [au]} \tag{42}$$
$$[\text{IntpS6}](t=0) = \text{init\_IntpS6} \quad \text{conc. [au]} \tag{43}$$
$$[\text{CellCycle}](t=0) = \text{init\_CellCycle} \quad \text{conc. [au]} \tag{44}$$

F.4.2. BaF3-EpoR: Reactions

The model contains 62 reactions:

$$v_1 = [\mathrm{BaF3\_Epo}] \cdot \mathrm{Epo\_degradation} \tag{45}$$

$$v_2 = \frac{[\mathrm{BaF3\_Epo}]^{\mathrm{nb}} \cdot \mathrm{EpoR\_activation1} \cdot \left([\mathrm{EpoR\_BaF3}] - [\mathrm{pEpoR}] \cdot \left(\frac{\mathrm{kPI3K}}{\mathrm{kPI3K}+\mathrm{kPI3K}\cdot\mathrm{kPI3Kras}+1} + \frac{\mathrm{kPI3K}\cdot\mathrm{kPI3Kras}}{\mathrm{kPI3K}+\mathrm{kPI3K}\cdot\mathrm{kPI3Kras}+1} + 1\right)\right)}{\left(\frac{[\mathrm{phos2}]}{\mathrm{Ki1}} + 1\right) \cdot \left([\mathrm{BaF3\_Epo}]^{\mathrm{nb}} + \mathrm{ksatb}^{\mathrm{nb}}\right)} \tag{46}$$

$$v_3 = \mathrm{Phosphatase\_activation1} \cdot [\mathrm{pEpoR}] \cdot ([\mathrm{Phosphataset\_BaF3}] - [\mathrm{actPhosphatase}]) \tag{47}$$

$$v_4 = \mathrm{Phosphatase\_deactivation1} \cdot [\mathrm{actPhosphatase}] \tag{48}$$

$$v_5 = [\mathrm{actPhosphatase}] \cdot [\mathrm{pEpoR}] \cdot \mathrm{pEpoR\_deactivation1} \tag{49}$$

$$v_6 = \frac{\mathrm{Phos\_act1} \cdot [\mathrm{pEpoR}]^{\mathrm{n1}}}{\mathrm{Ji1}^{\mathrm{n1}} + [\mathrm{pEpoR}]^{\mathrm{n1}}} \tag{50}$$

$$v_7 = \mathrm{Phos\_deact1} \cdot [\mathrm{phos2}] \tag{51}$$

$$v_8 = \mathrm{kSHIP1} \cdot ([\mathrm{Rp\_SHIP1}] - [\mathrm{SHIP1t\_BaF3}]) \cdot ([\mathrm{Rp\_SHIP1}] - [\mathrm{pEpoR}]) \tag{52}$$

$$v_9 = [\mathrm{Rp\_SHIP1}] \cdot \mathrm{lSHIP1} \tag{53}$$

$$v_{10} = \frac{[\mathrm{PI45P2\_BaF3}] \cdot \mathrm{kPI3K} \cdot [\mathrm{pEpoR}] \cdot \mathrm{vPIP3}}{\mathrm{kPI3K} + \mathrm{kPI3K} \cdot \mathrm{kPI3Kras} + 1} \tag{54}$$

$$v_{11} = \frac{[\mathrm{PI45P2\_BaF3}] \cdot \mathrm{kPI3K} \cdot \mathrm{kPI3Kras} \cdot \mathrm{main1} \cdot [\mathrm{pEpoR}] \cdot \mathrm{vPIP3}}{\mathrm{kPI3K} + \mathrm{kPI3K} \cdot \mathrm{kPI3Kras} + 1} \tag{55}$$

$$v_{12} = -[\mathrm{PIP3}] \cdot \mathrm{kshbasal} \cdot ([\mathrm{Rp\_SHIP1}] - [\mathrm{SHIP1t\_BaF3}]) \tag{56}$$

$$v_{13} = [\mathrm{PIP3}] \cdot [\mathrm{PTEN\_BaF3}] \cdot \mathrm{vPI45P2} \tag{57}$$

$$v_{14} = [\mathrm{PIP3}] \cdot [\mathrm{Rp\_SHIP1}] \cdot \mathrm{vPI34P2} \tag{58}$$

$$v_{15} = [\mathrm{PIP3}] \cdot \mathrm{kPDK1} \cdot ([\mathrm{PDK1t\_BaF3}] - [\mathrm{PIP3\_PDK1}]) \tag{59}$$

$$v_{16} = [\mathrm{PIP3\_PDK1}] \cdot \mathrm{lPDK1} \tag{60}$$

$$v_{17} = -[\mathrm{PIP3}] \cdot \mathrm{kAkt} \cdot ([\mathrm{PIP3\_Akt}] - [\mathrm{Aktt\_BaF3}] + [\mathrm{pAkt}]) \tag{61}$$

$$v_{18} = [\mathrm{PIP3\_Akt}] \cdot \mathrm{lAkt} \tag{62}$$

$$v_{19} = [\mathrm{PIP3\_Akt}] \cdot [\mathrm{PIP3\_PDK1}] \cdot \mathrm{vpAkt} \tag{63}$$

$$v_{20} = [\mathrm{PIP3\_Akt}] \cdot [\mathrm{PIP3\_PDK1}] \cdot \mathrm{vpAkt} \tag{64}$$

$$v_{21} = \frac{[\mathrm{PIP3\_Akt}] \cdot [\mathrm{PIP3\_PDK1}] \cdot \mathrm{vpAkt}}{\mathrm{Aktin} \cdot \mathrm{kaktinb} + 1.0} \tag{65}$$

$$v_{22} = \mathrm{dpAkt} \cdot [\mathrm{pAkt}] \tag{66}$$

$$v_{23} = -[\mathrm{PI45P2\_BaF3}] \cdot \mathrm{vPIP3\_basal} \cdot \left(\frac{\mathrm{kPI3K} \cdot [\mathrm{pEpoR}]}{\mathrm{kPI3K} + \mathrm{kPI3K} \cdot \mathrm{kPI3Kras} + 1} - [\mathrm{PI3Kt\_BaF3}] + \frac{\mathrm{kPI3K} \cdot \mathrm{kPI3Kras} \cdot [\mathrm{pEpoR}]}{\mathrm{kPI3K} + \mathrm{kPI3K} \cdot \mathrm{kPI3Kras} + 1}\right) \tag{67}$$

$$v_{24} = [\mathrm{PI45P2\_BaF3}] \cdot \mathrm{main1} \cdot \mathrm{vPIP3\_basal} \tag{68}$$

$$v_{25} = -\mathrm{kpes} \cdot [\mathrm{ppErk}] \cdot ([\mathrm{Rp\_Sos}] - \mathrm{Sostb} + [\mathrm{pSos}]) \tag{69}$$

$$v_{26} = \mathrm{kdpes} \cdot [\mathrm{pSos}] \tag{70}$$

$$v_{27} = \mathrm{kSos} \cdot ([\mathrm{Rp\_Sos}] - [\mathrm{pEpoR}]) \cdot ([\mathrm{Rp\_Sos}] - \mathrm{Sostb} + [\mathrm{pSos}]) \tag{71}$$

$$v_{28} = [\mathrm{Rp\_Sos}] \cdot \mathrm{lSos} \tag{72}$$

$$v_{29} = [\mathrm{Rp\_Sos}] \cdot \mathrm{kpes} \cdot [\mathrm{ppErk}] \tag{73}$$

$$v_{30} = -\mathrm{k\_back\_Ras} \cdot \left([\mathrm{GTP\_Ras}] - [\mathrm{Rast\_BaF3}] + \frac{\mathrm{kPI3K} \cdot \mathrm{kPI3Kras} \cdot [\mathrm{pEpoR}]}{\mathrm{kPI3K} + \mathrm{kPI3K} \cdot \mathrm{kPI3Kras} + 1}\right) \tag{74}$$

$$v_{31} = -[\mathrm{Rp\_Sos}] \cdot \mathrm{kaRas} \cdot \left([\mathrm{GTP\_Ras}] - [\mathrm{Rast\_BaF3}] + \frac{\mathrm{kPI3K} \cdot \mathrm{kPI3Kras} \cdot [\mathrm{pEpoR}]}{\mathrm{kPI3K} + \mathrm{kPI3K} \cdot \mathrm{kPI3Kras} + 1}\right) \tag{75}$$

$$v_{32} = [\mathrm{GTP\_Ras}] \cdot \mathrm{kdRas} \tag{76}$$

$$v_{33} = \mathrm{kpRaf} \cdot \left([\mathrm{GTP\_Ras}] + \frac{\mathrm{kPI3K} \cdot \mathrm{kPI3Kras} \cdot [\mathrm{pEpoR}]}{\mathrm{kPI3K} + \mathrm{kPI3K} \cdot \mathrm{kPI3Kras} + 1}\right) \cdot ([\mathrm{Raft\_BaF3}] - [\mathrm{pRaf}]) \tag{77}$$

$$v_{34} = \mathrm{lpRaf} \cdot [\mathrm{pRaf}] \tag{78}$$

$$v_{35} = -\mathrm{kpMek} \cdot [\mathrm{pRaf}] \cdot ([\mathrm{pMek}] - [\mathrm{Mekt\_BaF3}] + [\mathrm{ppMek}]) \tag{79}$$

$$v_{36} = \mathrm{lpMek} \cdot [\mathrm{pMek}] \tag{80}$$

$$v_{37} = \mathrm{kppMek} \cdot [\mathrm{pMek}] \cdot [\mathrm{pRaf}] \tag{81}$$

$$v_{38} = \text{lppMek} \cdot [\text{ppMek}] \tag{82}$$
$$v_{39} = -\text{kpErk} \cdot [\text{ppMek}] \cdot ([\text{pErk}] - [\text{Erkt\_BaF3}] + [\text{ppErk}]) \tag{83}$$
$$v_{40} = \text{bDUSP3} \cdot \text{lpErk} \cdot [\text{pErk}] \tag{84}$$
$$v_{41} = \frac{\text{kppErk} \cdot [\text{pErk}] \cdot [\text{ppMek}]}{\text{Mekin} \cdot \text{kmekinb} + 1.0} \tag{85}$$
$$v_{42} = \text{bDUSP3} \cdot \text{lppErk} \cdot [\text{ppErk}] \tag{86}$$
$$v_{43} = [\text{ppErk}] \tag{87}$$
$$v_{44} = [\text{pAkt}] \tag{88}$$
$$v_{45} = -\text{kvpakt} \cdot [\text{pAkt}] \cdot ([\text{TORC1}] + [\text{TORC2}] + [\text{mTORa}] - [\text{mTORt}]) \tag{89}$$

$$v_{46} = -\frac{[\text{RSKa}] \cdot \text{kvppErk} \cdot ([\text{TORC1}] + [\text{TORC2}] + [\text{mTORa}] - [\text{mTORt}])}{\text{RSKin} \cdot \text{kinrskb} + 1.0} \tag{90}$$
$$v_{47} = \text{kdactm} \cdot [\text{mTORa}] \tag{91}$$
$$v_{48} = -\frac{\text{kst1} \cdot ([\text{Raptorb}] - [\text{TORC1}]) \cdot ([\text{TORC1}] + [\text{TORC2}] - [\text{mTORa}])}{\text{Rapamycin} \cdot \text{kinrapab} + 1.0} \tag{92}$$
$$v_{49} = [\text{TORC1}] \cdot \text{kdt1} \tag{93}$$
$$v_{50} = [\text{TORC1}] \cdot \text{kdactm} \tag{94}$$
$$v_{51} = -\text{kst2} \cdot ([\text{Rictorb}] - [\text{TORC2}]) \cdot ([\text{TORC1}] + [\text{TORC2}] - [\text{mTORa}]) \tag{95}$$
$$v_{52} = [\text{TORC2}] \cdot \text{kdt2} \tag{96}$$
$$v_{53} = [\text{TORC2}] \cdot \text{kdactm} \tag{97}$$
$$v_{54} = -\frac{\text{karsk} \cdot [\text{ppErk}] \cdot ([\text{RSKa}] - [\text{RSKtb}])}{\text{RSKin} \cdot \text{kinrskb} + 1.0} \tag{98}$$
$$v_{55} = [\text{RSKa}] \cdot \text{kdrsk} \tag{99}$$
$$v_{56} = [\text{TORC1}] \cdot \text{kas6t} \cdot ([\text{S6t\_BaF3}] - [\text{pS6}]) \tag{100}$$

$$v_{57} = [\text{RSKa}] \cdot \text{kas6r} \cdot ([\text{S6t\_BaF3}] - [\text{pS6}]) \tag{101}$$
$$v_{58} = \text{kbacks6} \cdot ([\text{S6t\_BaF3}] - [\text{pS6}]) \tag{102}$$
$$v_{59} = \text{kdphoss6} \cdot [\text{pS6}] \tag{103}$$
$$v_{60} = \frac{\text{pboff}}{60} + \frac{\text{pbk1} \cdot (\text{cycleaktinb} \cdot [\text{pAkt}] + \text{cyclemekinb} \cdot [\text{ppErk}])}{\text{maxccb}} \tag{104}$$
$$v_{61} = [\text{pS6}] \tag{105}$$
$$v_{62} = \text{cycleaktinb} \cdot [\text{pAkt}] + \text{cyclemekinb} \cdot [\text{ppErk}] \tag{106}$$

F.4.3. BaF3-EpoR: ODE system

The ODE system determining the time evolution of the dynamical variables is given by:

$$\text{d}[\text{BaF3\_Epo}]/\text{dt} = -v_1 \tag{107}$$
$$\text{d}[\text{EpoR\_BaF3}]/\text{dt} = 0 \tag{108}$$
$$\text{d}[\text{Phosphataset\_BaF3}]/\text{dt} = 0 \tag{109}$$
$$\text{d}[\text{SHIP1t\_BaF3}]/\text{dt} = 0 \tag{110}$$
$$\text{d}[\text{PI3Kt\_BaF3}]/\text{dt} = 0 \tag{111}$$
$$\text{d}[\text{PI45P2\_BaF3}]/\text{dt} = 0 \tag{112}$$
$$\text{d}[\text{PTEN\_BaF3}]/\text{dt} = 0 \tag{113}$$
$$\text{d}[\text{PDK1t\_BaF3}]/\text{dt} = 0 \tag{114}$$
$$\text{d}[\text{Aktt\_BaF3}]/\text{dt} = 0 \tag{115}$$
$$\text{d}[\text{Rast\_BaF3}]/\text{dt} = 0 \tag{116}$$

$$\text{d}[\text{Raft\_BaF3}]/\text{dt} = 0 \tag{117}$$
$$\text{d}[\text{Mekt\_BaF3}]/\text{dt} = 0 \tag{118}$$
$$\text{d}[\text{Erkt\_BaF3}]/\text{dt} = 0 \tag{119}$$
$$\text{d}[\text{S6t\_BaF3}]/\text{dt} = 0 \tag{120}$$
$$\text{d}[\text{mTORt}]/\text{dt} = 0 \tag{121}$$
$$\text{d}[\text{Raptorb}]/\text{dt} = 0 \tag{122}$$
$$\text{d}[\text{Rictorb}]/\text{dt} = 0 \tag{123}$$
$$\text{d}[\text{RSKtb}]/\text{dt} = 0 \tag{124}$$
$$\text{d}[\text{pEpoR}]/\text{dt} = +v_2 - v_5 \tag{125}$$

$$\text{d[phos2]/dt} = +v_6 - v_7 \quad (126)$$

$$\text{d[actPhosphatase]/dt} = +v_3 - v_4 \quad (127)$$
$$\text{d[GTP\_Ras]/dt} = +v_{30} + v_{31} - v_{32} \quad (128)$$
$$\text{d[Rp\_SHIP1]/dt} = +v_8 - v_9 \quad (129)$$
$$\text{d[PIP3]/dt} = +v_{10} + v_{11} - v_{12} - v_{13} - v_{14} - v_{15} + v_{16} - v_{17} + v_{18} + v_{20} + v_{23} + v_{24} \quad (130)$$
$$\text{d[PIP3\_PDK1]/dt} = +v_{15} - v_{16} \quad (131)$$
$$\text{d[PIP3\_Akt]/dt} = +v_{17} - v_{18} - v_{19} \quad (132)$$
$$\text{d[pAkt]/dt} = +v_{21} - v_{22} \quad (133)$$
$$\text{d[pSos]/dt} = +v_{25} - v_{26} + v_{29} \quad (134)$$
$$\text{d[Rp\_Sos]/dt} = +v_{27} - v_{28} - v_{29} \quad (135)$$
$$\text{d[ppErk]/dt} = +v_{41} - v_{42} \quad (136)$$

$$\text{d[pRaf]/dt} = +v_{33} - v_{34} \quad (137)$$
$$\text{d[pMek]/dt} = +v_{35} - v_{36} - v_{37} + v_{38} \quad (138)$$
$$\text{d[ppMek]/dt} = +v_{37} - v_{38} \quad (139)$$
$$\text{d[pErk]/dt} = +v_{39} - v_{40} - v_{41} + v_{42} \quad (140)$$
$$\text{d[mTORa]/dt} = +v_{45} + v_{46} - v_{47} - v_{48} + v_{49} - v_{51} + v_{52} \quad (141)$$
$$\text{d[TORC1]/dt} = +v_{48} - v_{49} - v_{50} \quad (142)$$
$$\text{d[TORC2]/dt} = +v_{51} - v_{52} - v_{53} \quad (143)$$
$$\text{d[RSKa]/dt} = +v_{54} - v_{55} \quad (144)$$
$$\text{d[pS6]/dt} = +v_{56} + v_{57} + v_{58} - v_{59} \quad (145)$$
$$\text{d[Intperk]/dt} = +v_{43} \quad (146)$$

$$\text{d[Intpakt]/dt} = +v_{44} \quad (147)$$
$$\text{d[Proliferation]/dt} = +v_{60} \quad (148)$$
$$\text{d[IntpS6]/dt} = +v_{61} \quad (149)$$
$$\text{d[CellCycle]/dt} = +v_{62} \quad (150)$$

F.4.4. mCFU-E: Dynamic variables

The model contains 44 dynamic variables:

$$\text{[Epo](t = 0)} = \text{init\_Epo} \quad \text{conc. [nM]} \quad (151)$$
$$\text{[EpoR\_CFUE](t = 0)} = \text{init\_EpoR\_CFUE} \quad \text{conc. [nM]} \quad (152)$$
$$\text{[Phosphataset\_CFUE](t = 0)} = \text{init\_Phosphataset\_CFUE} \quad \text{conc. [nM]} \quad (153)$$
$$\text{[SHIP1t\_CFUE](t = 0)} = \text{init\_SHIP1t\_CFUE} \quad \text{conc. [nM]} \quad (154)$$
$$\text{[PI3Kt\_CFUE](t = 0)} = \text{init\_PI3Kt\_CFUE} \quad \text{conc. [nM]} \quad (155)$$
$$\text{[PI45P2\_CFUE](t = 0)} = \text{init\_PI45P2\_CFUE} \quad \text{conc. [nM]} \quad (156)$$
$$\text{[PTEN\_CFUE](t = 0)} = \text{init\_PTEN\_CFUE} \quad \text{conc. [nM]} \quad (157)$$
$$\text{[PDK1t\_CFUE](t = 0)} = \text{init\_PDK1t\_CFUE} \quad \text{conc. [nM]} \quad (158)$$
$$\text{[Aktt\_CFUE](t = 0)} = \text{init\_Aktt\_CFUE} \quad \text{conc. [nM]} \quad (159)$$
$$\text{[Rast\_CFUE](t = 0)} = \text{init\_Rast\_CFUE} \quad \text{conc. [nM]} \quad (160)$$

$$\text{[Raft\_CFUE](t = 0)} = \text{init\_Raft\_CFUE} \quad \text{conc. [nM]} \quad (161)$$
$$\text{[Mekt\_CFUE](t = 0)} = \text{init\_Mekt\_CFUE} \quad \text{conc. [nM]} \quad (162)$$
$$\text{[Erkt\_CFUE](t = 0)} = \text{init\_Erkt\_CFUE} \quad \text{conc. [nM]} \quad (163)$$
$$\text{[S6t\_CFUE](t = 0)} = \text{init\_S6t\_CFUE} \quad \text{conc. [nM]} \quad (164)$$
$$\text{[mTORt](t = 0)} = \text{init\_mTORt} \quad \text{conc. [nM]} \quad (165)$$
$$\text{[Raptorc](t = 0)} = \text{init\_Raptorc} \quad \text{conc. [nM]} \quad (166)$$
$$\text{[Rictorc](t = 0)} = \text{init\_Rictorc} \quad \text{conc. [nM]} \quad (167)$$
$$\text{[RSKtc](t = 0)} = \text{init\_RSKtc} \quad \text{conc. [nM]} \quad (168)$$
$$\text{[pEpoR](t = 0)} = \text{init\_pEpoR} \quad \text{conc. [nM]} \quad (169)$$
$$\text{[phos2](t = 0)} = \text{init\_phos2} \quad \text{conc. [nM]} \quad (170)$$

$$[\text{actPhosphatase}](t=0) = \text{init\_actPhosphatase} \quad \text{conc. [nM]} \tag{171}$$
$$[\text{GTP\_Ras}](t=0) = \text{init\_GTP\_Ras} \quad \text{conc. [nM]} \tag{172}$$
$$[\text{Rp\_SHIP1}](t=0) = \text{init\_Rp\_SHIP1} \quad \text{conc. [nM]} \tag{173}$$
$$[\text{PIP3}](t=0) = \text{init\_PIP3} \quad \text{conc. [nM]} \tag{174}$$
$$[\text{PIP3\_PDK1}](t=0) = \text{init\_PIP3\_PDK1} \quad \text{conc. [nM]} \tag{175}$$
$$[\text{PIP3\_Akt}](t=0) = \text{init\_PIP3\_Akt} \quad \text{conc. [nM]} \tag{176}$$
$$[\text{pAkt}](t=0) = \text{init\_pAkt} \quad \text{conc. [nM]} \tag{177}$$
$$[\text{pSos}](t=0) = \text{init\_pSos} \quad \text{conc. [nM]} \tag{178}$$
$$[\text{Rp\_Sos}](t=0) = \text{init\_Rp\_Sos} \quad \text{conc. [nM]} \tag{179}$$
$$[\text{ppErk}](t=0) = \text{init\_ppErk} \quad \text{conc. [nM]} \tag{180}$$

$$[\text{pRaf}](t=0) = \text{init\_pRaf} \quad \text{conc. [nM]} \tag{181}$$
$$[\text{pMek}](t=0) = \text{init\_pMek} \quad \text{conc. [nM]} \tag{182}$$
$$[\text{ppMek}](t=0) = \text{init\_ppMek} \quad \text{conc. [nM]} \tag{183}$$
$$[\text{pErk}](t=0) = \text{init\_pErk} \quad \text{conc. [nM]} \tag{184}$$
$$[\text{mTORa}](t=0) = \text{init\_mTORa} \quad \text{conc. [nM]} \tag{185}$$
$$[\text{TORC1}](t=0) = \text{init\_TORC1} \quad \text{conc. [nM]} \tag{186}$$
$$[\text{TORC2}](t=0) = \text{init\_TORC2} \quad \text{conc. [nM]} \tag{187}$$
$$[\text{RSKa}](t=0) = \text{init\_RSKa} \quad \text{conc. [nM]} \tag{188}$$
$$[\text{pS6}](t=0) = \text{init\_pS6} \quad \text{conc. [au]} \tag{189}$$

$$[\text{Intperk}](t=0) = \text{init\_Intperk} \quad \text{conc. [nM]} \tag{190}$$
$$[\text{Intpakt}](t=0) = \text{init\_Intpakt} \quad \text{conc. [nM]} \tag{191}$$
$$[\text{Proliferation}](t=0) = \text{init\_Proliferation} \quad \text{conc. [au]} \tag{192}$$
$$[\text{IntpS6}](t=0) = \text{init\_IntpS6} \quad \text{conc. [au]} \tag{193}$$
$$[\text{CellCycle}](t=0) = \text{init\_CellCycle} \quad \text{conc. [au]} \tag{194}$$

F.4.5. mCFU-E: Reactions

The model contains 61 reactions:

$$v_1 = \frac{[\text{Epo}]^{\text{nc}} \cdot \text{EpoR\_activation} \cdot \left([\text{EpoR\_CFUE}] - [\text{pEpoR}] \cdot \left(\frac{\text{kPI3K}}{\text{kPI3K}+\text{kPI3K}\cdot\text{kPI3Kras}+1} + \frac{\text{kPI3K}\cdot\text{kPI3Kras}}{\text{kPI3K}+\text{kPI3K}\cdot\text{kPI3Kras}+1} + 1\right)\right)}{\left(\frac{[\text{phos2}]}{\text{Ki}} + 1\right) \cdot \left([\text{Epo}]^{\text{nc}} + \text{ksatc}^{\text{nc}}\right)} \tag{195}$$

$$v_2 = \text{Phosphatase\_activation} \cdot [\text{pEpoR}] \cdot ([\text{Phosphataset\_CFUE}] - [\text{actPhosphatase}]) \tag{196}$$
$$v_3 = \text{Phosphatase\_deactivation} \cdot [\text{actPhosphatase}] \tag{197}$$
$$v_4 = [\text{actPhosphatase}] \cdot [\text{pEpoR}] \cdot \text{pEpoR\_deactivation} \tag{198}$$
$$v_5 = \frac{\text{Phos\_act} \cdot [\text{pEpoR}]^{\text{n}}}{\text{Ji}^{\text{n}} + [\text{pEpoR}]^{\text{n}}} \tag{199}$$
$$v_6 = \text{Phos\_deact} \cdot [\text{phos2}] \tag{200}$$
$$v_7 = \text{kSHIP1} \cdot ([\text{Rp\_SHIP1}] - [\text{SHIP1t\_CFUE}]) \cdot ([\text{Rp\_SHIP1}] - [\text{pEpoR}]) \tag{201}$$
$$v_8 = [\text{Rp\_SHIP1}] \cdot \text{lSHIP1} \tag{202}$$
$$v_9 = \frac{[\text{PI45P2\_CFUE}] \cdot \text{kPI3K} \cdot [\text{pEpoR}] \cdot \text{vPIP3}}{\text{kPI3K} + \text{kPI3K} \cdot \text{kPI3Kras} + 1} \tag{203}$$
$$v_{10} = \frac{[\text{PI45P2\_CFUE}] \cdot \text{kPI3K} \cdot \text{kPI3Kras} \cdot \text{main} \cdot [\text{pEpoR}] \cdot \text{vPIP3}}{\text{kPI3K} + \text{kPI3K} \cdot \text{kPI3Kras} + 1} \tag{204}$$
$$v_{11} = -[\text{PIP3}] \cdot \text{kshbasal} \cdot ([\text{Rp\_SHIP1}] - [\text{SHIP1t\_CFUE}]) \tag{205}$$
$$v_{12} = [\text{PIP3}] \cdot [\text{PTEN\_CFUE}] \cdot \text{vPI45P2} \tag{206}$$

$$v_{13} = [\text{PIP3}] \cdot [\text{Rp\_SHIP1}] \cdot \text{vPI34P2} \tag{207}$$
$$v_{14} = [\text{PIP3}] \cdot \text{kPDK1} \cdot ([\text{PDK1t\_CFUE}] - [\text{PIP3\_PDK1}]) \tag{208}$$
$$v_{15} = [\text{PIP3\_PDK1}] \cdot \text{lPDK1} \tag{209}$$
$$v_{16} = -[\text{PIP3}] \cdot \text{kAkt} \cdot ([\text{PIP3\_Akt}] - [\text{Aktt\_CFUE}] + [\text{pAkt}]) \tag{210}$$
$$v_{17} = [\text{PIP3\_Akt}] \cdot \text{lAkt} \tag{211}$$

$$v_{18} = [\text{PIP3\_Akt}] \cdot [\text{PIP3\_PDK1}] \cdot \text{vpAkt} \quad (212)$$
$$v_{19} = [\text{PIP3\_Akt}] \cdot [\text{PIP3\_PDK1}] \cdot \text{vpAkt} \quad (213)$$
$$v_{20} = \frac{[\text{PIP3\_Akt}] \cdot [\text{PIP3\_PDK1}] \cdot \text{vpAkt}}{\text{Aktin} \cdot \text{kaktinc} + 1.0} \quad (214)$$
$$v_{21} = \text{dpAkt} \cdot [\text{pAkt}] \quad (215)$$

$$v_{22} = -[\text{PI45P2\_CFUE}] \cdot \text{vPIP3\_basal} \cdot \left(\frac{\text{kPI3K} \cdot [\text{pEpoR}]}{\text{kPI3K} + \text{kPI3K} \cdot \text{kPI3Kras} + 1} - [\text{PI3Kt\_CFUE}] + \frac{\text{kPI3K} \cdot \text{kPI3Kras} \cdot [\text{pEpoR}]}{\text{kPI3K} + \text{kPI3K} \cdot \text{kPI3Kras} + 1}\right) \quad (216)$$

$$v_{23} = [\text{PI45P2\_CFUE}] \cdot \text{main} \cdot \text{vPIP3\_basal} \quad (217)$$
$$v_{24} = -\text{kpes} \cdot [\text{ppErk}] \cdot ([\text{Rp\_Sos}] - \text{Sostc} + [\text{pSos}]) \quad (218)$$
$$v_{25} = \text{kdpes} \cdot [\text{pSos}] \quad (219)$$
$$v_{26} = \text{kSos} \cdot ([\text{Rp\_Sos}] - [\text{pEpoR}]) \cdot ([\text{Rp\_Sos}] - \text{Sostc} + [\text{pSos}]) \quad (220)$$
$$v_{27} = [\text{Rp\_Sos}] \cdot \text{lSos} \quad (221)$$
$$v_{28} = [\text{Rp\_Sos}] \cdot \text{kpes} \cdot [\text{ppErk}] \quad (222)$$
$$v_{29} = -\text{k\_back\_Ras} \cdot \left([\text{GTP\_Ras}] - [\text{Rast\_CFUE}] + \frac{\text{kPI3K} \cdot \text{kPI3Kras} \cdot [\text{pEpoR}]}{\text{kPI3K} + \text{kPI3K} \cdot \text{kPI3Kras} + 1}\right) \quad (223)$$
$$v_{30} = -[\text{Rp\_Sos}] \cdot \text{kaRas} \cdot \left([\text{GTP\_Ras}] - [\text{Rast\_CFUE}] + \frac{\text{kPI3K} \cdot \text{kPI3Kras} \cdot [\text{pEpoR}]}{\text{kPI3K} + \text{kPI3K} \cdot \text{kPI3Kras} + 1}\right) \quad (224)$$
$$v_{31} = [\text{GTP\_Ras}] \cdot \text{kdRas} \quad (225)$$
$$v_{32} = \text{kpRaf} \cdot \left([\text{GTP\_Ras}] + \frac{\text{kPI3K} \cdot \text{kPI3Kras} \cdot [\text{pEpoR}]}{\text{kPI3K} + \text{kPI3K} \cdot \text{kPI3Kras} + 1}\right) \cdot ([\text{Raft\_CFUE}] - [\text{pRaf}]) \quad (226)$$
$$v_{33} = \text{lpRaf} \cdot [\text{pRaf}] \quad (227)$$

$$v_{34} = -\text{kpMek} \cdot [\text{pRaf}] \cdot ([\text{pMek}] - [\text{Mekt\_CFUE}] + [\text{ppMek}]) \quad (228)$$
$$v_{35} = \text{lpMek} \cdot [\text{pMek}] \quad (229)$$
$$v_{36} = \text{kppMek} \cdot [\text{pMek}] \cdot [\text{pRaf}] \quad (230)$$
$$v_{37} = \text{lppMek} \cdot [\text{ppMek}] \quad (231)$$
$$v_{38} = -\text{kpErk} \cdot [\text{ppMek}] \cdot ([\text{pErk}] - [\text{Erkt\_CFUE}] + [\text{ppErk}]) \quad (232)$$
$$v_{39} = \text{cDUSP3} \cdot \text{lpErk} \cdot [\text{pErk}] \quad (233)$$
$$v_{40} = \frac{\text{kppErk} \cdot [\text{pErk}] \cdot [\text{ppMek}]}{\text{Mekin} \cdot \text{kmekinc} + 1.0} \quad (234)$$
$$v_{41} = \text{cDUSP3} \cdot \text{lppErk} \cdot [\text{ppErk}] \quad (235)$$
$$v_{42} = [\text{ppErk}] \quad (236)$$
$$v_{43} = [\text{pAkt}] \quad (237)$$
$$v_{44} = -\text{kvpakt} \cdot [\text{pAkt}] \cdot ([\text{TORC1}] + [\text{TORC2}] + [\text{mTORa}] - [\text{mTORt}]) \quad (238)$$

$$v_{45} = -\frac{[\text{RSKa}] \cdot \text{kvppErk} \cdot ([\text{TORC1}] + [\text{TORC2}] + [\text{mTORa}] - [\text{mTORt}])}{\text{RSKin} \cdot \text{kinrskc} + 1.0} \quad (239)$$
$$v_{46} = \text{kdactm} \cdot [\text{mTORa}] \quad (240)$$
$$v_{47} = -\frac{\text{kst1} \cdot ([\text{Raptorc}] - [\text{TORC1}]) \cdot ([\text{TORC1}] + [\text{TORC2}] - [\text{mTORa}])}{\text{Rapamycin} \cdot \text{kinrapac} + 1.0} \quad (241)$$
$$v_{48} = [\text{TORC1}] \cdot \text{kdt1} \quad (242)$$
$$v_{49} = [\text{TORC1}] \cdot \text{kdactm} \quad (243)$$
$$v_{50} = -\text{kst2} \cdot ([\text{Rictorc}] - [\text{TORC2}]) \cdot ([\text{TORC1}] + [\text{TORC2}] - [\text{mTORa}]) \quad (244)$$
$$v_{51} = [\text{TORC2}] \cdot \text{kdactm} \quad (245)$$
$$v_{52} = [\text{TORC2}] \cdot \text{kdt2} \quad (246)$$
$$v_{53} = -\frac{\text{karsk} \cdot [\text{ppErk}] \cdot ([\text{RSKa}] - [\text{RSKtc}])}{\text{RSKin} \cdot \text{kinrskc} + 1.0} \quad (247)$$
$$v_{54} = [\text{RSKa}] \cdot \text{kdrsk} \quad (248)$$
$$v_{55} = [\text{TORC1}] \cdot \text{kas6t} \cdot ([\text{S6t\_CFUE}] - [\text{pS6}]) \quad (249)$$

$$v_{56} = [\text{RSKa}] \cdot \text{kas6r} \cdot ([\text{S6t\_CFUE}] - [\text{pS6}]) \quad (250)$$
$$v_{57} = \text{kbacks6} \cdot ([\text{S6t\_CFUE}] - [\text{pS6}]) \quad (251)$$
$$v_{58} = \text{kdphoss6} \cdot [\text{pS6}] \quad (252)$$

$$v_{59} = \frac{\text{pcoff}}{60} + \frac{[\text{pS6}] \cdot \text{pck1}}{\text{max6c}} \quad (253)$$
$$v_{60} = [\text{pS6}] \quad (254)$$
$$v_{61} = \text{cycleaktinc} \cdot [\text{pAkt}] + \text{cyclemekinc} \cdot [\text{ppErk}] \quad (255)$$

F.4.6. mCFU-E: ODE system

The ODE system determining the time evolution of the dynamical variables is given by:

$$\text{d[Epo]/dt} = 0 \quad (256)$$
$$\text{d[EpoR\_CFUE]/dt} = 0 \quad (257)$$
$$\text{d[Phosphataset\_CFUE]/dt} = 0 \quad (258)$$
$$\text{d[SHIP1t\_CFUE]/dt} = 0 \quad (259)$$
$$\text{d[PI3Kt\_CFUE]/dt} = 0 \quad (260)$$
$$\text{d[PI45P2\_CFUE]/dt} = 0 \quad (261)$$
$$\text{d[PTEN\_CFUE]/dt} = 0 \quad (262)$$
$$\text{d[PDK1t\_CFUE]/dt} = 0 \quad (263)$$
$$\text{d[Aktt\_CFUE]/dt} = 0 \quad (264)$$
$$\text{d[Rast\_CFUE]/dt} = 0 \quad (265)$$

$$\text{d[Raft\_CFUE]/dt} = 0 \quad (266)$$
$$\text{d[Mekt\_CFUE]/dt} = 0 \quad (267)$$
$$\text{d[Erkt\_CFUE]/dt} = 0 \quad (268)$$
$$\text{d[S6t\_CFUE]/dt} = 0 \quad (269)$$
$$\text{d[mTORt]/dt} = 0 \quad (270)$$
$$\text{d[Raptorc]/dt} = 0 \quad (271)$$
$$\text{d[Rictorc]/dt} = 0 \quad (272)$$
$$\text{d[RSKtc]/dt} = 0 \quad (273)$$
$$\text{d[pEpoR]/dt} = +v_1 - v_4 \quad (274)$$
$$\text{d[phos2]/dt} = +v_5 - v_6 \quad (275)$$

$$\text{d[actPhosphatase]/dt} = +v_2 - v_3 \quad (276)$$
$$\text{d[GTP\_Ras]/dt} = +v_{29} + v_{30} - v_{31} \quad (277)$$
$$\text{d[Rp\_SHIP1]/dt} = +v_7 - v_8 \quad (278)$$
$$\text{d[PIP3]/dt} = +v_9 + v_{10} - v_{11} - v_{12} - v_{13} - v_{14} + v_{15} - v_{16} + v_{17} + v_{19} + v_{22} + v_{23} \quad (279)$$
$$\text{d[PIP3\_PDK1]/dt} = +v_{14} - v_{15} \quad (280)$$
$$\text{d[PIP3\_Akt]/dt} = +v_{16} - v_{17} - v_{18} \quad (281)$$
$$\text{d[pAkt]/dt} = +v_{20} - v_{21} \quad (282)$$
$$\text{d[pSos]/dt} = +v_{24} - v_{25} + v_{28} \quad (283)$$
$$\text{d[Rp\_Sos]/dt} = +v_{26} - v_{27} - v_{28} \quad (284)$$
$$\text{d[ppErk]/dt} = +v_{40} - v_{41} \quad (285)$$

$$\text{d[pRaf]/dt} = +v_{32} - v_{33} \quad (286)$$
$$\text{d[pMek]/dt} = +v_{34} - v_{35} - v_{36} + v_{37} \quad (287)$$
$$\text{d[ppMek]/dt} = +v_{36} - v_{37} \quad (288)$$
$$\text{d[pErk]/dt} = +v_{38} - v_{39} - v_{40} + v_{41} \quad (289)$$
$$\text{d[mTORa]/dt} = +v_{44} + v_{45} - v_{46} - v_{47} + v_{48} - v_{50} + v_{52} \quad (290)$$
$$\text{d[TORC1]/dt} = +v_{47} - v_{48} - v_{49} \quad (291)$$
$$\text{d[TORC2]/dt} = +v_{50} - v_{51} - v_{52} \quad (292)$$
$$\text{d[RSKa]/dt} = +v_{53} - v_{54} \quad (293)$$
$$\text{d[pS6]/dt} = +v_{55} + v_{56} + v_{57} - v_{58} \quad (294)$$
$$\text{d[Intperk]/dt} = +v_{42} \quad (295)$$

$$\text{d[Intpakt]/dt} = +v_{43} \quad (296)$$
$$\text{d[Proliferation]/dt} = +v_{59} \quad (297)$$

$$d[IntpS6]/dt = +v_{60} \tag{298}$$
$$d[CellCycle]/dt = +v_{61} \tag{299}$$

F.4.7. 32D-EpoR: Dynamic variables

The model contains 44 dynamic variables:

$$[BaF3\_Epo](t=0) = init\_BaF3\_Epo \quad conc. [nM] \tag{300}$$
$$[EpoR\_32D](t=0) = init\_EpoR\_32D \quad conc. [nM] \tag{301}$$
$$[Phosphataset\_32D](t=0) = init\_Phosphataset\_32D \quad conc. [nM] \tag{302}$$
$$[SHIP1t\_32D](t=0) = init\_SHIP1t\_32D \quad conc. [nM] \tag{303}$$
$$[PI3Kt\_32D](t=0) = init\_PI3Kt\_32D \quad conc. [nM] \tag{304}$$
$$[PI45P2\_32D](t=0) = init\_PI45P2\_32D \quad conc. [nM] \tag{305}$$
$$[PTEN\_32D](t=0) = init\_PTEN\_32D \quad conc. [nM] \tag{306}$$
$$[PDK1t\_32D](t=0) = init\_PDK1t\_32D \quad conc. [nM] \tag{307}$$
$$[Aktt\_32D](t=0) = init\_Aktt\_32D \quad conc. [nM] \tag{308}$$
$$[Rast\_32D](t=0) = init\_Rast\_32D \quad conc. [nM] \tag{309}$$

$$[Raft\_32D](t=0) = init\_Raft\_32D \quad conc. [nM] \tag{310}$$
$$[Mekt\_32D](t=0) = init\_Mekt\_32D \quad conc. [nM] \tag{311}$$
$$[Erkt\_32D](t=0) = init\_Erkt\_32D \quad conc. [nM] \tag{312}$$
$$[S6t\_32D](t=0) = init\_S6t\_32D \quad conc. [nM] \tag{313}$$
$$[mTORt](t=0) = init\_mTORt \quad conc. [nM] \tag{314}$$
$$[Raptord](t=0) = init\_Raptord \quad conc. [nM] \tag{315}$$
$$[Rictord](t=0) = init\_Rictord \quad conc. [nM] \tag{316}$$
$$[RSKtd](t=0) = init\_RSKtd \quad conc. [nM] \tag{317}$$
$$[pEpoR](t=0) = init\_pEpoR \quad conc. [nM] \tag{318}$$
$$[phos2](t=0) = init\_phos2 \quad conc. [nM] \tag{319}$$

$$[actPhosphatase](t=0) = init\_actPhosphatase \quad conc. [nM] \tag{320}$$
$$[GTP\_Ras](t=0) = init\_GTP\_Ras \quad conc. [nM] \tag{321}$$
$$[Rp\_SHIP1](t=0) = init\_Rp\_SHIP1 \quad conc. [nM] \tag{322}$$
$$[PIP3](t=0) = init\_PIP3 \quad conc. [nM] \tag{323}$$
$$[PIP3\_PDK1](t=0) = init\_PIP3\_PDK1 \quad conc. [nM] \tag{324}$$
$$[PIP3\_Akt](t=0) = init\_PIP3\_Akt \quad conc. [nM] \tag{325}$$
$$[pAkt](t=0) = init\_pAkt \quad conc. [nM] \tag{326}$$
$$[pSos](t=0) = init\_pSos \quad conc. [nM] \tag{327}$$
$$[Rp\_Sos](t=0) = init\_Rp\_Sos \quad conc. [nM] \tag{328}$$
$$[ppErk](t=0) = init\_ppErk \quad conc. [nM] \tag{329}$$

$$[pRaf](t=0) = init\_pRaf \quad conc. [nM] \tag{330}$$
$$[pMek](t=0) = init\_pMek \quad conc. [nM] \tag{331}$$
$$[ppMek](t=0) = init\_ppMek \quad conc. [nM] \tag{332}$$
$$[pErk](t=0) = init\_pErk \quad conc. [nM] \tag{333}$$
$$[mTORa](t=0) = init\_mTORa \quad conc. [nM] \tag{334}$$
$$[TORC1](t=0) = init\_TORC1 \quad conc. [nM] \tag{335}$$
$$[TORC2](t=0) = init\_TORC2 \quad conc. [nM] \tag{336}$$
$$[RSKa](t=0) = init\_RSKa \quad conc. [nM] \tag{337}$$
$$[pS6](t=0) = init\_pS6 \quad conc. [au] \tag{338}$$
$$[Intperk](t=0) = init\_Intperk \quad conc. [nM] \tag{339}$$

$$[Intpakt](t=0) = init\_Intpakt \quad conc. [nM] \tag{340}$$
$$[Proliferation](t=0) = init\_Proliferation \quad conc. [au] \tag{341}$$
$$[IntpS6](t=0) = init\_IntpS6 \quad conc. [au] \tag{342}$$
$$[CellCycle](t=0) = init\_CellCycle \quad conc. [au] \tag{343}$$

F.4.8. 32D-EpoR: Reactions

The model contains 62 reactions:

$$v_1 = [\text{BaF3\_Epo}] \cdot \text{Epo\_degradation} \quad (344)$$

$$v_2 = \frac{[\text{BaF3\_Epo}]^{\text{nb}} \cdot \text{EpoR\_activation1} \cdot \left([\text{EpoR\_32D}] - [\text{pEpoR}] \cdot \left(\frac{\text{kPI3K}}{\text{kPI3K}+\text{kPI3K}\cdot\text{kPI3Kras}+1} + \frac{\text{kPI3K}\cdot\text{kPI3Kras}}{\text{kPI3K}+\text{kPI3K}\cdot\text{kPI3Kras}+1} + 1\right)\right)}{\left(\frac{[\text{phos2}]}{\text{Ki1}} + 1\right) \cdot \left([\text{BaF3\_Epo}]^{\text{nb}} + \text{ksatb}^{\text{nb}}\right)} \quad (345)$$

$$v_3 = \text{Phosphatase\_activation1} \cdot [\text{pEpoR}] \cdot ([\text{Phosphataset\_32D}] - [\text{actPhosphatase}]) \quad (346)$$

$$v_4 = \text{Phosphatase\_deactivation1} \cdot [\text{actPhosphatase}] \quad (347)$$

$$v_5 = [\text{actPhosphatase}] \cdot [\text{pEpoR}] \cdot \text{pEpoR\_deactivation1} \quad (348)$$

$$v_6 = \frac{\text{Phos\_act1} \cdot [\text{pEpoR}]^{\text{n1}}}{\text{Ji1}^{\text{n1}} + [\text{pEpoR}]^{\text{n1}}} \quad (349)$$

$$v_7 = \text{Phos\_deact1} \cdot [\text{phos2}] \quad (350)$$

$$v_8 = \text{kSHIP1} \cdot ([\text{Rp\_SHIP1}] - [\text{SHIP1t\_32D}]) \cdot ([\text{Rp\_SHIP1}] - [\text{pEpoR}]) \quad (351)$$

$$v_9 = [\text{Rp\_SHIP1}] \cdot \text{lSHIP1} \quad (352)$$

$$v_{10} = \frac{[\text{PI45P2\_32D}] \cdot \text{kPI3K} \cdot [\text{pEpoR}] \cdot \text{vPIP3}}{\text{kPI3K} + \text{kPI3K} \cdot \text{kPI3Kras} + 1} \quad (353)$$

$$v_{11} = \frac{[\text{PI45P2\_32D}] \cdot \text{kPI3K} \cdot \text{kPI3Kras} \cdot \text{main1} \cdot [\text{pEpoR}] \cdot \text{vPIP3}}{\text{kPI3K} + \text{kPI3K} \cdot \text{kPI3Kras} + 1} \quad (354)$$

$$v_{12} = -[\text{PIP3}] \cdot \text{kshbasal} \cdot ([\text{Rp\_SHIP1}] - [\text{SHIP1t\_32D}]) \quad (355)$$

$$v_{13} = [\text{PIP3}] \cdot [\text{PTEN\_32D}] \cdot \text{vPI45P2} \quad (356)$$

$$v_{14} = [\text{PIP3}] \cdot [\text{Rp\_SHIP1}] \cdot \text{vPI34P2} \quad (357)$$

$$v_{15} = [\text{PIP3}] \cdot \text{kPDK1} \cdot ([\text{PDK1t\_32D}] - [\text{PIP3\_PDK1}]) \quad (358)$$

$$v_{16} = [\text{PIP3\_PDK1}] \cdot \text{lPDK1} \quad (359)$$

$$v_{17} = -[\text{PIP3}] \cdot \text{kAkt} \cdot ([\text{PIP3\_Akt}] - [\text{Aktt\_32D}] + [\text{pAkt}]) \quad (360)$$

$$v_{18} = [\text{PIP3\_Akt}] \cdot \text{lAkt} \quad (361)$$

$$v_{19} = [\text{PIP3\_Akt}] \cdot [\text{PIP3\_PDK1}] \cdot \text{vpAkt} \quad (362)$$

$$v_{20} = [\text{PIP3\_Akt}] \cdot [\text{PIP3\_PDK1}] \cdot \text{vpAkt} \quad (363)$$

$$v_{21} = \frac{[\text{PIP3\_Akt}] \cdot [\text{PIP3\_PDK1}] \cdot \text{vpAkt}}{\text{Aktin} \cdot \text{kaktind} + 1.0} \quad (364)$$

$$v_{22} = \text{dpAkt} \cdot [\text{pAkt}] \quad (365)$$

$$v_{23} = -[\text{PI45P2\_32D}] \cdot \text{vPIP3\_basal} \cdot \left(\frac{\text{kPI3K} \cdot [\text{pEpoR}]}{\text{kPI3K} + \text{kPI3K} \cdot \text{kPI3Kras} + 1} - [\text{PI3Kt\_32D}] + \frac{\text{kPI3K} \cdot \text{kPI3Kras} \cdot [\text{pEpoR}]}{\text{kPI3K} + \text{kPI3K} \cdot \text{kPI3Kras} + 1}\right) \quad (366)$$

$$v_{24} = [\text{PI45P2\_32D}] \cdot \text{main1} \cdot \text{vPIP3\_basal} \quad (367)$$

$$v_{25} = -\text{kpes} \cdot [\text{ppErk}] \cdot ([\text{Rp\_Sos}] - \text{Sostb} + [\text{pSos}]) \quad (368)$$

$$v_{26} = \text{kdpes} \cdot [\text{pSos}] \quad (369)$$

$$v_{27} = \text{kSos} \cdot ([\text{Rp\_Sos}] - [\text{pEpoR}]) \cdot ([\text{Rp\_Sos}] - \text{Sostb} + [\text{pSos}]) \quad (370)$$

$$v_{28} = [\text{Rp\_Sos}] \cdot \text{lSos} \quad (371)$$

$$v_{29} = [\text{Rp\_Sos}] \cdot \text{kpes} \cdot [\text{ppErk}] \quad (372)$$

$$v_{30} = -\text{k\_back\_Ras} \cdot \left([\text{GTP\_Ras}] - [\text{Rast\_32D}] + \frac{\text{kPI3K} \cdot \text{kPI3Kras} \cdot [\text{pEpoR}]}{\text{kPI3K} + \text{kPI3K} \cdot \text{kPI3Kras} + 1}\right) \quad (373)$$

$$v_{31} = -[\text{Rp\_Sos}] \cdot \text{kaRas} \cdot \left([\text{GTP\_Ras}] - [\text{Rast\_32D}] + \frac{\text{kPI3K} \cdot \text{kPI3Kras} \cdot [\text{pEpoR}]}{\text{kPI3K} + \text{kPI3K} \cdot \text{kPI3Kras} + 1}\right) \quad (374)$$

$$v_{32} = [\text{GTP\_Ras}] \cdot \text{kdRas} \quad (375)$$

$$v_{33} = \text{kpRaf} \cdot \left([\text{GTP\_Ras}] + \frac{\text{kPI3K} \cdot \text{kPI3Kras} \cdot [\text{pEpoR}]}{\text{kPI3K} + \text{kPI3K} \cdot \text{kPI3Kras} + 1}\right) \cdot ([\text{Raft\_32D}] - [\text{pRaf}]) \quad (376)$$

$$v_{34} = \text{lpRaf} \cdot [\text{pRaf}] \quad (377)$$

$$v_{35} = -\text{kpMek} \cdot [\text{pRaf}] \cdot ([\text{pMek}] - [\text{Mekt\_32D}] + [\text{ppMek}]) \quad (378)$$

$$v_{36} = \text{lpMek} \cdot [\text{pMek}] \quad (379)$$

$$v_{37} = \text{kppMek} \cdot [\text{pMek}] \cdot [\text{pRaf}] \quad (380)$$

$$v_{38} = \mathrm{lppMek} \cdot [\mathrm{ppMek}] \tag{381}$$
$$v_{39} = -\mathrm{kpErk} \cdot [\mathrm{ppMek}] \cdot ([\mathrm{pErk}] - [\mathrm{Erkt\_32D}] + [\mathrm{ppErk}]) \tag{382}$$
$$v_{40} = \mathrm{dDUSP3} \cdot \mathrm{lpErk} \cdot [\mathrm{pErk}] \tag{383}$$
$$v_{41} = \frac{\mathrm{kppErk} \cdot [\mathrm{pErk}] \cdot [\mathrm{ppMek}]}{\mathrm{Mekin} \cdot \mathrm{kmekind} + 1.0} \tag{384}$$
$$v_{42} = \mathrm{dDUSP3} \cdot \mathrm{lppErk} \cdot [\mathrm{ppErk}] \tag{385}$$
$$v_{43} = [\mathrm{ppErk}] \tag{386}$$
$$v_{44} = [\mathrm{pAkt}] \tag{387}$$
$$v_{45} = -\mathrm{kvpakt} \cdot [\mathrm{pAkt}] \cdot ([\mathrm{TORC1}] + [\mathrm{TORC2}] + [\mathrm{mTORa}] - [\mathrm{mTORt}]) \tag{388}$$

$$v_{46} = -\frac{[\mathrm{RSKa}] \cdot \mathrm{kvppErk} \cdot ([\mathrm{TORC1}] + [\mathrm{TORC2}] + [\mathrm{mTORa}] - [\mathrm{mTORt}])}{\mathrm{RSKin} \cdot \mathrm{kinrskb} + 1.0} \tag{389}$$
$$v_{47} = \mathrm{kdactm} \cdot [\mathrm{mTORa}] \tag{390}$$
$$v_{48} = -\frac{\mathrm{kst1} \cdot ([\mathrm{Raptord}] - [\mathrm{TORC1}]) \cdot ([\mathrm{TORC1}] + [\mathrm{TORC2}] - [\mathrm{mTORa}])}{\mathrm{Rapamycin} \cdot \mathrm{kinrapab} + 1.0} \tag{391}$$
$$v_{49} = [\mathrm{TORC1}] \cdot \mathrm{kdt1} \tag{392}$$
$$v_{50} = [\mathrm{TORC1}] \cdot \mathrm{kdactm} \tag{393}$$
$$v_{51} = -\mathrm{kst2} \cdot ([\mathrm{Rictord}] - [\mathrm{TORC2}]) \cdot ([\mathrm{TORC1}] + [\mathrm{TORC2}] - [\mathrm{mTORa}]) \tag{394}$$
$$v_{52} = [\mathrm{TORC2}] \cdot \mathrm{kdt2} \tag{395}$$
$$v_{53} = [\mathrm{TORC2}] \cdot \mathrm{kdactm} \tag{396}$$
$$v_{54} = -\frac{\mathrm{karsk} \cdot [\mathrm{ppErk}] \cdot ([\mathrm{RSKa}] - [\mathrm{RSKtd}])}{\mathrm{RSKin} \cdot \mathrm{kinrskb} + 1.0} \tag{397}$$
$$v_{55} = [\mathrm{RSKa}] \cdot \mathrm{kdrsk} \tag{398}$$
$$v_{56} = [\mathrm{TORC1}] \cdot \mathrm{kas6t} \cdot ([\mathrm{S6t\_32D}] - [\mathrm{pS6}]) \tag{399}$$

$$v_{57} = [\mathrm{RSKa}] \cdot \mathrm{kas6r} \cdot ([\mathrm{S6t\_32D}] - [\mathrm{pS6}]) \tag{400}$$
$$v_{58} = \mathrm{kbacks6} \cdot ([\mathrm{S6t\_32D}] - [\mathrm{pS6}]) \tag{401}$$
$$v_{59} = \mathrm{kdphoss6} \cdot [\mathrm{pS6}] \tag{402}$$
$$v_{60} = \frac{\mathrm{pdoff}}{60} + \frac{\mathrm{pdk1} \cdot (\mathrm{cycleaktind} \cdot [\mathrm{pAkt}] + \mathrm{cyclemekind} \cdot [\mathrm{ppErk}])}{\mathrm{maxccd}} \tag{403}$$
$$v_{61} = [\mathrm{pS6}] \tag{404}$$
$$v_{62} = \mathrm{cycleaktind} \cdot [\mathrm{pAkt}] + \mathrm{cyclemekind} \cdot [\mathrm{ppErk}] \tag{405}$$

F.4.9. 32D-EpoR: ODE system

The ODE system determining the time evolution of the dynamical variables is given by:

$$\mathrm{d[BaF3\_Epo]/dt} = -v_1 \tag{406}$$
$$\mathrm{d[EpoR\_32D]/dt} = 0 \tag{407}$$
$$\mathrm{d[Phosphataset\_32D]/dt} = 0 \tag{408}$$
$$\mathrm{d[SHIP1t\_32D]/dt} = 0 \tag{409}$$
$$\mathrm{d[PI3Kt\_32D]/dt} = 0 \tag{410}$$
$$\mathrm{d[PI45P2\_32D]/dt} = 0 \tag{411}$$
$$\mathrm{d[PTEN\_32D]/dt} = 0 \tag{412}$$
$$\mathrm{d[PDK1t\_32D]/dt} = 0 \tag{413}$$
$$\mathrm{d[Aktt\_32D]/dt} = 0 \tag{414}$$
$$\mathrm{d[Rast\_32D]/dt} = 0 \tag{415}$$

$$\mathrm{d[Raft\_32D]/dt} = 0 \tag{416}$$
$$\mathrm{d[Mekt\_32D]/dt} = 0 \tag{417}$$
$$\mathrm{d[Erkt\_32D]/dt} = 0 \tag{418}$$
$$\mathrm{d[S6t\_32D]/dt} = 0 \tag{419}$$
$$\mathrm{d[mTORt]/dt} = 0 \tag{420}$$
$$\mathrm{d[Raptord]/dt} = 0 \tag{421}$$
$$\mathrm{d[Rictord]/dt} = 0 \tag{422}$$
$$\mathrm{d[RSKtd]/dt} = 0 \tag{423}$$

$$d[\mathrm{pEpoR}]/dt = +v_2 - v_5 \quad (424)$$
$$d[\mathrm{phos2}]/dt = +v_6 - v_7 \quad (425)$$

$$d[\mathrm{actPhosphatase}]/dt = +v_3 - v_4 \quad (426)$$
$$d[\mathrm{GTP\_Ras}]/dt = +v_{30} + v_{31} - v_{32} \quad (427)$$
$$d[\mathrm{Rp\_SHIP1}]/dt = +v_8 - v_9 \quad (428)$$
$$d[\mathrm{PIP3}]/dt = +v_{10} + v_{11} - v_{12} - v_{13} - v_{14} - v_{15} + v_{16} - v_{17} + v_{18} + v_{20} + v_{23} + v_{24} \quad (429)$$
$$d[\mathrm{PIP3\_PDK1}]/dt = +v_{15} - v_{16} \quad (430)$$
$$d[\mathrm{PIP3\_Akt}]/dt = +v_{17} - v_{18} - v_{19} \quad (431)$$
$$d[\mathrm{pAkt}]/dt = +v_{21} - v_{22} \quad (432)$$
$$d[\mathrm{pSos}]/dt = +v_{25} - v_{26} + v_{29} \quad (433)$$
$$d[\mathrm{Rp\_Sos}]/dt = +v_{27} - v_{28} - v_{29} \quad (434)$$
$$d[\mathrm{ppErk}]/dt = +v_{41} - v_{42} \quad (435)$$

$$d[\mathrm{pRaf}]/dt = +v_{33} - v_{34} \quad (436)$$
$$d[\mathrm{pMek}]/dt = +v_{35} - v_{36} - v_{37} + v_{38} \quad (437)$$
$$d[\mathrm{ppMek}]/dt = +v_{37} - v_{38} \quad (438)$$
$$d[\mathrm{pErk}]/dt = +v_{39} - v_{40} - v_{41} + v_{42} \quad (439)$$
$$d[\mathrm{mTORa}]/dt = +v_{45} + v_{46} - v_{47} - v_{48} + v_{49} - v_{51} + v_{52} \quad (440)$$
$$d[\mathrm{TORC1}]/dt = +v_{48} - v_{49} - v_{50} \quad (441)$$
$$d[\mathrm{TORC2}]/dt = +v_{51} - v_{52} - v_{53} \quad (442)$$
$$d[\mathrm{RSKa}]/dt = +v_{54} - v_{55} \quad (443)$$
$$d[\mathrm{pS6}]/dt = +v_{56} + v_{57} + v_{58} - v_{59} \quad (444)$$
$$d[\mathrm{Intperk}]/dt = +v_{43} \quad (445)$$

$$d[\mathrm{Intpakt}]/dt = +v_{44} \quad (446)$$
$$d[\mathrm{Proliferation}]/dt = +v_{60} \quad (447)$$
$$d[\mathrm{IntpS6}]/dt = +v_{61} \quad (448)$$
$$d[\mathrm{CellCycle}]/dt = +v_{62} \quad (449)$$

The ODE system was solved by a parallelized implementation of the CVODES algorithm (Hindmarsh et al. 2005). It also supplies the parameter sensitivities utilized for parameter estimation.

G. Analysis of Epo-induced changes and steady-state levels of signaling components

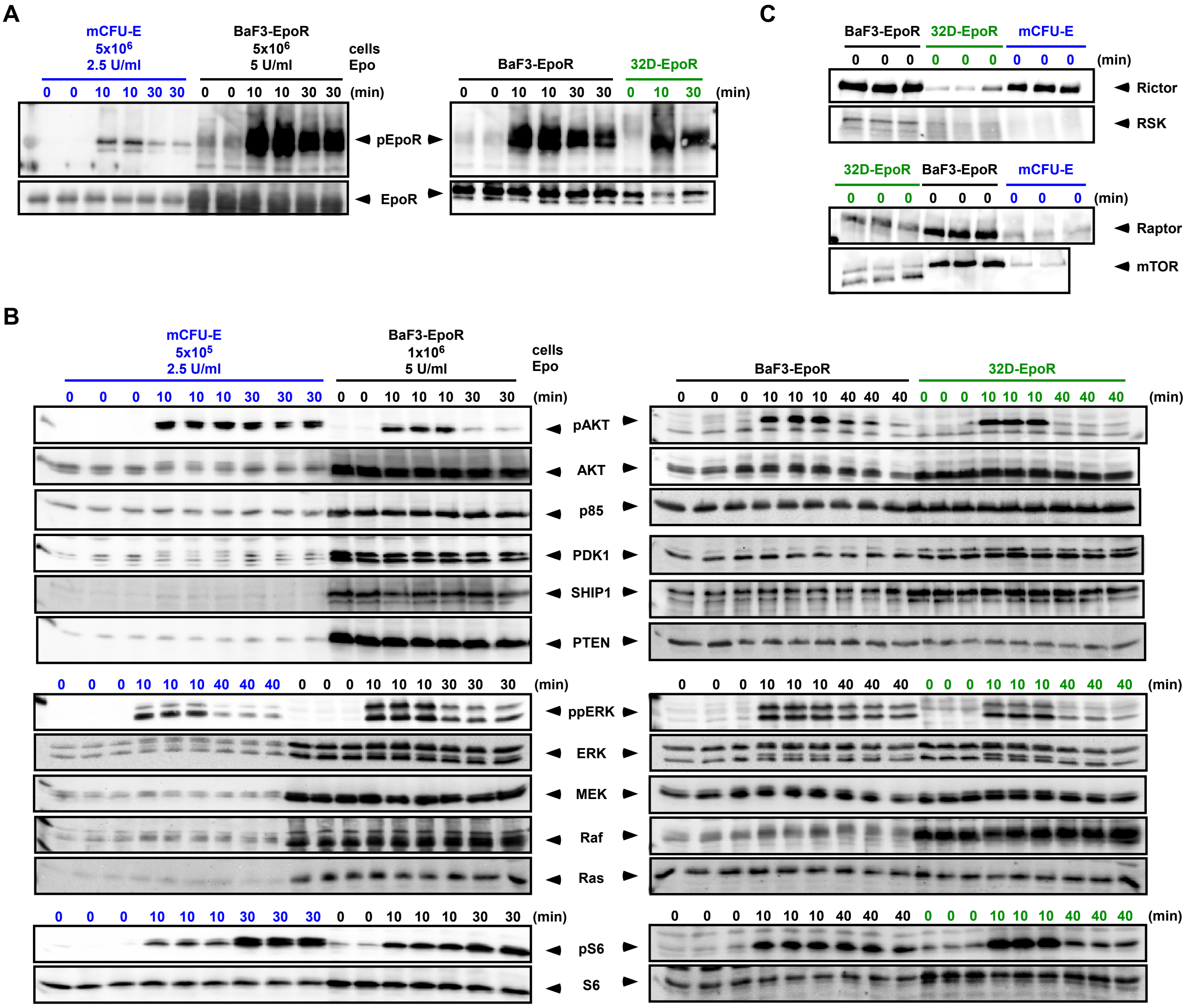

Figure S7: Analysis of Epo-induced changes and steady-state levels of signaling components in BaF3-EpoR, mCFU-E and 32D-EpoR cells. Unless indicated otherwise 5×10^5 growth-factor deprived cells were stimulated with 50 U/ml Epo for indicated time points. Cellular lysates were used as total cellular lysate for quantitative immunoblotting. Signals were quantified using ImageQuant software and were the basis for calculation of the protein abundance (see **Tab. 1**). (A) Determination of amounts of EpoR and pEpoR in mCFU-E and BaF3-EpoR and 32D-EpoR cells. (B) Determination of amounts of AKT, PDK1, SHIP1, PTEN, ERK, MEK, Raf, Ras and S6 in 32D-EpoR cells relative to BaF3-EpoR cells. (C) Lysates of 2×10^6 32D-EpoR or BaF3-EpoR cells, or 7×10^6 mCFU-E cells were subjected to immunoprecipitation. Determination of amounts of Rictor, Raptor, RSK and mTOR in BaF3-EpoR and 32D-EpoR cells relative to mCFU-E cells.

H. Epo depletion by mCFU-E and BaF3-EpoR cells

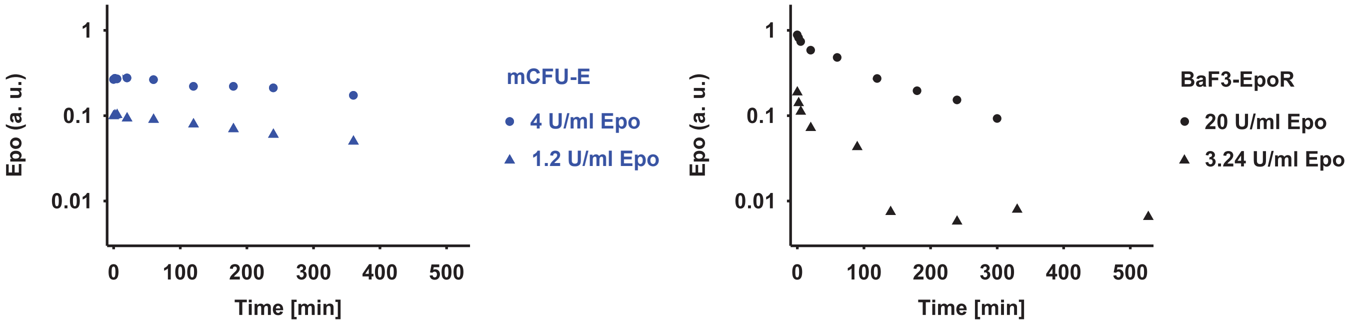

Figure S8: Epo depletion by mCFU-E and BaF3-EpoR cells. Dynamics of free Epo in the presence of mCFU-E cells (left panel) or BaF3-EpoR cells (right panel). mCFU-E cells were washed three times and starved for 1 h at a concentration of 1×10^6 cells/ml in PANSERIN 601, containing 1 % BSA. BaF3-EpoR were washed three times and starved for 3 h at a concentration of 1×10^6 cells/ml in RPMI 1640, containing 1 % penicillin/streptomycin and 1 % BSA. After starvation, cells were adjusted to a final concentration of 4×10^7 cells/ml in 350 µl. Cells were stimulated with Epo at 37°C and 90 rpm in a thermomixer. At indicated time points, cells were centrifuged at 4°C and the supernatant was stored at -80°C. Measurement of Epo in the supernatant was performed by ELISA using the Quantikine IVD ELISA Kit (R&D DEP00).

I. Epo-induced activation of EpoR, GTP-Ras, AKT, ERK and S6 in mCFU-E and BaF3-EpoR cells for model calibration

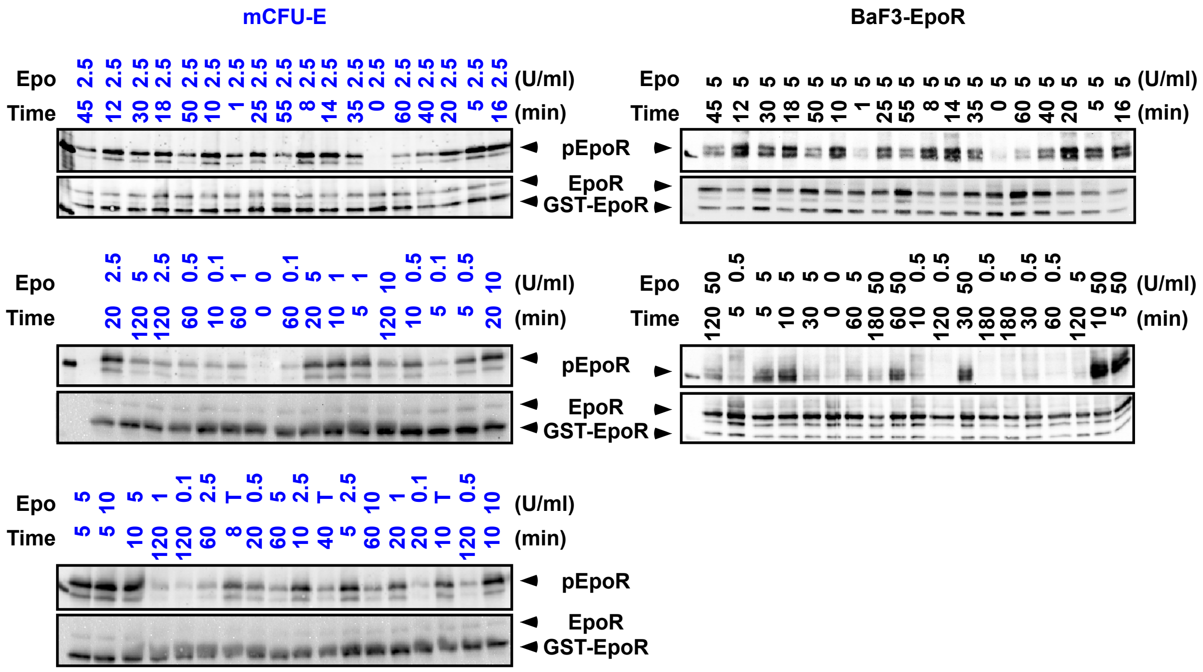

Figure S9: Dynamics of Epo-induced EpoR phosphorylation in mCFU-E and BaF3-EpoR cells. 5×10^5 mCFU-E or 1×10^6 BaF3-EpoR cells were growth-factor deprived and stimulated with indicated Epo doses for indicated times. The recombinant protein of the cytoplasmic domain of the EpoR fused to a GST tag (EpoR-GST) served as calibrator. EpoR and calibrator were co-immunoprecipitated from cellular lysates and subjected to quantitative immunoblotting. The measurements shown in the top panel were used for model calibration. The measurements in the middle and the bottom panel represent independent repetitions. *T* indicates samples generated in mCFU-E cells transduced with the empty pMOWS vector. Sample order was randomized to reduce correlated error.

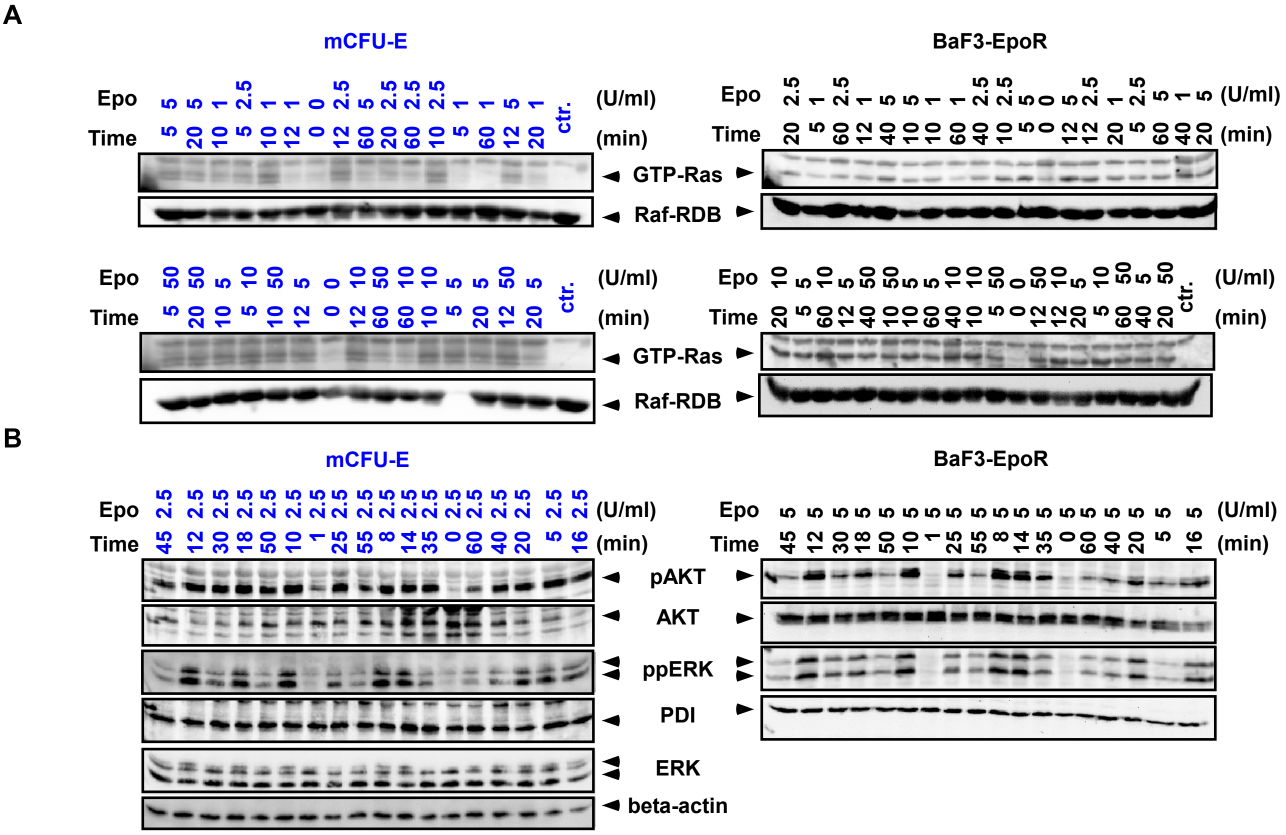

Figure S10: Epo-induced dynamics of GTP-Ras, pAKT and ppERK in mCFU-E and BaF3-EpoR cells. (A) Determination of Ras activation by GST-Raf pulldown in each 5×10^5 mCFU-E or 1×10^6 BaF3-EpoR cells stimulated with Epo. GTP-Ras was eluted from cell lysates after pulldown using a fusion protein harboring GST fused to the Ras binding domain of Raf-1 (Raf-RDB). The levels of GTP-Ras and Raf-RBD (loading control) in cellular lysates were determined by quantitative immunoblotting. *ctr.* indicates Raf-RBD pulldown without cellular lysates as control. (B) The levels of pAKT Ser473, total AKT, ppERK and total ERK in each 5×10^5 mCFU-E or 1×10^6 BaF3-EpoR cells stimulated with Epo were determined by quantitative immunoblotting. PDI and beta-actin served as loading control.

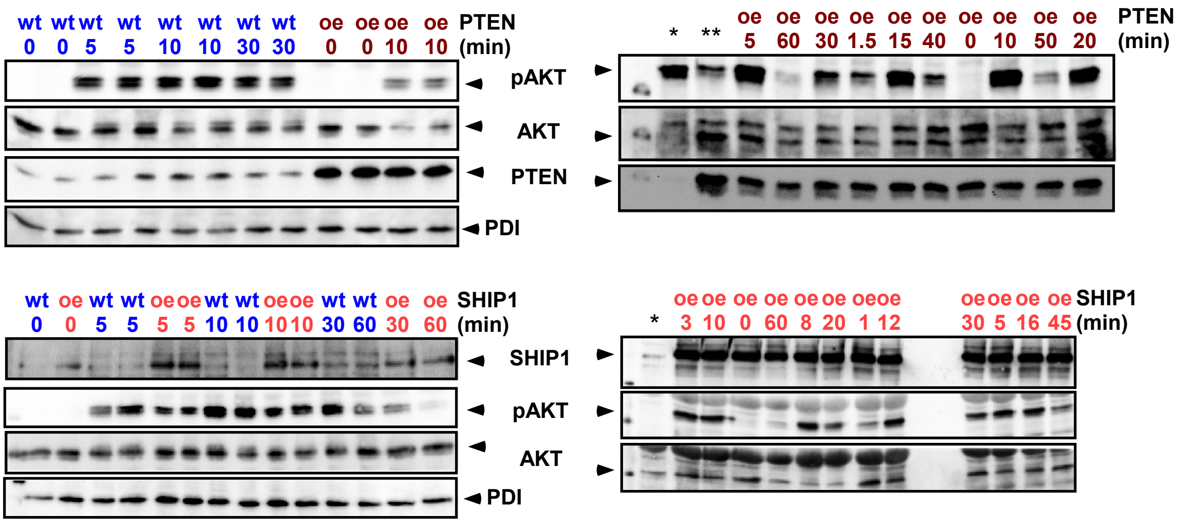

Figure S11: Overexpression of PTEN and SHIP1 in mCFU-E cells. mCFU-E cells retrovirally transduced with PTEN, SHIP1 or vector control were stimulated with 2.5 U/ml Epo for the indicated times. Effects of PTEN overexpression (upper panel) or SHIP1 overexpression (lower panel) on pAKT Ser473 and Thr308, and the levels of PTEN and SHIP1 were determined by quantitative immunoblotting of each 5×10^5 mCFU-E cells. PDI served as loading control. $*$ indicates a non-transduced sample as negative control, $**$ indicates a PTEN-transduced sample from another independent experiment as positive control for transduction. *wt*: wild type, *oe*: overexpression.

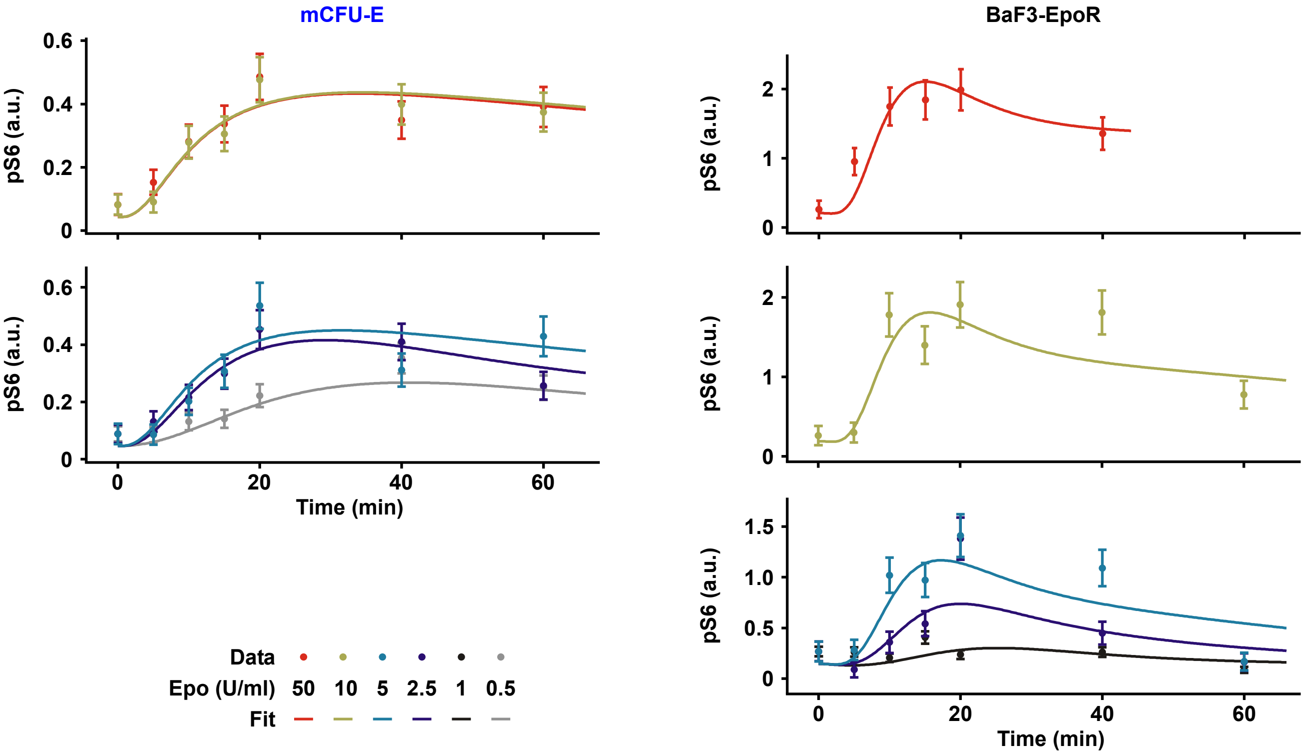

Figure S12: Model calibration with experimental data of Epo-induced dynamics of pS6 in mCFU-E and BaF3-EpoR cells. Growth-factor deprived cells were stimulated with indicated (color-coded) Epo doses for indicated times, and cellular lysates were subjected to quantitative immunoblotting. Filled circles indicate quantitative experimental data, error bars reflect standard deviations derived from the error model, solid lines represent model trajectories.

J. Experimental overexpression of SHIP1 and PTEN in BaF3-EpoR cells and simulation of knockdown

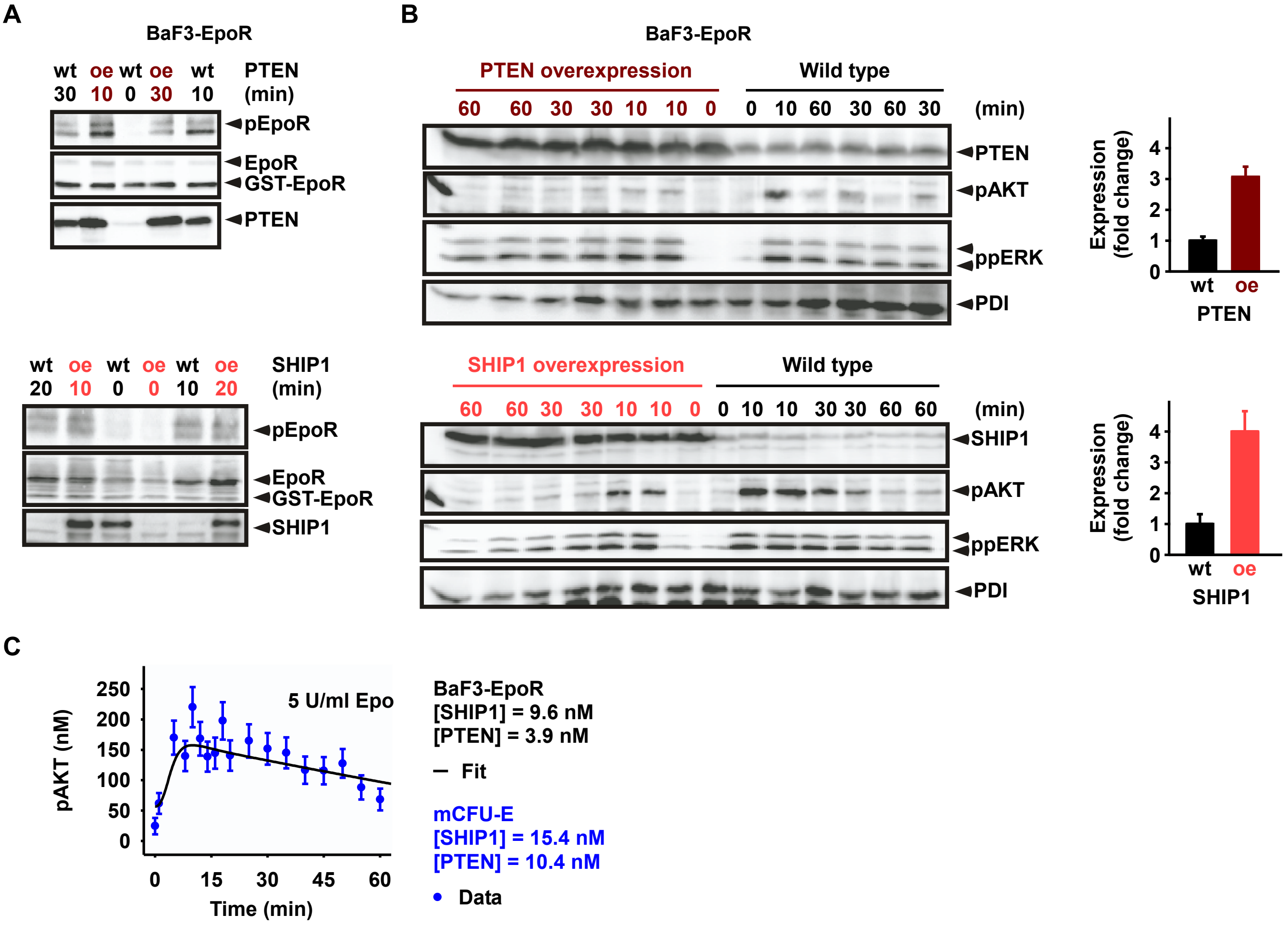

Figure S13: Impact of SHIP1 and PTEN overexpression on AKT phosphorylation in BaF3-EpoR cells. Each 1×10^7 growth-factor deprived cells were stimulated with 5 U/ml Epo for indicated times. (A)PTEN overexpression (upper panel) or SHIP1 overexpression (lower panel) in BaF3-EpoR cells yielded pEpoR levels similar to wild-type BaF3-EpoR cells. The recombinant protein of the cytoplasmic domain of the EpoR fused to a GST tag (EpoR-GST) was used as a calibrator. EpoR, EpoR-GST and either PTEN or SHIP1 were co-immunoprecipitated from cellular lysates. GST-EpoR served as loading control. (B) Time-course of BaF3-EpoR cells either overexpressing PTEN (upper panel), SHIP1 (lower panel) or the empty pMOWS vector as control to determine the effects of PTEN and SHIP1 overexpression on pAKT and ppERK determined by quantitative immunoblotting. PDI served as a loading control. Levels of PTEN and SHIP1 are depicted as bar charts. Overexpression of PTEN and SHIP1 was quantified and remained constant over the time-course experiment. *wt*: wild type, *oe*: overexpression. (C) To achieve in BaF3-EpoR cells pAKT levels comparable to pAKT in mCFU-E cells, protein abundance of SHIP1 and PTEN in BaF3-EpoR cells were estimated with the mathematical model. Best fit between experimental data of pAKT in CFU-E cells was achieved for the mathematical model adapted to protein abundance present in BaF3-EpoR cells with estimated [PTEN] = 9.6 nM (i.e. 27.4-fold decrease) and [SHIP1] = 3.9 nM (8.7-fold decrease).

K. Basal mRNA expression of DUSP family members in mCFU-E and BaF3-EpoR cells

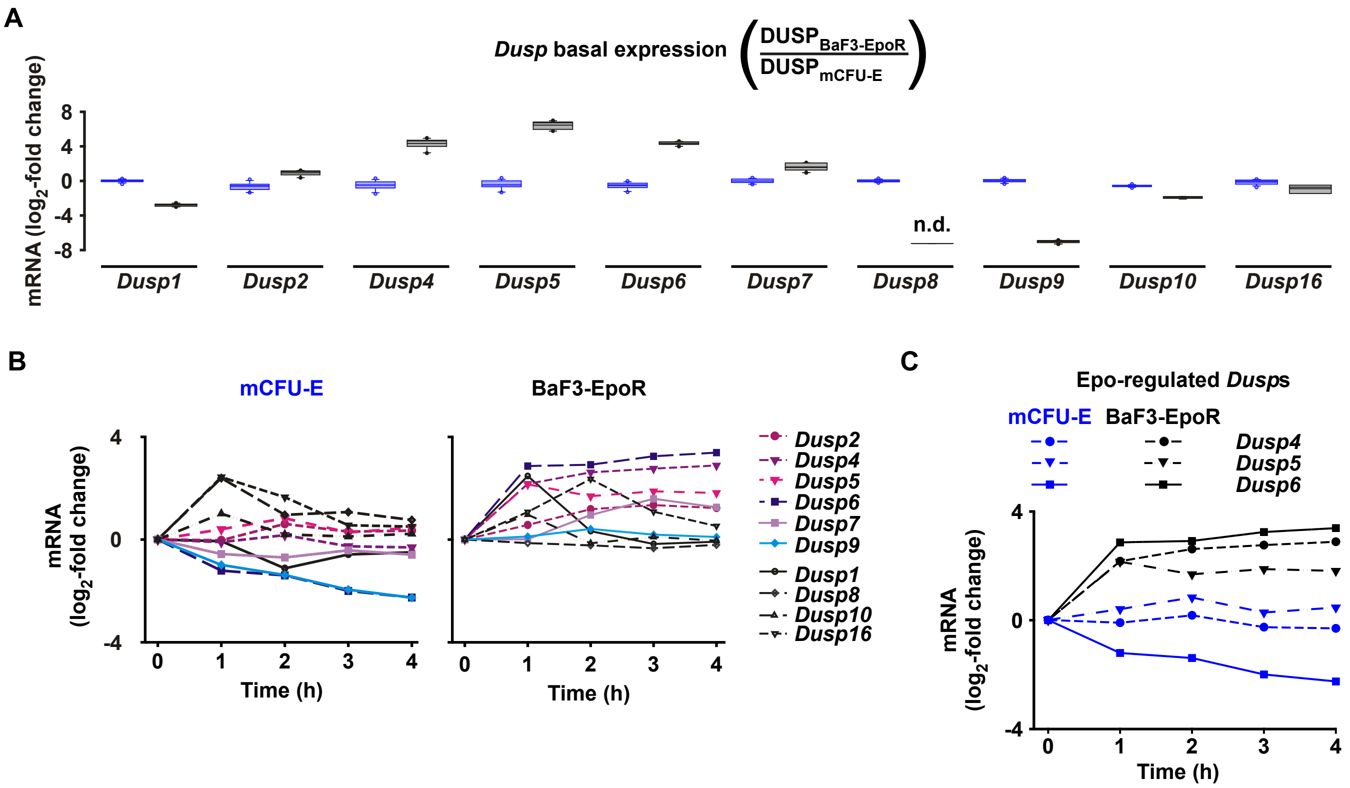

Figure S14: Basal mRNA expression of DUSP family members in mCFU-E and BaF3-EpoR cells. Per condition, total RNA from 3×10^6 cells was isolated. (A) Validation of gene expression patterns of DUSP family members from the microarray analysis by quantitative RT-PCR. 1 µg RNA of the growth-factor deprived mCFU-E or BaF3-EpoR cells from 3 or 4 independent experiments were converted to cDNA by reverse transcription. Analysis of DUSP family members was performed with all samples on the same plate allowing a direct comparison of the mRNA levels between mCFU-E and BaF3-EpoR cells. mRNA expression represented as $\log_2$-fold change of DUSP level in BaF3-EpoR cells relative to mCFU-E cells. *n.d.* not detected. (B) Expression profiling of time-resolved Epo-induced DUSP genes. Growth-factor deprived mCFU-E and BaF3-EpoR cells were stimulated with 1 U/ml Epo. RNA was subjected to microarray analysis and profiles of known DUSP genes were compiled. $\log_2$-fold change of mRNA levels was calculated relative to mean gene expression at time point 0 h. (C) Same data as shown in (B) but time-resolved expression of only *Dusp4*, *Dusp5* and *Dusp6* in mCFU-E and BaF3-EpoR cells.

L. Comparison of AKT, ERK and S6 activation dynamics in mCFU-E, BaF3-EpoR and 32D-EpoR cells by model simulations

The model predictions for the signaling dynamics of 32D-EpoR cells in response to Epo stimulation were in good agreement with experimental data for ppERK and pS6 (see **Fig. 4A**). For pAKT, the peak time and signal duration were predicted correctly while the model overestimated the peak amplitude and steady state of pAKT. The comparison between model and data for 32D-EpoR cells involved no further fitting but relied on the measured protein abundance.
Given the similarities in protein abundance of BaF3-EpoR and 32D-EpoR cells (see **Fig. 8C**), one could assume similar signaling dynamics for the two cell types. We simulated the activation of AKT, ERK and S6 in response to 50 U/ml Epo for mCFU-E, BaF3-EpoR and 32D-EpoR cells with the mathematical model adapted to the protein abundance of mCFU-E, BaF3-EpoR and 32D-EpoR cells (**Fig. S12**). Despite similarities in protein abundance, the model predicted differences in peak time, signal duration, peak amplitude and steady state of model trajectories (**Fig. S15A**).

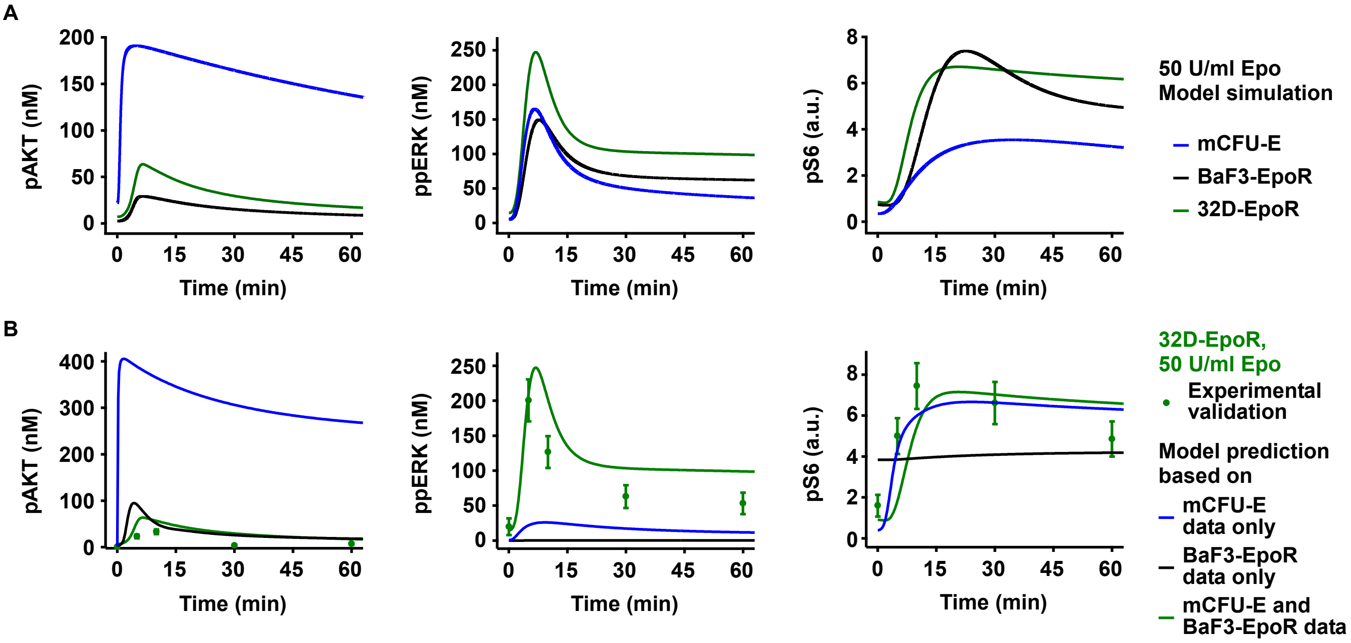

Figure S15: Comparison of AKT, ERK and S6 activation dynamics by model simulations. (A) The mathematical model adapted to the protein abundance of mCFU-E, BaF3-EpoR and 32D-EpoR cells was applied to simulate pAKT, ppERK and pS6 dynamics of mCFU-E, BaF3-EpoR and 32D-EpoR cells in response to 50 U/ml Epo. Dynamics of the trajectories appear qualitatively different, which is caused by the cell-type-specific protein abundance as all kinetic parameters are shared among the three cell types. (B) Comparison of predictions for AKT, ERK and S6 activation dynamics in 32D-EpoR cells in response to 50 U/ml Epo. The mathematical model was adapted to cell-type-specific protein abundance of 32D-EpoR cells and calibrated to experimental data either from only mCFU-E cells or from only BaF3-EpoR cells, or from both, mCFU-E and BaF3-EpoR cells (see **Fig. 4A**).

To test whether the utilization of protein abundance determined for BaF3-EpoR cells could be predictive for Epo-induced activation dynamics of AKT, ERK and S6 measured in 32D-EpoR cells, we fitted the mathematical model adapted to the protein abundance of mCFU-E, BaF3-EpoR and 32D-EpoR cells to the data of Epo-stimulated 32D-EpoR cells shown in **Fig. 4A**. An individual scaling parameter was estimated to compare the shape of simulated signaling dynamics with the experimental data. We found a better agreement between experimental data of pAKT and ppERK dynamics in 32D-EpoR cells with simulations by the mathematical model adapted to the protein abundance of 32D-EpoR cells compared to BaF3-EpoR cells (**Tab. S2**).

Table S2: Comparison of goodness of the fit for model simulations of mCFU-E, BaF3-EpoR and 32D-EpoR cells to experimental data for 32D-EpoR cells. An individual scaling parameter was estimated to each five data points per condition. Given is the χ^2.

Species	mCFU-E	BaF3-EpoR	32D-EpoR
pAKT	38.3	22.4	21.0
ppERK	4.3	10.6	5.6
pS6	28.7	14.7	22.7

Protein abundance of 32D-EpoR cells is therefore predictive of the shape of the Epo-induced activation dynamics of AKT, ERK and S6 in 32D-EpoR cells.
To test the predictive power of the mathematical model calibrated to only a single cell type, we simulated the activation dynamics of AKT, ERK and S6 in 32D-EpoR cells in response to 50 U/ml Epo with a mathematical model only calibrated with experimental data for either mCFU-E or BaF3-EpoR cells (**Fig. S15B**). The mathematical model calibrated with mCFU-E data only predicted a too high amplitude for pAKT dynamics and too low ERK activation in response to Epo stimulation of 32D-EpoR cells but a decent agreement for time-resolved pS6 (overall χ^2=48,009). The mathematical model calibrated with BaF3-EpoR data only predicted a slightly too high and too fast pAKT amplitude and a too low activation of ppERK whereas the dynamic shape of pS6 of 32D-EpoR cells was not in agreement with the experimental data of 32D-EpoR cells in response to 50 U/ml Epo stimulation (overall χ^2=604). Only the mathematical model calibrated with experimental data of both, mCFU-E and BaF3-EpoR cells, was able to predict activation of AKT, ERK and S6 in good agreement with experimental data (overall χ^2=284) even though the pAKT peak amplitude was overestimated as mentioned above (see **Fig. 4A**).

M. Expression of individual cell-cycle genes in mCFU-E, BaF3-EpoR and 32D-EpoR cells in response to Epo stimulation and impact of inhibitor treatment

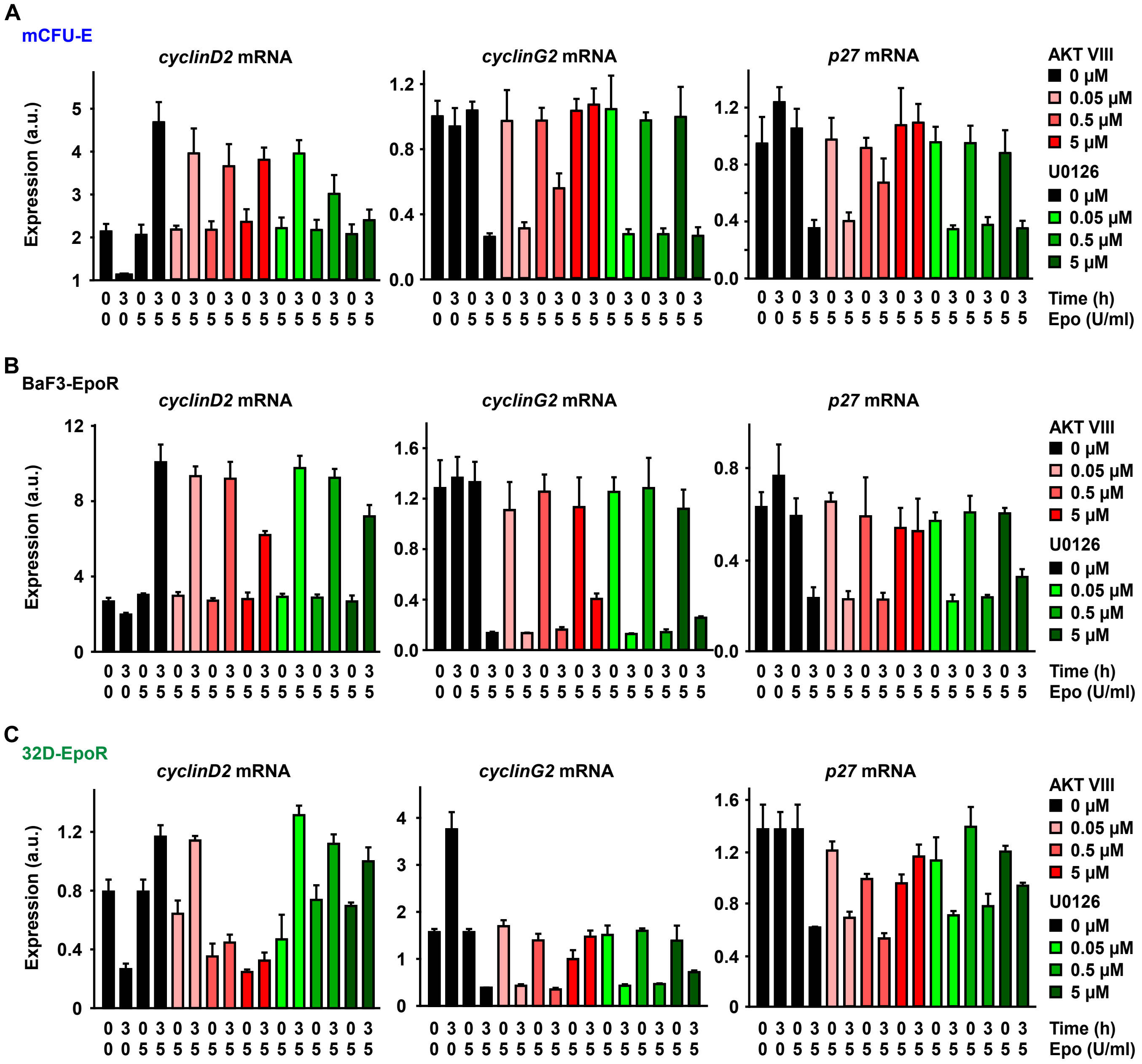

Figure S16: Expression of individual cell-cycle genes in mCFU-E, BaF3-EpoR and 32D-EpoR cells in response to Epo stimulation and inhibitor treatment. Growth-factor deprived cells were pretreated for 30 min with indicated inhibitor concentrations followed by stimulation with 5 U/ml Epo for indicated times. Per condition, total RNA from 3×10^6 cells was isolated. The expression of *cyclinD2*, *cyclinG2* and *p27* was measured by quantitative RT-PCR, and data was normalized to the *Rpl32* gene. Expression is depicted as mean ± standard deviation. Data of mCFU-E cells is shown in (A), BaF3-EpoR cells in (B), and 32D-EpoR cells in (C). N=3.

N. Linear regression to link the integrated pAKT response and the integrated ppERK response with the cell-cycle indicator in mCFU-E, BaF3-EpoR and 32D-EpoR cells

We assumed that the Epo-induced activation of AKT and ERK is required for the expression of the cell-cycle regulating genes *cyclinD2*, *cyclinG2* and *p27*, which we found to be induced upon Epo stimulation (**Fig. S16**). As mRNA expression was detected after 3 h of Epo stimulation (**Fig. S4**), the integrated response of pAKT and ppERK within the first hour of stimulation was considered to control expression of cell-cycle regulating genes. Since the integral was used, the inaccuracy of the scale of pAKT in 32D-EpoR cells (**Fig. 4A**) played only a minor role. The Epo-induced mRNA expression of the cell-cycle regulating genes *cyclinD2*, *cyclinG2* and *p27*, which was determined by quantitative RT-PCR, was used to calculate a value for the cell-cycle indicator:

$$\text{cell–cycle indicator} = \frac{\mathit{cyclinD2}}{\sqrt{\mathit{cyclinG2} \times \mathit{p27}}}. \tag{450}$$

The cell-cycle indicator was determined in mCFU-E and BaF3-EpoR cells 3 h upon Epo stimulation as exemplarily shown in **Fig. 1F**. The integrated pAKT and the integrated ppERK response in mCFU-E and BaF3-EpoR cells within 1 h of Epo stimulation was determined as the area under the curve. We linked the integrated pAKT and the integrated ppERK response to the cell-cycle indicator by linear regression analysis (**Fig. S17A**).

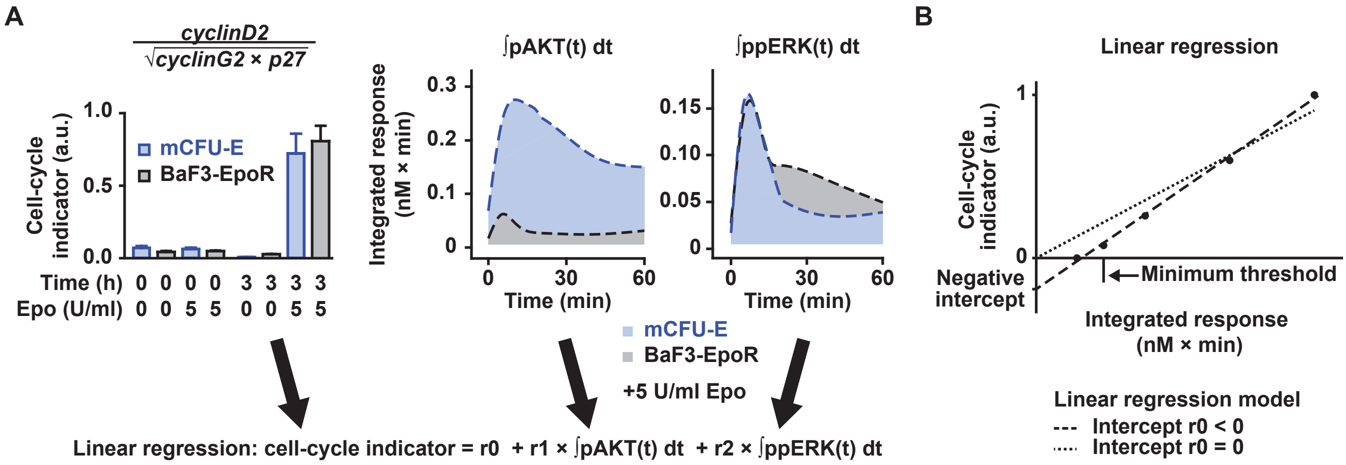

Figure S17: Linear regression to link the integrated pAKT and the integrated ppERK response with the cell-cycle indicator. (A) Left panel: The cell-cycle indicator was calculated from the expression levels of single genes *cyclinD2*, *cyclinG2*, *p27* obtained by quantitative RT-PCR shown in **Fig. S16**. Middle and right panel: Schematic representation of the area under the curve for integrated response of Epo-induced AKT and ERK activation dynamics in mCFU-E and BaF3-EpoR cells (splines from **Fig. 1E**). Linear regression was used to describe the link of integrated pAKT and integrated ppERK with the cell-cycle indicator. (B) Schematically, a minimum threshold of integrated pAKT or integrated ppERK is required to achieve a value for the cell-cycle indicator > 0. Linear regression of those values results in an intercept $r0 < 0$, which implies negative concentrations for the cell-cycle indicator. Therefore the regression model was forced through the origin with $r0 = 0$.

While the integrated pAKT response, the integrated ppERK response and the cell-cycle indicator are increased upon Epo stimulation, treatment with AKT inhibitor AKT VIII or MEK inhibitor U0126 reduces them. To investigate this link, we determined the integrated pAKT response, the integrated ppERK response (see **Fig. 5B**) and the cell-cycle indicator (see **Fig. 6B**, **Fig. S16**) in mCFU-E, BaF3-EpoR and 32D-EpoR cells for 60 minutes treatment with 5 U/ml Epo and in the presence of either AKT VIII or U0126. The maximum of the cell-cycle indicator was scaled to 1 (**Tab. S3**).
Upon exposure to increasing inhibitor doses, integrals of pAKT and ppERK and the cell-cycle indicator were reduced. Residual Epo-induced signaling activity at high inhibitor doses is insufficient to induce target gene expression. Cells need to cross a minimum threshold of signaling activity to progress through the cell cycle. Such a behavior is depicted as linear function with negative intercept (**Fig. S17B**). However, we decided to force the regression through zero because the cell-cycle indicator represents a concentration ratio that cannot be negative. We decided against non-linear regression since the model is over-parameterized in that case. Our linear regression model is given in the following:

$$\text{cell–cycle indicator} = r1 \times \int \text{pAKT}(t)\,\text{d}t \; + \; r2 \times \int \text{ppERK}(t)\,\text{d}t. \tag{451}$$

Table S3: Data used for linear regression analysis to link the integrated pAKT response and the integrated ppERK response with the cell-cycle indicator in mCFU-E, BaF3-EpoR and 32D-EpoR cells. The integrated pAKT response and the integrated ppERK response were simulated with the mathematical model for stimulation of mCFU-E, BaF3-EpoR and 32D-EpoR cells with 5 U/ml Epo for 60 minutes in the presence of AKT VIII or U0126. The cell-cycle indicator (CCI) was calculated from experimental data for mCFU-E, BaF3-EpoR and 32D-EpoR cells under the same conditions, shown in **Fig. 6B**.

		simulated data						experimental data		
		BaF3-EpoR		mCFU-E		32D-EpoR		BaF3-EpoR	mCFU-E	32D-EpoR
AKT VIII (μM)	U0126 (μM)	Int. pAKT (μM×min)	Int. ppERK (μM×min)	Int. pAKT (μM×min)	Int. ppERK (μM×min)	Int. pAKT (μM×min)	Int. ppERK (μM×min)	CCI (a.u.)	CCI (a.u.)	CCI (a.u.)
0	0	0.61	3.14	9.23	3.24	1.29	5.05	1.00	1.00	1.00
0.05	0	0.56	3.14	8.34	3.24	1.18	5.05	0.94	0.68	0.88
0.5	0	0.32	3.14	4.48	3.24	0.65	5.05	0.84	0.36	0.43
5	0	0.06	3.14	0.80	3.24	0.12	5.05	0.24	0.21	0.10
0	0	0.61	3.14	9.23	3.24	1.29	5.05	1.00	1.00	1.00
0	0.05	0.61	2.86	9.23	2.71	1.29	4.98	1.03	0.77	1.00
0	0.5	0.61	1.67	9.23	1.20	1.29	4.45	0.89	0.51	0.78
0	5	0.61	0.36	9.23	0.20	1.29	2.38	0.44	0.38	0.51

Linear regression analysis was performed with *R* and the two coefficients $r1$ and $r2$ were determined individually for mCFU-E, BaF3-EpoR and 32D-EpoR cells and implemented in our mathematical model to simulate the cell-cycle indicator under the experimentally tested conditions. Model simulations were in agreement with experimental data for physiologically more relevant intermediate inhibitor doses. The highest inhibitor dose was not well captured (see **Fig. 6B**). However, the overall high correlation between the cell-cycle indicator values simulated by the mathematical model and the experimental data (**Fig. S18**) confirmed that the minimal linear regression model (Eq. 451) is sufficient to describe the link between the integrated pAKT response and the integrated ppERK response with the cell-cycle indicator for Epo stimulation and single inhibitor treatment of mCFU-E, BaF3-EpoR and 32D-EpoR cells.

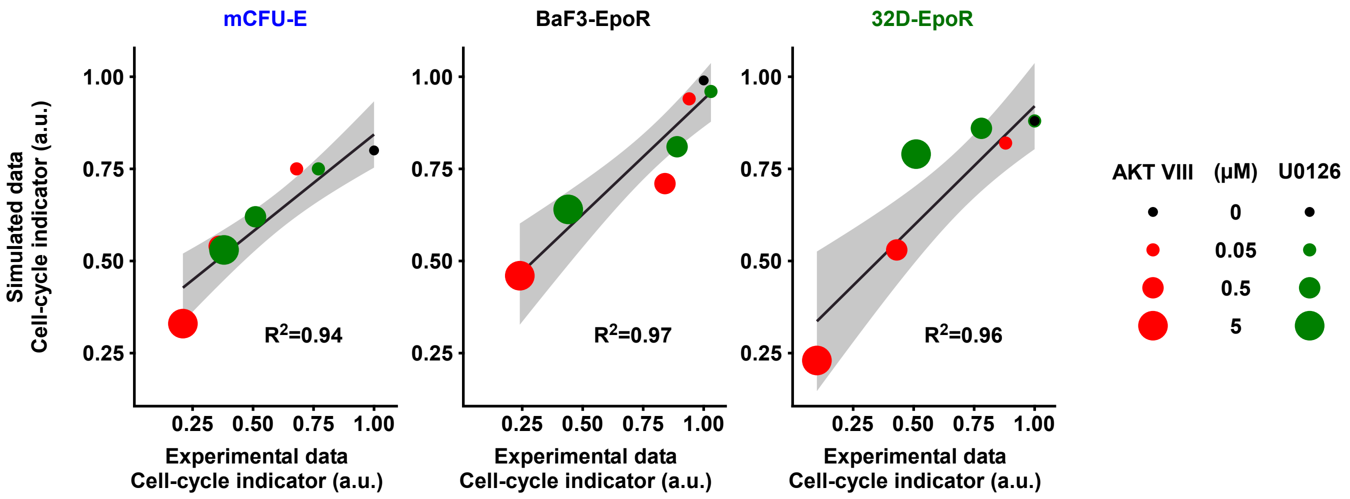

Figure S18: Correlation between experimentally determined and simulated cell-cycle indicator for mCFU-E, BaF3-EpoR and 32D-EpoR cells stimulated with 5 U/ml Epo in the presence of either AKT VIII or U0126. Expression of cell-cycle genes was measured by quantitative RT-PCR after 3 h to calculate the cell-cycle indicator. The link of the integrated pAKT response and the integrated ppERK response with the cell-cycle indicator determined by the linear regression analysis (see Eq. 451) was implemented in the mathematical model and used to simulate the cell-cycle indicator under the experimental conditions. Shaded regions indicate 95 % confidence interval. Linear regression analysis was performed with *R*.

O. Multiple linear regression to link the integrated pS6 response and/or the cell-cycle indicator with proliferation in mCFU-E, BaF3-EpoR and 32D-EpoR cells

To test how the Epo-induced integrated pS6 response and the Epo-induced cell-cycle indicator are influenced by the inhibitor treatment, we used the mathematical model to simulate both variables in mCFU-E, BaF3-EpoR and 32D-EpoR cells upon stimulation with 5 U/ml Epo in the presence of various doses of either AKT VIII or U0126. We plotted these simulated values of both variables against each other and found that they were not correlated (**Fig. 6C**). This shows that the integrated pS6 response and the cell-cycle indicator represent independent processes.

Epo-induced proliferation in the presence of inhibitors could be determined by either the integrated pS6 response, or the cell-cycle indicator, or a combination of both. To determine the impact of inhibitors on Epo-induced proliferation, we linked the integrated pS6 response and/or the cell-cycle indicator with proliferation in mCFU-E, BaF3-EpoR and 32D-EpoR cells by multiple linear regression analysis. We used the mathematical model to simulate the integrated pS6 response and the cell-cycle indicator in mCFU-E, BaF3-EpoR and 32D-EpoR cells upon stimulation with 5 U/ml Epo in the presence of various doses of either AKT VIII or U0126. These values were linked to experimental proliferation data determined by the Coulter counter assay under the same conditions. The maximum of the integrated pS6 and the proliferation data was set to 1 (**Tab. S4**).

Table S4: Data used for multiple linear regression to link the integrated pS6 response and/or the cell-cycle indicator with proliferation in mCFU-E, BaF3-EpoR and 32D-EpoR cells. The integrated pS6 response and the cell-cycle indicator (CCI) were simulated with the mathematical model for stimulation with 5 U/ml Epo for 60 minutes under given presence of inhibitors AKT VIII or U0126. Proliferation data was determined by Coulter counter assay.

		simulated data						experimental data		
		BaF3-EpoR		mCFU-E		32D-EpoR		BaF3-EpoR	mCFU-E	32D-EpoR
AKT VIII (μM)	U0126 (μM)	Int. pS6 (a. u.)	CCI (a. u.)	Int. pS6 (a. u.)	CCI (a. u.)	Int. pS6 (a. u.)	CCI (a. u.)	Proliferation (a. u.)	Proliferation (a. u.)	Proliferation (a. u.)
0	0	1.00	1.00	1.00	1.00	1.00	1.00	1.00	0.97	0.84
0.005	0	1.00	0.99	0.99	0.99	1.00	0.99	0.95	1.00	0.85
0.05	0	1.00	0.95	0.93	0.94	1.00	0.93	0.91	0.94	0.80
0.5	0	1.00	0.72	0.60	0.67	0.98	0.60	0.86	0.92	0.77
5	0	0.99	0.46	0.28	0.41	0.97	0.27	0.69	0.79	0.35
0	0	1.00	1.00	1.00	1.00	1.00	1.00	1.00	0.97	0.84
0	0.005	0.99	1.00	1.00	0.99	1.00	1.00	0.95	0.97	0.90
0	0.05	0.92	0.96	0.99	0.94	0.99	1.00	0.86	0.98	0.81
0	0.5	0.58	0.81	0.95	0.77	0.90	0.98	0.86	0.99	0.96
0	5	0.17	0.64	0.92	0.66	0.54	0.90	0.77	0.95	0.74

Multiple linear regression was considered since the system was over-parameterized when we tested non-linear models. The three tested linear regression models are listed below:

- **integrated pS6 only**: proliferation = $r3 + r4 \times \int \mathrm{pS6}(t)\,\mathrm{d}t$
- **cell-cycle indicator only**: proliferation = $r5 + r6 \times \text{cell–cycle indicator}$
- **integrated pS6 & cell-cycle indicator**: proliferation = $r7 + r8 \times \int \mathrm{pS6}(t)\,\mathrm{d}t \; + r9 \times \text{cell–cycle indicator}$

Multiple linear regression analysis was performed with *R* and the coefficients were determined to describe the Epo-induced proliferation data of mCFU-E, BaF3-EpoR and 32D-EpoR cells in the presence of single inhibitors. As the three tested linear regression models contain different numbers of coefficients, model selection was performed based on Akaike's information criterion (AIC; Burnham and Anderson 2002) to see, which model is most informative. The higher the absolute value of the calculated AIC, the more informative is the respective model to describe Epo-induced proliferation in the presence of single inhibitors (**Tab. S5**). Values in bold were chosen as the best, even if they were not significantly better than the second best.

Table S5: Multiple linear regression analysis and model selection to link the integrated pS6 response and/or the cell-cycle indicator with the impact of the single inhibitors on proliferation in mCFU-E, BaF3-EpoR and 32D-EpoR cells. Akaike's information criterion (AIC) is shown for values given in **Tab. S4**. Regression model with the highest absolute of the AIC in bold was selected as best for each cell type.

**Regression model**	**mCFU-E**	**BaF3-EpoR**	**32D-EpoR**
integrated pS6 only	**45.1**	15.0	2.7
cell-cycle indicator only	34.1	**32.4**	**18.8**
integrated pS6 and cell-cycle indicator	43.2	30.9	17.3

By multiple linear regression analysis and model selection we found that for mCFU-E cells the impact of the single inhibitors on Epo-induced proliferation is best described by the integrated pS6 response whereas for BaF3-EpoR and 32D-EpoR cells the best model considered only the cell-cycle indicator.

P. Dose-responses of Epo-induced proliferation in mCFU-E, BaF3-EpoR and 32D-EpoR cells

To further validate the determined link between Epo-induced signaling and Epo-induced proliferation, we predicted and experimentally validated numbers of mCFU-E, BaF3-EpoR and 32D-EpoR cells in response to a broad range of Epo dose

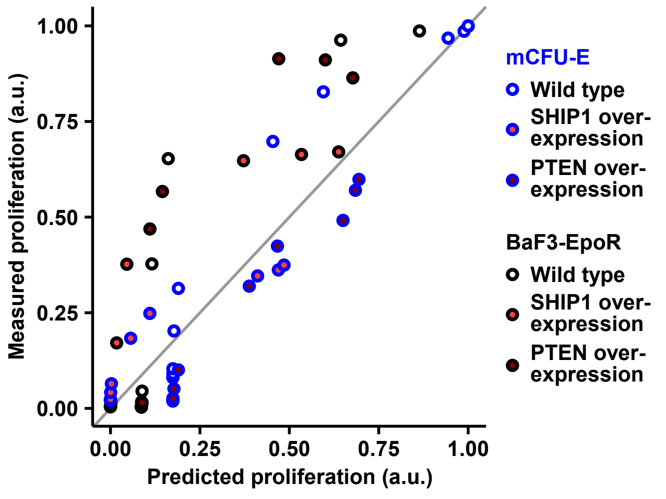

Figure S19: Correlation between the prediction and the experimental validation of Epo-induced proliferation of wild-type and SHIP1- or PTEN-overexpression mCFU-E and BaF3-EpoR cells. Proliferation was experimentally assessed using [^{3}H]-Thymidine incorporation 14 h (mCFU-E) or 38 h (BaF3-EpoR) after retroviral transduction with SHIP1 or PTEN construct for overexpression. Data as represented in **Fig. 7B**. Diagonal line as guide to the eye. Pearson's coefficient of correlation $R^2 = 0.88$.

by the Coulter counter assay. The mathematical model predicted a proliferative response of mCFU-E cells at very low Epo doses and similar dose-dependency of BaF3-EpoR and 32D-EpoR cells at higher Epo doses (**Fig. S20A**). We measured the proliferative response to different Epo doses by the Coulter counter assay after 14 h for mCFU-E cells and after 38 h for BaF3-EpoR and 32D-EpoR cells and observed, as predicted by the mathematical model, a proliferative response of mCFU-E cells at lower Epo doses than for BaF3-EpoR and 32D-EpoR cells, which were comparable in their dose-dependency (**Fig. S20B**).

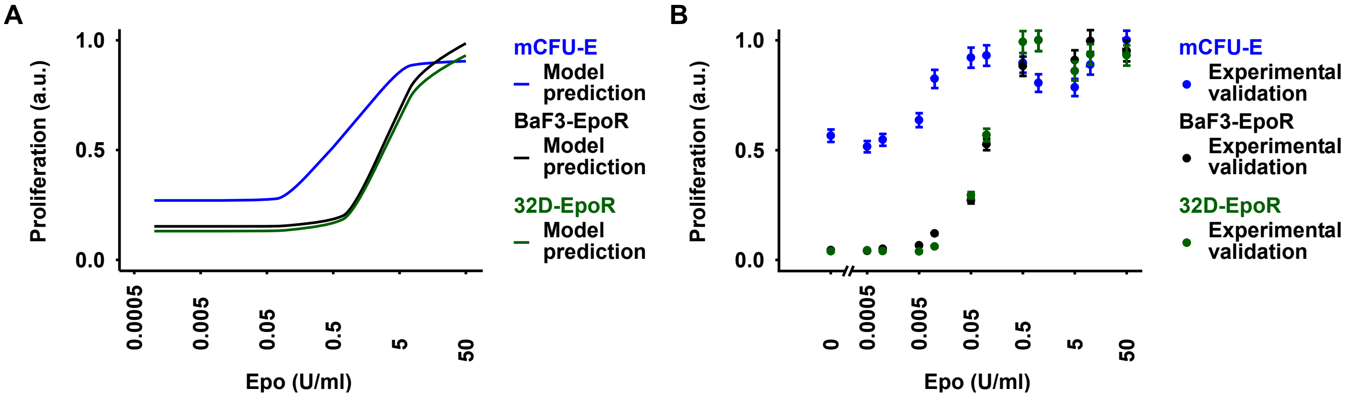

Figure S20: Proliferation of mCFU-E, BaF3-EpoR and 32D-EpoR cells in response to a broad range of Epo concentration. (A) Model prediction. (B) Experimental validation by Coulter counter assay. Proliferation of growth-factor deprived cells was assessed after 14 h for mCFU-E cells or after 38 h for BaF3-EpoR and 32D-EpoR cells. Error bars represent standard deviation from three biological replicates.

The mathematical model predicted a proliferative response of mCFU-E cells even for very low Epo doses due to residual signaling activity (**Fig. S20A**). Proliferation of mCFU-E cells even in the absence of Epo was also observed experimentally (**Fig. S20B**) because mCFU-E cells have been exposed to Epo in the fetal liver before they were prepared and growth-factor deprived. The differences in the absolute Epo-dose dependency between model simulation and experimental data (**Fig. S20**) can be explained by the fact that the model was never trained to proliferation data at Epo doses other than 5 U/ml. Basal and saturating signaling events may change the linear connection from signaling to proliferation. Thus only the relative change in Epo-dose dependency of proliferation should be considered here. The tendencies were correctly predicted: mCFU-E cells showed a proliferative response at lower Epo doses compared to BaF3-EpoR and 32D-EpoR cells, which showed a similar dose-dependency of their Epo-induced proliferative response. The different Epo-dose dependencies of the experimental data shown in **Fig. S20B** compared to the experimental data shown in **Fig. 1A** are originating from the different experimental techniques. While numbers of cells were determined by the Coulter counter assay in **Fig. S20B**, DNA replication was determined by [^{3}H]-Thymidine incorporation in **Fig. 1A**, and both processes are occurring on different time scales.

Q. Doubling times of mCFU-E, BaF3-EpoR and 32D-EpoR cells

To explain the different doubling times of mCFU-E and BaF3-EpoR cells (see **Fig. 1B**), we revisited the idea of cells first integrating growth-factor signals and then synthesizing DNA prior to division (Jones and Kazlauskas 2000). Cells grow during the G1 phase of the cell cycle and the integrated pS6 response controls protein synthesis and thus cell growth. The

variability of cell-cycle length arises from the variability in G1 phase (Prescott 1968). Subsequent progression through the cell cycle is rather constant in length (**Fig. S21A**). The timing of the G1-S progression is tightly regulated, e.g. by *cyclinD2*, *cyclinG2* and *p27* (Fang et al. 2007), and was utilized for the calculation of the cell-cycle indicator (see Eq. 450). Cell size could be the critical step determining the length of the G1 phase.
The cytoplasmic volume (see **Tab. 1**) of BaF3-EpoR cells (1,382.2 µm^3) is almost twice as large as the cytoplasmic volume of mCFU-E cells (692.5 µm^3). Assuming a linear relation between cell volume and time to grow, the length of G1 phase, T_G, should be roughly twice as long for BaF3-EpoR cells compared to mCFU-E cells, given their size. The duration of subsequent cell cycle progression, T_S, should be invariant in length between the cell types. The doubling time, $T_D = T_G + T_S$, was obtained experimentally to be 13.1 h for mCFU-E cells and 18.7 h for BaF3-EpoR cells (see **Fig. 1B**). Solving the following equation system:

$$\begin{aligned} \text{CFU–E}: \quad 13.1h &= T_G + T_S \\ \text{BaF3–EpoR}: \quad 18.7h &= 2T_G + T_S \end{aligned}$$

yields $T_G = 5.6h$, $T_S = 7.5h$.
32D-EpoR cells exhibit a cytoplasmic volume of 1,406.2 µm^3 (see **Tab. 1**), which is very similar to BaF3-EpoR cells (1,382.2 µm^3). If our assumption of a linear relation between cell volume and time to grow were true, 32D-EpoR cells should show proliferation dynamics similar to BaF3-EpoR cells. We measured cell numbers of 32D-EpoR and BaF3-EpoR cells time-resolved in response to 50 U/ml Epo by trypan blue exclusion assay and found no major differences between proliferation dynamics of BaF3-EpoR and 32D-EpoR cells (**Fig. S21B**). This observation supported our assumption that growth times can be inferred from cell volumes.

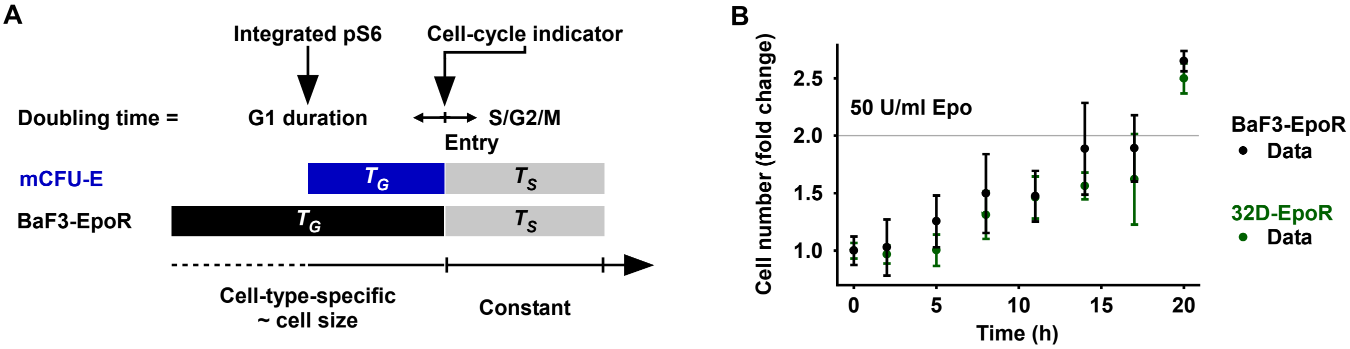

Figure S21: Proliferation as a two-step process controlling proliferation dynamics of mCFU-E, BaF3-EpoR and 32D-EpoR cells. (A) Scheme of doubling time depending on the duration of the G1 phase and progression through the cell cycle upon entry into the S/G2/M phase. While the length of the G1 phase depends on cell size and thus growth, which is controlled by the integrated pS6 response, time of subsequent cell-cycle progression is invariant, but the timing of entry into the S/G2/M phase is tightly regulated by the expression of cell-cycle regulating genes, e.g. *cyclinD2*, *cyclinG2*, *p27*, which are summarized in the cell-cycle indicator. (B) BaF3-EpoR and 32D-EpoR cells were deprived from growth factors for 1 h and stimulated with 50 U/ml Epo and counted manually by trypan blue exclusion assay using a hemocytometer. Error bars represent standard deviation of four biological replicates.

R. Cell-type-specific links of signaling to Epo-induced proliferation in the presence of AKT an/or MEK inhibitors

Upon Epo stimulation and in presence of either AKT VIII or U0126, 32D-EpoR cells exhibit similarities to BaF3-EpoR cells in their signaling response (see **Fig. 5B**) but similarities to mCFU-E cells in their proliferative response (see **Fig. 6C**) to increasing inhibitor doses.

R.1. Analysis of cell-type-specific differences in inhibitor sensitivity of the Epo-induced signaling responses

To explain the cell-type-specific differences in inhibitor sensitivity of the Epo-induced signaling responses, we analyzed the inhibitor parameters estimated by the mathematical model. The profile likelihoods calculated by the mathematical model showed no significant differences for the sensitivity towards the AKT inhibitor between mCFU-E, BaF3-EpoR and 32D-EpoR cells (**Fig. S22A**). However, the mathematical model suggested that 32D-EpoR cells are less sensitive to the MEK inhibitor as the estimated inhibition parameter $mekin$ was significantly smaller for 32D-EpoR cells than for mCFU-E and BaF3-EpoR cells (**Fig. S22B**). This reduced sensitivity of 32D-EpoR cells towards the MEK inhibitor could be explained by the high abundance of MEK in 32D-EpoR cells. 32D-EpoR cells possess more MEK than BaF3-EpoR cells

(see **Tab. 1**), and the increased MEK abundance could potentially buffer the effect of U0126 because MEK is the substrate of the inhibitor.

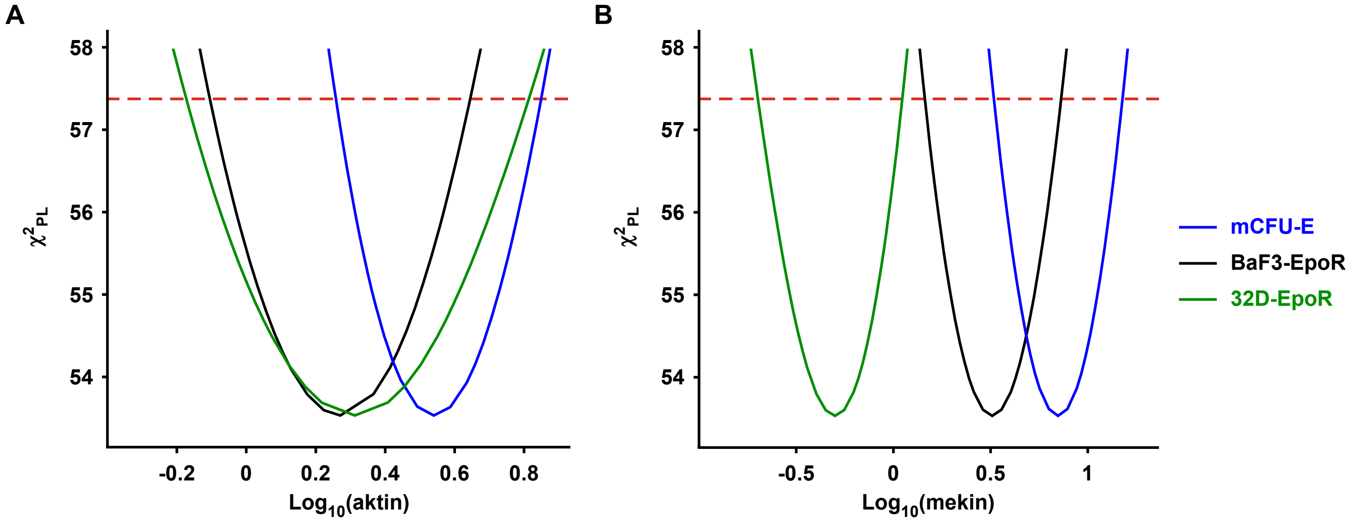

Figure S22: Profile likelihoods for AKT and MEK inhibitor parameters in mCFU-E, BaF3-EpoR and 32D-EpoR cells. χ^2 of the profile likelihood (PL) is given for the parameter for the AKT VIII sensitivity $kaktin$ in (A) and the parameter for the U0126 sensitivity $kmekin$ in (B). The solid line indicates the profile likelihood. The dashed red line indicates the threshold to assess point-wise 95 % confidence intervals.

R.2. Analysis of integrated responses of pAKT and ppERK and pS6 in mCFU-E, BaF3-EpoR and 32D-EpoR cells depending on protein abundance of pathway components

To further understand the cell-type-specific wiring of signal transduction to proliferation, we analyzed the integrated responses of pAKT and ppERK and pS6 in mCFU-E, BaF3-EpoR and 32D-EpoR cells. We computed the relative sensitivities (response coefficients) of the integrated pS6 response as shown in Fig. 4B and the relative sensitivities of the sum of integrated response of pAKT and ppERK. These coefficients calculated with the mathematical model indicate how much the integrated signaling responses depend on protein abundance of signaling pathway components. The higher the absolute value of the sensitivity, the more sensitive is the integrated response to changes in the abundance of the respective protein. We found that the key determinants for both, the integrated pS6 response and the sum of the integrated response of pAKT and ppERK, were in mCFU-E cells the PI3K/AKT pathway components, whereas in BaF3-EpoR and 32D-EpoR cells, the MAPK signaling axis was dominating both responses (**Fig. S23**).

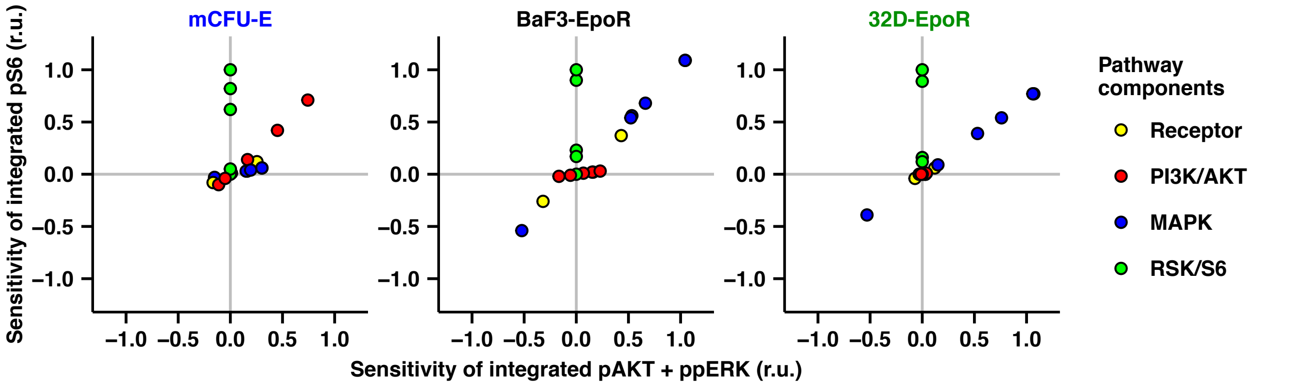

Figure S23: Dependency of the sum of the integrated response of pAKT and ppERK on the abundance of pathway components versus dependency of the integrated pS6 response on the abundance of pathway components. Relative sensitivities (response coefficients) were calculated with the mathematical model for mCFU-E, BaF3-EpoR and 32D-EpoR cells. The higher the absolute value of the calculated sensitivity, the more sensitive is the integrated response to changes in the abundance of the respective pathway component. Key determinants for both, the integrated pS6 response and the sum of the integrated response of pAKT and ppERK, lie on the diagonal line in the scatter plot.

The reason for the low sensitivity of the integrated pS6 response and the sum of the integrated response of pAKT and ppERK on PI3K/AKT pathway components in BaF3-EpoR and 32D-EpoR cells as predicted by the mathematical model (**Fig. S23**) could be the low AKT activation compared to mCFU-E cells (see Fig. S15). The PI3K/AKT pathway components contribute only slightly to the sum of integrated pAKT and ppERK in BaF3-EpoR and 32D-EpoR cells because pAKT levels in these cell lines are ≈10 times lower than in the mCFU-E cells (see **Tab. S3**).
To understand how the different levels of pAKT and ppERK regulate the cell-cycle indicator in a cell-type-specific manner, we further analyzed the coefficients $r1$ and $r2$ (see Eq. 451) determined by the linear regression analysis to link the integrated pAKT and the integrated ppERK response with the cell-cycle indicator in mCFU-E, BaF3-EpoR and 32D-EpoR cells (see **Appendix N**, page 30). The linear regression analysis suggested that all coefficients were very similar except for the coefficients in BaF3-EpoR and 32D-EpoR cells linking the integrated pAKT response to the cell-cycle indicator (**Fig. S24A**). As these significantly higher coefficients for the contribution of the integrated pAKT response to the cell-cycle indicator in BaF3-EpoR and 32D-EpoR cells were coinciding with the low levels of the integrated pAKT response in these cells, we analyzed this connection further. We simulated with the mathematical model the integrated responses of pAKT and the integrated responses of ppERK for mCFU-E, BaF3-EpoR and 32D-EpoR cells upon 60 min stimulation with 5 U/ml Epo. We plotted the coefficients determined by the linear regression analysis (see **Appendix N**, page 30) against these values and found a strong negative correlation (**Fig. S24B**). This negative correlation implied that both, the integrated pAKT response and the integrated ppERK response, contribute equally to the cell-cycle indicator because the high coefficients for the contribution of the integrated pAKT response to the cell-cycle indicator in BaF3-EpoR and 32D-EpoR cells compensate for low AKT phosphorylation levels, which is schematically shown in **Fig. S24C**.

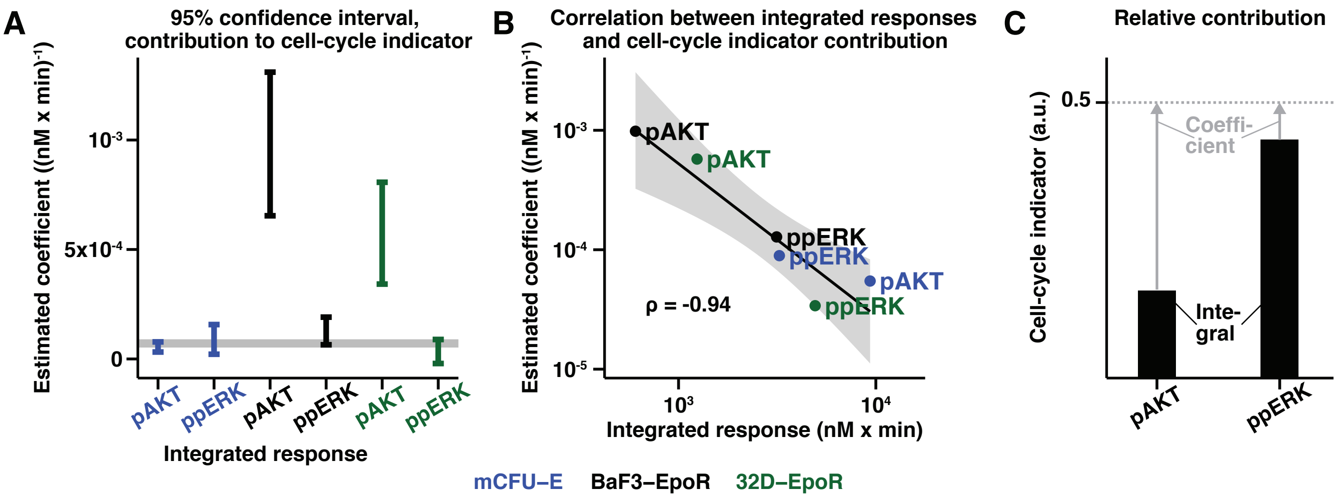

Figure S24: Relative contribution of the integrated pAKT response and the integrated ppERK response to the cell-cycle indicator in mCFU-E, BaF3-EpoR and 32D-EpoR cells determined by linear regression analysis and the mathematical model. (A) 95% confidence intervals of coefficients determined by linear regression analysis to link the integrated pAKT response and the integrated ppERK response with the cell-cycle indicator. Most of the estimated coefficients are in a similar range, which is indicated in grey as guide to the eye. (B) Estimated coefficients for the contribution of the integrated pAKT response and the integrated ppERK response to the cell-cycle indicator versus the absolute value of the respective integrated response determined by linear regression analysis and the mathematical model. Shaded region indicates 95 % confidence interval. (C) Schematic representation of the equal relative contribution of the integrated pAKT response and integrated ppERK response to the cell-cycle indicator. If the integrated pAKT response is low, as e. g. in BaF3-EpoR and 32D-EpoR cells, the coefficient for the relative contribution of the integrated pAKT response to the cell-cycle indicator determined by linear regression analysis is rather high.

R.3. Analysis of the cell-type-specific link from either the integrated pS6 response or the cell-cycle indicator to proliferation upon Epo stimulation and inhibitor treatment of mCFU-E, BaF3-EpoR and 32D-EpoR cells

We determined the link from either the integrated pS6 response and/or the cell-cycle indicator to proliferation by multiple linear regression analysis and found that in mCFU-E cells Epo-induced proliferation in the presence of single inhibitors is best described by the integrated pS6 response whereas in BaF3-EpoR and 32D-EpoR cells the cell-cycle indicator describes proliferation under these conditions best (see **Appendix O**, page 31). We have already seen that the integrated pS6 response and the cell-cycle indicator represent independent processes (see **Fig. ??**). However, we wanted to test whether their contribution to proliferation is mutually exclusive. We simulated with the mathematical model for mCFU-E, BaF3-EpoR and 32D-EpoR cells with the cell-cycle indicator linked to the Epo-induced proliferation in the presence of single inhibitors

and found a rather low correlation for mCFU-E cells but good agreement between model simulation and experimental data for BaF3-EpoR and 32D-EpoR cells (**Fig. S25A**). Conversely, the integrated pS6 response linked to the Epo-induced proliferation in the presence of single inhibitors yielded a high coefficient of determination for mCFU-E cells but rather low agreement between model simulation and experimental data for BaF3-EpoR and 32D-EpoR cells (**Fig. S25B**). These observations strengthen our findings from the model selection upon multiple linear regression that the "integrated pS6 response only" is the best model in mCFU-E cells and the "cell-cycle indicator only" is the best model in BaF3-EpoR and 32D-EpoR cells to describe Epo-induced proliferation in the presence of single inhibitors (see **Tab. S5**).

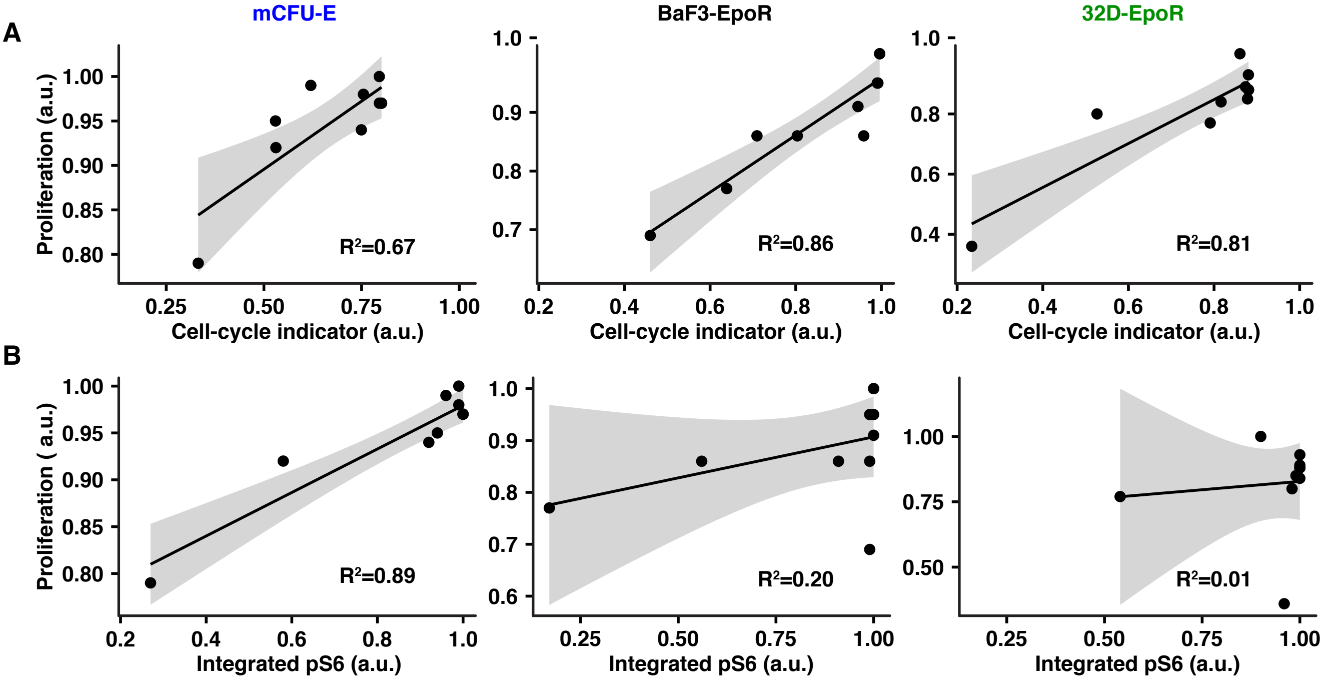

Figure S25: Correlation between experimentally measured proliferation upon Epo stimulation and single inhibitor treatment in mCFU-E, BaF3-EpoR and 32D-EpoR cells and the cell-cycle indicator or the integrated pS6 response. Cells were stimulated with 5 U/ml Epo and treated with different doses of either AKT VIII or U0126, proliferation was measured by the Coulter counter assay. Shaded regions indicate 95 % confidence interval. Linear regression analysis was performed with *R*. (A) Correlation between proliferation and the cell-cycle indicator. (B) Correlation between proliferation and the integrated pS6 response.

As we finally (**Fig. 7B**, **Fig. 8F**) predict whether the co-treatment of AKT VIII and U0126 has more effect on Epo-induced proliferation than the single inhibitors, we systematically tested all linear regression models from **Tab. S5** for the link of either the integrated pS6 response and/or the cell-cycle indicator with proliferation upon Epo stimulation and inhibitor treatment. If both, the integrated pS6 response and the cell-cycle indicator contribute to proliferation, the mathematical model suggested a combined inhibitor effect on Epo-induced proliferation in BaF3-EpoR cells and to a lower extent also in 32D-EpoR cells but not in mCFU-E cells (**Fig. S26A**). With only the cell-cycle indicator linked to proliferation, the mathematical model predicted a combined inhibitor effect on Epo-induced proliferation in mCFU-E and BaF3-EpoR cells and to a lower extent also in 32D-EpoR cells (**Fig. S26B**). With only the integrated pS6 response linked to proliferation, an effect of the AKT inhibitor on Epo-induced proliferation in mCFU-E cells and no inhibition in BaF3-EpoR and 32D-EpoR cells was suggested by the mathematical model (**Fig. S26C**). In experimental validation experiments it was observed that the co-treatment of AKT VIII and U0126 exerted a combined effect on Epo-induced proliferation in BaF3-EpoR cells but not mCFU-E or 32D-EpoR cells (**Fig. S26D**).

We have shown that the control of Epo-induced proliferation in the presence of AKT an/or MEK inhibitors are cell-type-specific. The mathematical model links signaling, gene expression and proliferation based on the cell-type-specific protein abundance. In mCFU-E cells proliferation is controlled by the integrated pS6 response and its preferential activation through PI3K/AKT pathway components is caused by lower protein abundance in the MAPK signaling axis whereas in BaF3-EpoR and 32D-EpoR cells show high abundance of MEK, which in 32D-EpoR cells seems to buffer the effect of the MEK inhibitor on signaling, the cell-cycle indicator and proliferation (**Fig. S27**).

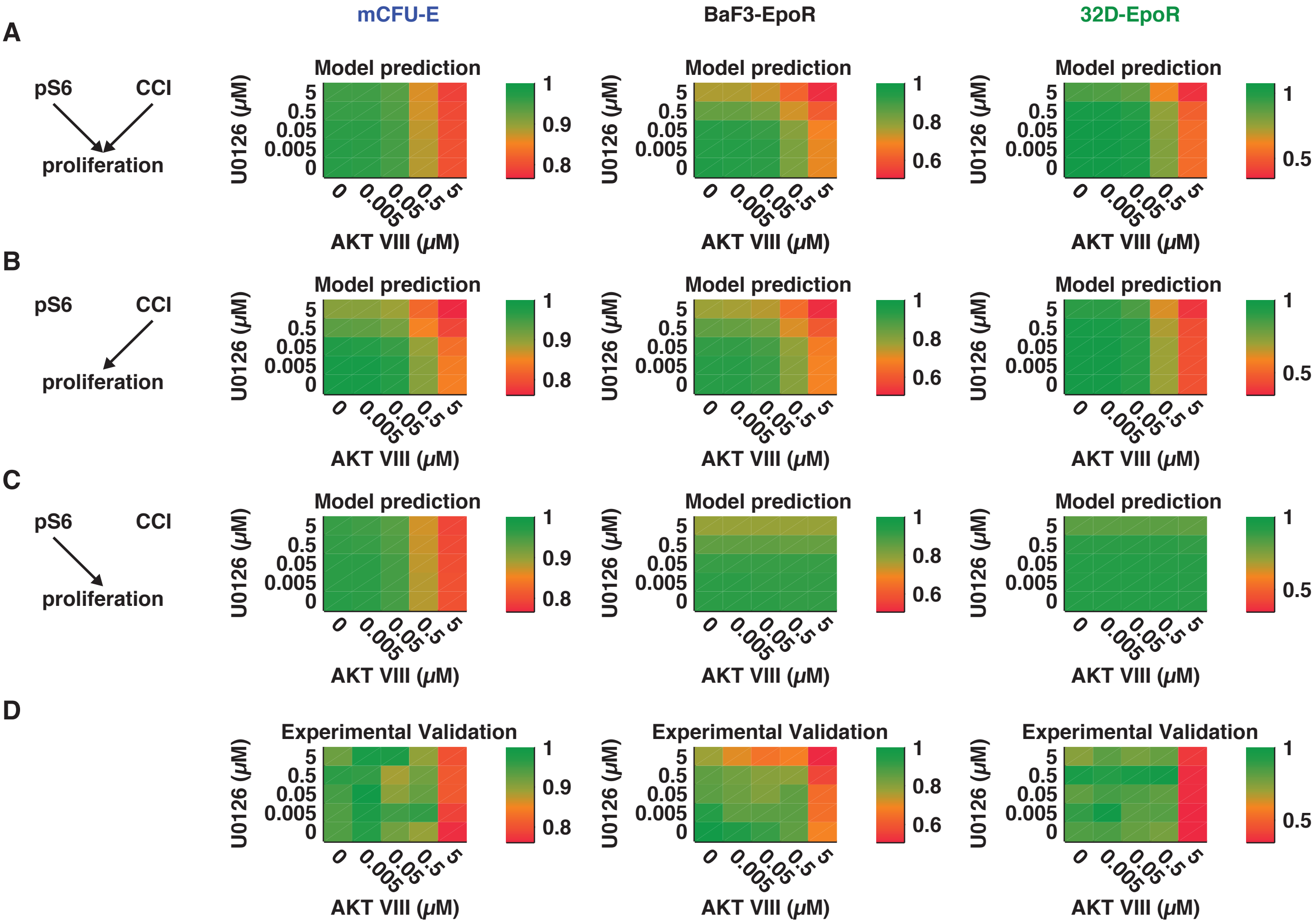

Figure S26: Predictions and experimental validations of proliferation upon Epo stimulation and co-treatment of inhibitors in mCFU-E, BaF3-EpoR and 32D-EpoR cells. For A-C, a representative model is shown on the left. (A) Model prediction for both, the integrated pS6 response and the cell-cycle indicator, contributing to proliferation. (B) Model prediction for only the cell-cycle indicator contributing to proliferation. (C) Model prediction for only the integrated pS6 response contributing to proliferation. (D) Experimental validation. Growth-factor deprived cells were stimulated with 5 U/ml Epo and treated with indicated doses of AKT VIII and U0126. Proliferation was determined by the Coulter counter assay. Only single-inhibitor treatment was used for model calibration. Multi-dimensional linear regression analysis was performed and model selection based on Akaike's information criterion (see **Tab. S5**). *pS6* integrated pS6 response, *CCI* cell-cycle indicator.

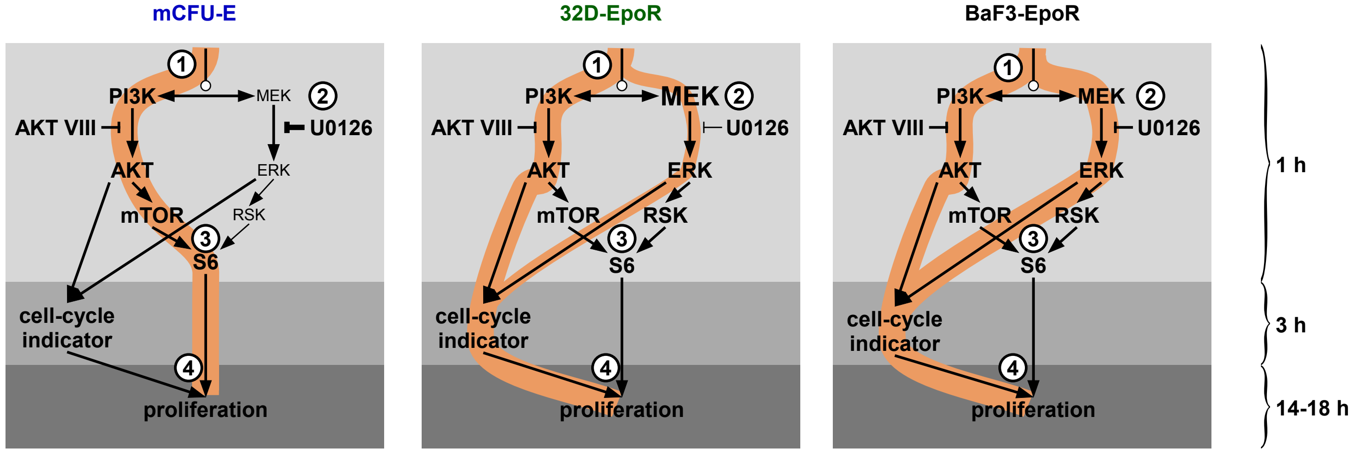

Figure S27: Proposed scheme for cell-type-specific information processing of Epo stimulation. Simplified view of key components of the model for mCFU-E, BaF3-EpoR and 32D-EpoR cells. Three distinct time scales: signal transduction within 1 hour upon stimulation, gene expression from 3 hours on, and proliferation from 14 hours on. (1) Signal input triggers activation of the AKT and the ERK pathway of the network. Protein abundance determines information flow through the components. Systematic model reduction showed for instance negligible contributions of adapters Gab1/2. (2) Preferential activation of S6 through pAKT in mCFU-E cells is caused by lower abundance of MEK whereas particularly high amounts of MEK in 32D-EpoR cells reduce the impact of U0126 compared to BaF3-EpoR cells. (3) Sensitivity analysis (see **Fig. 4B**) suggested cell-type-specific contribution of AKT and ERK to integrated S6 activation that can be explained by protein abundance of the PI3K/AKT and the MEK/ERK branch. (4) Integrated pS6 as a proxy for cell growth is the key determinant of proliferation in mCFU-E cells because cell size is critical for these primary cells. In BaF3-EpoR and 32D-EpoR cells, growth is not rate-limiting and here the cell-cycle indicator describes proliferation best.

S. Predicting the effect of inhibitor treatment on Epo-induced proliferation in hCFU-E cells based on protein abundance

We asked whether the effect of treatment with single inhibitors and co-treatment of AKT and MEK inhibitors on Epo-induced proliferation of human colony-forming unit-erythroid (hCFU-E) cells can be predicted with our integrated mathematical model based on protein abundance of signaling components.
Predictions of AKT/MEK inhibitor treatment effects on Epo-induced proliferation required three additional information:

1. **The link from either integrated pS6 (i. e. cell growth in G1) or the cell-cycle indicator (i. e. G1-S progression) to proliferation.** As hCFU-E cells are considerably larger than mCFU-E cells (see **Fig. 8B**) and double much slower (**Fig. S28**), we assumed that cell growth is not the critical step, but proliferation is rather controlled by the timing of G1-S progression during the cell cycle, which is determined by the cell-cycle indicator (see **Fig. S21A**).

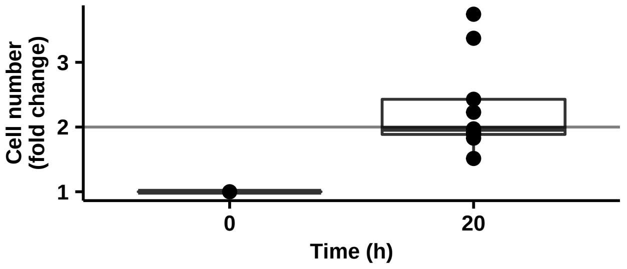

Figure S28: Proliferation of hCFU-E cells in response to 5 U/ml Epo. Obtained hCFU-E cells on differentiation day 3 were deprived from growth factors for an hour and subsequently stimulated with 5 U/ml Epo. After 20 h, cell numbers were determined by manual counting using trypan blue exclusion assay. The upper/lower whisker of the boxplots correspond to highest/lowest value within 1.5 × inter-quartile range. N=9.

The link from the cell-cycle indicator to proliferation upon Epo stimulation and single-inhibitor treatment was very similar in BaF3-EpoR and 32D-EpoR cells. We therefore assumed that the same link could also be present in hCFU-E cells. The link of the cell-cycle indicator with proliferation upon Epo stimulation and single-inhibitor treatment determined by the linear regression analysis (see **Appendix N**, page 30) was not significantly different in BaF3-EpoR and 32D-EpoR cells (**Tab. S6**). We therefore averaged the contribution and implemented these values in our integrative mathematical model.

2. **The strength of the AKT and the MEK inhibitor.** We simulated the effect of U0126 on the integrated ppERK response in BaF3-EpoR cells for a protein abundance of MEK ranging from 2,190 nM to 8,760 nM with the

Table S6: Contribution of the cell-cycle indicator to Epo-induced proliferation in the presence of single inhibitors for BaF3-EpoR and 32D-EpoR cells. Coefficients for proliferation = m × cell-cycle indicator + n obtained for BaF3-EpoR and 32D-EpoR cells (see **Tab. S5**). Upper and lower bound of confidence interval (CI) were obtained with *R*.

Cell type	m	2.5% CI	97.5% CI	n	2.5% CI	97.5% CI
**BaF3-EpoR**	0.49	0.33	0.65	0.47	0.33	0.61
**32D-EpoR**	0.61	0.44	1.02	0.26	0.04	0.49

mathematical model and found a linear dependency between MEK abundance and the strength of the MEK inhibitor. In mCFU-E cells, the strength of U0126, kmekinc, was estimated to 6.8 nM^{-1} with $[\mathrm{MEK}]_{\mathrm{mCFU-E}}$ = 1,460 nM. In BaF3-EpoR cells, the strength of U0126, kmekinb, was estimated to 2.8 nM^{-1} with $[\mathrm{MEK}]_{\mathrm{BaF3-EpoR}}$ = 4,380 nM. These values resulted in the following equation system:

$$\begin{aligned} \mathrm{mCFU\text{–}E}: \quad 6.8 &= m \times 1,460 + n \\ \mathrm{BaF3\text{–}EpoR}: \quad 2.8 &= m \times 4,380 + n \end{aligned}$$

Thus for hCFU-E cells with $[\mathrm{MEK}]_{\mathrm{hCFU-E}}$ = 892.4 nM, determined by quantitative mass spectrometry in combination with the "proteomic ruler" method (see **Fig. 8E**), the strength of U0126 in hCFU-E cells, kmekinh, can be calculated with the following equation:

$$\mathrm{hCFU\text{–}E}: \mathrm{kmekinh} = -0.0014 \times [\mathrm{MEK}]_{\mathrm{hCFU\text{–}E}} + 8.8$$

Similarly, the strength of AKT VIII, kaktinh, can be calculated:

$$\begin{aligned} \mathrm{mCFU\text{–}E}: \quad 3.5 &= m \times 407 + n \\ \mathrm{BaF3\text{–}EpoR}: \quad 1.9 &= m \times 510 + n \end{aligned}$$

Thus for hCFU-E cells with $[\mathrm{AKT}]_{\mathrm{hCFU-E}}$ = 24.4 nM, the strength of AKT VIII in hCFU-E cells, kaktinh, can be calculated with the following equation:

$$\mathrm{hCFU\text{–}E}: \mathrm{kaktinh} = -0.0155 \times [\mathrm{AKT}]_{\mathrm{hCFU\text{–}E}} + 9.8$$

These calculations yielded kmekinh = 7.55 nM^{-1} and kaktinh = 9.42 nM^{-1}. We implemented these values into our integrative mathematical model.

3. **The contribution of integrated pAKT and ppERK to the cell-cycle indicator.** We applied the concept from **Appendix R** and **Figure S24**, and assumed that integrated pAKT and integrated ppERK contributed equally to the cell-cycle indicator. The contribution of integrated pAKT in hCFU-E cells, cycleaktinh, and the contribution of integrated ppERK in hCFU-E cells, cyclemekinh, to the cell-cycle indicator, which correspond to $r1$ and $r2$ in Eq. 451, were calculated accordingly, given that the highest value of the cell-cycle indicator, maxcch, is the sum of both integrated responses in absence of inhibitor treatment for 5 U/ml Epo stimulation.

$$\begin{aligned} \mathrm{cycleaktinh} &= 0.50\ (\mathrm{nM} \times \mathrm{min})^{-1} \\ \mathrm{cyclemekinh} &= 1.47\ (\mathrm{nM} \times \mathrm{min})^{-1}, \\ \mathrm{maxcch} &= 32.1 \end{aligned}$$

With these calculations, we were able to predict based on protein abundance of signaling components the effect of AKT VIII and U0126 on Epo-induced proliferation in hCFU-E cells. The mathematical model suggested that the individual inhibitors, AKT VIII and U0126, and the combination thereof exhibited an effect on Epo-induced proliferation (**Fig. 8E**). During expansion and differentiation of $CD34^{+}$ cells, we observed differences between the three independent healthy stem

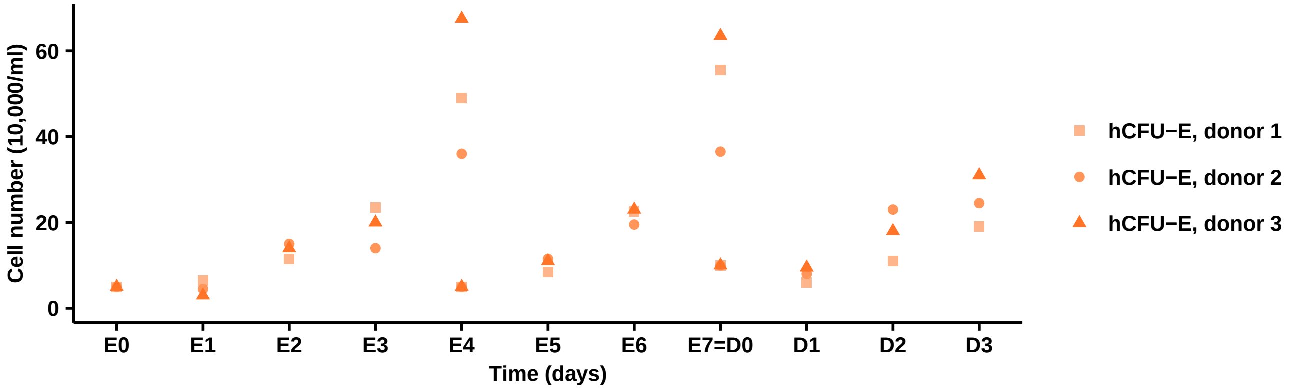

Figure S29: Expansion and differentiation of CD34$^+$ cells mobilized into the peripheral blood of three independent healthy human stem cell donors. To obtain hCFU-E cells, CD34$^+$ were expanded for 7 days and then differentiated for three days until the experiment was performed. On expansion (E) day 4 and differentiation (D) 0, cells were subcultivated.

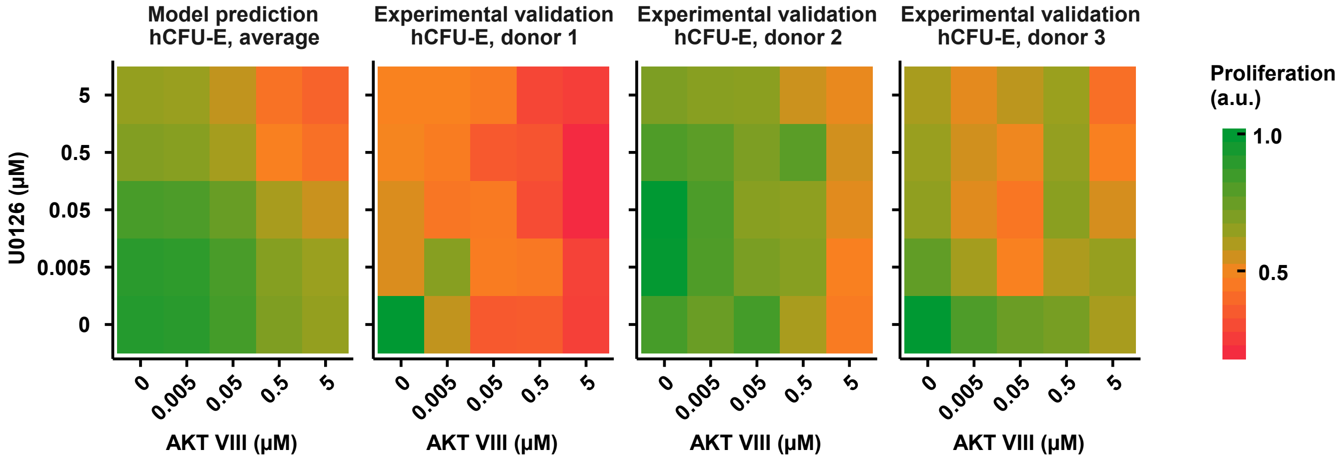

Figure S30: Prediction of proliferation upon Epo stimulation and single or co-treatment with AKT VIII and U0126 in hCFU-E cells based on protein abundance. Left panel: Model prediction of proliferation upon combined inhibitor treatment and 5 U/ml Epo stimulation. Maximum proliferation was scaled to 1. Other panels: Experimental validation of proliferation upon combined inhibitor treatment and 5 U/ml Epo stimulation of hCFU-E cells derived from three independent healthy donors. Proliferation was measured as cell numbers by trypan blue exclusion assay after 96 h. Maximum was scaled to 1. Data is represented as mean. N=3.

cell donors (**Fig. S29**). The effect of different combinations of AKT VIII and U0126 on Epo-induced proliferation in hCFU-E cells was visible to various degrees for the three donors (**Fig. S30**).
When we compared the cell numbers during cultivation of hCFU-E cells, we found that donor 3 showed a significantly higher fold change compared to donor 1 ($p = 0.038$) and donor 2 ($p = 0.009$) after three days of subcultivation (**Fig. S31**). However, the effect of the individual and combined treatment with the inhibitors, AKT VIII and U0126, on the Epo-induced proliferation of hCFU-E cells were in good agreement between donor 1 and donor 3 (**Fig. S32**) as indicated by a Pearson's coefficient of correlation $R^2 = 0.88$. In line with our observations on the proliferation during cultivation of the CD34$^+$ cells (**Fig. S31**), hCFU-E cells from donor 3 exhibited relatively high proliferation compared to donor 1 upon Epo stimulation and inhibitor treatment because almost all data points lie below the diagonal line (**Fig. S32**).

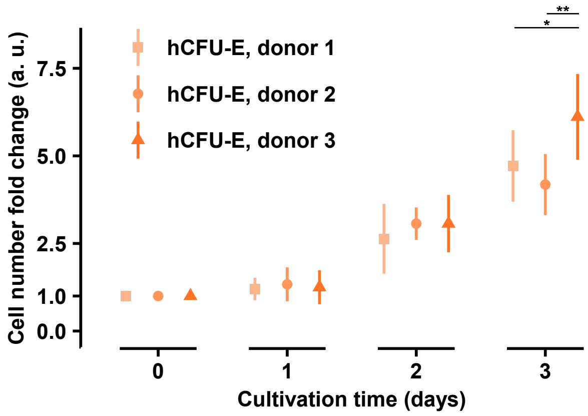

Figure S31: Comparison of proliferation of CD34$^+$ cells mobilized into the peripheral blood of three independent, healthy human stem cell donors. Cell numbers during cultivation were determined by the trypan blue blue exclusion assay. Data is represented as mean, error bars indicate standard error of the mean. Experiments were performed at least three times. Paired Student's t-test, * $p < 0.05$, ** $p < 0.01$.

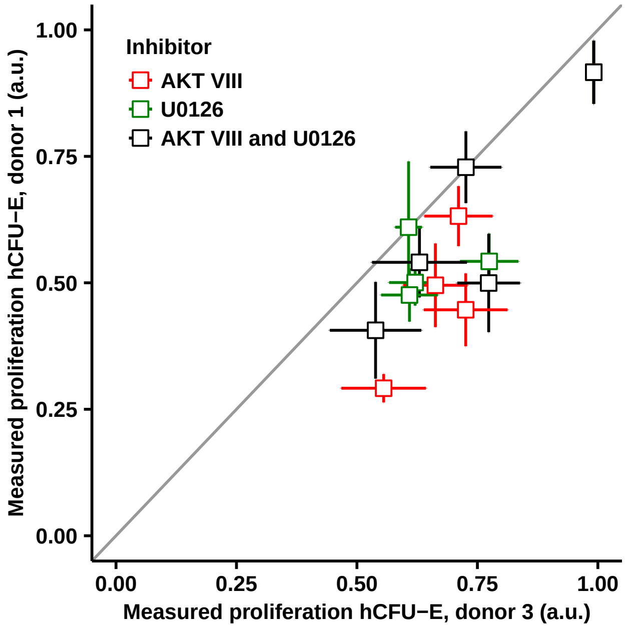

Figure S32: Comparison of proliferation upon Epo stimulation and single or co-treatment with AKT VIII and U0126 in hCFU-E cells from two independent healthy stem cell donors. hCFU-E cells were treated for 96 h with 5 U/ml Epo and 0, 0.005, 0.05, 0.5 or 5 µM of a single inhibitor or both inhibitors, AKT VIII and U0126. Data is represented as mean, error bars indicate standard error of the mean. Experiments were performed twice with each three biological replicates. Pearson's coefficient of correlation $R^2 = 0.88$. N=6.

The mathematical model suggested that in murine CFU-E cells, treatment with the AKT inhibitor only exhibited an effect on Epo-induced proliferation (**Fig. 6C, 7B**) whereas Epo-induced proliferation of human CFU-E cells is affected by the treatment with the individual inhibitors, AKT VIII and U0126, and the combination thereof (**Fig. 8F**). When we compared our experimental results for the impact of the inhibitors on Epo-induced proliferation of mCFU-E and hCFU-E cells, we found that, in line with these model predictions, the impact of AKT VIII (p=0.3) was not significantly different between mCFU-E and hCFU-E cells whereas U0126 (p=0.03) and the combinatorial inhibitor treatment (p=0.03) exhibited significantly larger effects on Epo-promoted proliferation of hCFU-E than mCFU-E cells (**Fig. 8G**).

References

Bachmann, J, Raue, A, Schilling, M, Böhm, ME, Kreutz, C, Kaschek, D, Busch, H, Gretz, N, Lehmann, WD, Timmer, J, and Klingmüller, U (2011). Division of labor by dual feedback regulators controls JAK2/STAT5 signaling over broad ligand range. *Molecular Systems Biology* 7.516, 1–15.

Burnham, KP and Anderson, DR (2002). *Model Selection and Multimodel Inference: A Practical Information-Theoretic Approach*. 2nd ed. New York: Springer, 488.

Fang, J, Menon, M, Kapelle, W, Bogacheva, O, Bogachev, O, Houde, E, Browne, S, Sathyanarayana, P, and Wojchowski, DM (2007). EPO modulation of cell-cycle regulatory genes, and cell division, in primary bone marrow erythroblasts. *Blood* 110.7, 2361–70.

Hindmarsh, AC, Brown, PN, Grant, KE, Lee, SL, Serban, R, Shumaker, DE, and Woodward, CS (2005). {SUNDIALS}: Suite of nonlinear and differential/algebraic equation solvers. *ACM T Math Software* 31.3, 363–396.

Jones, SM and Kazlauskas, A (2000). Connecting signaling and cell cycle progression in growth factor-stimulated cells. *Oncogene* 19, 5558–5567.

Prescott, DM (1968). Regulation of cell reproduction. *Cancer research* 28.9, 1815–20.
