# Supplementary figures and images for: Protein abundance of AKT and ERK pathway components governs cell type‐specific regulation of proliferation (part 2 of 3)
Source: Mol Syst Biol. 2017 Jan 25;13(1):904. doi: 10.15252/msb.20167258 (PMC5293153; doi:10.15252/msb.20167258)

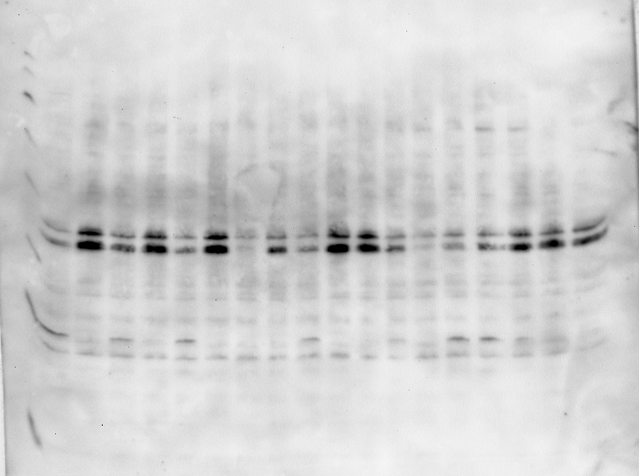

Supplement: Supplementary file 5 — Source Data for Appendix [file MSB-13-904-s013.zip › Source_Data_for_Appendix/Figure_S10/panel_B/CFUE_ppERK.tif]

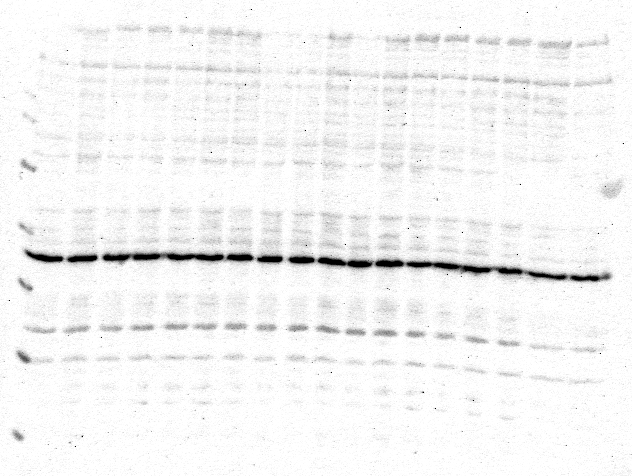

Supplement: Supplementary file 5 — Source Data for Appendix [file MSB-13-904-s013.zip › Source_Data_for_Appendix/Figure_S10/panel_B/BaF3_PDI.jpg]

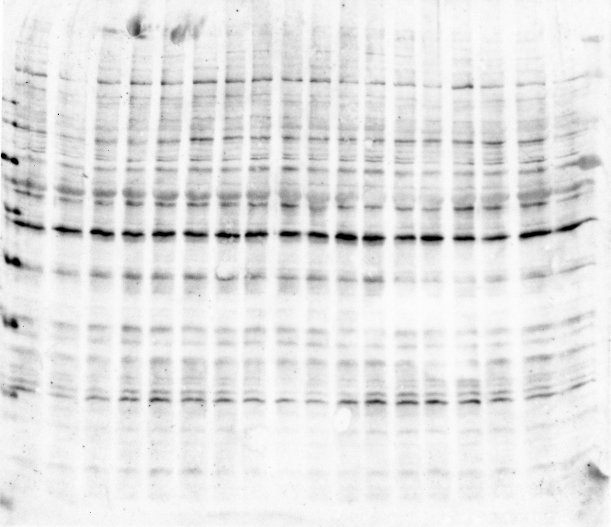

Supplement: Supplementary file 5 — Source Data for Appendix [file MSB-13-904-s013.zip › Source_Data_for_Appendix/Figure_S10/panel_B/CFUE_PDI.tif]

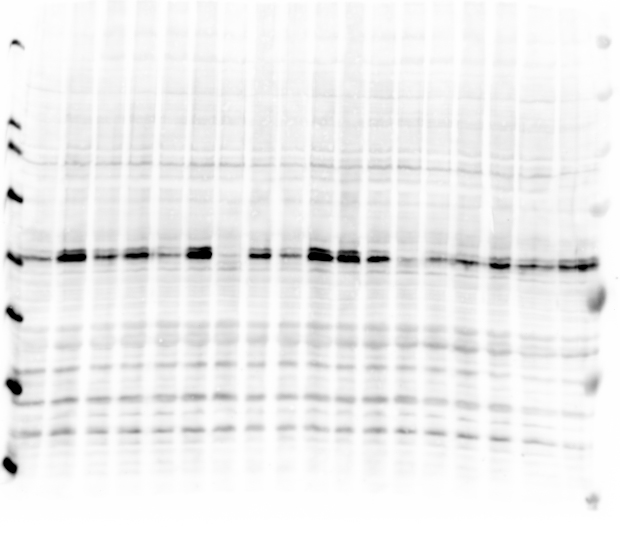

Supplement: Supplementary file 5 — Source Data for Appendix [file MSB-13-904-s013.zip › Source_Data_for_Appendix/Figure_S10/panel_B/BaF3_pAKT.jpg]

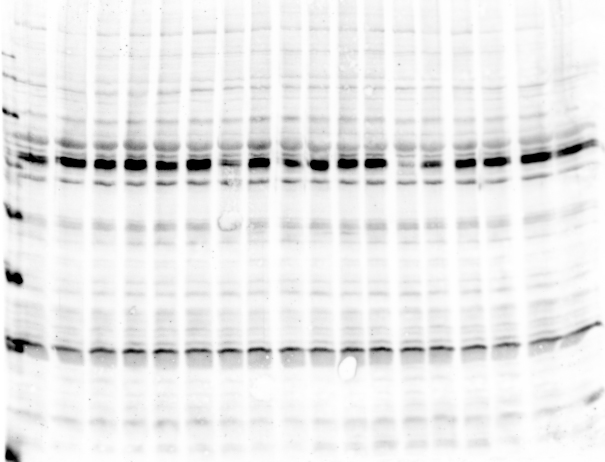

Supplement: Supplementary file 5 — Source Data for Appendix [file MSB-13-904-s013.zip › Source_Data_for_Appendix/Figure_S10/panel_B/CFUE_pAKT.jpg]

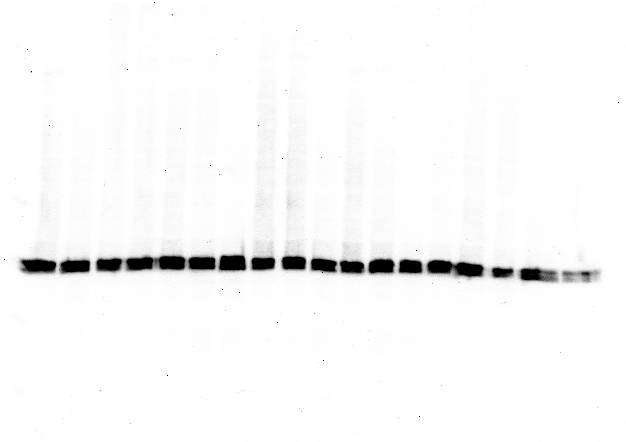

Supplement: Supplementary file 5 — Source Data for Appendix [file MSB-13-904-s013.zip › Source_Data_for_Appendix/Figure_S10/panel_B/BaF3_AKT.jpg]

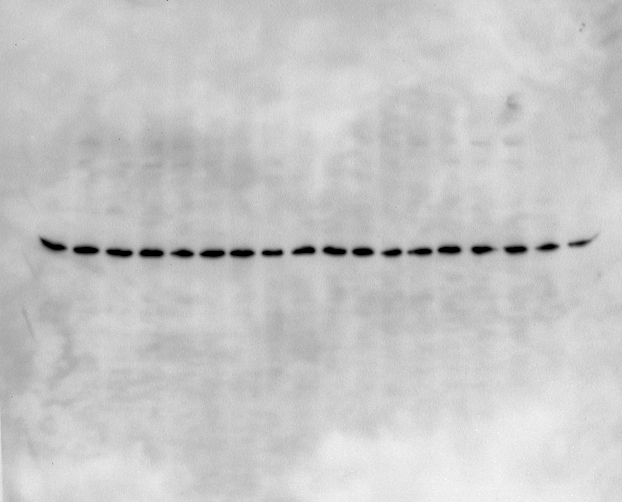

Supplement: Supplementary file 5 — Source Data for Appendix [file MSB-13-904-s013.zip › Source_Data_for_Appendix/Figure_S10/panel_B/CFUE_actin.jpg]

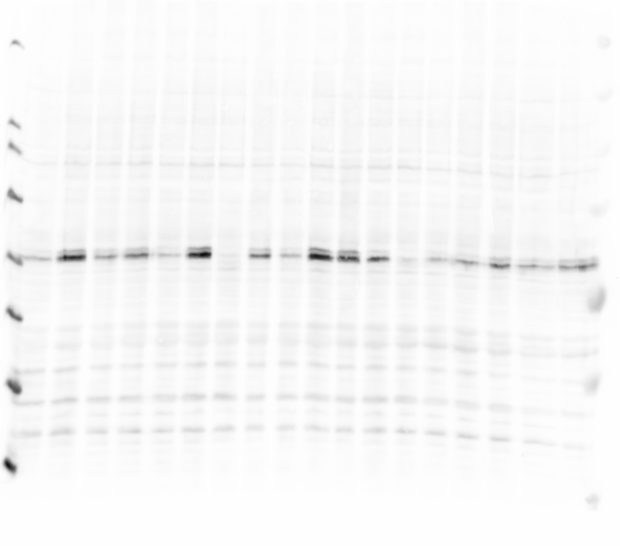

Supplement: Supplementary file 5 — Source Data for Appendix [file MSB-13-904-s013.zip › Source_Data_for_Appendix/Figure_S10/panel_B/BaF3_pAKT.tif]

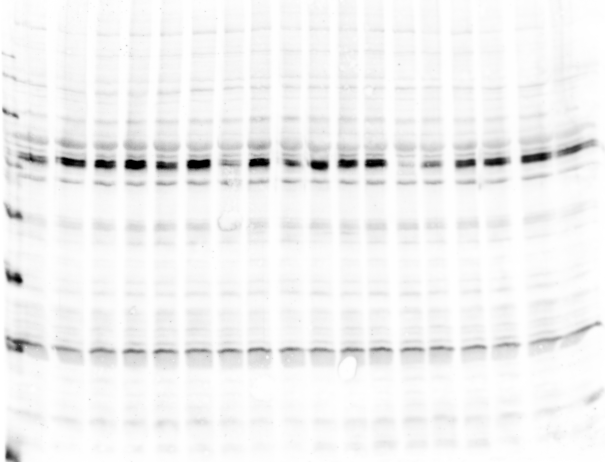

Supplement: Supplementary file 5 — Source Data for Appendix [file MSB-13-904-s013.zip › Source_Data_for_Appendix/Figure_S10/panel_B/CFUE_pAKT.tif]

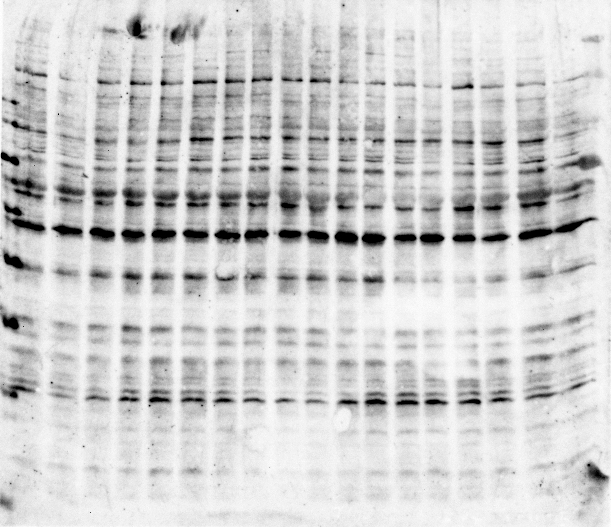

Supplement: Supplementary file 5 — Source Data for Appendix [file MSB-13-904-s013.zip › Source_Data_for_Appendix/Figure_S10/panel_B/CFUE_PDI.jpg]

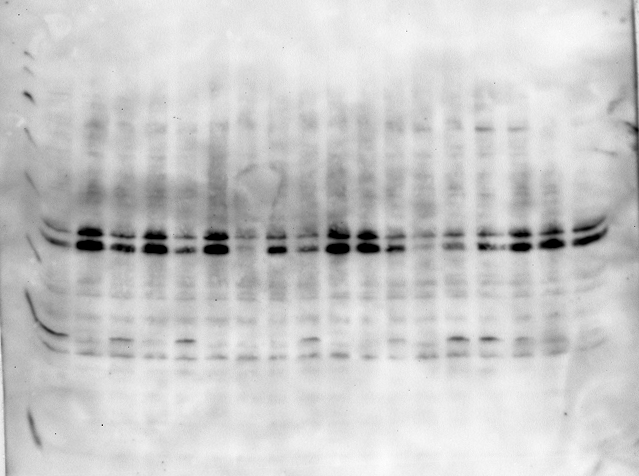

Supplement: Supplementary file 5 — Source Data for Appendix [file MSB-13-904-s013.zip › Source_Data_for_Appendix/Figure_S10/panel_B/CFUE_ppERK.jpg]

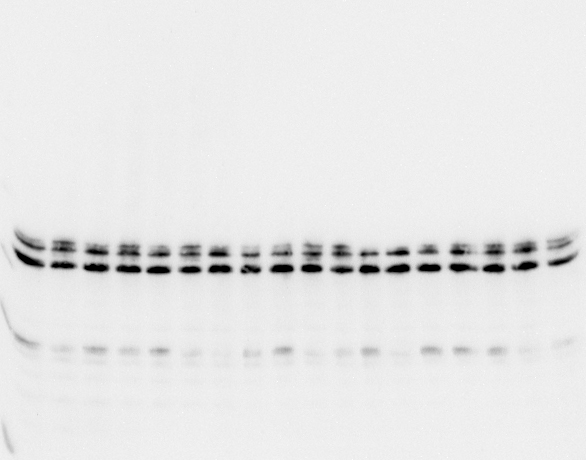

Supplement: Supplementary file 5 — Source Data for Appendix [file MSB-13-904-s013.zip › Source_Data_for_Appendix/Figure_S10/panel_B/CFUE_ERK.jpg]

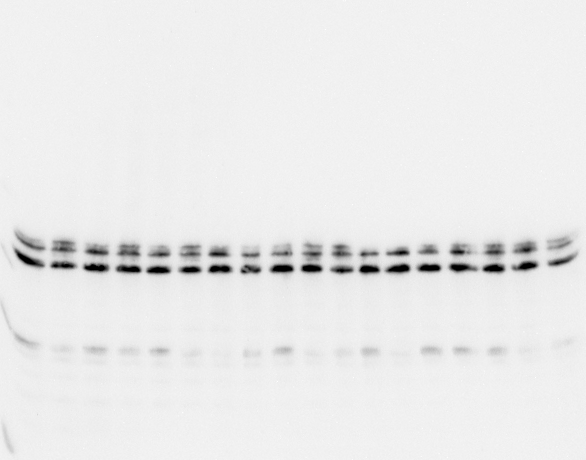

Supplement: Supplementary file 5 — Source Data for Appendix [file MSB-13-904-s013.zip › Source_Data_for_Appendix/Figure_S10/panel_B/CFUE_ERK.tif]

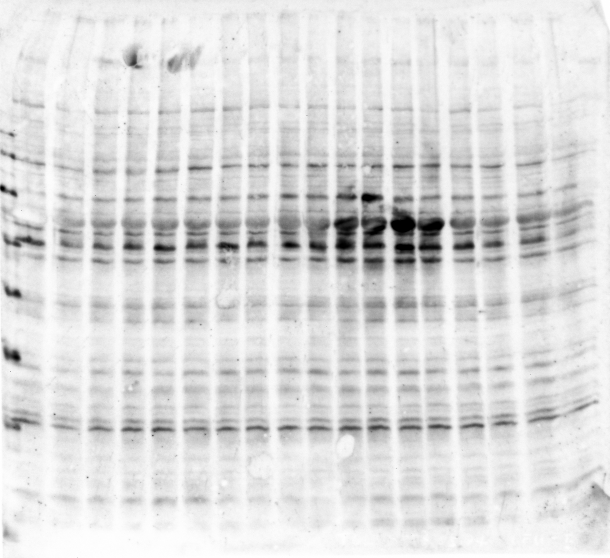

Supplement: Supplementary file 5 — Source Data for Appendix [file MSB-13-904-s013.zip › Source_Data_for_Appendix/Figure_S10/panel_B/CFUE_AKT.tif]

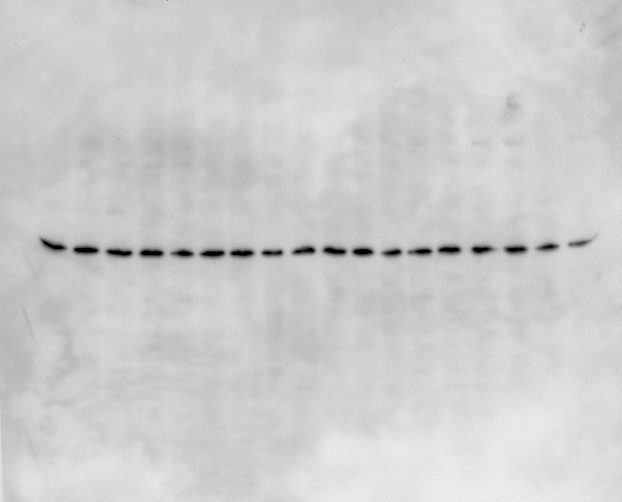

Supplement: Supplementary file 5 — Source Data for Appendix [file MSB-13-904-s013.zip › Source_Data_for_Appendix/Figure_S10/panel_B/CFUE_actin.tif]

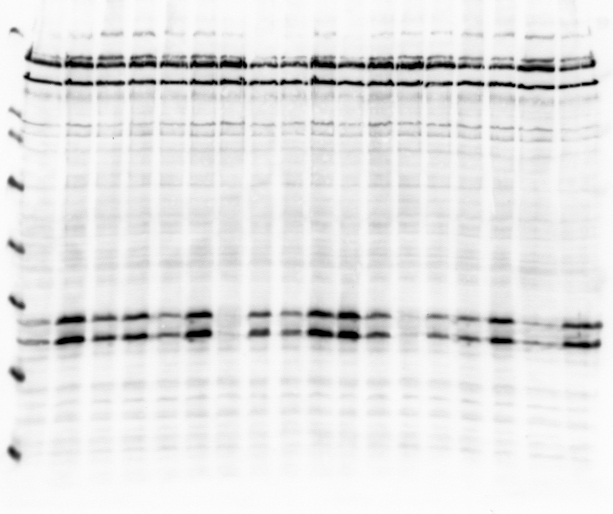

Supplement: Supplementary file 5 — Source Data for Appendix [file MSB-13-904-s013.zip › Source_Data_for_Appendix/Figure_S10/panel_B/BaF3_ppERK.jpg]

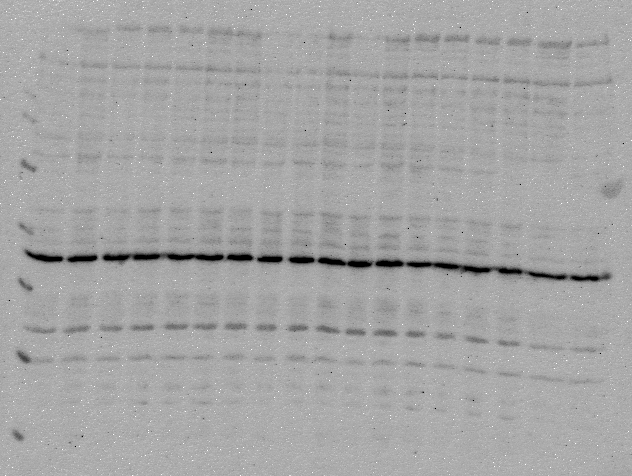

Supplement: Supplementary file 5 — Source Data for Appendix [file MSB-13-904-s013.zip › Source_Data_for_Appendix/Figure_S10/panel_B/BaF3_PDI.tif]

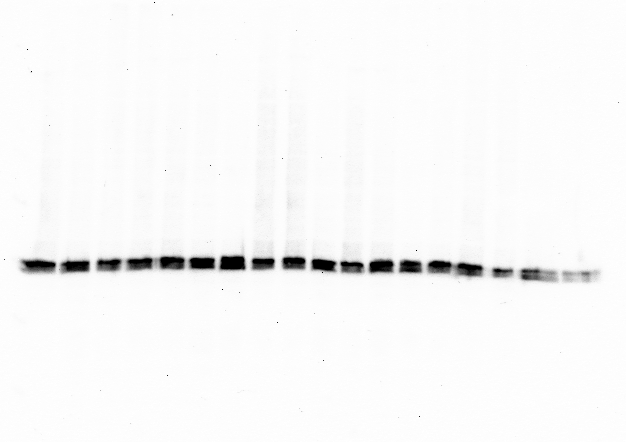

Supplement: Supplementary file 5 — Source Data for Appendix [file MSB-13-904-s013.zip › Source_Data_for_Appendix/Figure_S10/panel_B/BaF3_AKT.tif]

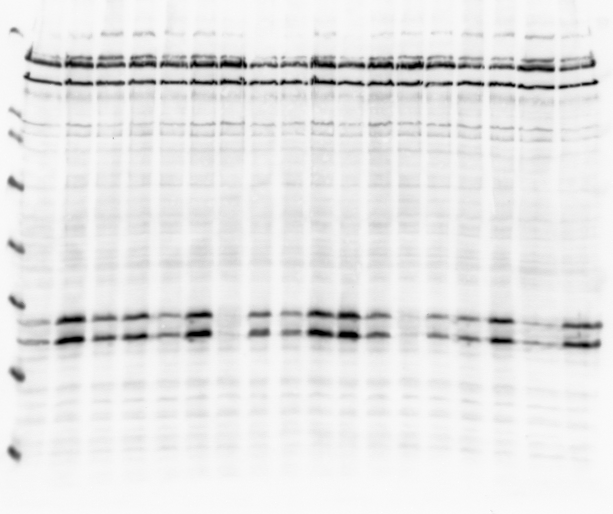

Supplement: Supplementary file 5 — Source Data for Appendix [file MSB-13-904-s013.zip › Source_Data_for_Appendix/Figure_S10/panel_B/BaF3_ppERK.tif]

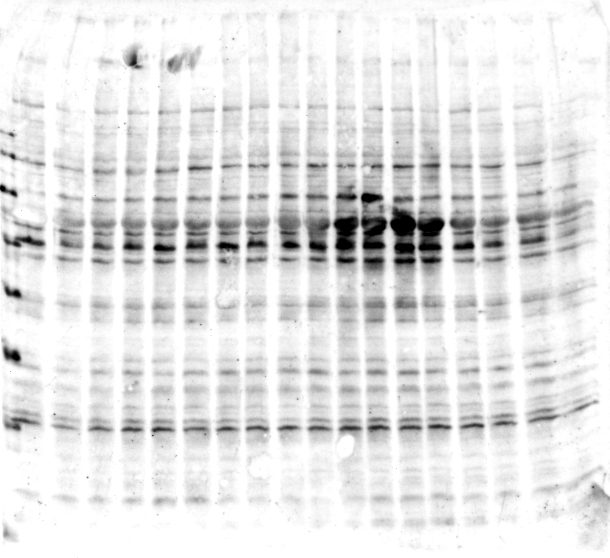

Supplement: Supplementary file 5 — Source Data for Appendix [file MSB-13-904-s013.zip › Source_Data_for_Appendix/Figure_S10/panel_B/CFUE_AKT.jpg]

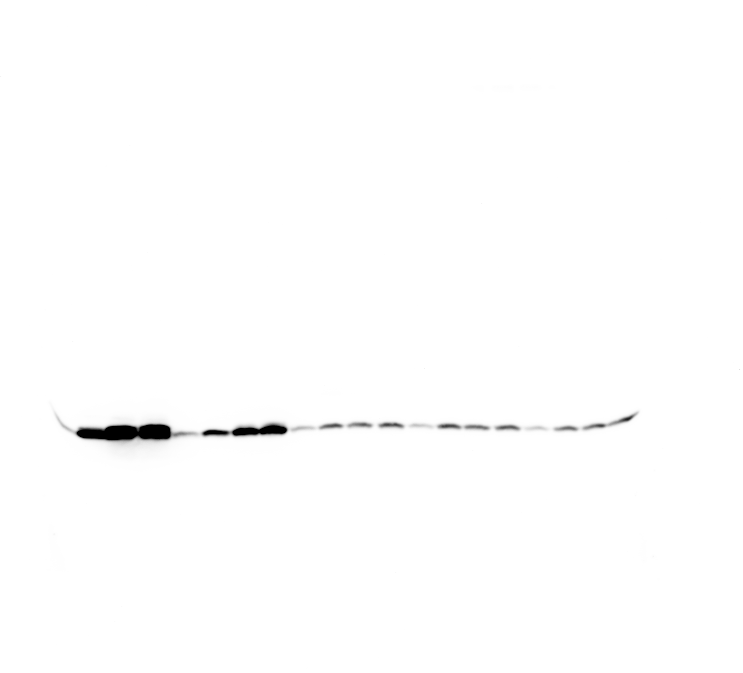

Supplement: Supplementary file 5 — Source Data for Appendix [file MSB-13-904-s013.zip › Source_Data_for_Appendix/Figure_S05/panel_A/CFUE_Rapamycin_pS6.tif]

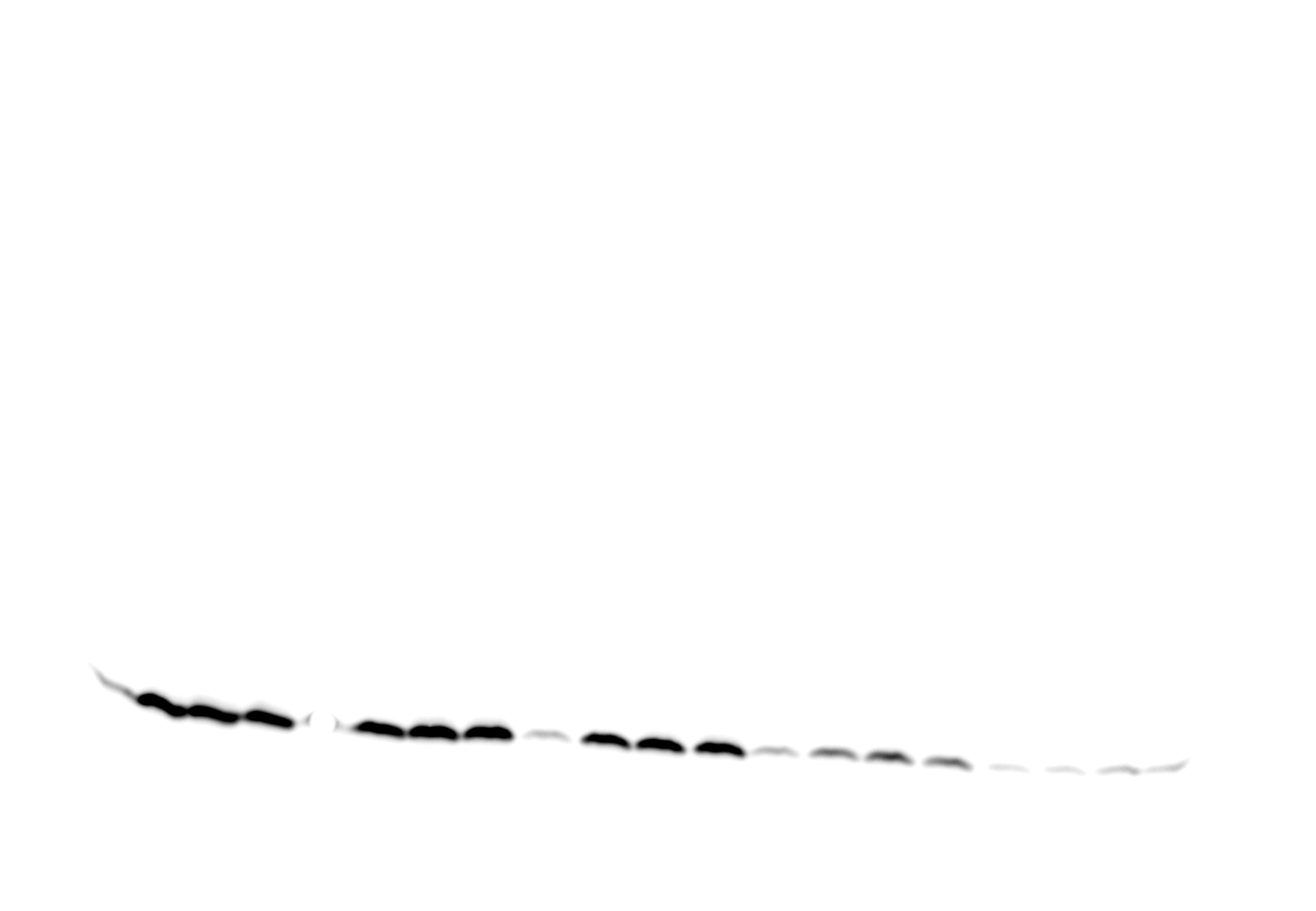

Supplement: Supplementary file 5 — Source Data for Appendix [file MSB-13-904-s013.zip › Source_Data_for_Appendix/Figure_S05/panel_A/BaF3_BI-D1870_pS6.jpg]

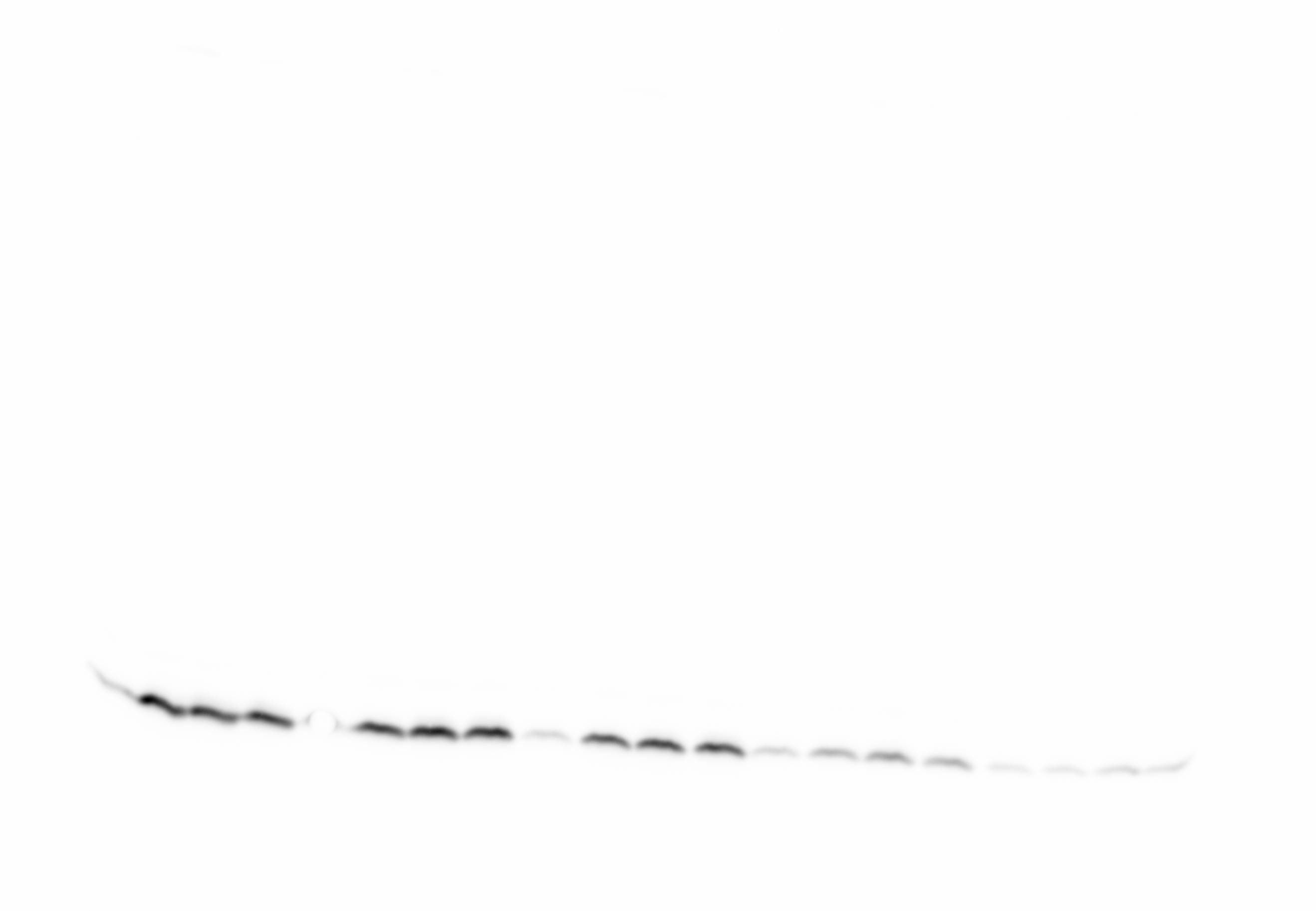

Supplement: Supplementary file 5 — Source Data for Appendix [file MSB-13-904-s013.zip › Source_Data_for_Appendix/Figure_S05/panel_A/BaF3_BI-D1870_pS6.tif]

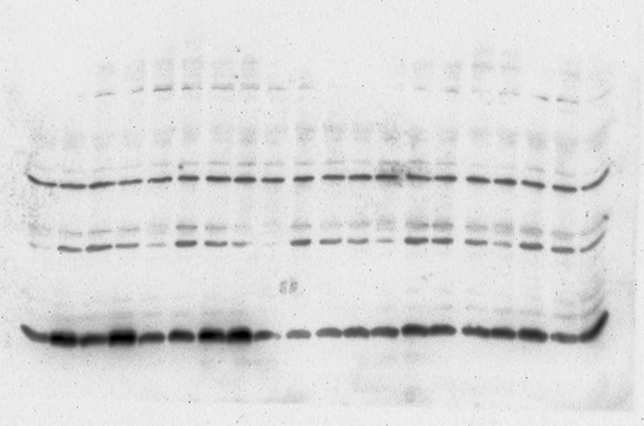

Supplement: Supplementary file 5 — Source Data for Appendix [file MSB-13-904-s013.zip › Source_Data_for_Appendix/Figure_S05/panel_A/BaF3_Rapamycin_PDI.tif]

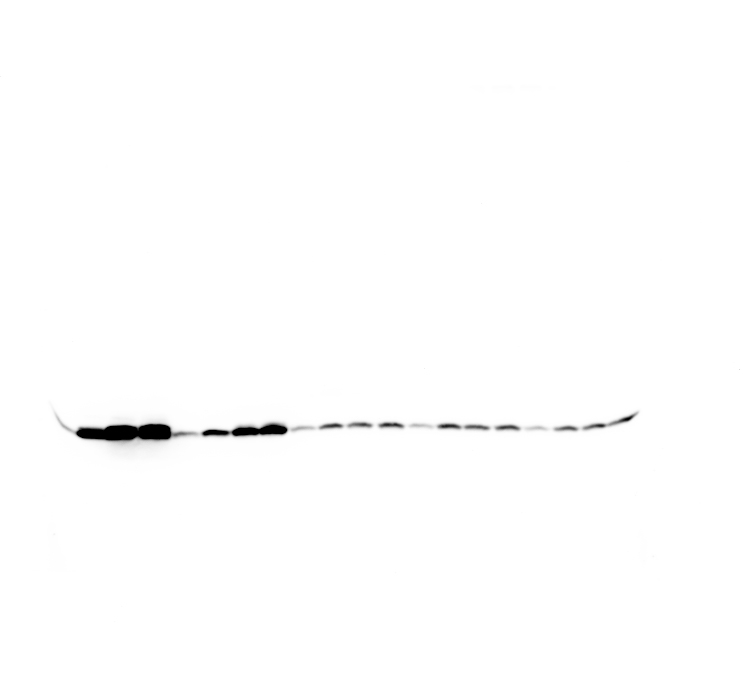

Supplement: Supplementary file 5 — Source Data for Appendix [file MSB-13-904-s013.zip › Source_Data_for_Appendix/Figure_S05/panel_A/CFUE_Rapamycin_pS6.jpg]

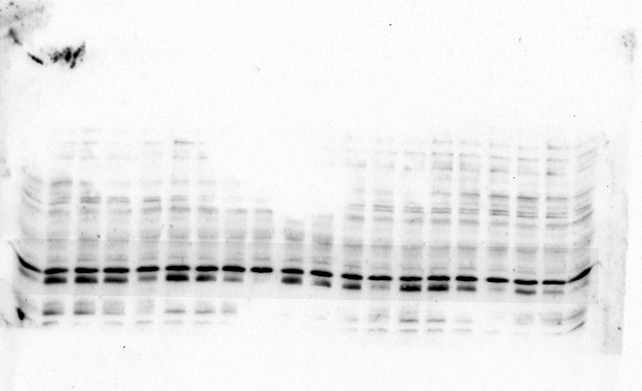

Supplement: Supplementary file 5 — Source Data for Appendix [file MSB-13-904-s013.zip › Source_Data_for_Appendix/Figure_S05/panel_A/CFUE_BI-D1870_PDI.jpg]

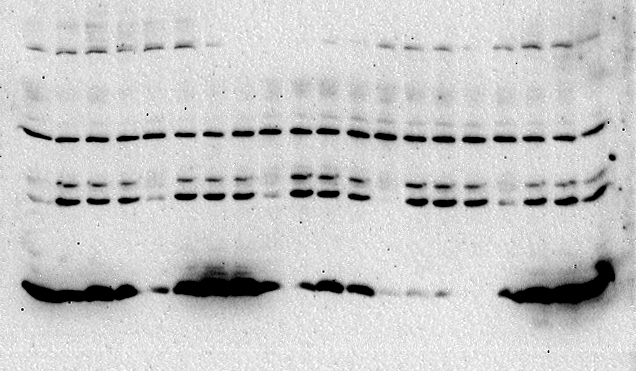

Supplement: Supplementary file 5 — Source Data for Appendix [file MSB-13-904-s013.zip › Source_Data_for_Appendix/Figure_S05/panel_A/BaF3_BI-D1870_PDI.jpg]

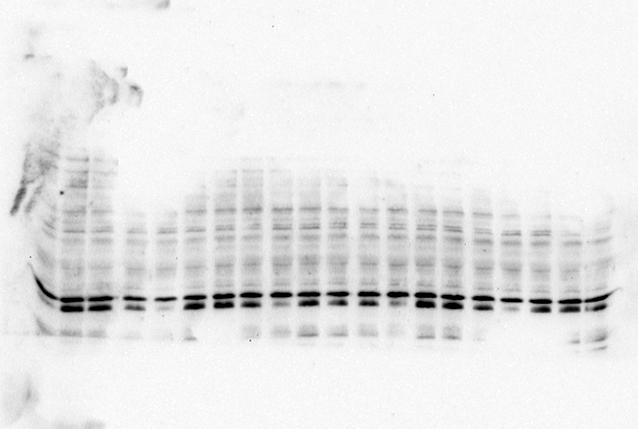

Supplement: Supplementary file 5 — Source Data for Appendix [file MSB-13-904-s013.zip › Source_Data_for_Appendix/Figure_S05/panel_A/CFUE_Rapamycin_PDI.jpg]

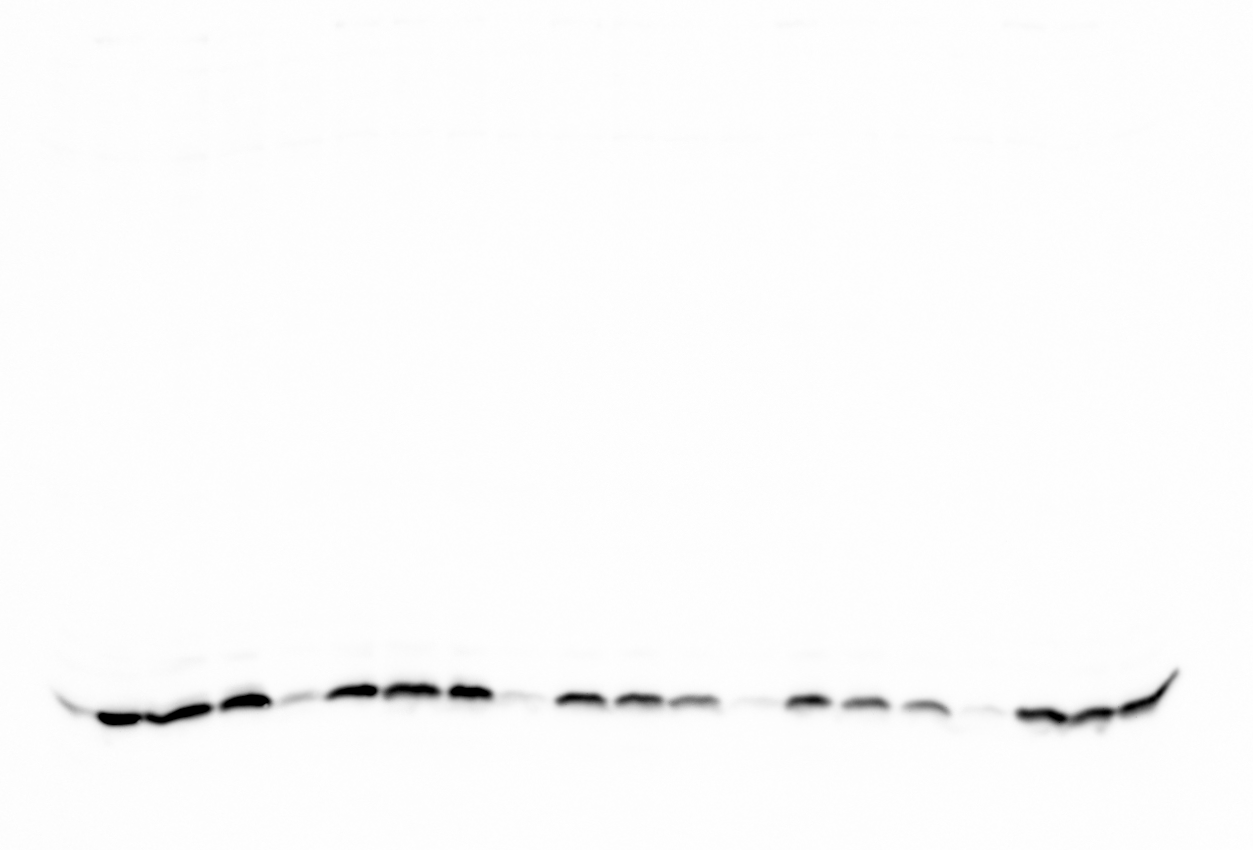

Supplement: Supplementary file 5 — Source Data for Appendix [file MSB-13-904-s013.zip › Source_Data_for_Appendix/Figure_S05/panel_A/BaF3_Rapamycin_pS6.tif]

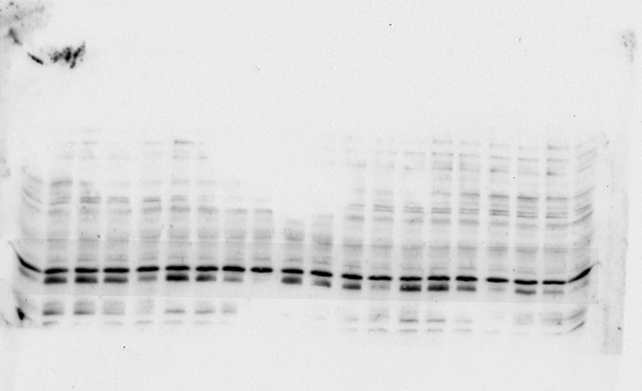

Supplement: Supplementary file 5 — Source Data for Appendix [file MSB-13-904-s013.zip › Source_Data_for_Appendix/Figure_S05/panel_A/CFUE_BI-D1870_PDI.tif]

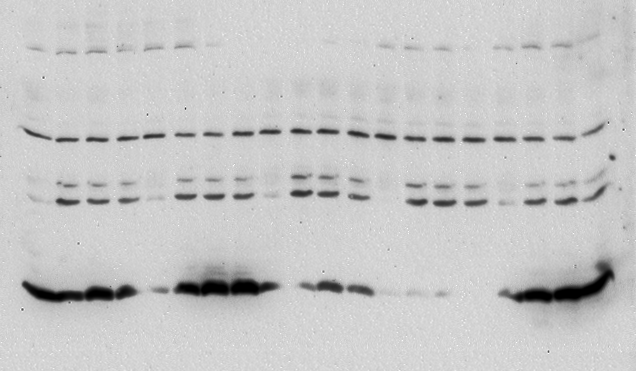

Supplement: Supplementary file 5 — Source Data for Appendix [file MSB-13-904-s013.zip › Source_Data_for_Appendix/Figure_S05/panel_A/BaF3_BI-D1870_PDI.tif]

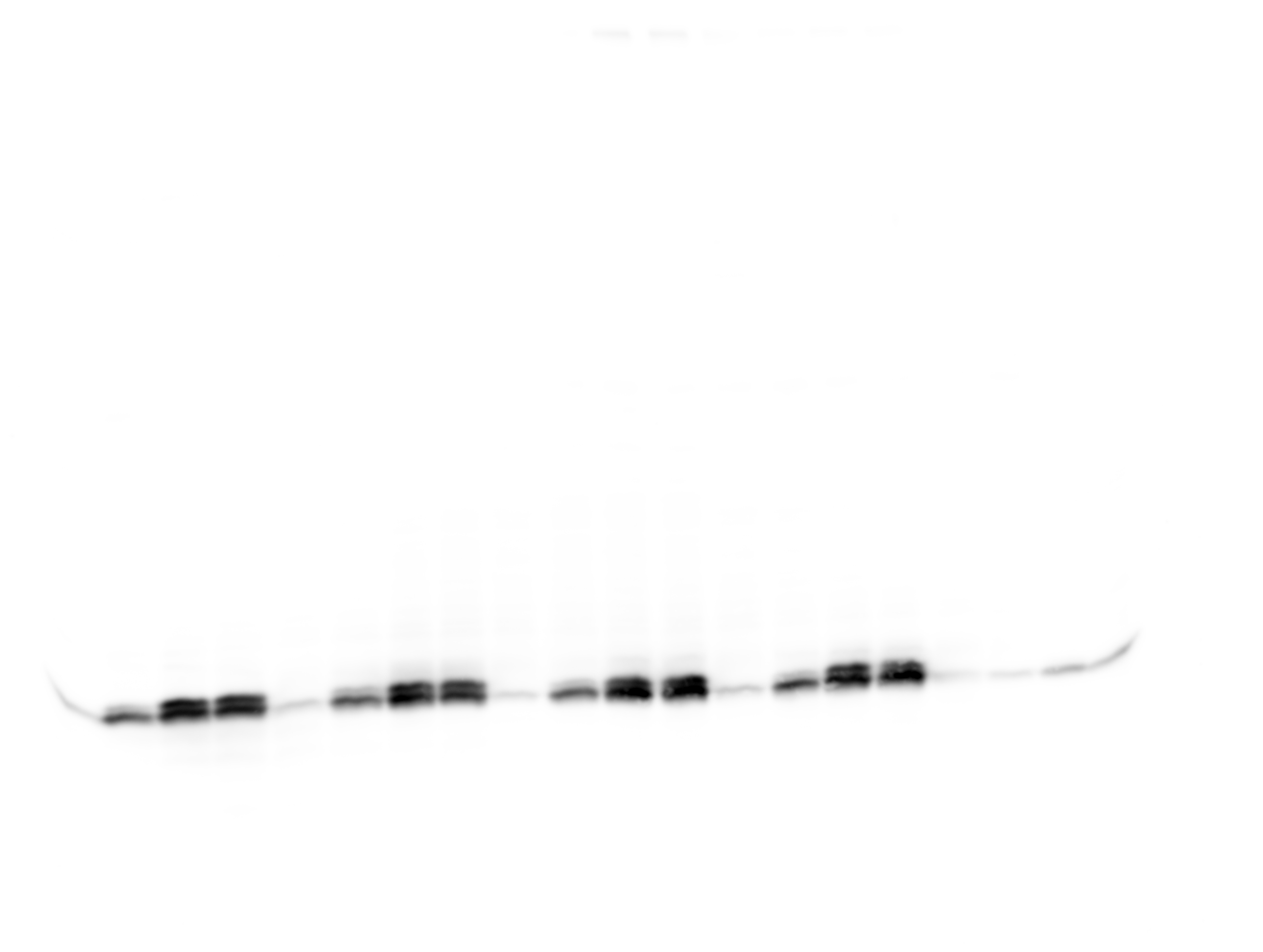

Supplement: Supplementary file 5 — Source Data for Appendix [file MSB-13-904-s013.zip › Source_Data_for_Appendix/Figure_S05/panel_A/CFUE_BI-D1870_pS6.jpg]

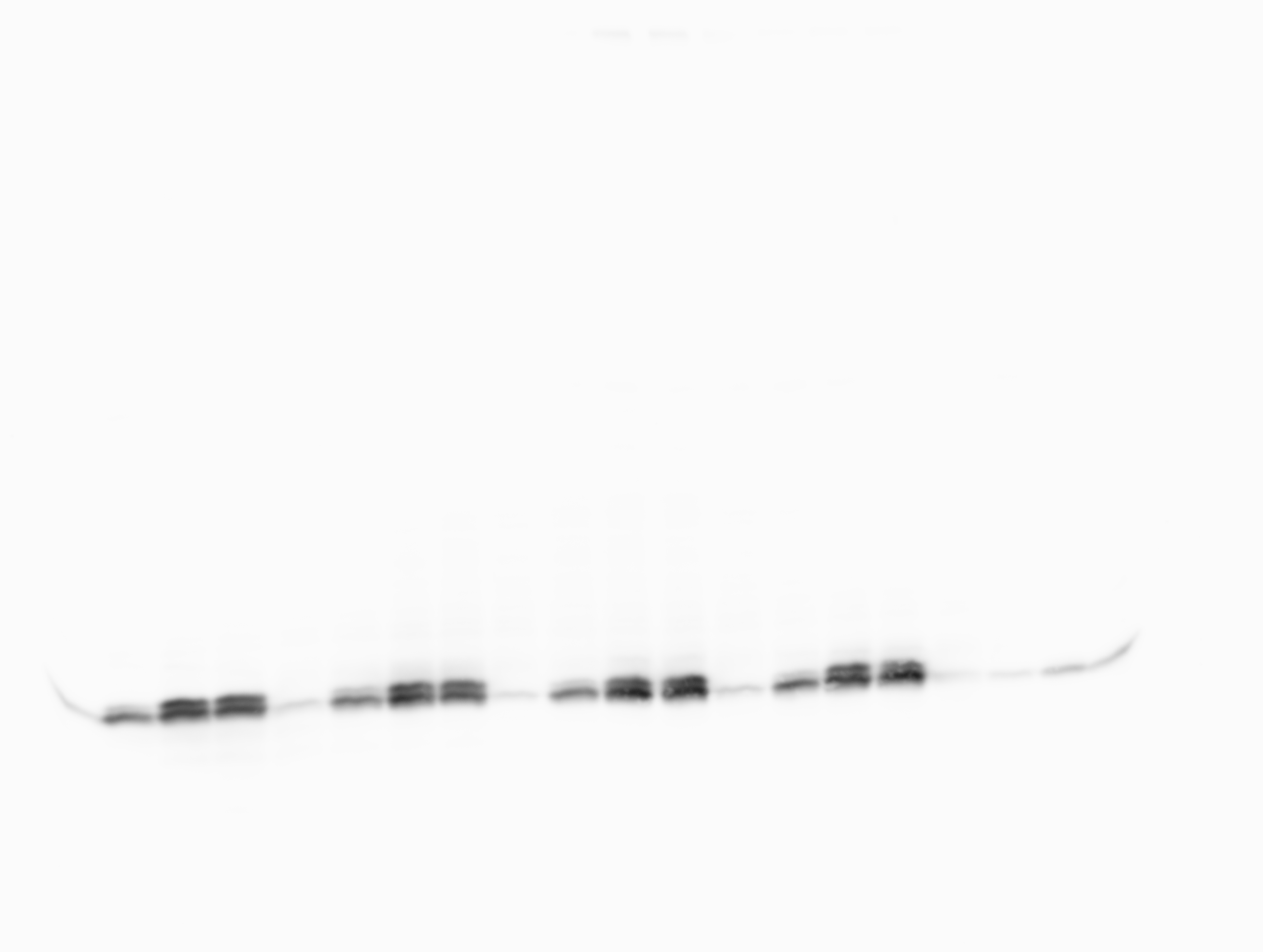

Supplement: Supplementary file 5 — Source Data for Appendix [file MSB-13-904-s013.zip › Source_Data_for_Appendix/Figure_S05/panel_A/CFUE_BI-D1870_pS6.tif]

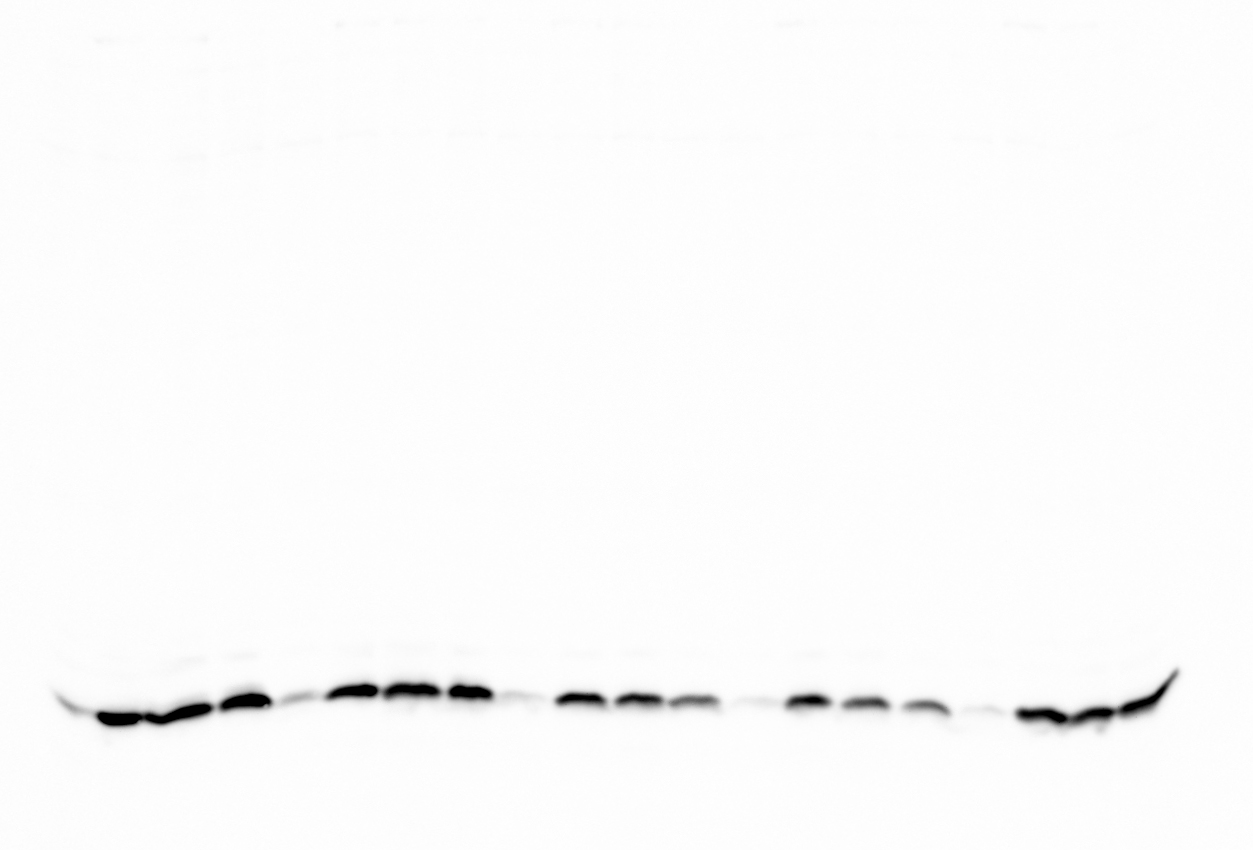

Supplement: Supplementary file 5 — Source Data for Appendix [file MSB-13-904-s013.zip › Source_Data_for_Appendix/Figure_S05/panel_A/BaF3_Rapamycin_pS6.jpg]

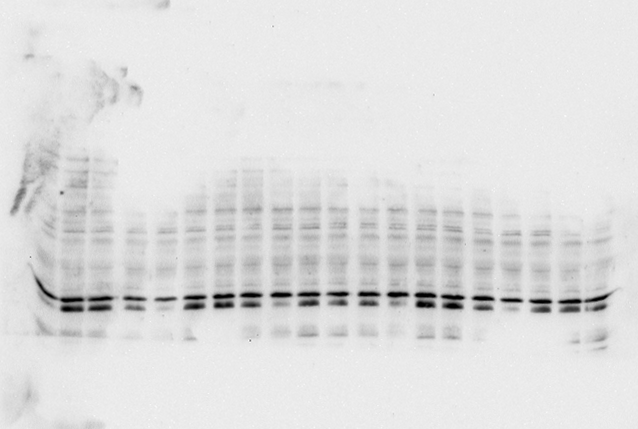

Supplement: Supplementary file 5 — Source Data for Appendix [file MSB-13-904-s013.zip › Source_Data_for_Appendix/Figure_S05/panel_A/CFUE_Rapamycin_PDI.tif]

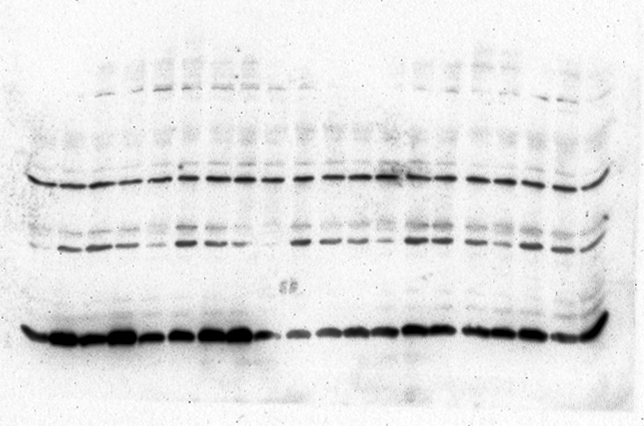

Supplement: Supplementary file 5 — Source Data for Appendix [file MSB-13-904-s013.zip › Source_Data_for_Appendix/Figure_S05/panel_A/BaF3_Rapamycin_PDI.jpg]

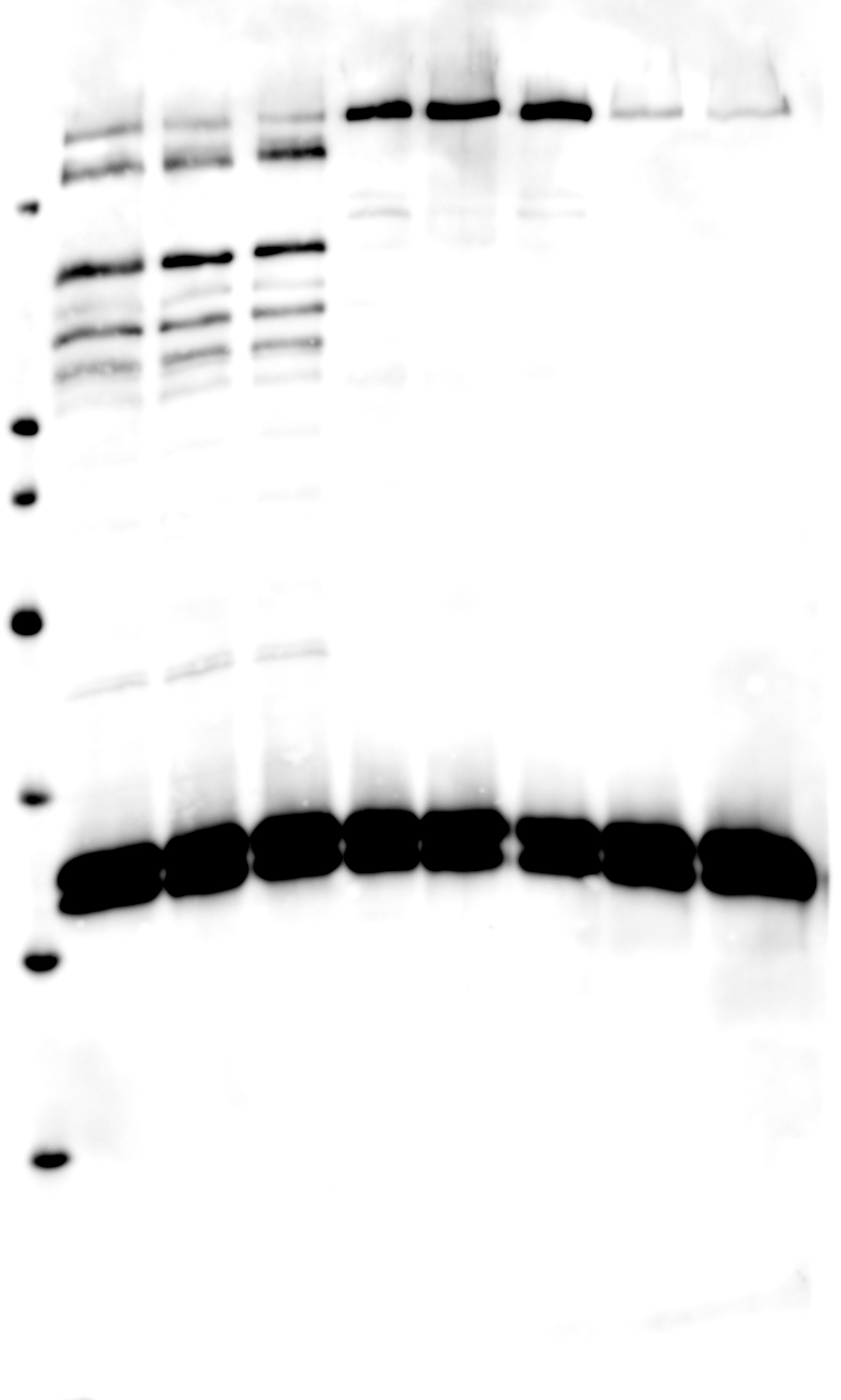

Supplement: Supplementary file 5 — Source Data for Appendix [file MSB-13-904-s013.zip › Source_Data_for_Appendix/Figure_S07/panel_C/mTOR.jpg]

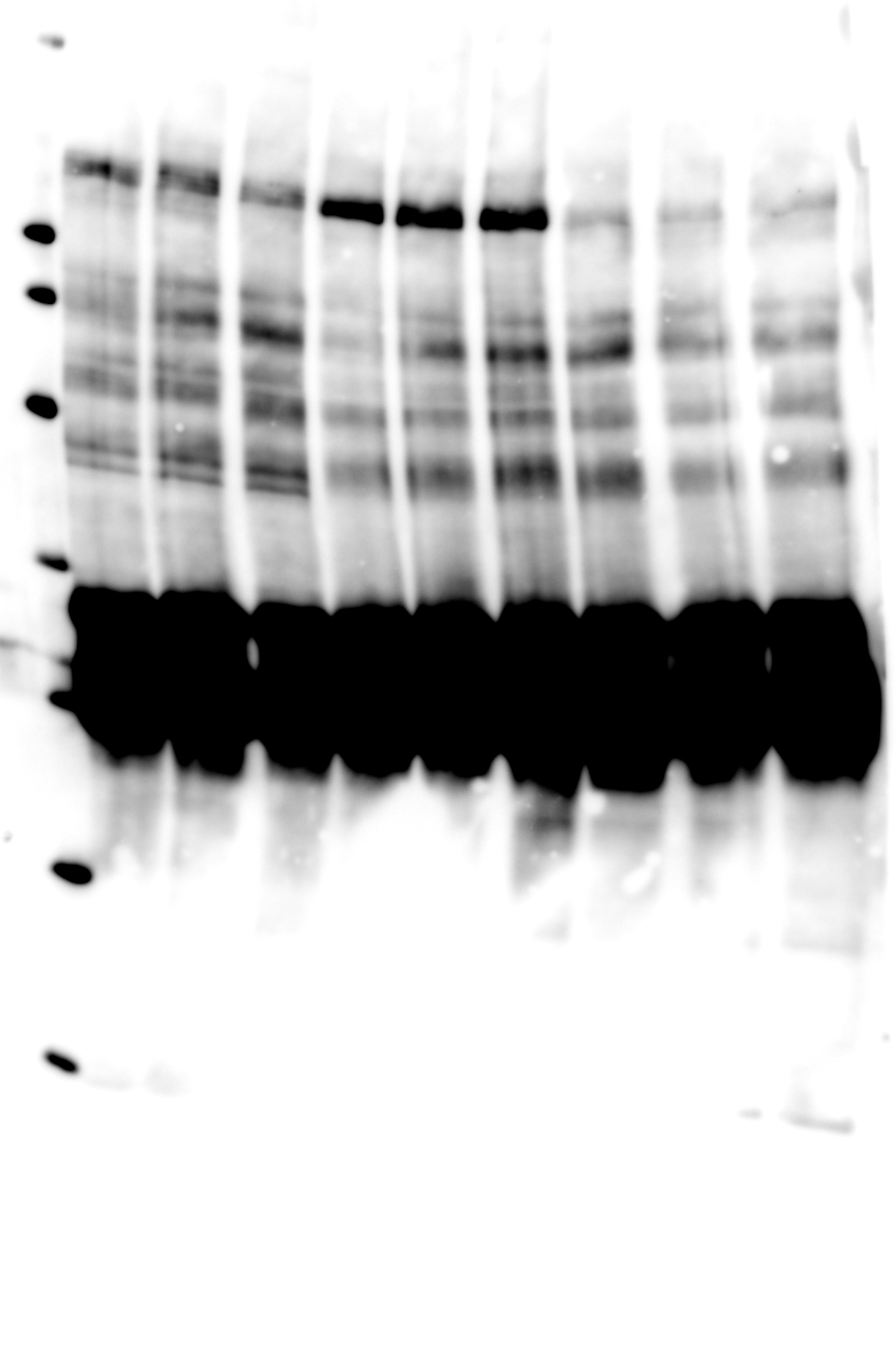

Supplement: Supplementary file 5 — Source Data for Appendix [file MSB-13-904-s013.zip › Source_Data_for_Appendix/Figure_S07/panel_C/Raptor.jpg]

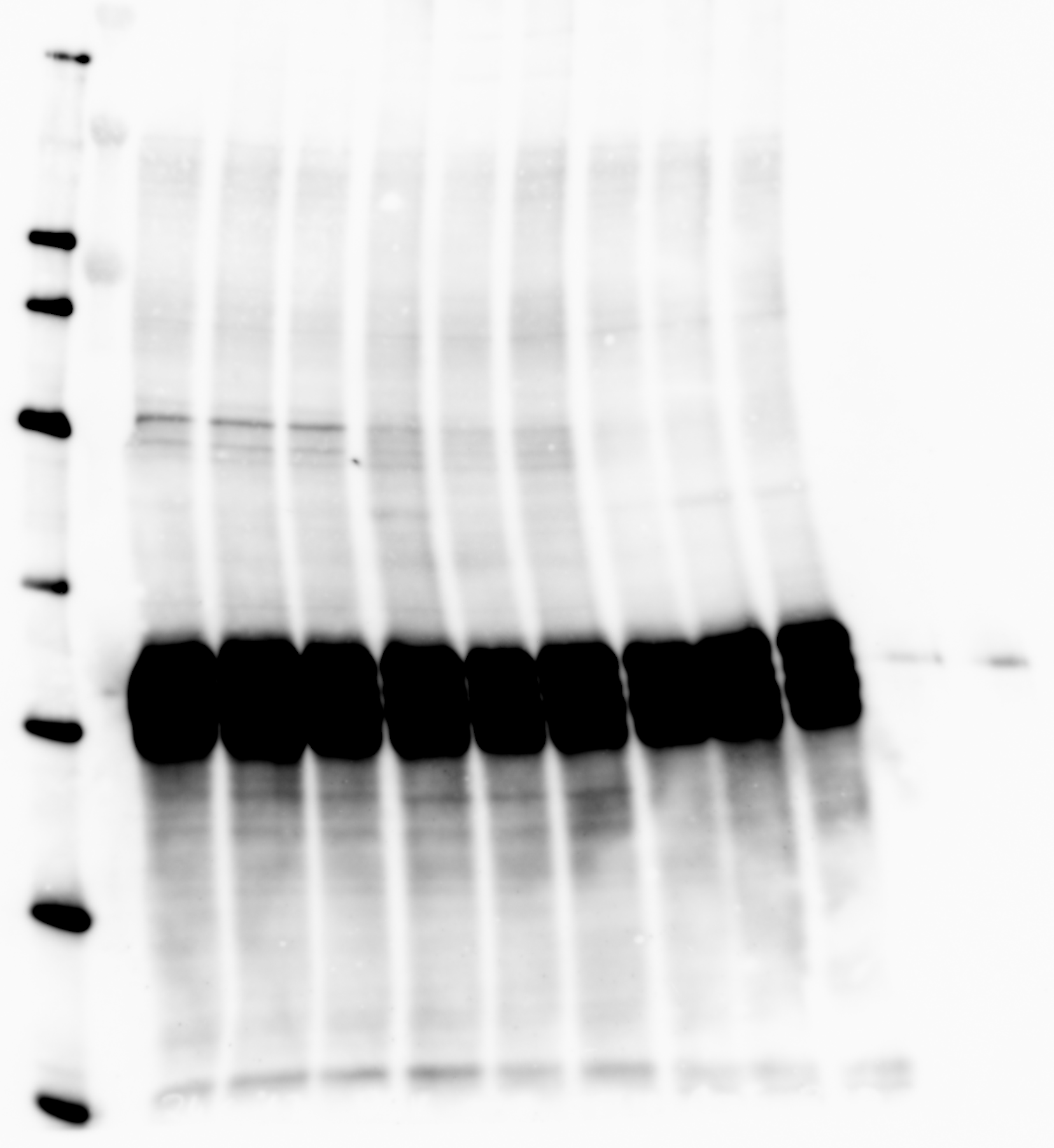

Supplement: Supplementary file 5 — Source Data for Appendix [file MSB-13-904-s013.zip › Source_Data_for_Appendix/Figure_S07/panel_C/RSK.tif]

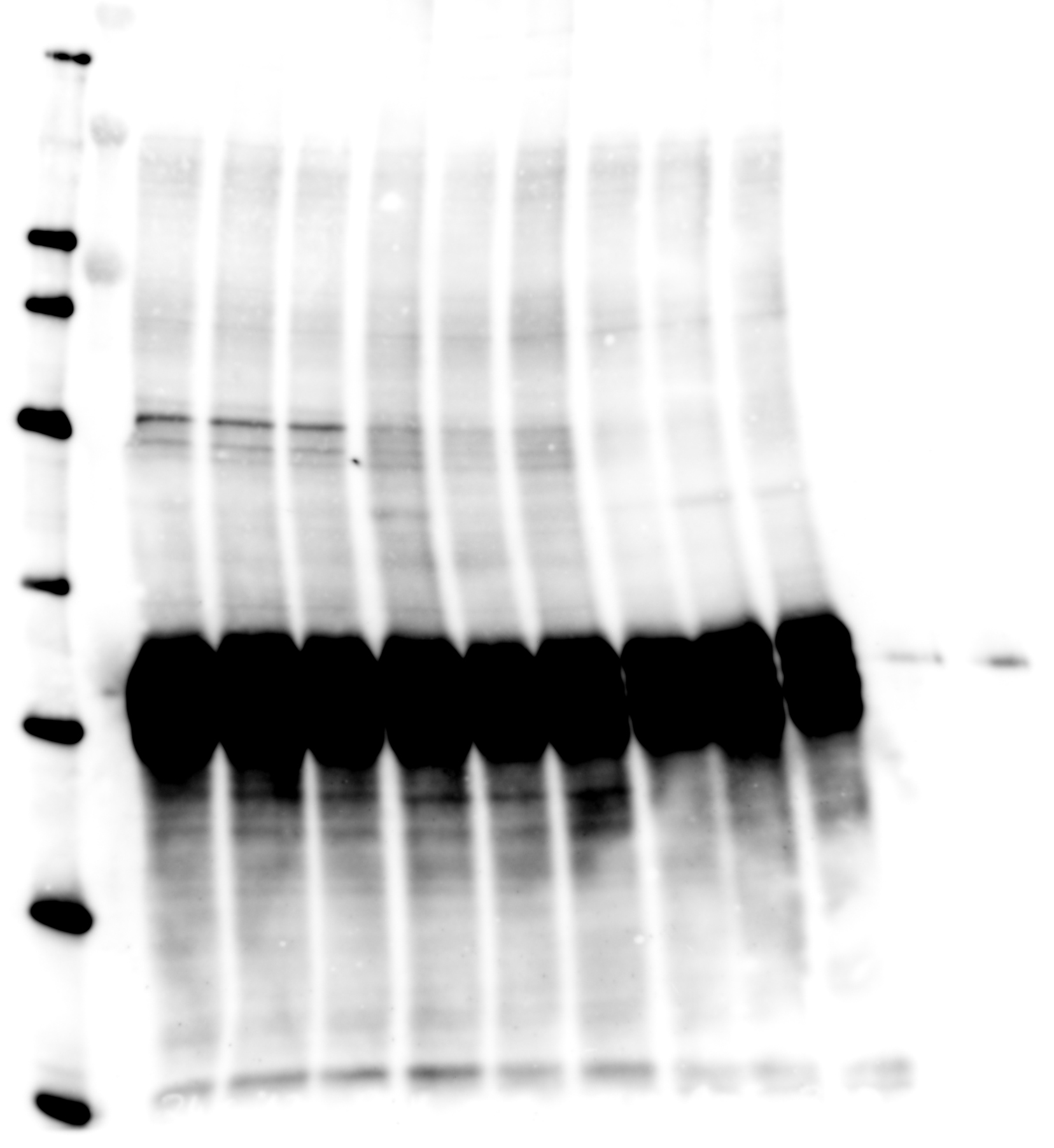

Supplement: Supplementary file 5 — Source Data for Appendix [file MSB-13-904-s013.zip › Source_Data_for_Appendix/Figure_S07/panel_C/RSK.jpg]

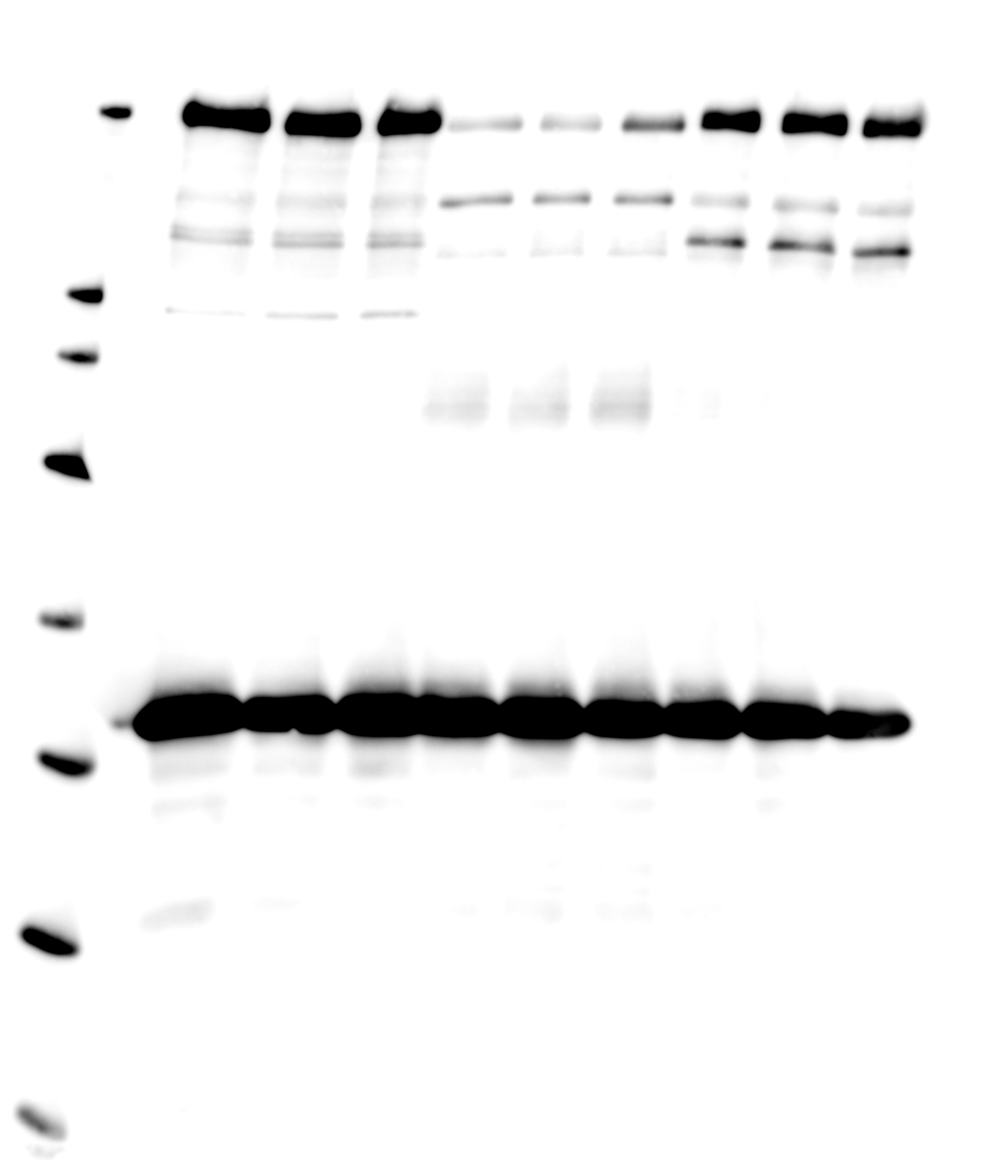

Supplement: Supplementary file 5 — Source Data for Appendix [file MSB-13-904-s013.zip › Source_Data_for_Appendix/Figure_S07/panel_C/Rictor.jpg]

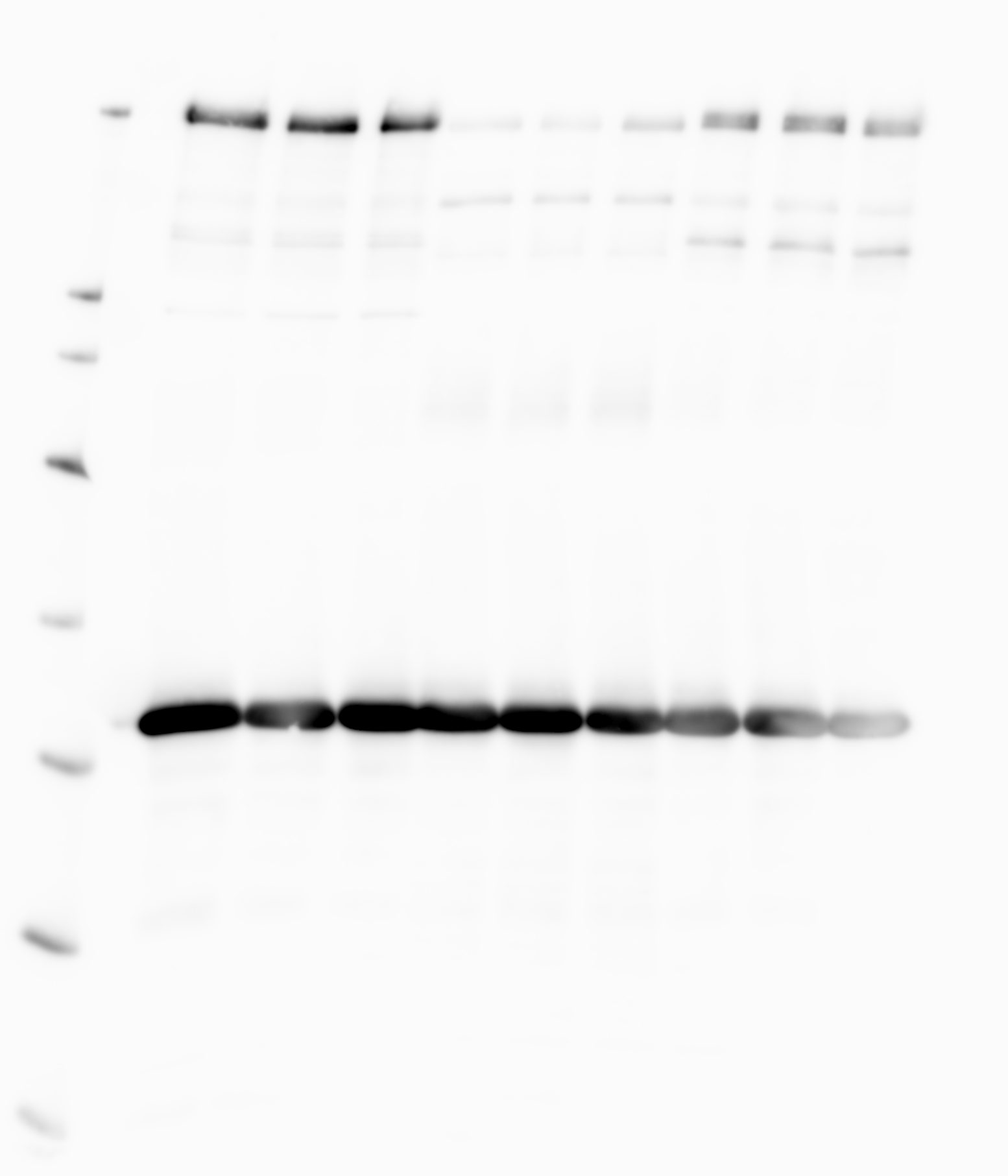

Supplement: Supplementary file 5 — Source Data for Appendix [file MSB-13-904-s013.zip › Source_Data_for_Appendix/Figure_S07/panel_C/Rictor.tif]

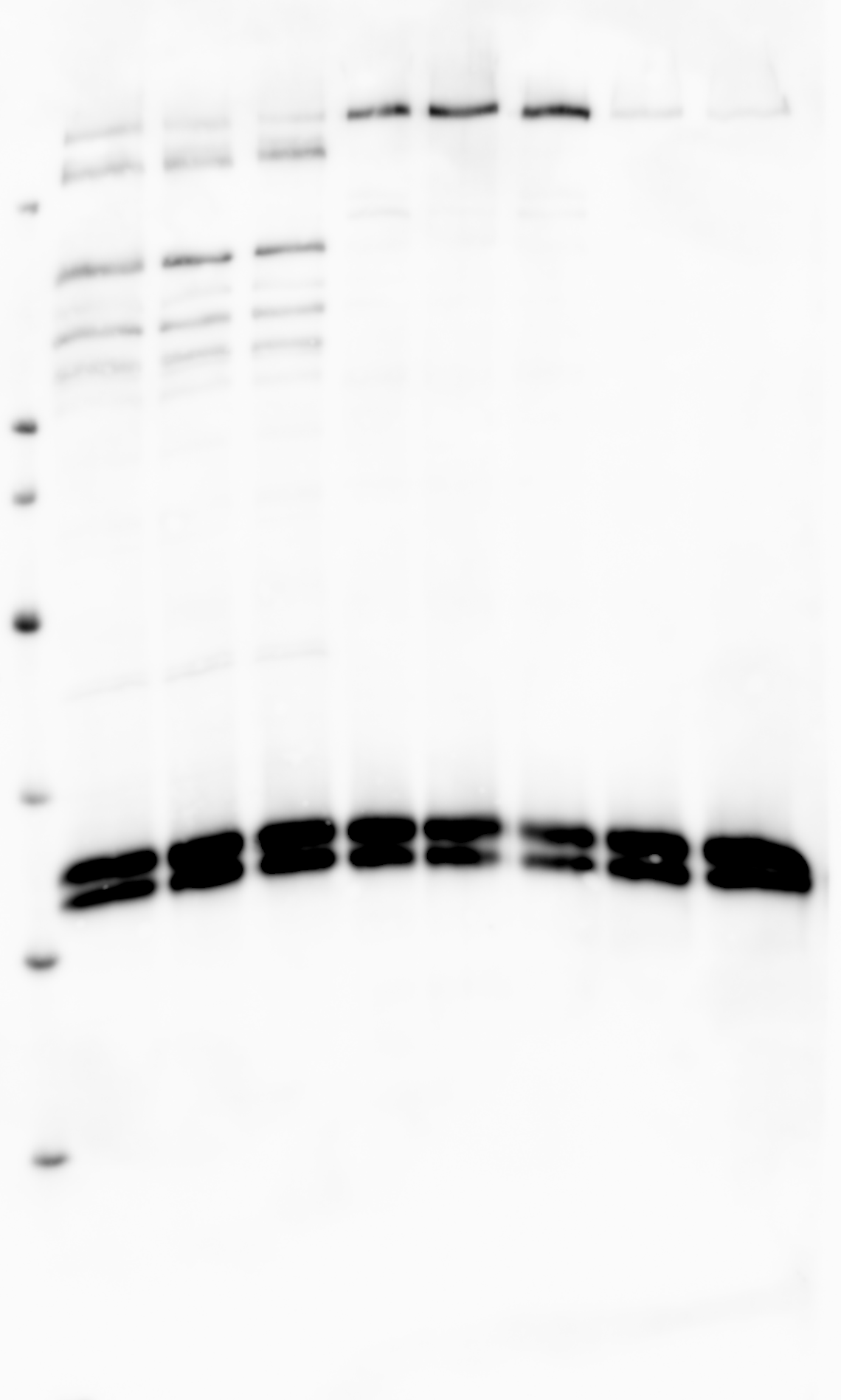

Supplement: Supplementary file 5 — Source Data for Appendix [file MSB-13-904-s013.zip › Source_Data_for_Appendix/Figure_S07/panel_C/mTOR.tif]

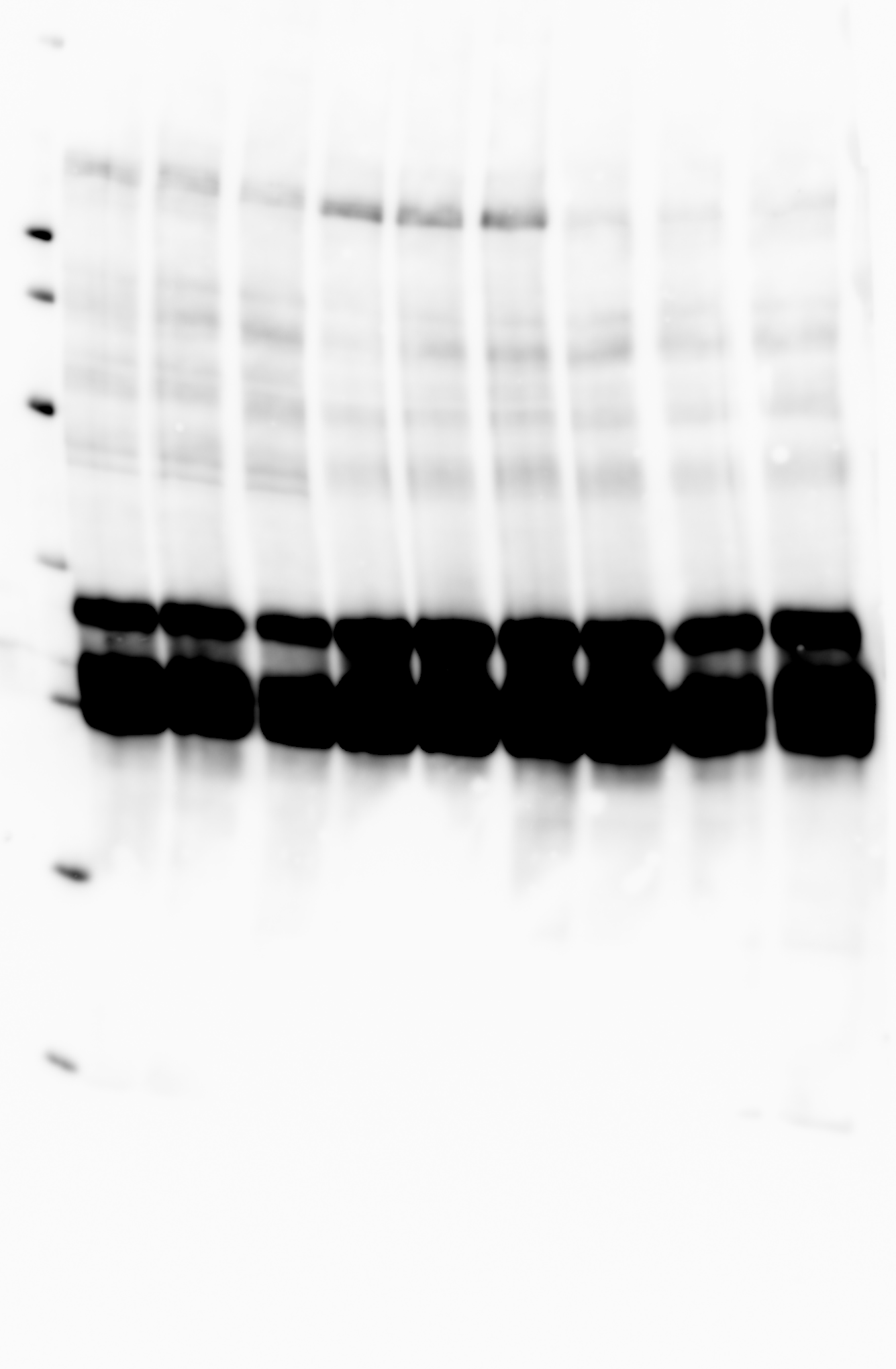

Supplement: Supplementary file 5 — Source Data for Appendix [file MSB-13-904-s013.zip › Source_Data_for_Appendix/Figure_S07/panel_C/Raptor.tif]

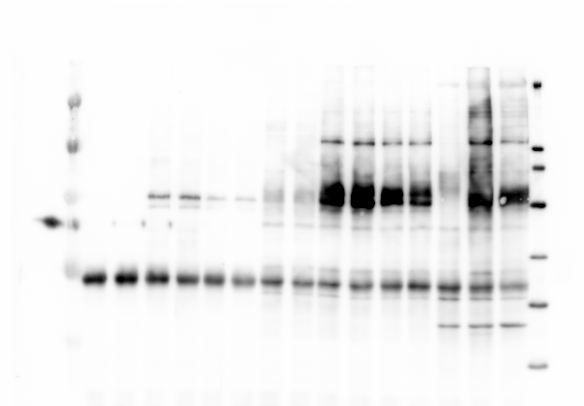

Supplement: Supplementary file 5 — Source Data for Appendix [file MSB-13-904-s013.zip › Source_Data_for_Appendix/Figure_S07/panel_A/BothPanels_pEpoR.tif]

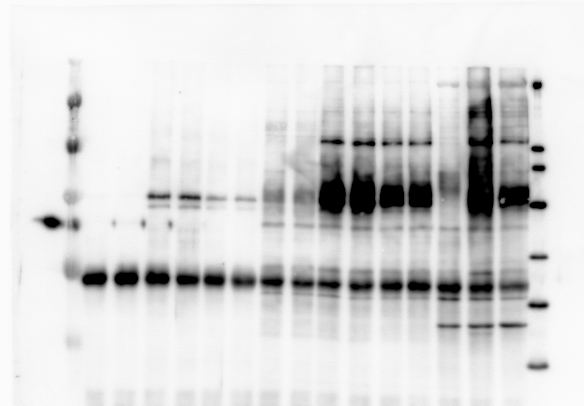

Supplement: Supplementary file 5 — Source Data for Appendix [file MSB-13-904-s013.zip › Source_Data_for_Appendix/Figure_S07/panel_A/LeftPanel_pEpoR.jpg]

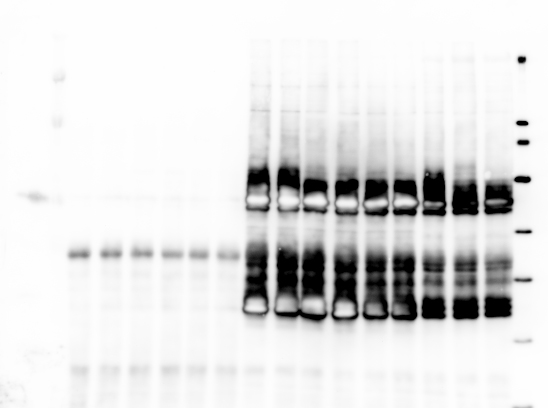

Supplement: Supplementary file 5 — Source Data for Appendix [file MSB-13-904-s013.zip › Source_Data_for_Appendix/Figure_S07/panel_A/LeftPanel_EpoR.jpg]

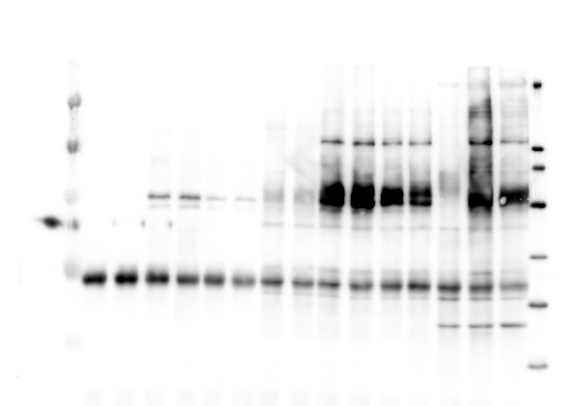

Supplement: Supplementary file 5 — Source Data for Appendix [file MSB-13-904-s013.zip › Source_Data_for_Appendix/Figure_S07/panel_A/RightPanel_pEpoR.jpg]

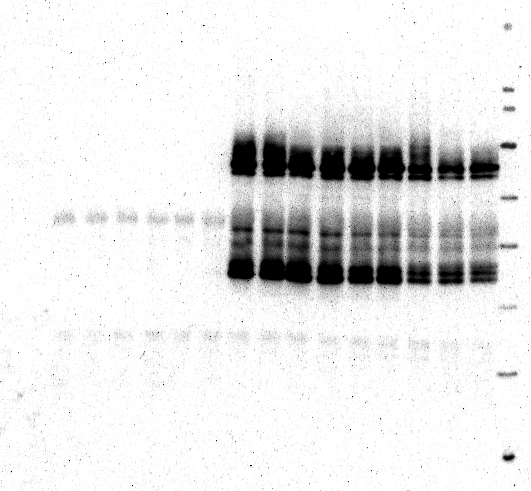

Supplement: Supplementary file 5 — Source Data for Appendix [file MSB-13-904-s013.zip › Source_Data_for_Appendix/Figure_S07/panel_A/RightPanel_EpoR.jpg]

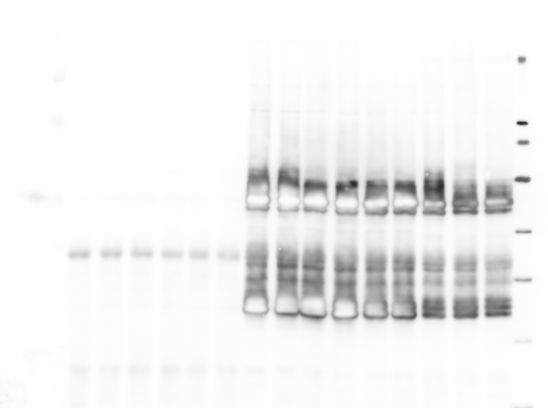

Supplement: Supplementary file 5 — Source Data for Appendix [file MSB-13-904-s013.zip › Source_Data_for_Appendix/Figure_S07/panel_A/LeftPanel_EpoR.tif]

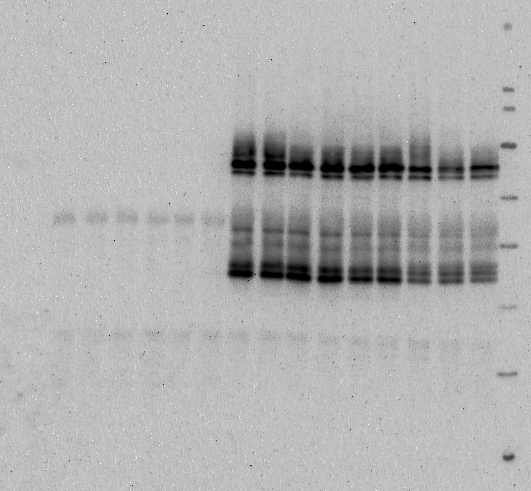

Supplement: Supplementary file 5 — Source Data for Appendix [file MSB-13-904-s013.zip › Source_Data_for_Appendix/Figure_S07/panel_A/RightPanel_EpoR.tif]

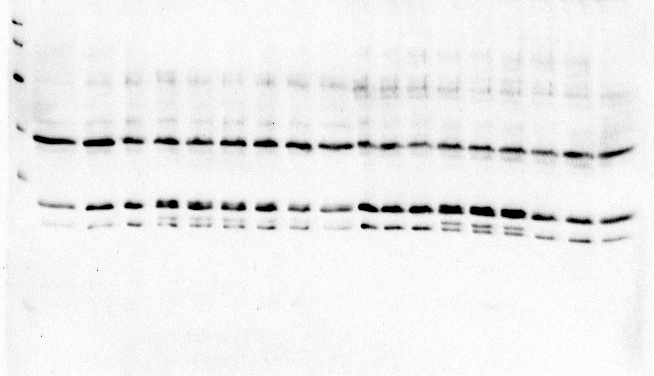

Supplement: Supplementary file 5 — Source Data for Appendix [file MSB-13-904-s013.zip › Source_Data_for_Appendix/Figure_S07/panel_B/RightPanel_PTEN.jpg]

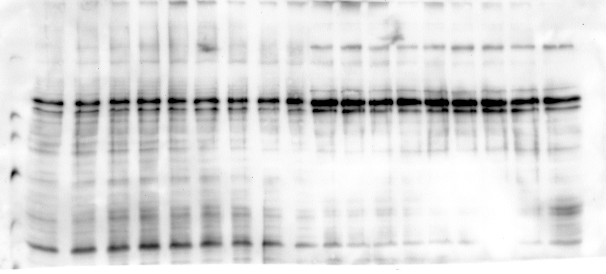

Supplement: Supplementary file 5 — Source Data for Appendix [file MSB-13-904-s013.zip › Source_Data_for_Appendix/Figure_S07/panel_B/RightPanel_SHIP1.jpg]

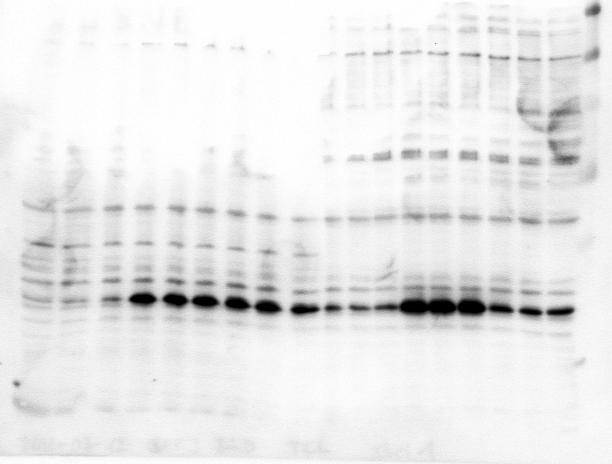

Supplement: Supplementary file 5 — Source Data for Appendix [file MSB-13-904-s013.zip › Source_Data_for_Appendix/Figure_S07/panel_B/RightPanel_pS6.jpg]

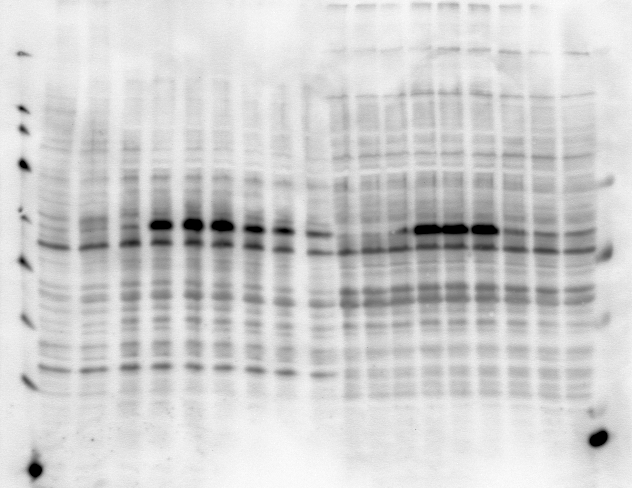

Supplement: Supplementary file 5 — Source Data for Appendix [file MSB-13-904-s013.zip › Source_Data_for_Appendix/Figure_S07/panel_B/RightPanel_pAKT.tif]

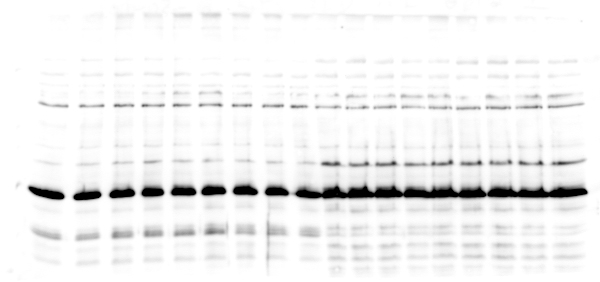

Supplement: Supplementary file 5 — Source Data for Appendix [file MSB-13-904-s013.zip › Source_Data_for_Appendix/Figure_S07/panel_B/RightPanel_p85.jpg]

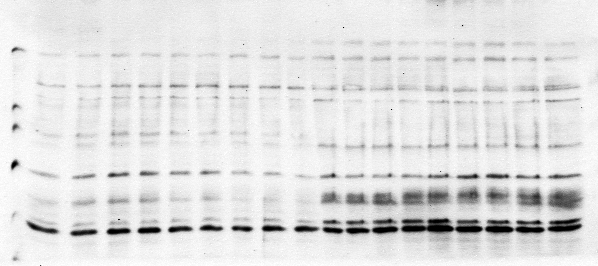

Supplement: Supplementary file 5 — Source Data for Appendix [file MSB-13-904-s013.zip › Source_Data_for_Appendix/Figure_S07/panel_B/RightPanel_PDK1.jpg]

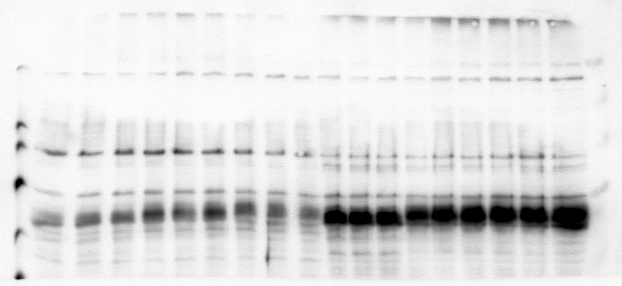

Supplement: Supplementary file 5 — Source Data for Appendix [file MSB-13-904-s013.zip › Source_Data_for_Appendix/Figure_S07/panel_B/RightPanel_Raf.jpg]

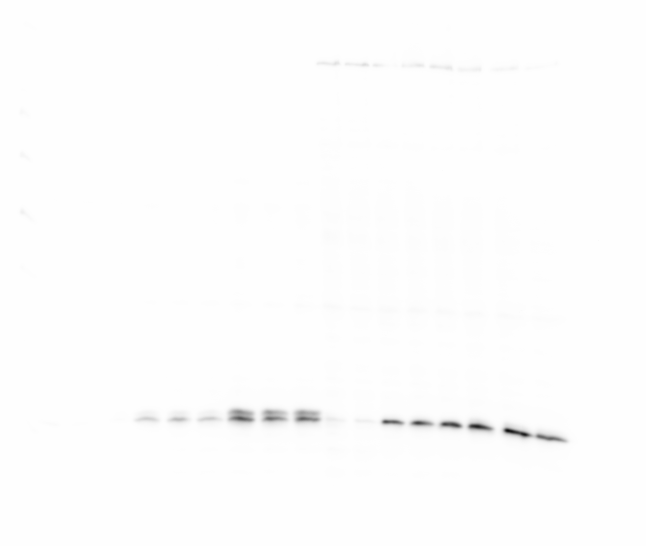

Supplement: Supplementary file 5 — Source Data for Appendix [file MSB-13-904-s013.zip › Source_Data_for_Appendix/Figure_S07/panel_B/LeftPanel_pS6.tif]

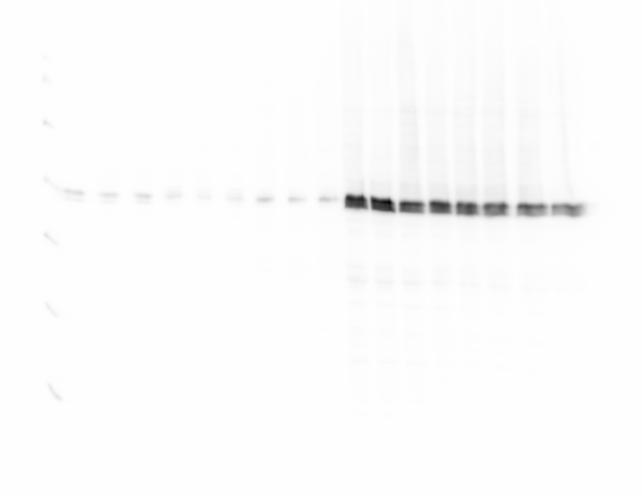

Supplement: Supplementary file 5 — Source Data for Appendix [file MSB-13-904-s013.zip › Source_Data_for_Appendix/Figure_S07/panel_B/LeftPanel_AKT.tif]

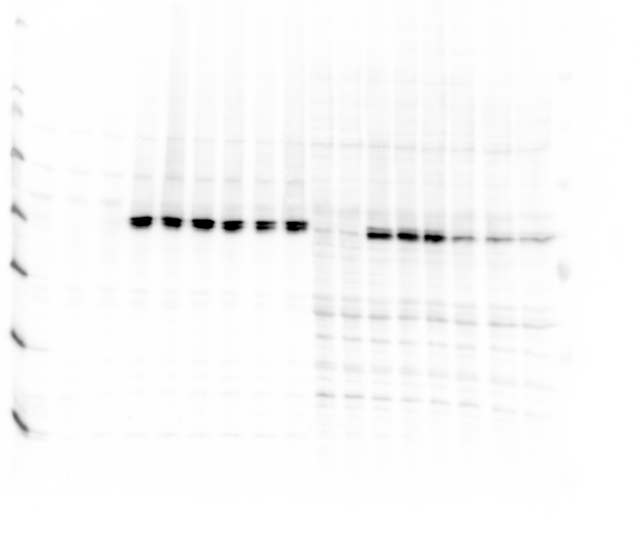

Supplement: Supplementary file 5 — Source Data for Appendix [file MSB-13-904-s013.zip › Source_Data_for_Appendix/Figure_S07/panel_B/LeftPanel_pAKT.tif]

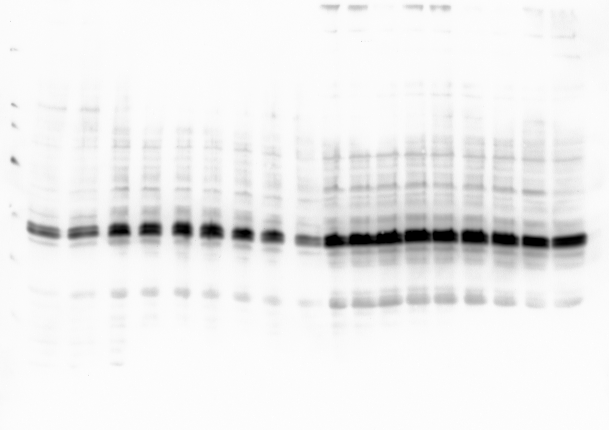

Supplement: Supplementary file 5 — Source Data for Appendix [file MSB-13-904-s013.zip › Source_Data_for_Appendix/Figure_S07/panel_B/RightPanel_AKT.tif]

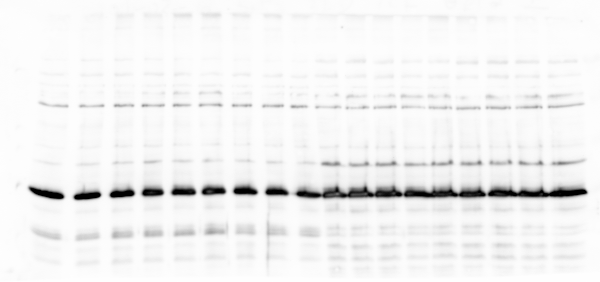

Supplement: Supplementary file 5 — Source Data for Appendix [file MSB-13-904-s013.zip › Source_Data_for_Appendix/Figure_S07/panel_B/RightPanel_p85.tif]

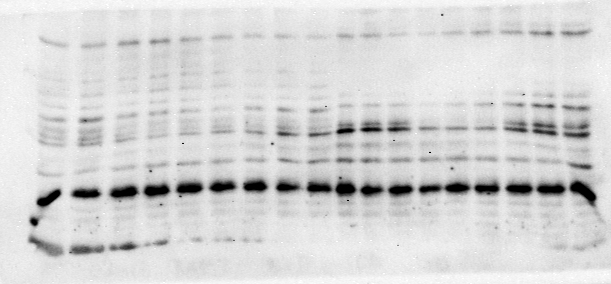

Supplement: Supplementary file 5 — Source Data for Appendix [file MSB-13-904-s013.zip › Source_Data_for_Appendix/Figure_S07/panel_B/RightPanel_Ras.jpg]

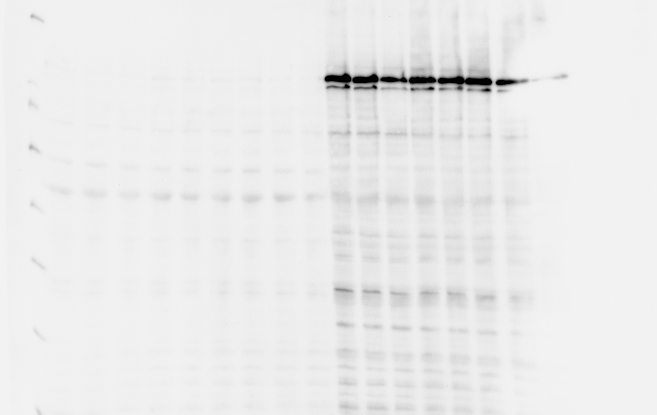

Supplement: Supplementary file 5 — Source Data for Appendix [file MSB-13-904-s013.zip › Source_Data_for_Appendix/Figure_S07/panel_B/LeftPanel_SHIP1.jpg]

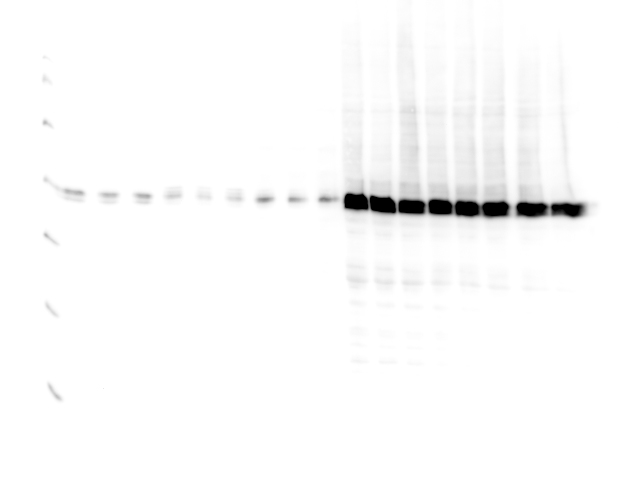

Supplement: Supplementary file 5 — Source Data for Appendix [file MSB-13-904-s013.zip › Source_Data_for_Appendix/Figure_S07/panel_B/LeftPanel_AKT.jpg]

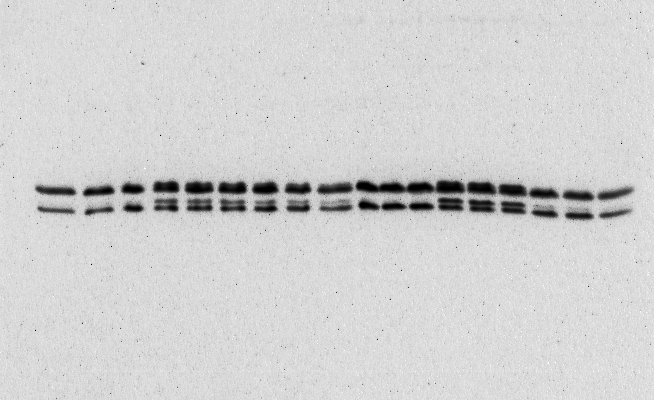

Supplement: Supplementary file 5 — Source Data for Appendix [file MSB-13-904-s013.zip › Source_Data_for_Appendix/Figure_S07/panel_B/RightPanel_ERK.tif]

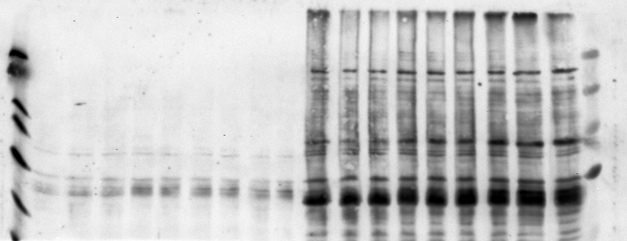

Supplement: Supplementary file 5 — Source Data for Appendix [file MSB-13-904-s013.zip › Source_Data_for_Appendix/Figure_S07/panel_B/LeftPanel_Raf.jpg]

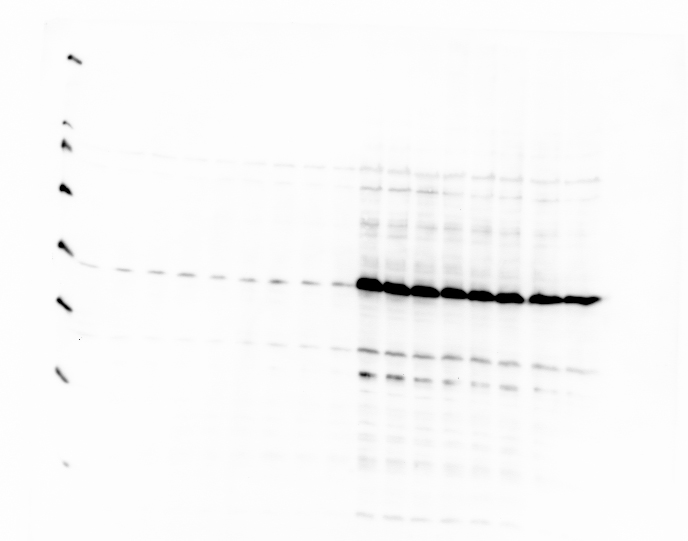

Supplement: Supplementary file 5 — Source Data for Appendix [file MSB-13-904-s013.zip › Source_Data_for_Appendix/Figure_S07/panel_B/LeftPanel_PTEN.jpg]

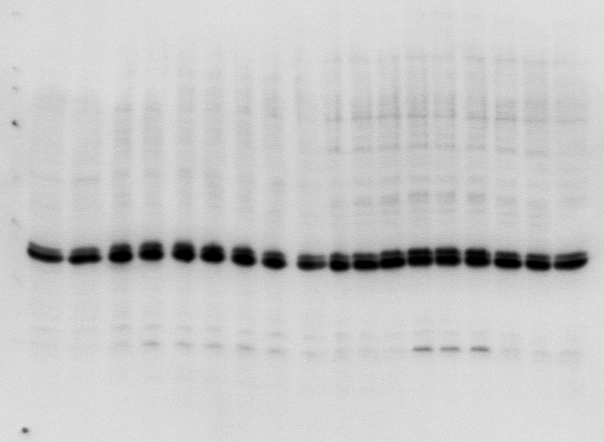

Supplement: Supplementary file 5 — Source Data for Appendix [file MSB-13-904-s013.zip › Source_Data_for_Appendix/Figure_S07/panel_B/RightPanel_MEK.tif]

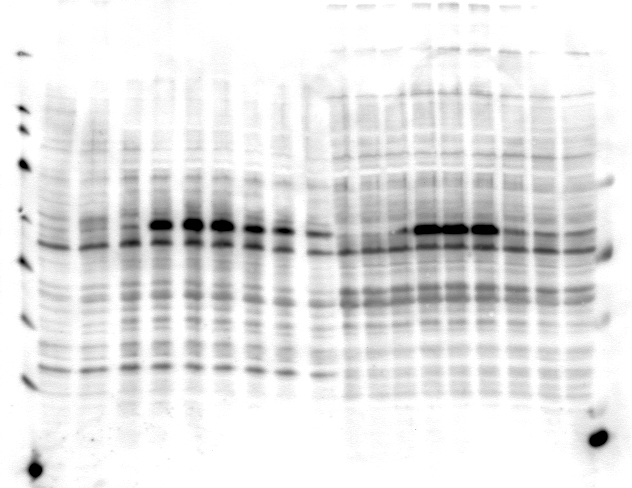

Supplement: Supplementary file 5 — Source Data for Appendix [file MSB-13-904-s013.zip › Source_Data_for_Appendix/Figure_S07/panel_B/RightPanel_pAKT.jpg]

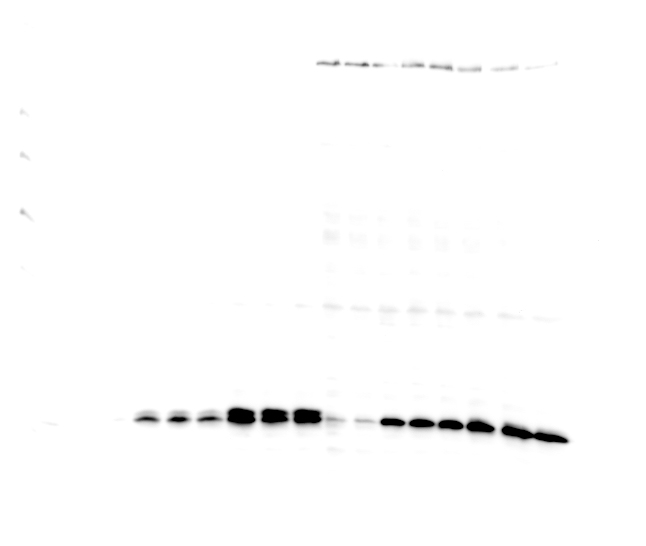

Supplement: Supplementary file 5 — Source Data for Appendix [file MSB-13-904-s013.zip › Source_Data_for_Appendix/Figure_S07/panel_B/LeftPanel_pS6.jpg]

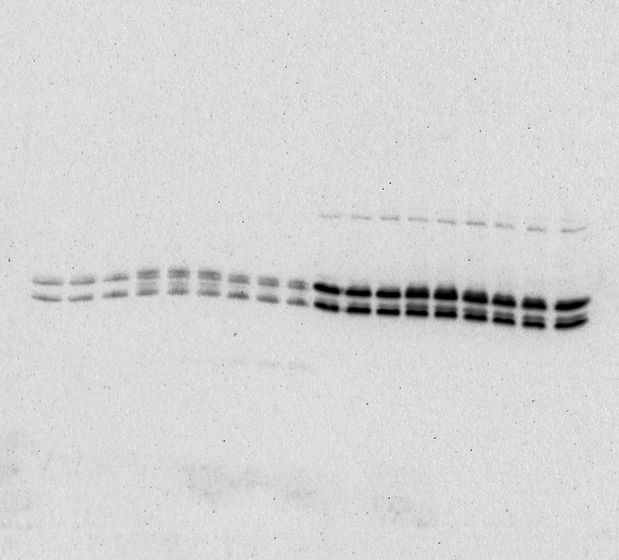

Supplement: Supplementary file 5 — Source Data for Appendix [file MSB-13-904-s013.zip › Source_Data_for_Appendix/Figure_S07/panel_B/LeftPanel_ERK.tif]

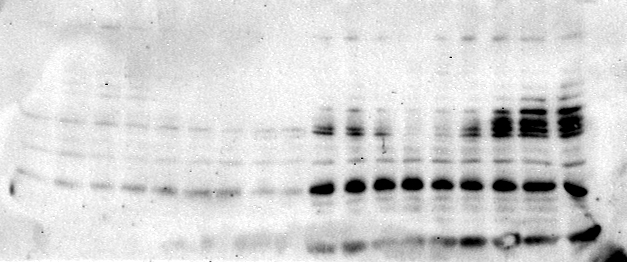

Supplement: Supplementary file 5 — Source Data for Appendix [file MSB-13-904-s013.zip › Source_Data_for_Appendix/Figure_S07/panel_B/LeftPanel_Ras.jpg]

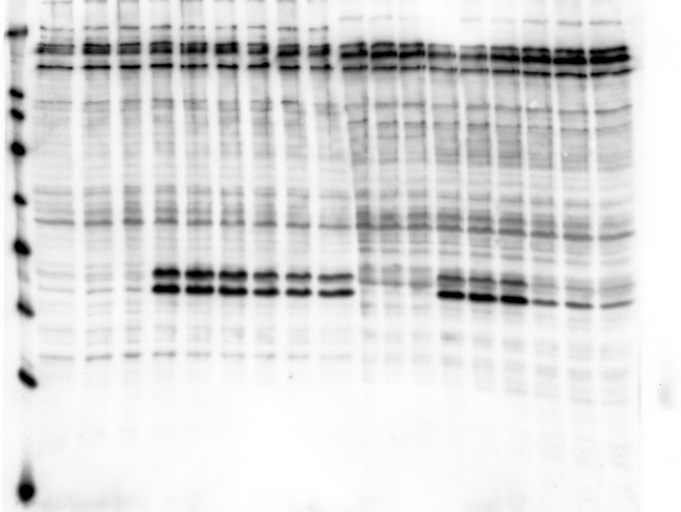

Supplement: Supplementary file 5 — Source Data for Appendix [file MSB-13-904-s013.zip › Source_Data_for_Appendix/Figure_S07/panel_B/RightPanel_ppERK.jpg]

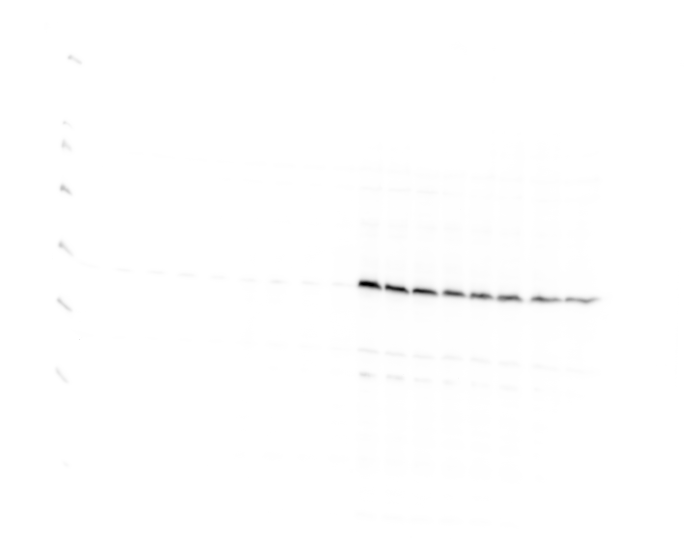

Supplement: Supplementary file 5 — Source Data for Appendix [file MSB-13-904-s013.zip › Source_Data_for_Appendix/Figure_S07/panel_B/LeftPanel_PTEN.tif]

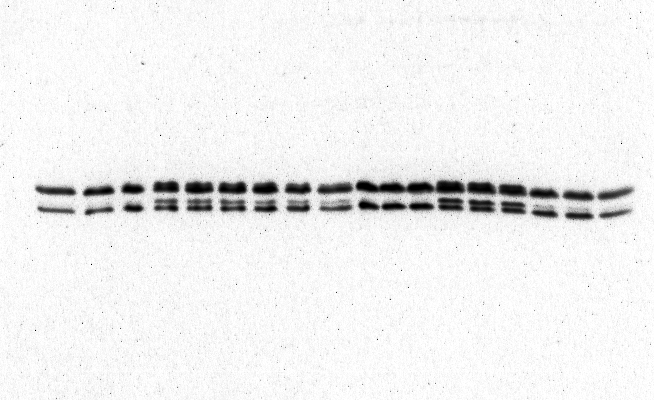

Supplement: Supplementary file 5 — Source Data for Appendix [file MSB-13-904-s013.zip › Source_Data_for_Appendix/Figure_S07/panel_B/RightPanel_ERK.jpg]

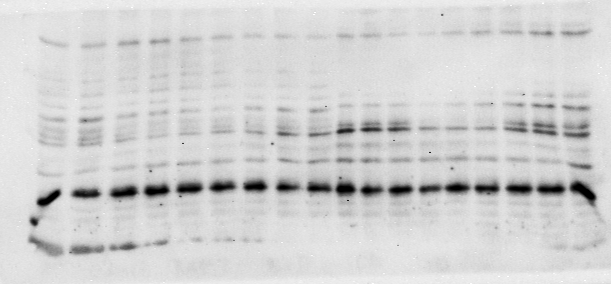

Supplement: Supplementary file 5 — Source Data for Appendix [file MSB-13-904-s013.zip › Source_Data_for_Appendix/Figure_S07/panel_B/RightPanel_Ras.tif]

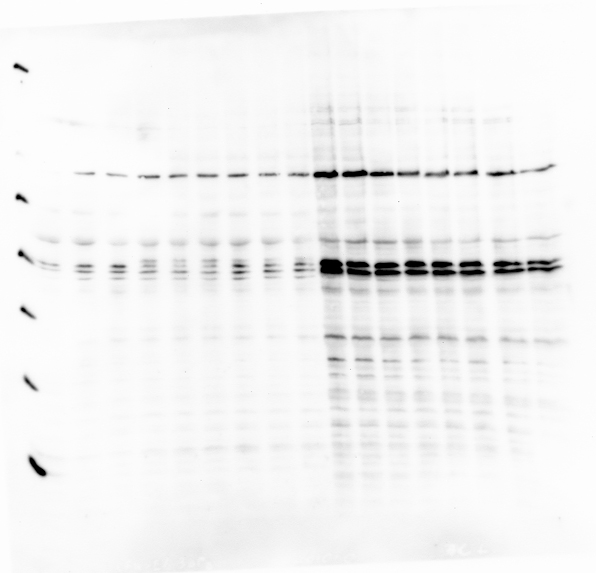

Supplement: Supplementary file 5 — Source Data for Appendix [file MSB-13-904-s013.zip › Source_Data_for_Appendix/Figure_S07/panel_B/LeftPanel_PDK1.jpg]

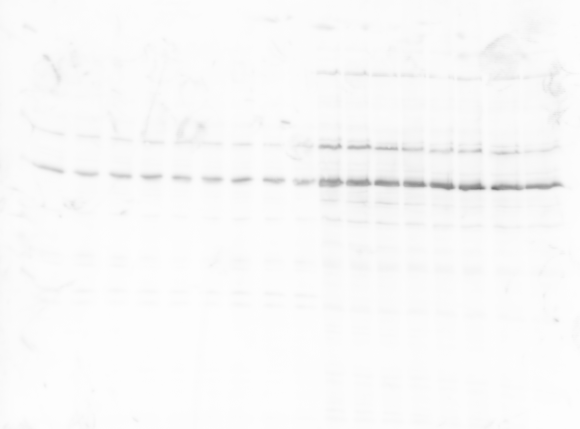

Supplement: Supplementary file 5 — Source Data for Appendix [file MSB-13-904-s013.zip › Source_Data_for_Appendix/Figure_S07/panel_B/LeftPanel_p85.tif]

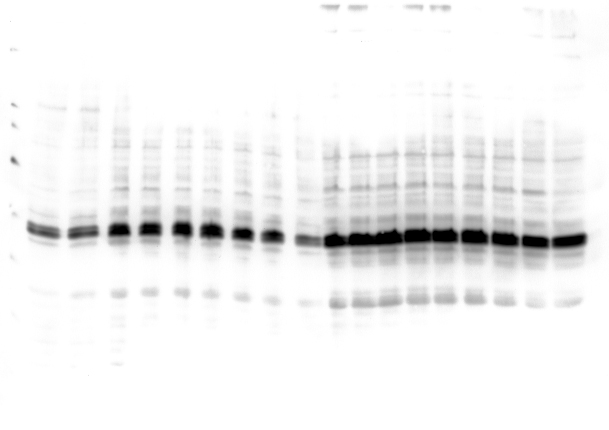

Supplement: Supplementary file 5 — Source Data for Appendix [file MSB-13-904-s013.zip › Source_Data_for_Appendix/Figure_S07/panel_B/RightPanel_AKT.jpg]

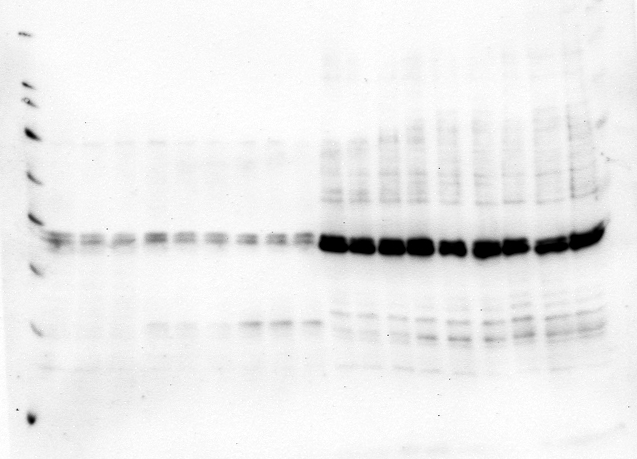

Supplement: Supplementary file 5 — Source Data for Appendix [file MSB-13-904-s013.zip › Source_Data_for_Appendix/Figure_S07/panel_B/LeftPanel_MEK.jpg]

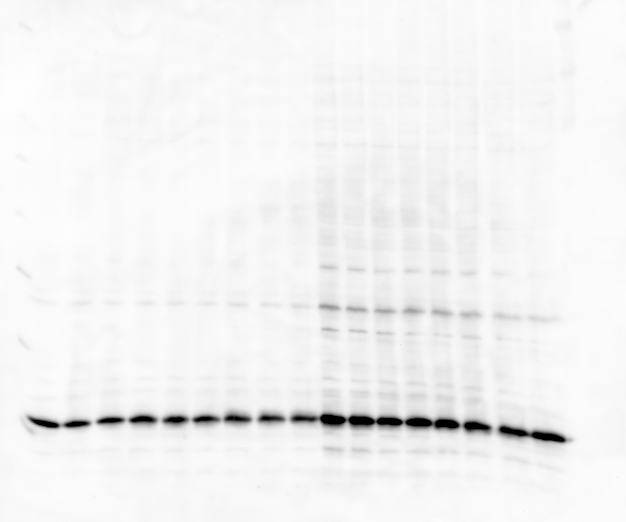

Supplement: Supplementary file 5 — Source Data for Appendix [file MSB-13-904-s013.zip › Source_Data_for_Appendix/Figure_S07/panel_B/LeftPanel_S6.tif]

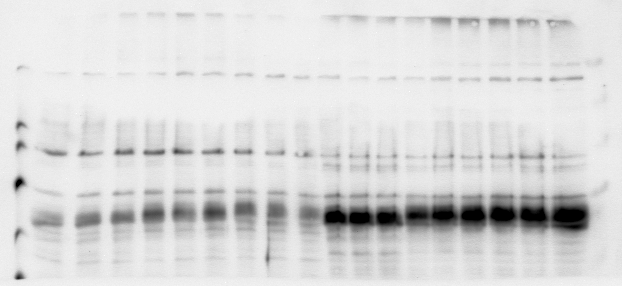

Supplement: Supplementary file 5 — Source Data for Appendix [file MSB-13-904-s013.zip › Source_Data_for_Appendix/Figure_S07/panel_B/RightPanel_Raf.tif]

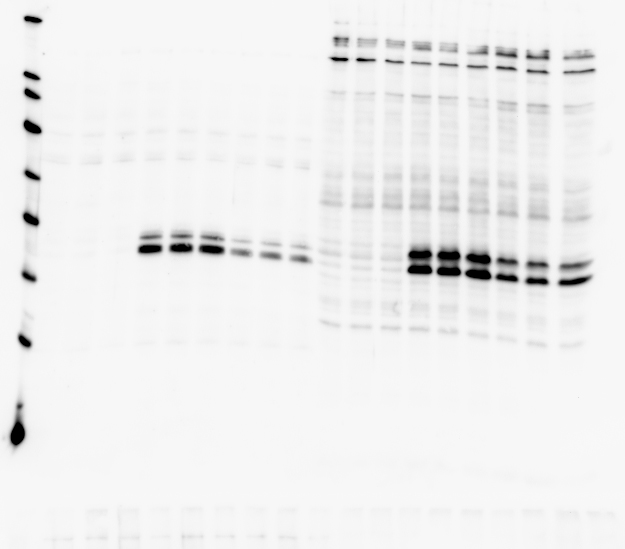

Supplement: Supplementary file 5 — Source Data for Appendix [file MSB-13-904-s013.zip › Source_Data_for_Appendix/Figure_S07/panel_B/LeftPanel_ppERK.jpg]

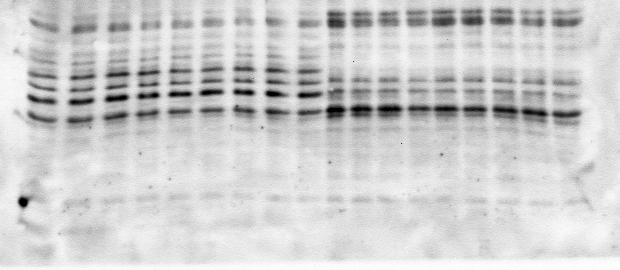

Supplement: Supplementary file 5 — Source Data for Appendix [file MSB-13-904-s013.zip › Source_Data_for_Appendix/Figure_S07/panel_B/RightPanel_S6.tif]

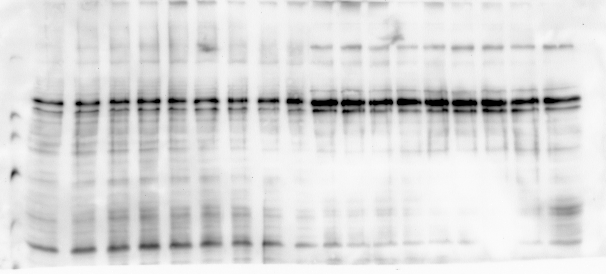

Supplement: Supplementary file 5 — Source Data for Appendix [file MSB-13-904-s013.zip › Source_Data_for_Appendix/Figure_S07/panel_B/RightPanel_SHIP1.tif]

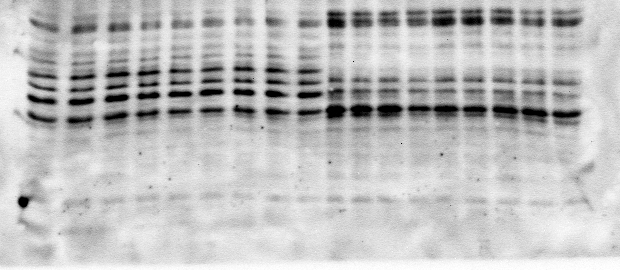

Supplement: Supplementary file 5 — Source Data for Appendix [file MSB-13-904-s013.zip › Source_Data_for_Appendix/Figure_S07/panel_B/RightPanel_S6.jpg]

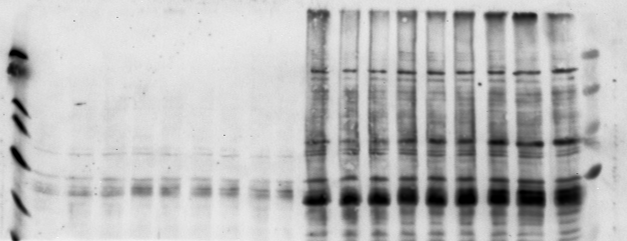

Supplement: Supplementary file 5 — Source Data for Appendix [file MSB-13-904-s013.zip › Source_Data_for_Appendix/Figure_S07/panel_B/LeftPanel_Raf.tif]

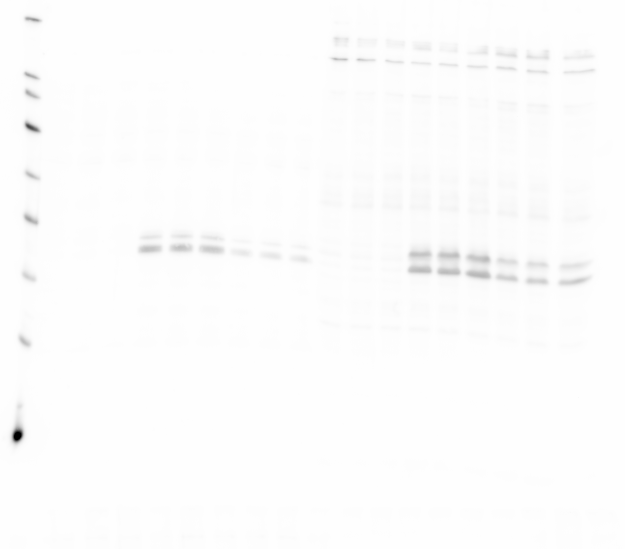

Supplement: Supplementary file 5 — Source Data for Appendix [file MSB-13-904-s013.zip › Source_Data_for_Appendix/Figure_S07/panel_B/LeftPanel_ppERK.tif]

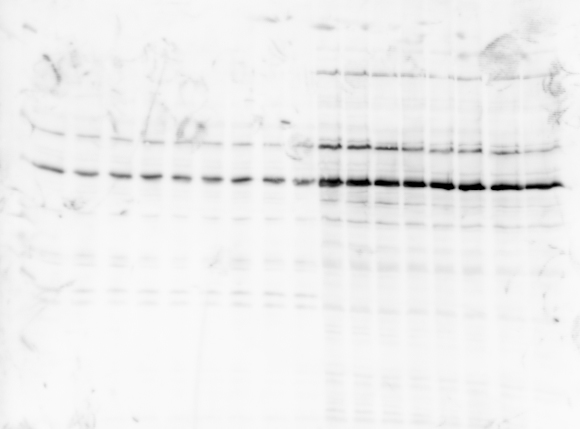

Supplement: Supplementary file 5 — Source Data for Appendix [file MSB-13-904-s013.zip › Source_Data_for_Appendix/Figure_S07/panel_B/LeftPanel_p85.jpg]

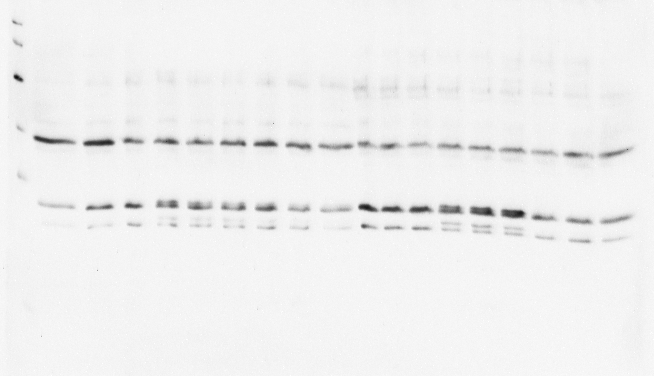

Supplement: Supplementary file 5 — Source Data for Appendix [file MSB-13-904-s013.zip › Source_Data_for_Appendix/Figure_S07/panel_B/RightPanel_PTEN.tif]

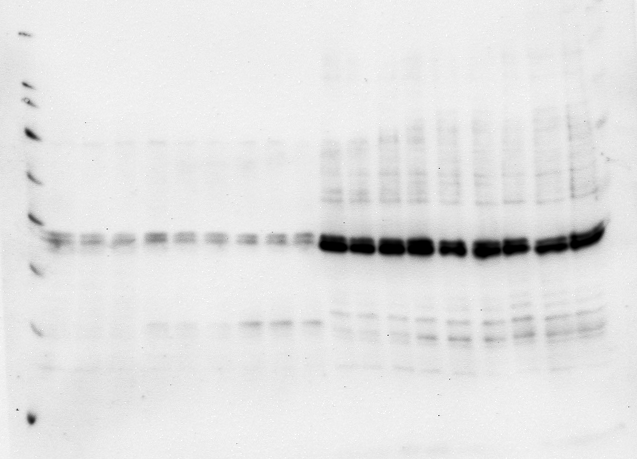

Supplement: Supplementary file 5 — Source Data for Appendix [file MSB-13-904-s013.zip › Source_Data_for_Appendix/Figure_S07/panel_B/LeftPanel_MEK.tif]

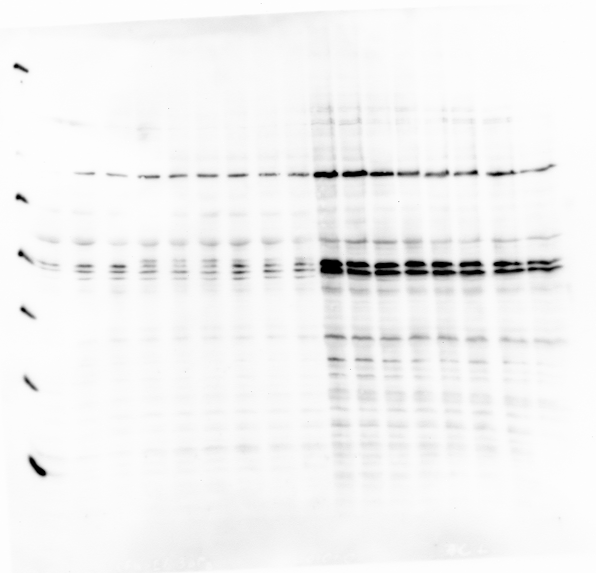

Supplement: Supplementary file 5 — Source Data for Appendix [file MSB-13-904-s013.zip › Source_Data_for_Appendix/Figure_S07/panel_B/LeftPanel_PDK1.tif]

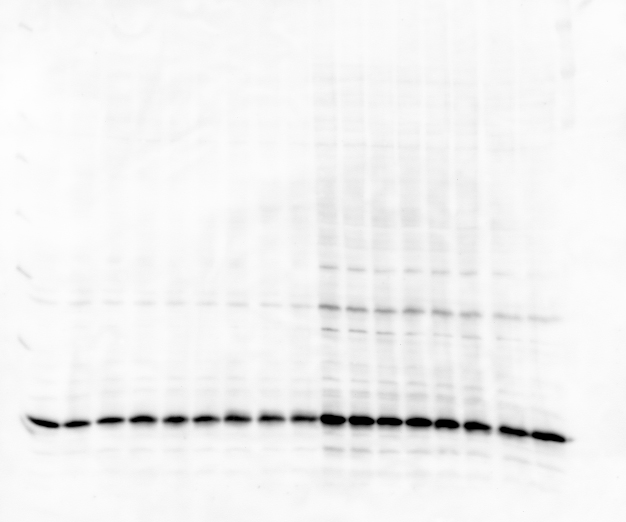

Supplement: Supplementary file 5 — Source Data for Appendix [file MSB-13-904-s013.zip › Source_Data_for_Appendix/Figure_S07/panel_B/LeftPanel_S6.jpg]

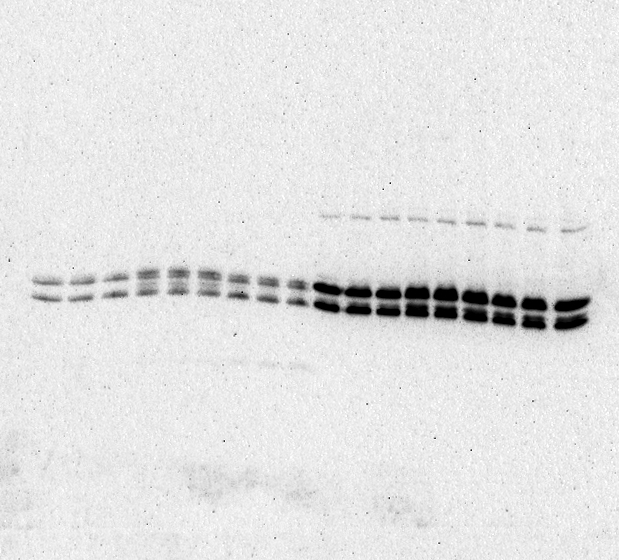

Supplement: Supplementary file 5 — Source Data for Appendix [file MSB-13-904-s013.zip › Source_Data_for_Appendix/Figure_S07/panel_B/LeftPanel_ERK.jpg]

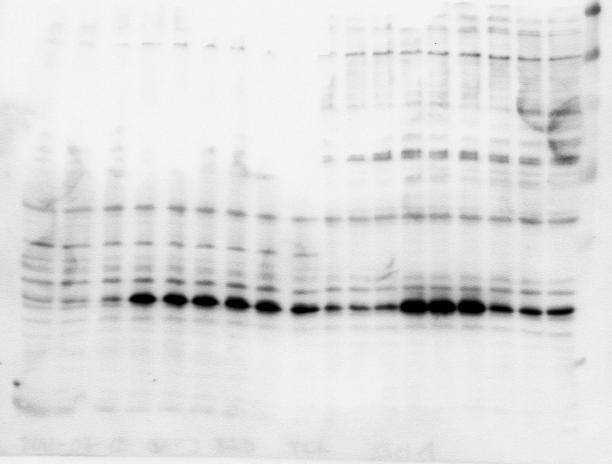

Supplement: Supplementary file 5 — Source Data for Appendix [file MSB-13-904-s013.zip › Source_Data_for_Appendix/Figure_S07/panel_B/RightPanel_pS6.tif]

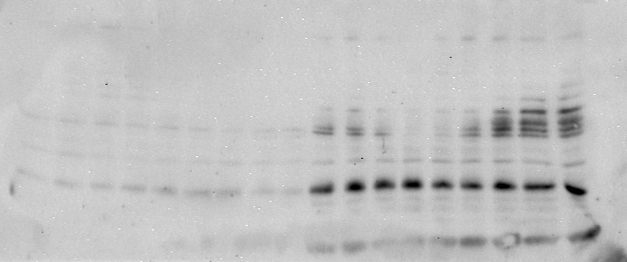

Supplement: Supplementary file 5 — Source Data for Appendix [file MSB-13-904-s013.zip › Source_Data_for_Appendix/Figure_S07/panel_B/LeftPanel_Ras.tif]

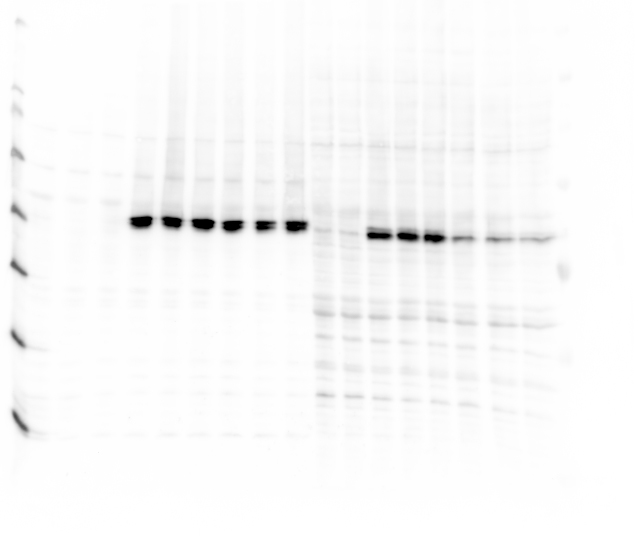

Supplement: Supplementary file 5 — Source Data for Appendix [file MSB-13-904-s013.zip › Source_Data_for_Appendix/Figure_S07/panel_B/LeftPanel_pAKT.jpg]

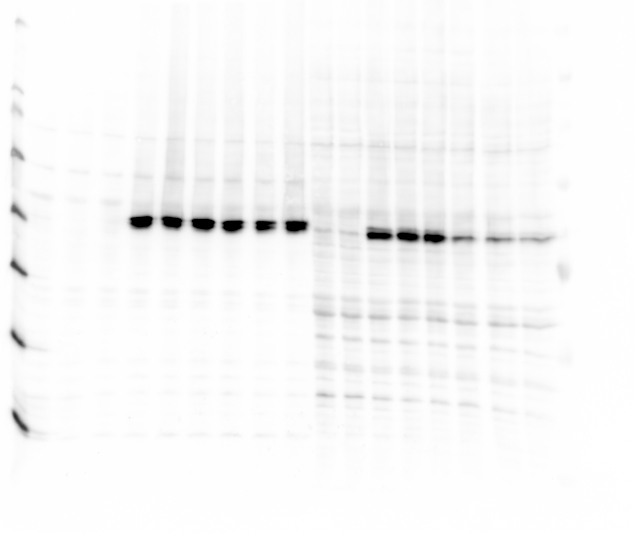

Supplement: Supplementary file 7 — Source Data for Figure 1 [file MSB-13-904-s005.zip › Source_Data_for_Figure_1/Figure01D/All_JPEG/Figure01D_pAKT.jpg]
