# Supplementary figures and images for: Protein abundance of AKT and ERK pathway components governs cell type‐specific regulation of proliferation (part 1 of 3)
Source: Mol Syst Biol. 2017 Jan 25;13(1):904. doi: 10.15252/msb.20167258 (PMC5293153; doi:10.15252/msb.20167258)

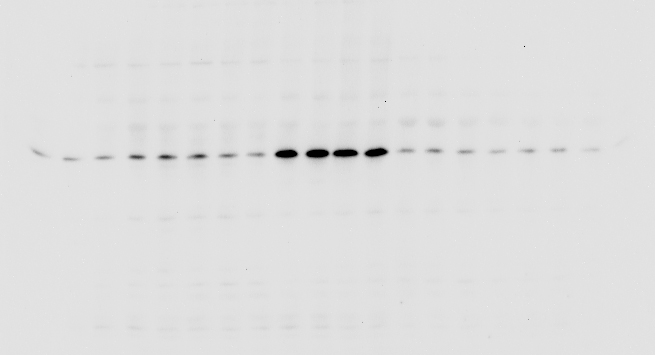

Supplement: Supplementary file 5 — Source Data for Appendix [file MSB-13-904-s013.zip › Source_Data_for_Appendix/Figure_S11/PTENoe_LeftPanel_PTEN.jpg]

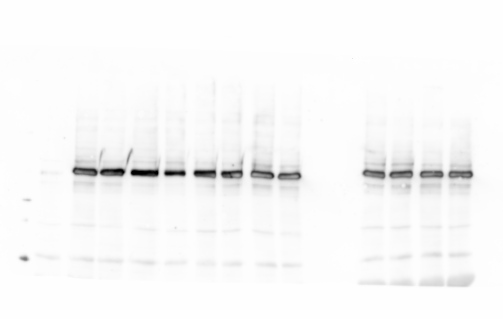

Supplement: Supplementary file 5 — Source Data for Appendix [file MSB-13-904-s013.zip › Source_Data_for_Appendix/Figure_S11/SHIP1oe_RightPanel_SHIP1.tif]

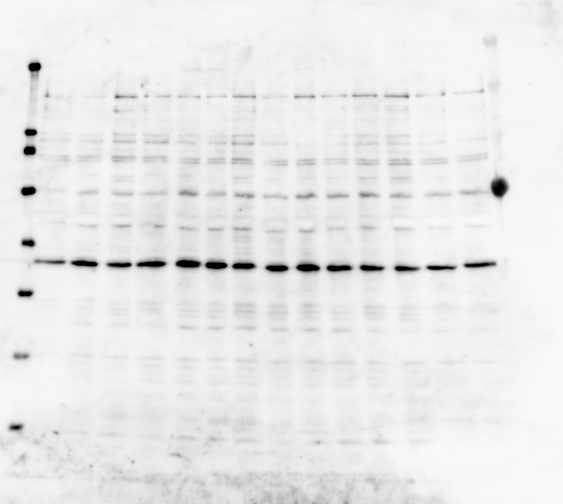

Supplement: Supplementary file 5 — Source Data for Appendix [file MSB-13-904-s013.zip › Source_Data_for_Appendix/Figure_S11/SHIP1oe_LeftPanel_PDI.jpg]

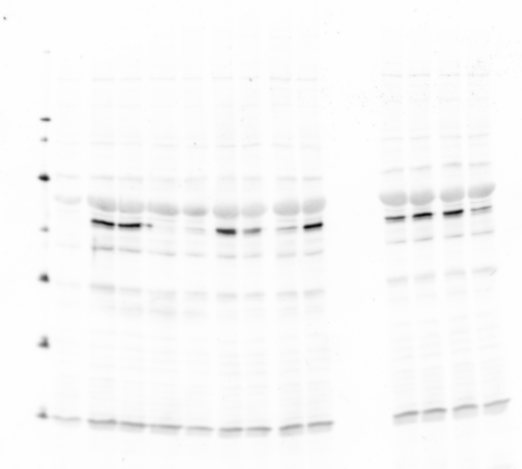

Supplement: Supplementary file 5 — Source Data for Appendix [file MSB-13-904-s013.zip › Source_Data_for_Appendix/Figure_S11/SHIP1oe_RightPanel_pAKT.tif]

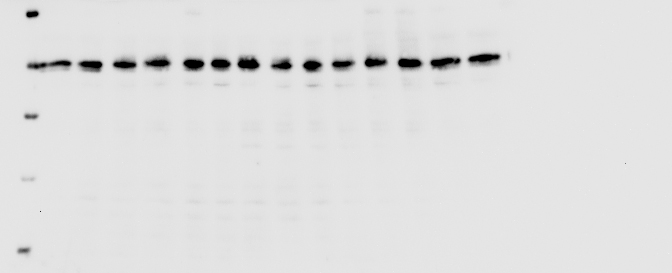

Supplement: Supplementary file 5 — Source Data for Appendix [file MSB-13-904-s013.zip › Source_Data_for_Appendix/Figure_S11/SHIP1oe_LeftPanel_AKT.jpg]

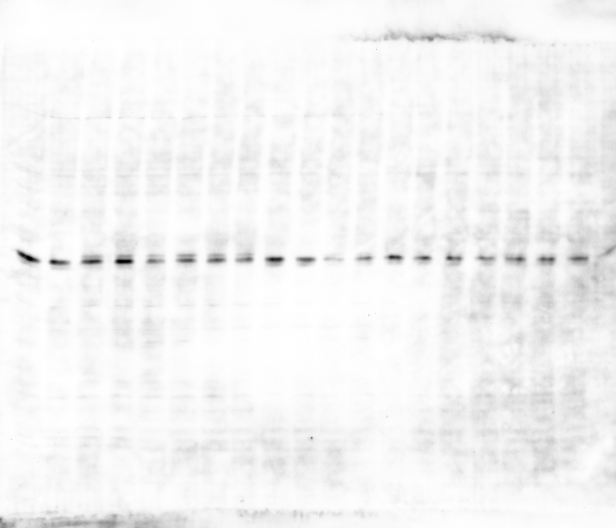

Supplement: Supplementary file 5 — Source Data for Appendix [file MSB-13-904-s013.zip › Source_Data_for_Appendix/Figure_S11/PTENoe_LeftPanel_AKT.tif]

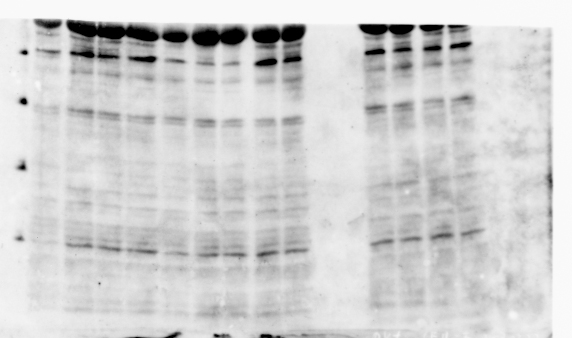

Supplement: Supplementary file 5 — Source Data for Appendix [file MSB-13-904-s013.zip › Source_Data_for_Appendix/Figure_S11/SHIP1oe_RightPanel_AKT.jpg]

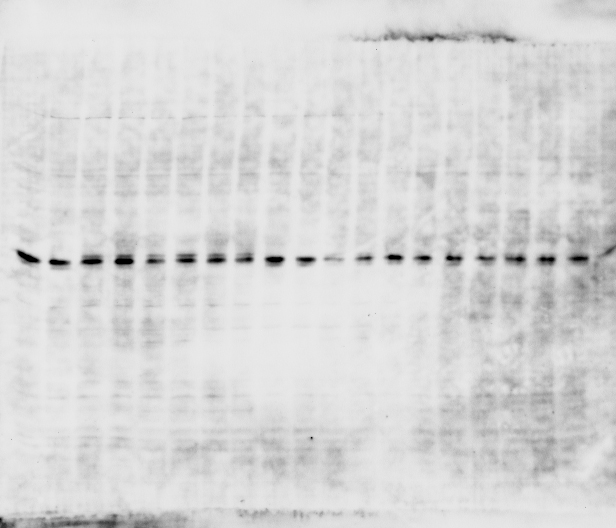

Supplement: Supplementary file 5 — Source Data for Appendix [file MSB-13-904-s013.zip › Source_Data_for_Appendix/Figure_S11/PTENoe_LeftPanel_AKT.jpg]

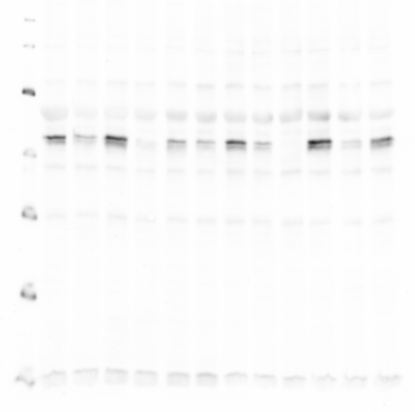

Supplement: Supplementary file 5 — Source Data for Appendix [file MSB-13-904-s013.zip › Source_Data_for_Appendix/Figure_S11/PTENoe_RightPanel_pAKT.tif]

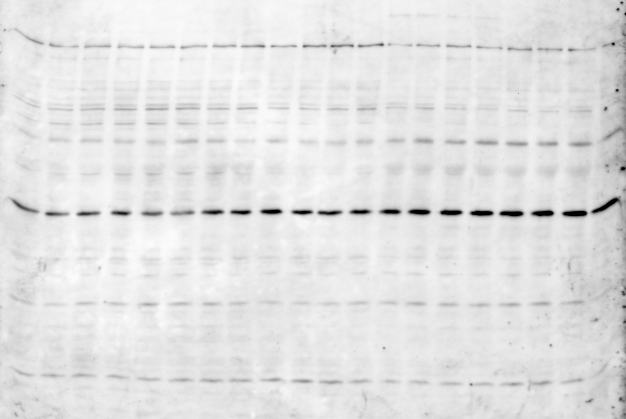

Supplement: Supplementary file 5 — Source Data for Appendix [file MSB-13-904-s013.zip › Source_Data_for_Appendix/Figure_S11/PTENoe_LeftPanel_PDI.tif]

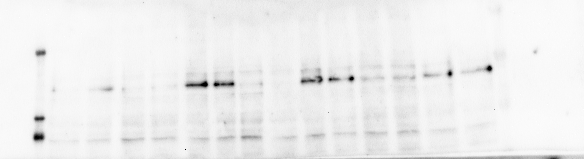

Supplement: Supplementary file 5 — Source Data for Appendix [file MSB-13-904-s013.zip › Source_Data_for_Appendix/Figure_S11/SHIP1oe_LeftPanel_SHIP1.tif]

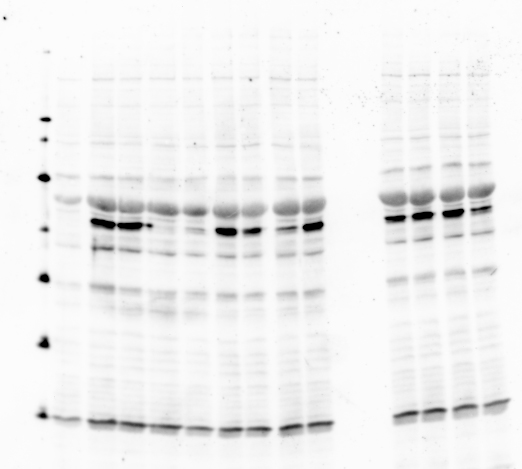

Supplement: Supplementary file 5 — Source Data for Appendix [file MSB-13-904-s013.zip › Source_Data_for_Appendix/Figure_S11/SHIP1oe_RightPanel_pAKT.jpg]

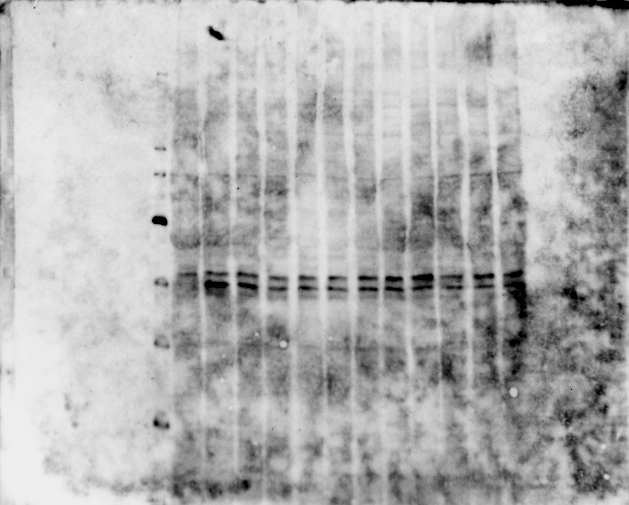

Supplement: Supplementary file 5 — Source Data for Appendix [file MSB-13-904-s013.zip › Source_Data_for_Appendix/Figure_S11/PTENoe_RightPanel_AKT.jpg]

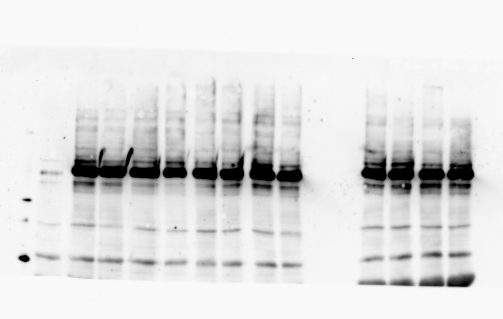

Supplement: Supplementary file 5 — Source Data for Appendix [file MSB-13-904-s013.zip › Source_Data_for_Appendix/Figure_S11/SHIP1oe_RightPanel_SHIP1.jpg]

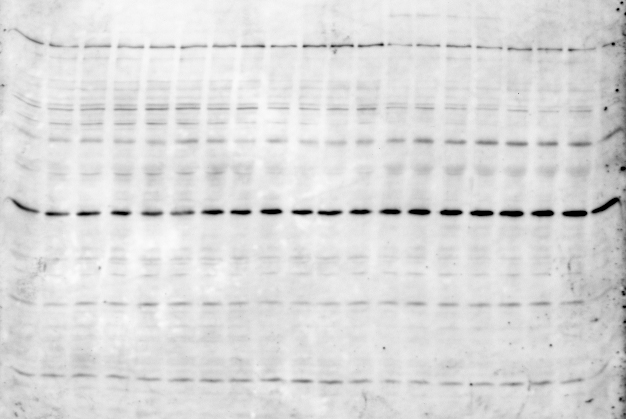

Supplement: Supplementary file 5 — Source Data for Appendix [file MSB-13-904-s013.zip › Source_Data_for_Appendix/Figure_S11/PTENoe_LeftPanel_PDI.jpg]

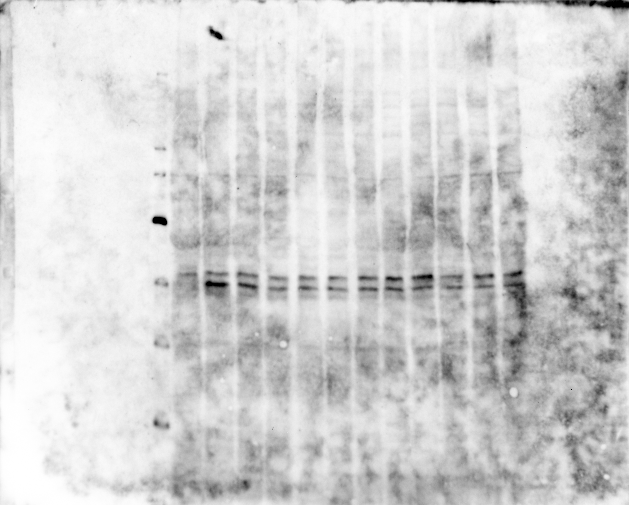

Supplement: Supplementary file 5 — Source Data for Appendix [file MSB-13-904-s013.zip › Source_Data_for_Appendix/Figure_S11/PTENoe_RightPanel_AKT.tif]

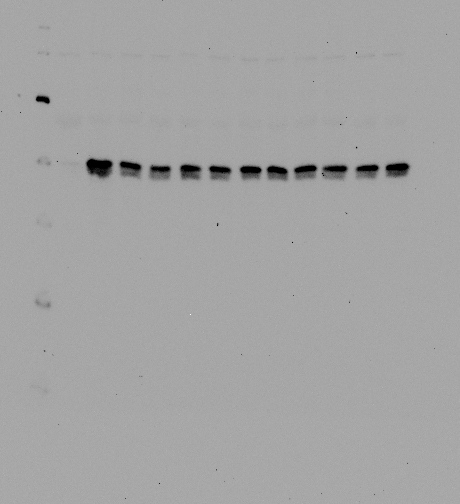

Supplement: Supplementary file 5 — Source Data for Appendix [file MSB-13-904-s013.zip › Source_Data_for_Appendix/Figure_S11/PTENoe_RightPanel_PTEN.jpg]

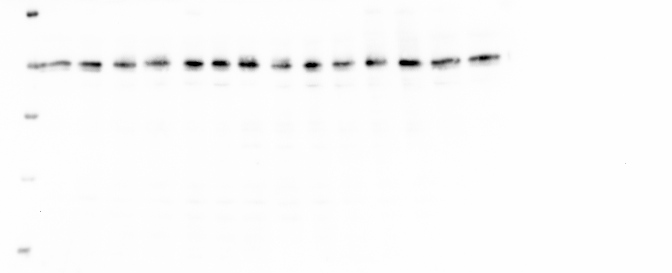

Supplement: Supplementary file 5 — Source Data for Appendix [file MSB-13-904-s013.zip › Source_Data_for_Appendix/Figure_S11/SHIP1oe_LeftPanel_AKT.tif]

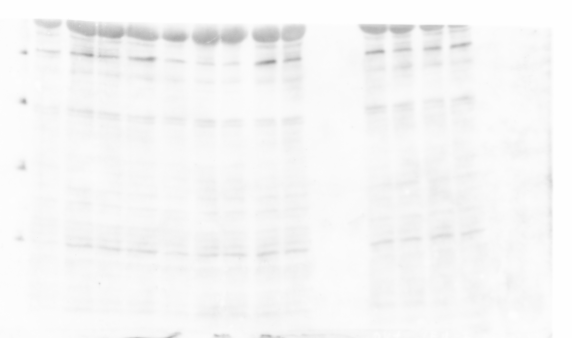

Supplement: Supplementary file 5 — Source Data for Appendix [file MSB-13-904-s013.zip › Source_Data_for_Appendix/Figure_S11/SHIP1oe_RightPanel_AKT.tif]

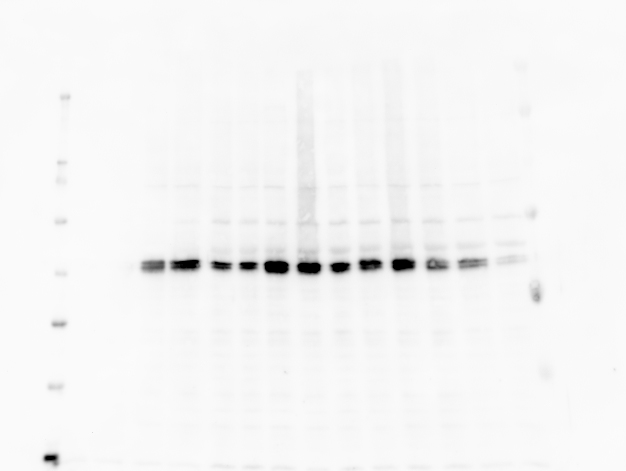

Supplement: Supplementary file 5 — Source Data for Appendix [file MSB-13-904-s013.zip › Source_Data_for_Appendix/Figure_S11/SHIP1oe_LeftPanel_pAKT.jpg]

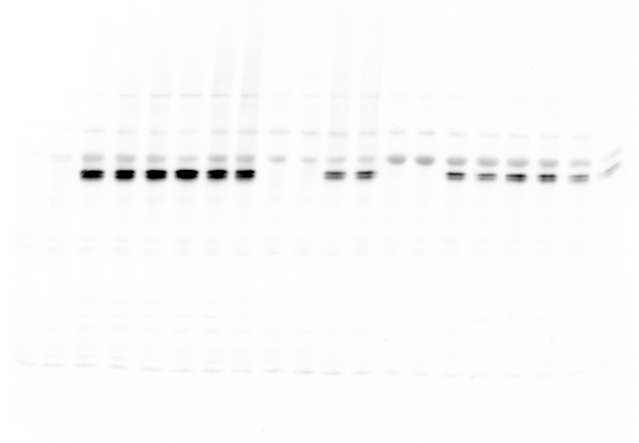

Supplement: Supplementary file 5 — Source Data for Appendix [file MSB-13-904-s013.zip › Source_Data_for_Appendix/Figure_S11/PTENoe_LeftPanel_pAKT.jpg]

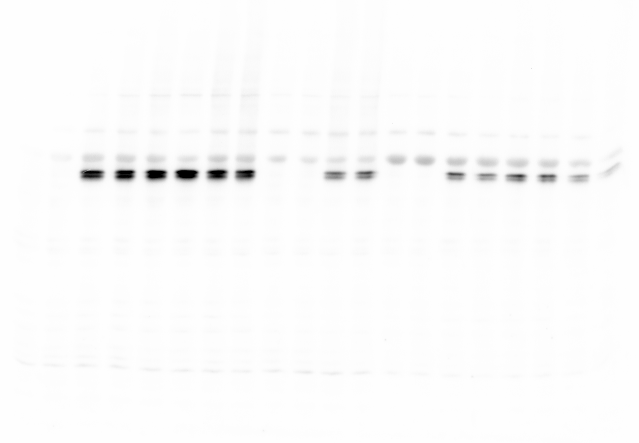

Supplement: Supplementary file 5 — Source Data for Appendix [file MSB-13-904-s013.zip › Source_Data_for_Appendix/Figure_S11/PTENoe_LeftPanel_pAKT.tif]

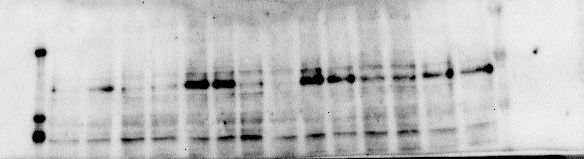

Supplement: Supplementary file 5 — Source Data for Appendix [file MSB-13-904-s013.zip › Source_Data_for_Appendix/Figure_S11/SHIP1oe_LeftPanel_SHIP1.jpg]

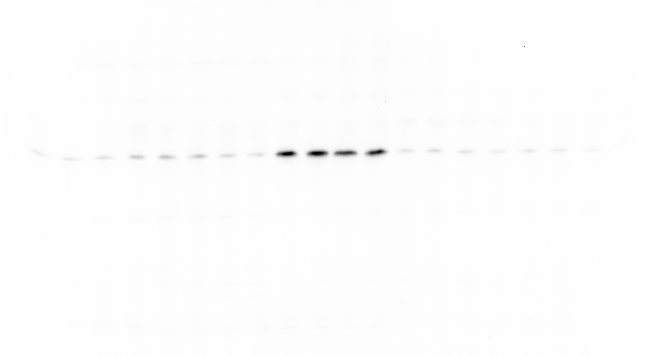

Supplement: Supplementary file 5 — Source Data for Appendix [file MSB-13-904-s013.zip › Source_Data_for_Appendix/Figure_S11/PTENoe_LeftPanel_PTEN.tif]

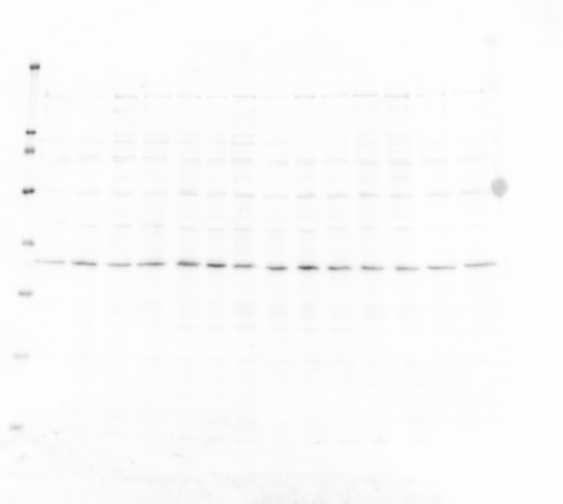

Supplement: Supplementary file 5 — Source Data for Appendix [file MSB-13-904-s013.zip › Source_Data_for_Appendix/Figure_S11/SHIP1oe_LeftPanel_PDI.tif]

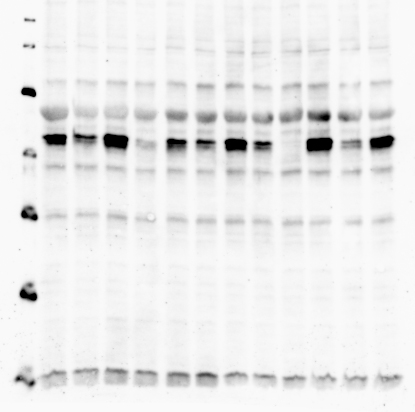

Supplement: Supplementary file 5 — Source Data for Appendix [file MSB-13-904-s013.zip › Source_Data_for_Appendix/Figure_S11/PTENoe_RightPanel_pAKT.jpg]

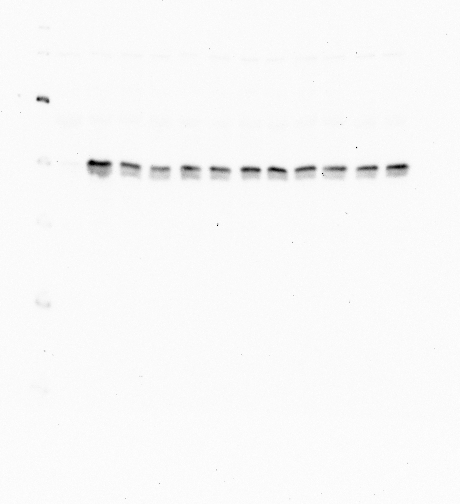

Supplement: Supplementary file 5 — Source Data for Appendix [file MSB-13-904-s013.zip › Source_Data_for_Appendix/Figure_S11/PTENoe_RightPanel_PTEN.tif]

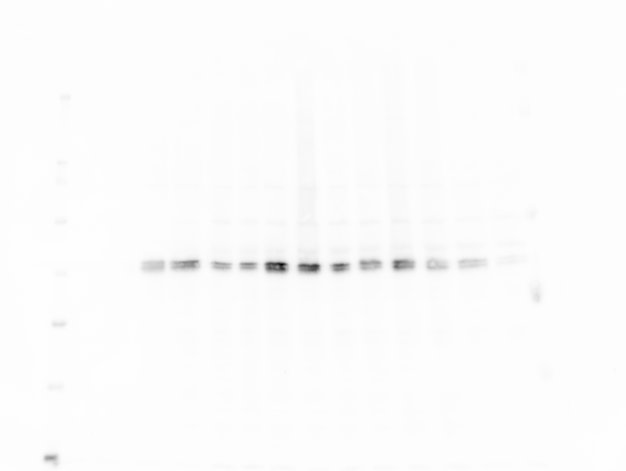

Supplement: Supplementary file 5 — Source Data for Appendix [file MSB-13-904-s013.zip › Source_Data_for_Appendix/Figure_S11/SHIP1oe_LeftPanel_pAKT.tif]

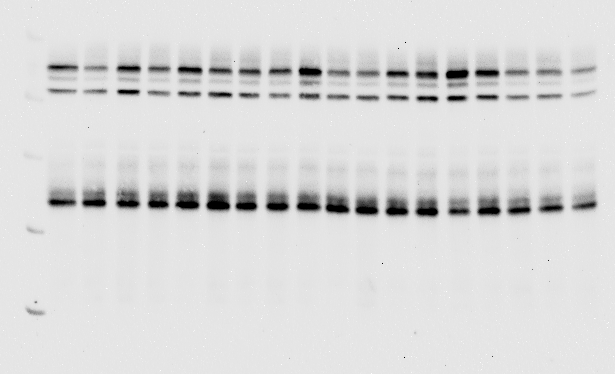

Supplement: Supplementary file 5 — Source Data for Appendix [file MSB-13-904-s013.zip › Source_Data_for_Appendix/Figure_S09/UpperPanel_BaF3_EpoR_GST-EpoR.tif]

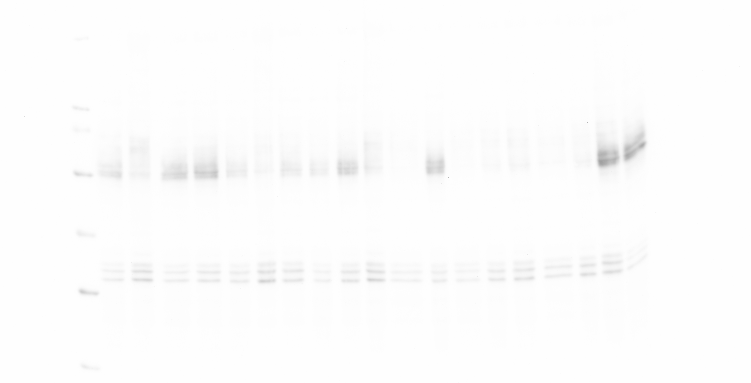

Supplement: Supplementary file 5 — Source Data for Appendix [file MSB-13-904-s013.zip › Source_Data_for_Appendix/Figure_S09/MiddlePanel_BaF3_pEpoR.tif]

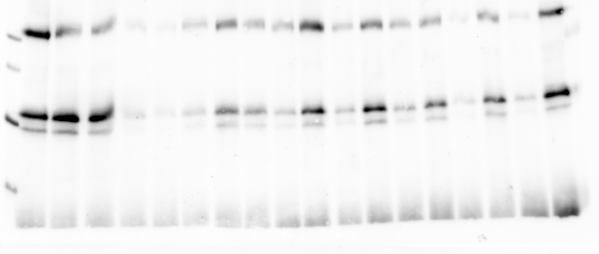

Supplement: Supplementary file 5 — Source Data for Appendix [file MSB-13-904-s013.zip › Source_Data_for_Appendix/Figure_S09/LowerPanel_CFUE_pEpoR.tif]

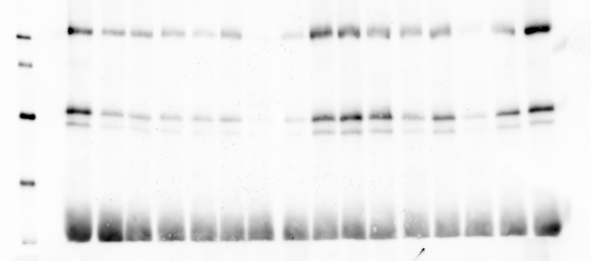

Supplement: Supplementary file 5 — Source Data for Appendix [file MSB-13-904-s013.zip › Source_Data_for_Appendix/Figure_S09/MiddlePanel_CFUE_pEpoR.tif]

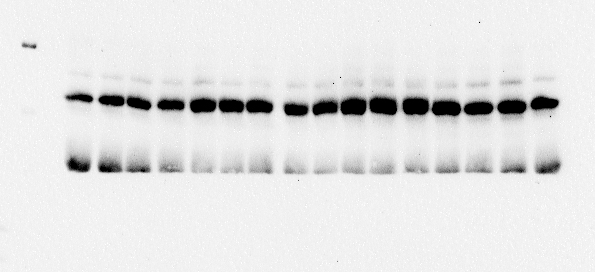

Supplement: Supplementary file 5 — Source Data for Appendix [file MSB-13-904-s013.zip › Source_Data_for_Appendix/Figure_S09/MiddlePanel_CFUE_EpoR_GST-EpoR.jpg]

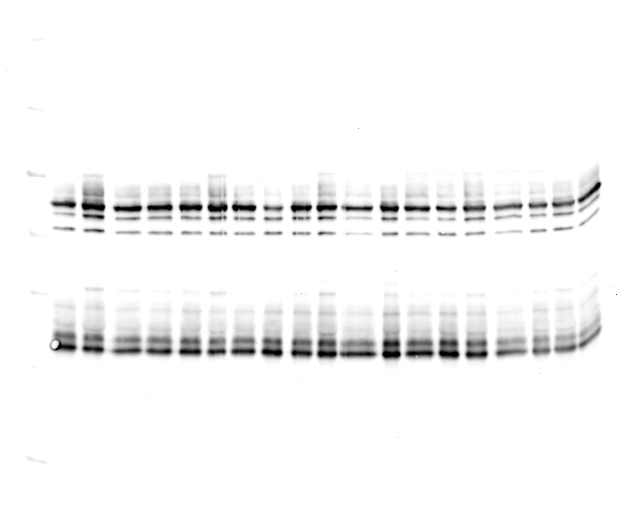

Supplement: Supplementary file 5 — Source Data for Appendix [file MSB-13-904-s013.zip › Source_Data_for_Appendix/Figure_S09/MiddlePanel_BaF3_EpoR_GST-EpoR.jpg]

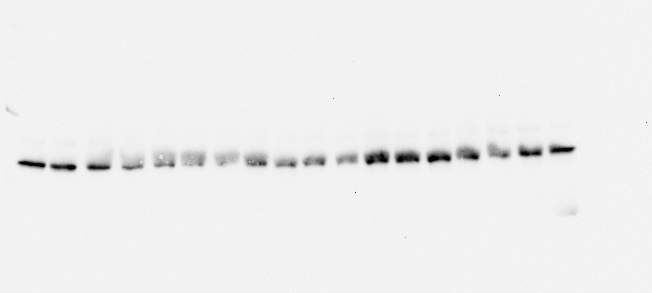

Supplement: Supplementary file 5 — Source Data for Appendix [file MSB-13-904-s013.zip › Source_Data_for_Appendix/Figure_S09/LowerPanel_CFUE_EpoR_GST-EpoR.tif]

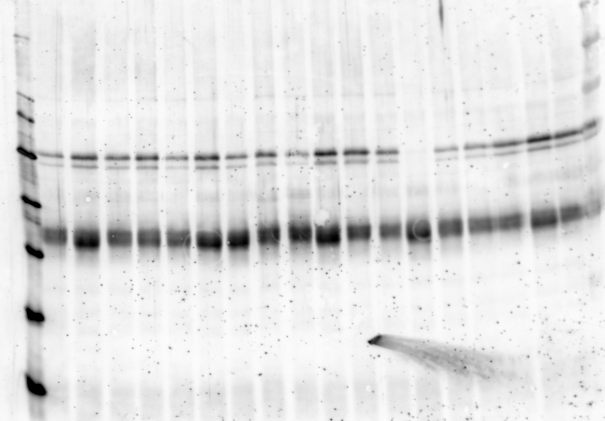

Supplement: Supplementary file 5 — Source Data for Appendix [file MSB-13-904-s013.zip › Source_Data_for_Appendix/Figure_S09/UpperPanel_CFUE_pEpoR.tif]

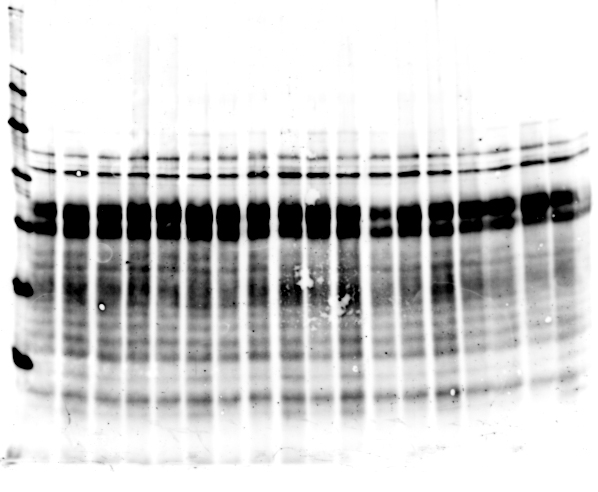

Supplement: Supplementary file 5 — Source Data for Appendix [file MSB-13-904-s013.zip › Source_Data_for_Appendix/Figure_S09/UpperPanel_CFUE_EpoR_GST-EpoR.jpg]

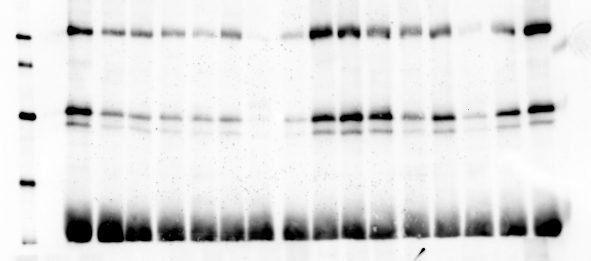

Supplement: Supplementary file 5 — Source Data for Appendix [file MSB-13-904-s013.zip › Source_Data_for_Appendix/Figure_S09/MiddlePanel_CFUE_pEpoR.jpg]

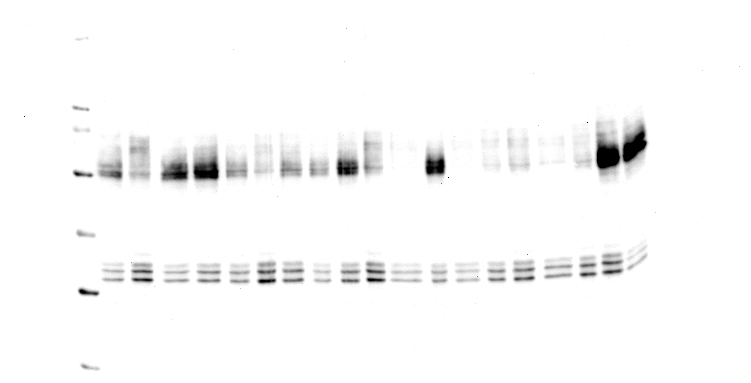

Supplement: Supplementary file 5 — Source Data for Appendix [file MSB-13-904-s013.zip › Source_Data_for_Appendix/Figure_S09/MiddlePanel_BaF3_pEpoR.jpg]

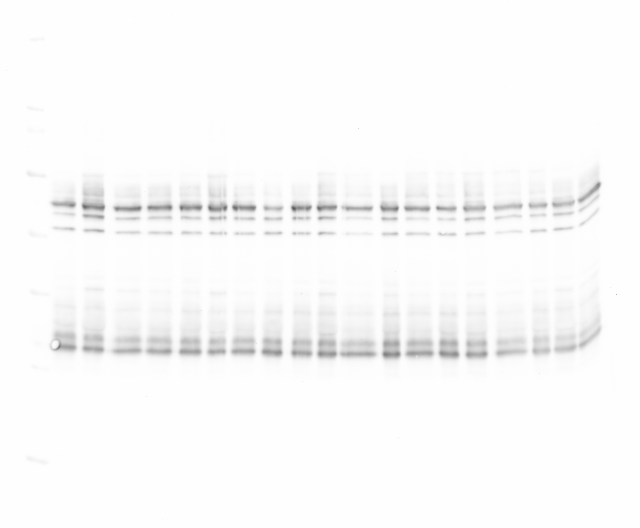

Supplement: Supplementary file 5 — Source Data for Appendix [file MSB-13-904-s013.zip › Source_Data_for_Appendix/Figure_S09/MiddlePanel_BaF3_EpoR_GST-EpoR.tif]

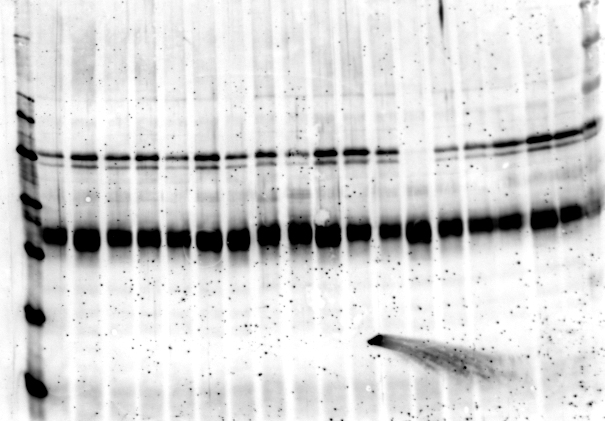

Supplement: Supplementary file 5 — Source Data for Appendix [file MSB-13-904-s013.zip › Source_Data_for_Appendix/Figure_S09/UpperPanel_CFUE_pEpoR.jpg]

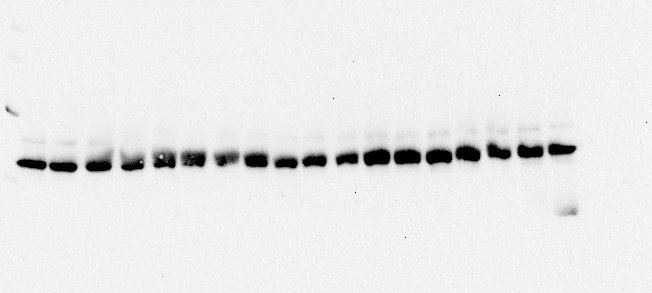

Supplement: Supplementary file 5 — Source Data for Appendix [file MSB-13-904-s013.zip › Source_Data_for_Appendix/Figure_S09/LowerPanel_CFUE_EpoR_GST-EpoR.jpg]

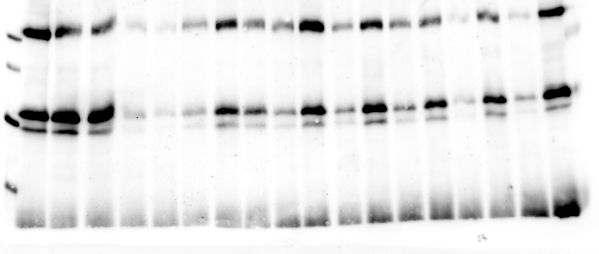

Supplement: Supplementary file 5 — Source Data for Appendix [file MSB-13-904-s013.zip › Source_Data_for_Appendix/Figure_S09/LowerPanel_CFUE_pEpoR.jpg]

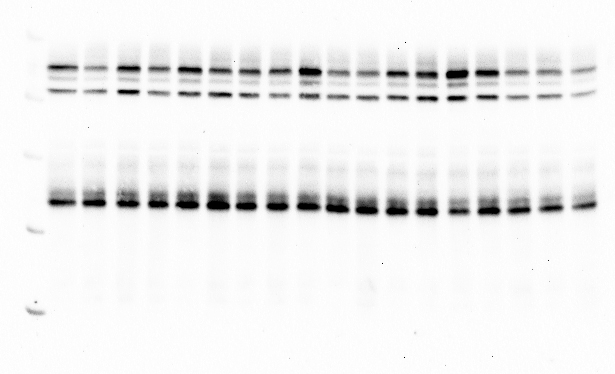

Supplement: Supplementary file 5 — Source Data for Appendix [file MSB-13-904-s013.zip › Source_Data_for_Appendix/Figure_S09/UpperPanel_BaF3_EpoR_GST-EpoR.jpg]

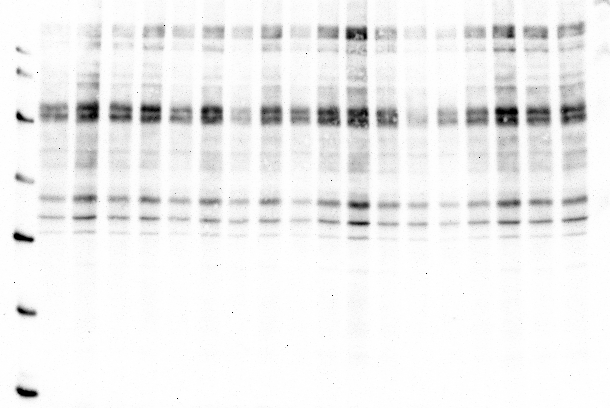

Supplement: Supplementary file 5 — Source Data for Appendix [file MSB-13-904-s013.zip › Source_Data_for_Appendix/Figure_S09/UpperPanel_BaF3_pEpoR.tif]

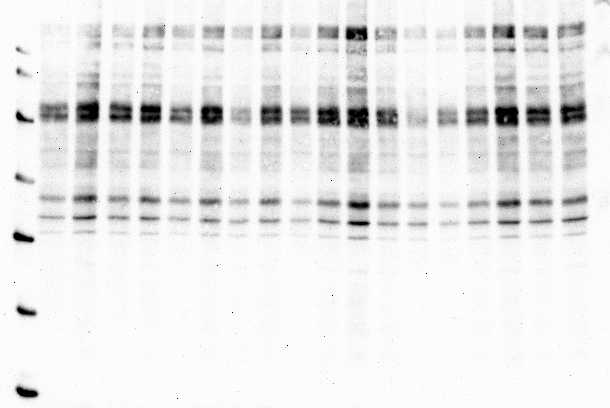

Supplement: Supplementary file 5 — Source Data for Appendix [file MSB-13-904-s013.zip › Source_Data_for_Appendix/Figure_S09/UpperPanel_BaF3_pEpoR.jpg]

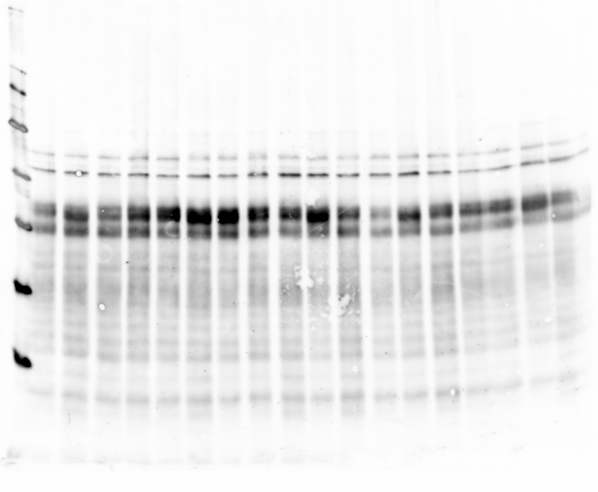

Supplement: Supplementary file 5 — Source Data for Appendix [file MSB-13-904-s013.zip › Source_Data_for_Appendix/Figure_S09/UpperPanel_CFUE_EpoR_GST-EpoR.tif]

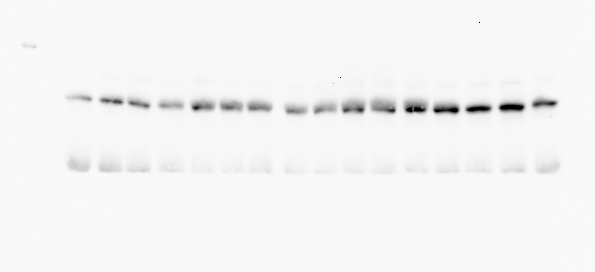

Supplement: Supplementary file 5 — Source Data for Appendix [file MSB-13-904-s013.zip › Source_Data_for_Appendix/Figure_S09/MiddlePanel_CFUE_EpoR_GST-EpoR.tif]

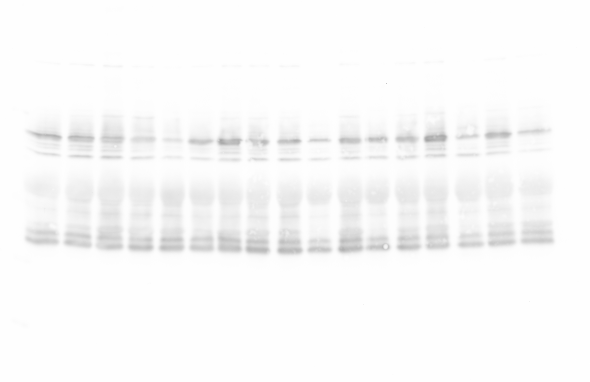

Supplement: Supplementary file 5 — Source Data for Appendix [file MSB-13-904-s013.zip › Source_Data_for_Appendix/Figure_S13/panel_A/LowerPanel_EpoR_GST-EpoR.tif]

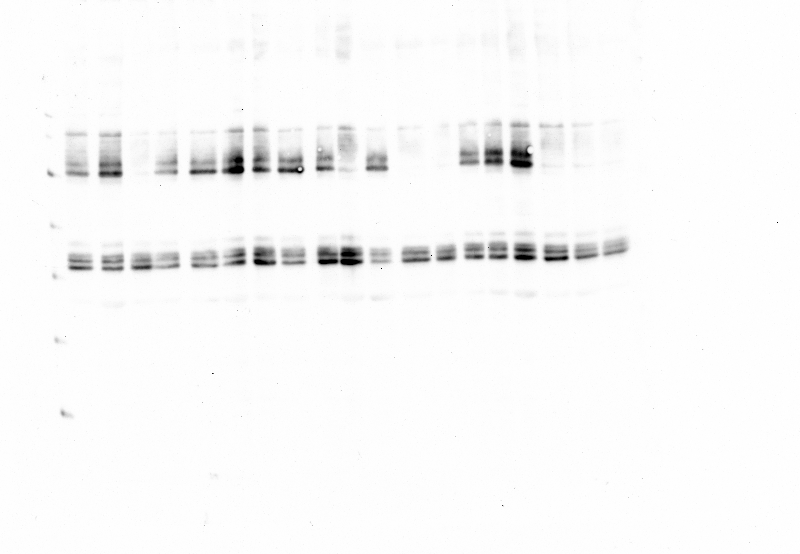

Supplement: Supplementary file 5 — Source Data for Appendix [file MSB-13-904-s013.zip › Source_Data_for_Appendix/Figure_S13/panel_A/UpperPanel_pEpoR.tif]

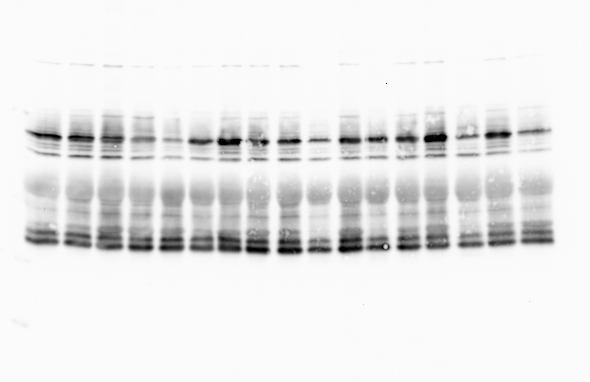

Supplement: Supplementary file 5 — Source Data for Appendix [file MSB-13-904-s013.zip › Source_Data_for_Appendix/Figure_S13/panel_A/LowerPanel_EpoR_GST-EpoR.jpg]

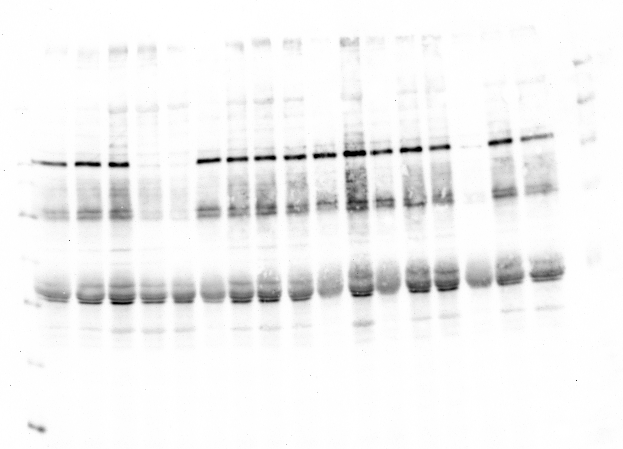

Supplement: Supplementary file 5 — Source Data for Appendix [file MSB-13-904-s013.zip › Source_Data_for_Appendix/Figure_S13/panel_A/LowerPanel_pEpoR.tif]

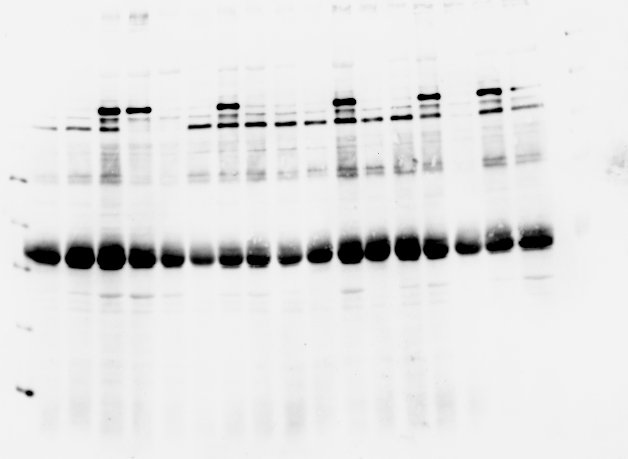

Supplement: Supplementary file 5 — Source Data for Appendix [file MSB-13-904-s013.zip › Source_Data_for_Appendix/Figure_S13/panel_A/LowerPanel_SHIP1.jpg]

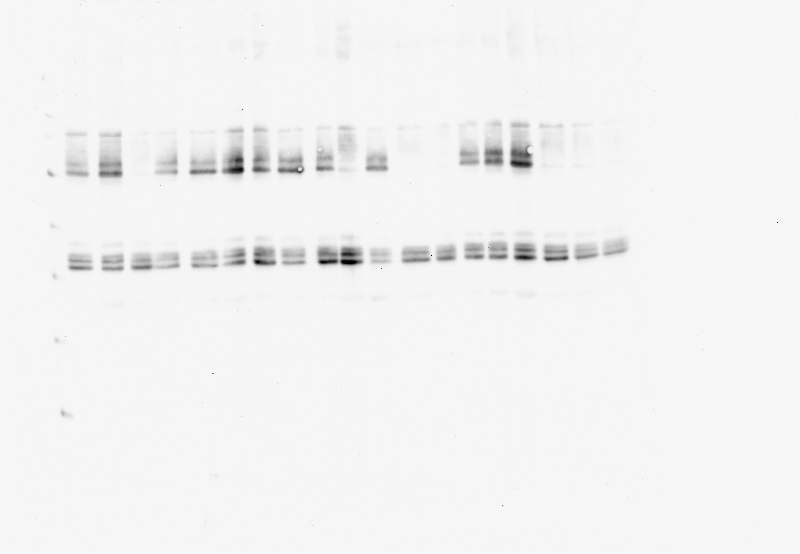

Supplement: Supplementary file 5 — Source Data for Appendix [file MSB-13-904-s013.zip › Source_Data_for_Appendix/Figure_S13/panel_A/UpperPanel_pEpoR.jpg]

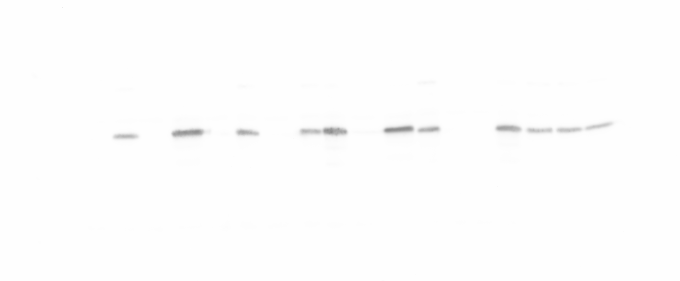

Supplement: Supplementary file 5 — Source Data for Appendix [file MSB-13-904-s013.zip › Source_Data_for_Appendix/Figure_S13/panel_A/UpperPanel_PTEN.tif]

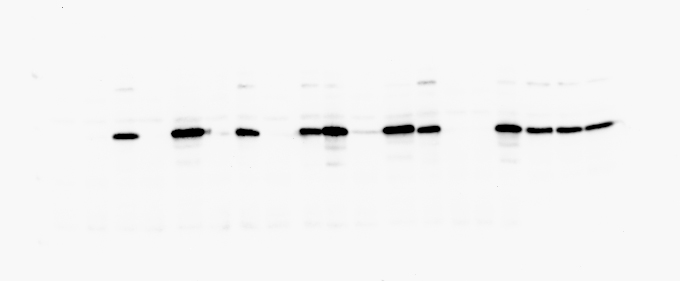

Supplement: Supplementary file 5 — Source Data for Appendix [file MSB-13-904-s013.zip › Source_Data_for_Appendix/Figure_S13/panel_A/UpperPanel_PTEN.jpg]

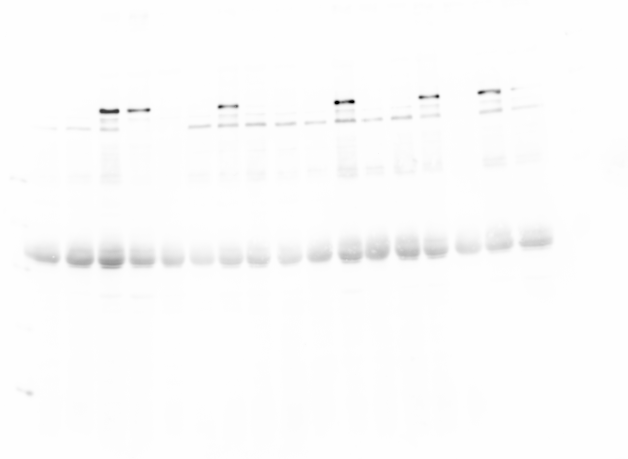

Supplement: Supplementary file 5 — Source Data for Appendix [file MSB-13-904-s013.zip › Source_Data_for_Appendix/Figure_S13/panel_A/LowerPanel_SHIP1.tif]

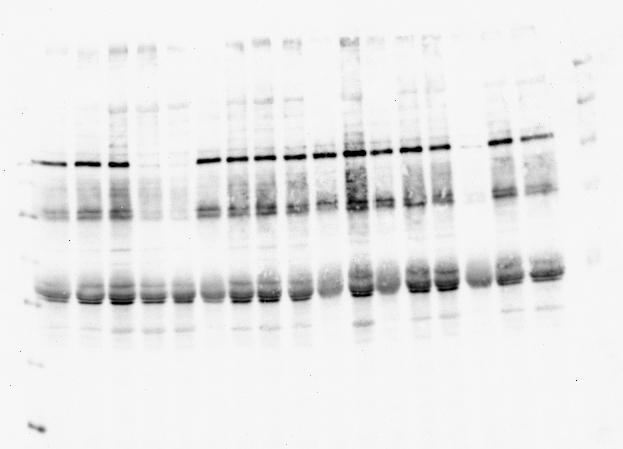

Supplement: Supplementary file 5 — Source Data for Appendix [file MSB-13-904-s013.zip › Source_Data_for_Appendix/Figure_S13/panel_A/LowerPanel_pEpoR.jpg]

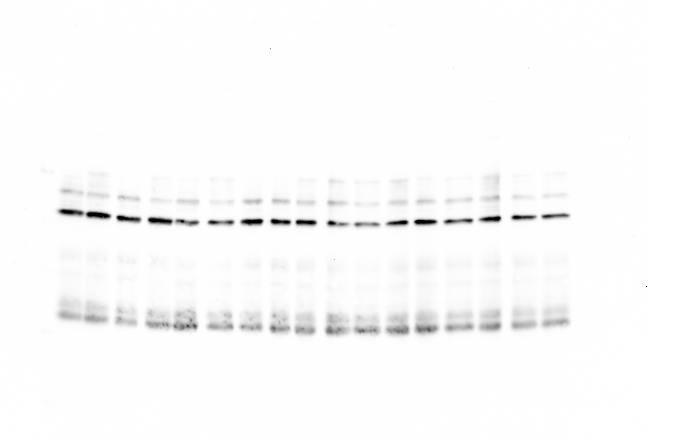

Supplement: Supplementary file 5 — Source Data for Appendix [file MSB-13-904-s013.zip › Source_Data_for_Appendix/Figure_S13/panel_A/UpperPanel_EpoR_GST-EpoR.tif]

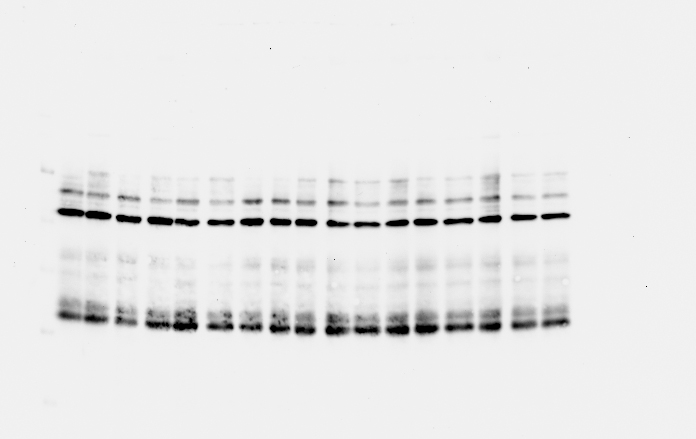

Supplement: Supplementary file 5 — Source Data for Appendix [file MSB-13-904-s013.zip › Source_Data_for_Appendix/Figure_S13/panel_A/UpperPanel_EpoR_GST-EpoR.jpg]

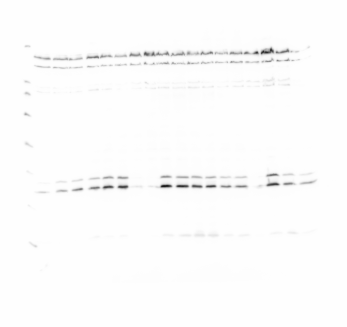

Supplement: Supplementary file 5 — Source Data for Appendix [file MSB-13-904-s013.zip › Source_Data_for_Appendix/Figure_S13/panel_B/LowerPanel_ppERK.tif]

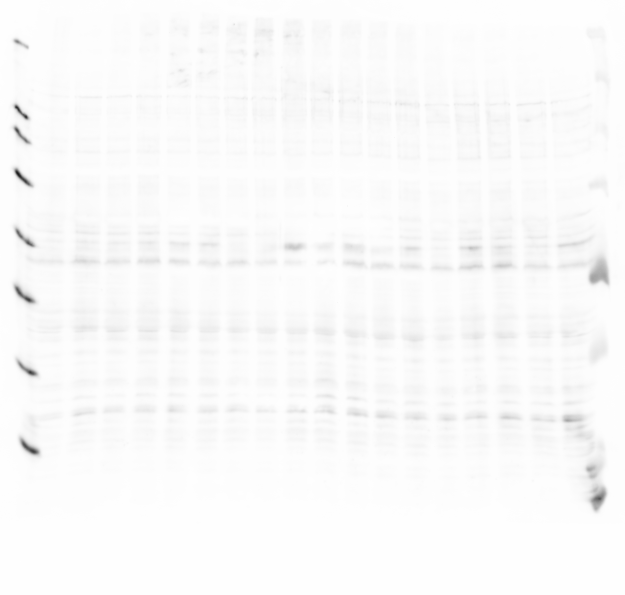

Supplement: Supplementary file 5 — Source Data for Appendix [file MSB-13-904-s013.zip › Source_Data_for_Appendix/Figure_S13/panel_B/UpperPanel_pAKT.tif]

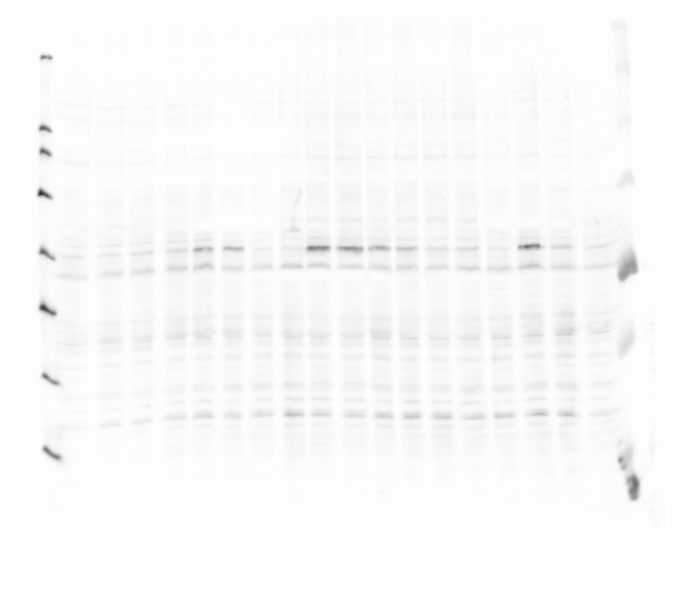

Supplement: Supplementary file 5 — Source Data for Appendix [file MSB-13-904-s013.zip › Source_Data_for_Appendix/Figure_S13/panel_B/LowerPanel_pAKT.tif]

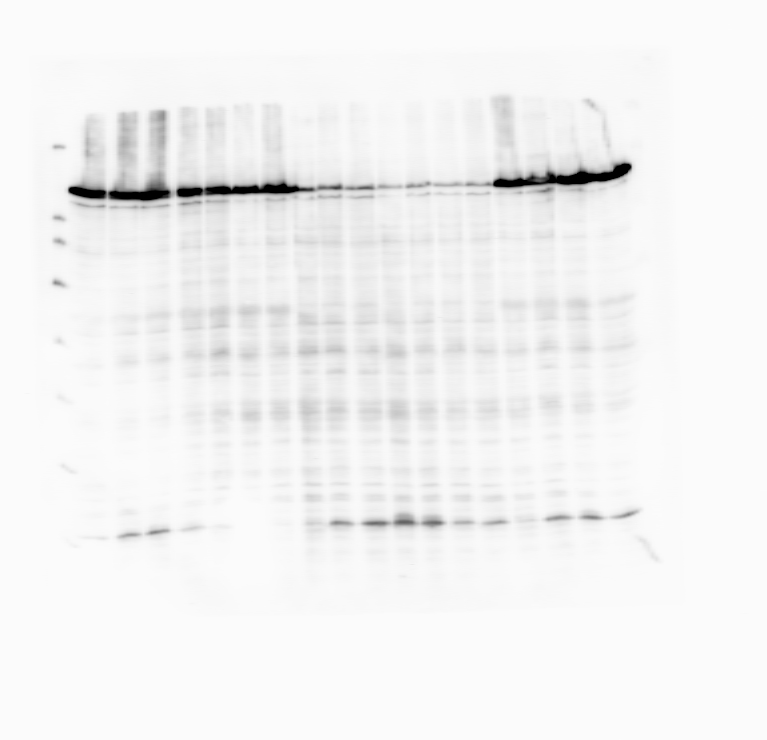

Supplement: Supplementary file 5 — Source Data for Appendix [file MSB-13-904-s013.zip › Source_Data_for_Appendix/Figure_S13/panel_B/LowerPanel_SHIP1.jpg]

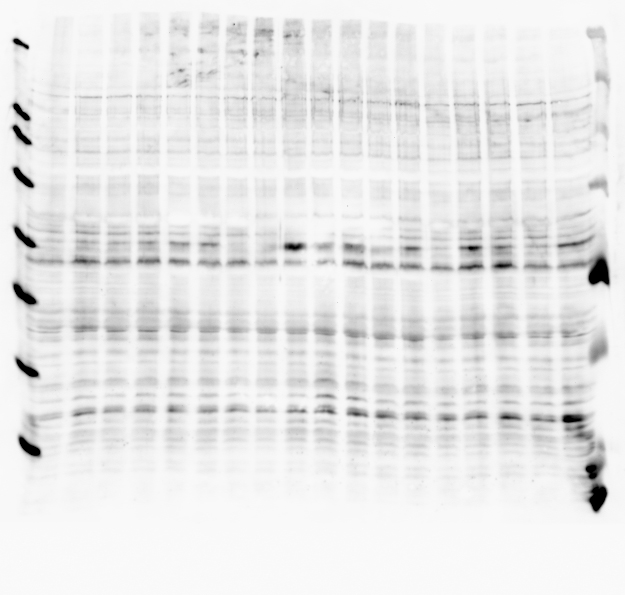

Supplement: Supplementary file 5 — Source Data for Appendix [file MSB-13-904-s013.zip › Source_Data_for_Appendix/Figure_S13/panel_B/UpperPanel_pAKT.jpg]

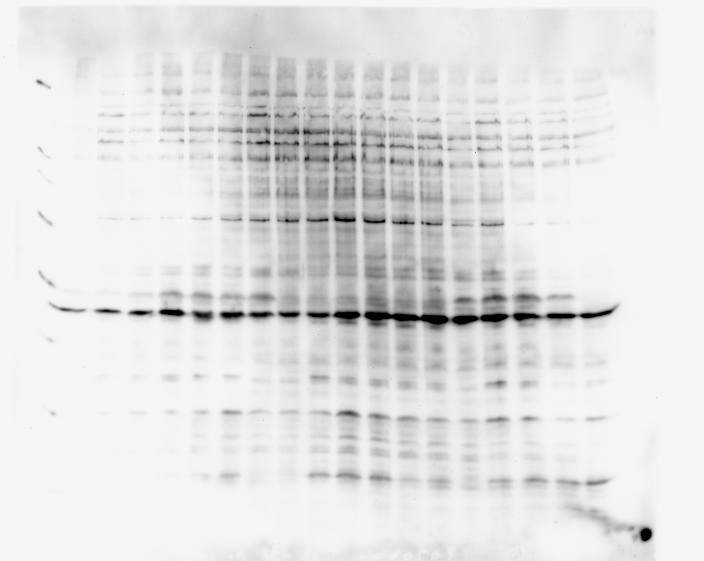

Supplement: Supplementary file 5 — Source Data for Appendix [file MSB-13-904-s013.zip › Source_Data_for_Appendix/Figure_S13/panel_B/UpperPanel_PDI.jpg]

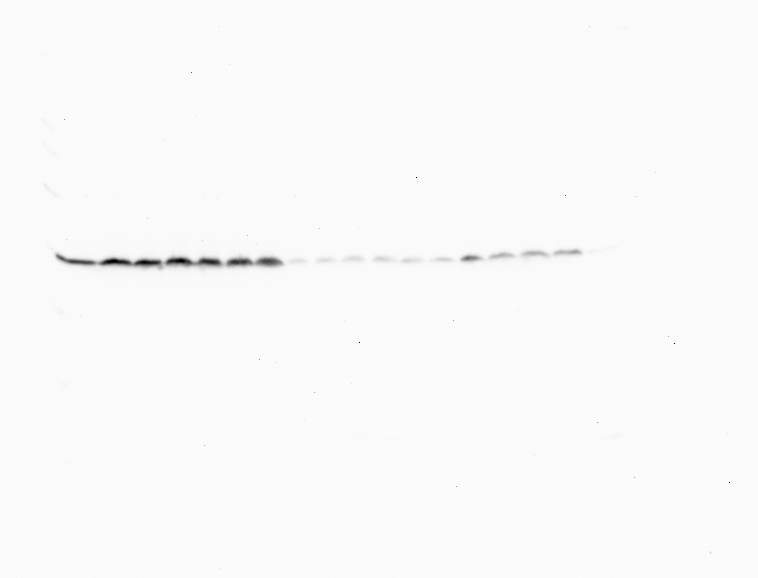

Supplement: Supplementary file 5 — Source Data for Appendix [file MSB-13-904-s013.zip › Source_Data_for_Appendix/Figure_S13/panel_B/UpperPanel_PTEN.tif]

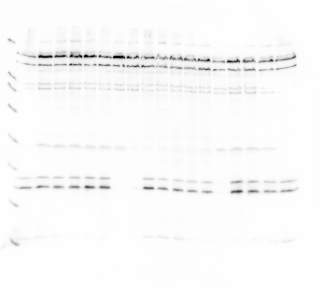

Supplement: Supplementary file 5 — Source Data for Appendix [file MSB-13-904-s013.zip › Source_Data_for_Appendix/Figure_S13/panel_B/UpperPanel_ppERK.tif]

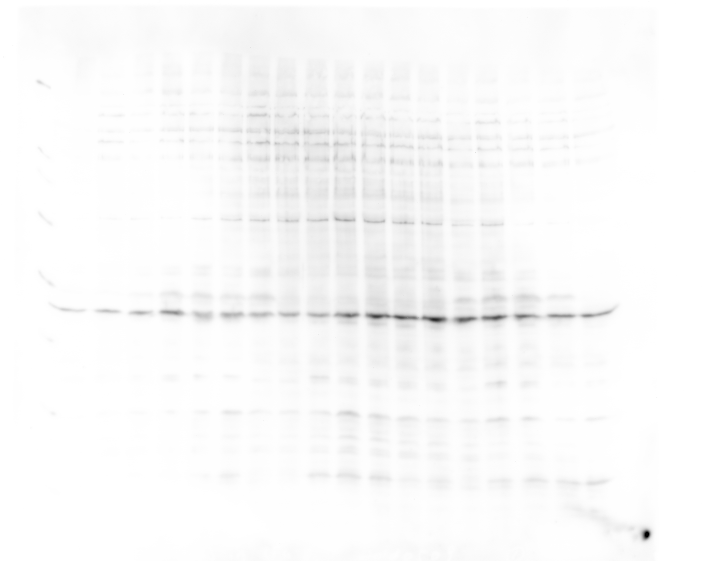

Supplement: Supplementary file 5 — Source Data for Appendix [file MSB-13-904-s013.zip › Source_Data_for_Appendix/Figure_S13/panel_B/UpperPanel_PDI.tif]

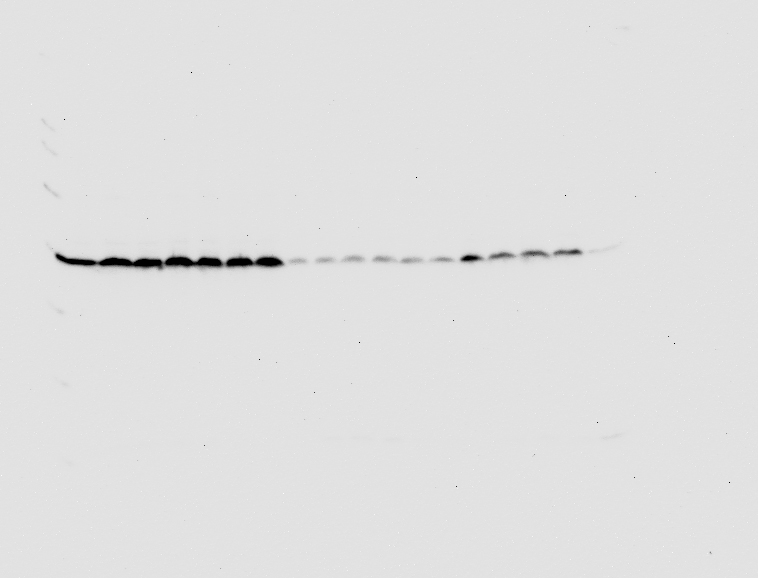

Supplement: Supplementary file 5 — Source Data for Appendix [file MSB-13-904-s013.zip › Source_Data_for_Appendix/Figure_S13/panel_B/UpperPanel_PTEN.jpg]

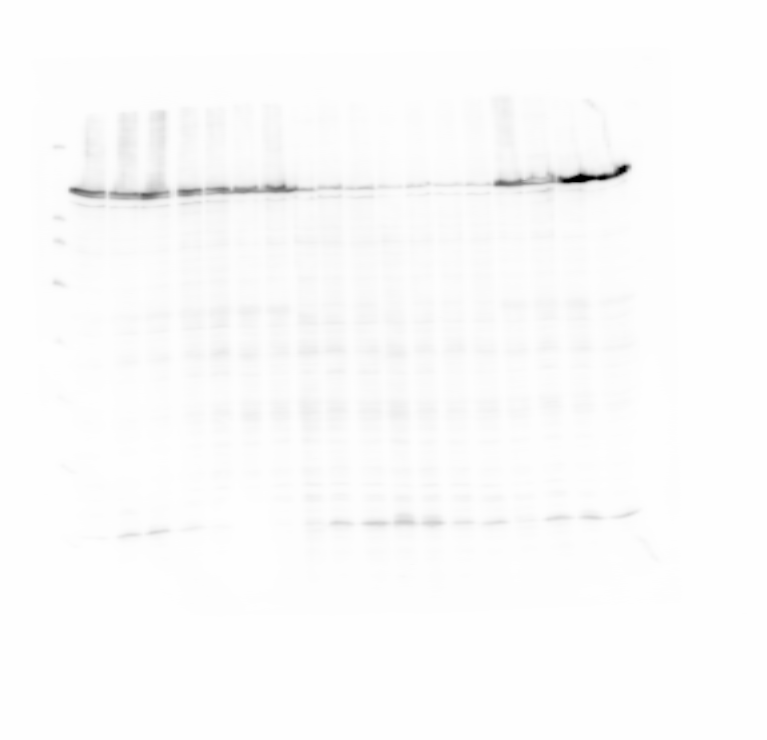

Supplement: Supplementary file 5 — Source Data for Appendix [file MSB-13-904-s013.zip › Source_Data_for_Appendix/Figure_S13/panel_B/LowerPanel_SHIP1.tif]

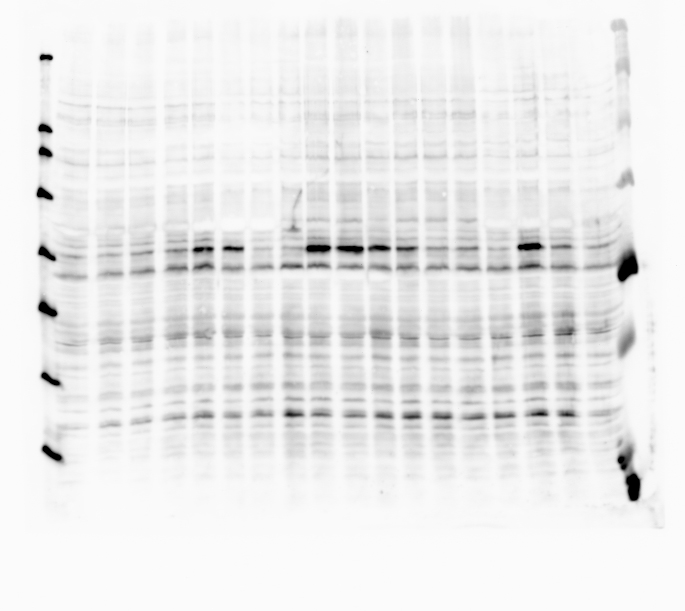

Supplement: Supplementary file 5 — Source Data for Appendix [file MSB-13-904-s013.zip › Source_Data_for_Appendix/Figure_S13/panel_B/LowerPanel_pAKT.jpg]

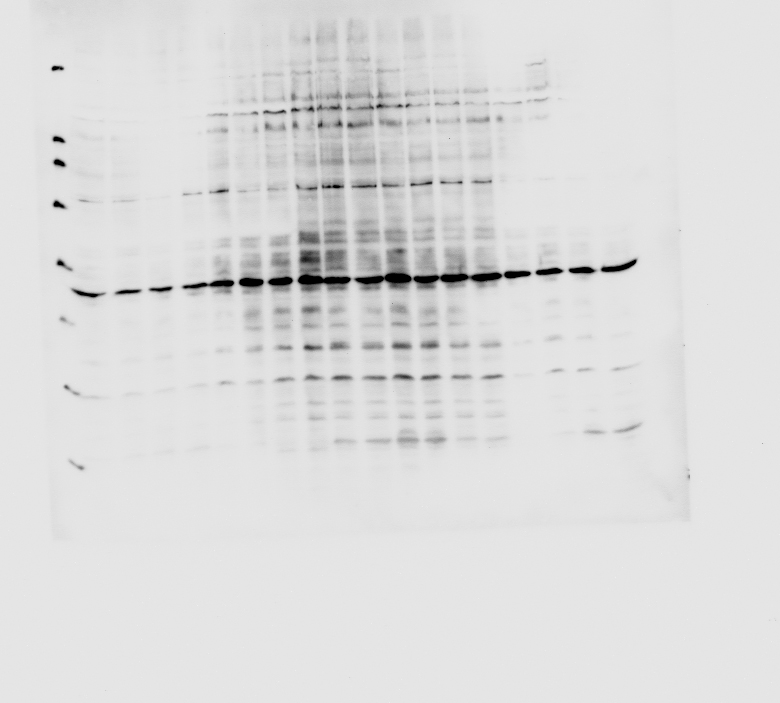

Supplement: Supplementary file 5 — Source Data for Appendix [file MSB-13-904-s013.zip › Source_Data_for_Appendix/Figure_S13/panel_B/LowerPanel_PDI.jpg]

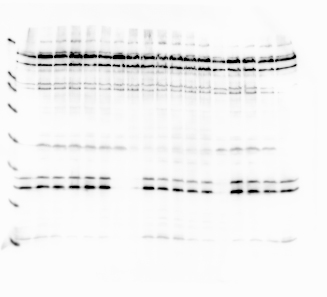

Supplement: Supplementary file 5 — Source Data for Appendix [file MSB-13-904-s013.zip › Source_Data_for_Appendix/Figure_S13/panel_B/UpperPanel_ppERK.jpg]

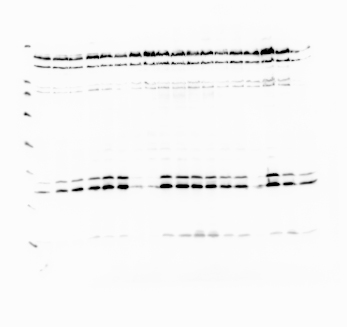

Supplement: Supplementary file 5 — Source Data for Appendix [file MSB-13-904-s013.zip › Source_Data_for_Appendix/Figure_S13/panel_B/LowerPanel_ppERK.jpg]

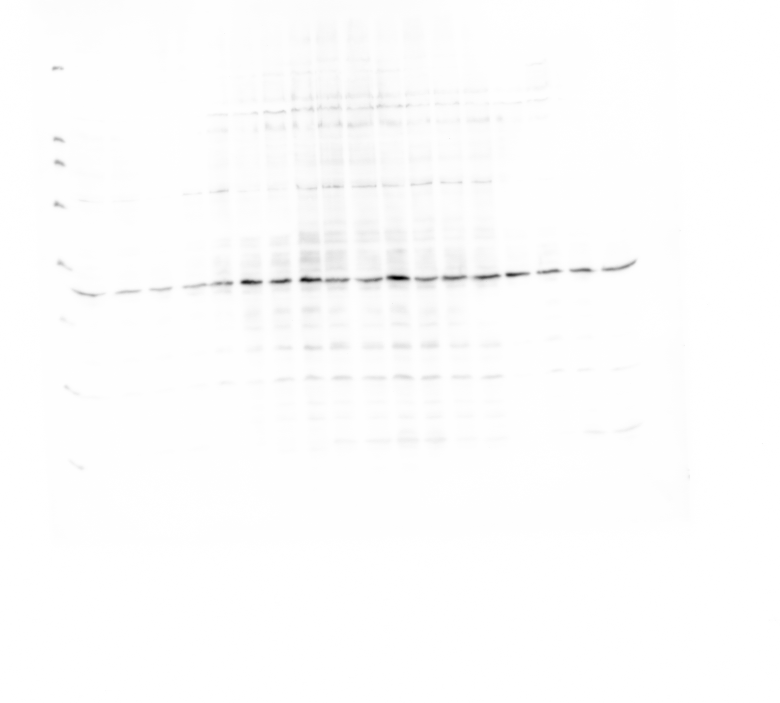

Supplement: Supplementary file 5 — Source Data for Appendix [file MSB-13-904-s013.zip › Source_Data_for_Appendix/Figure_S13/panel_B/LowerPanel_PDI.tif]

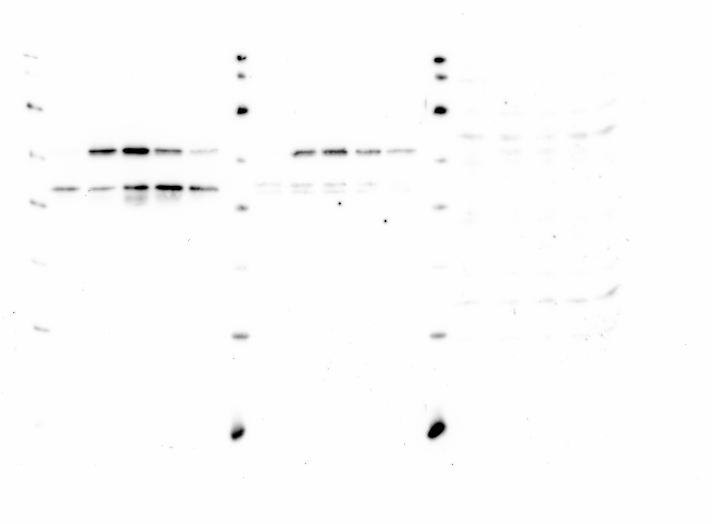

Supplement: Supplementary file 5 — Source Data for Appendix [file MSB-13-904-s013.zip › Source_Data_for_Appendix/Figure_S02/panel_B/CFUE_pAKTThr308.tif]

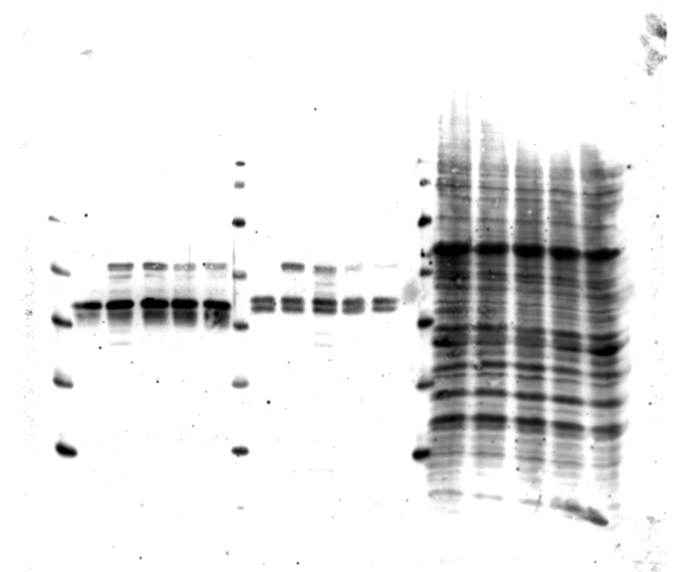

Supplement: Supplementary file 5 — Source Data for Appendix [file MSB-13-904-s013.zip › Source_Data_for_Appendix/Figure_S02/panel_B/BaF3_pAKTThr308.jpg]

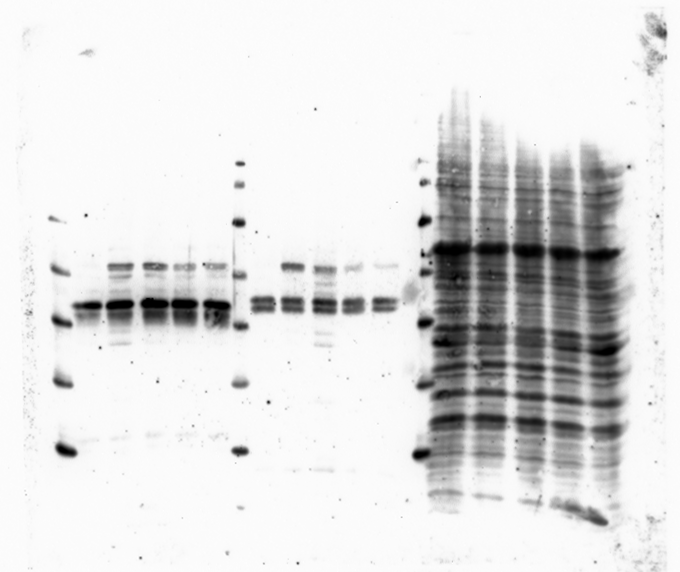

Supplement: Supplementary file 5 — Source Data for Appendix [file MSB-13-904-s013.zip › Source_Data_for_Appendix/Figure_S02/panel_B/BaF3_pAKTThr308.tif]

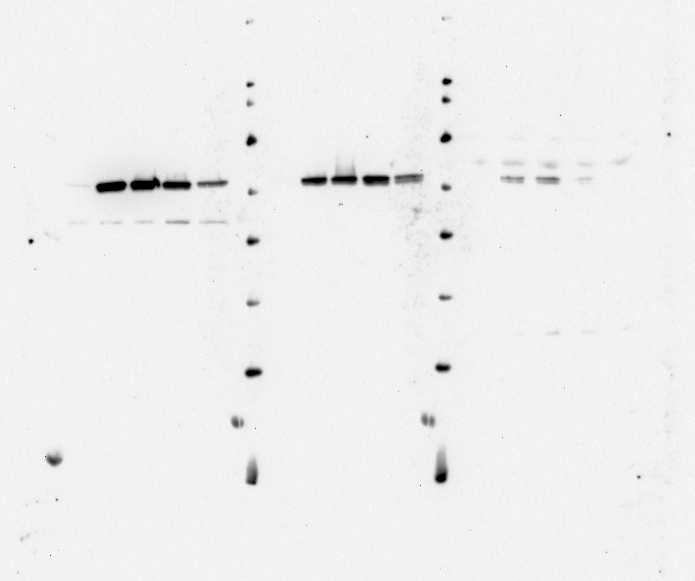

Supplement: Supplementary file 5 — Source Data for Appendix [file MSB-13-904-s013.zip › Source_Data_for_Appendix/Figure_S02/panel_B/CFUE_pAKTSer473.jpg]

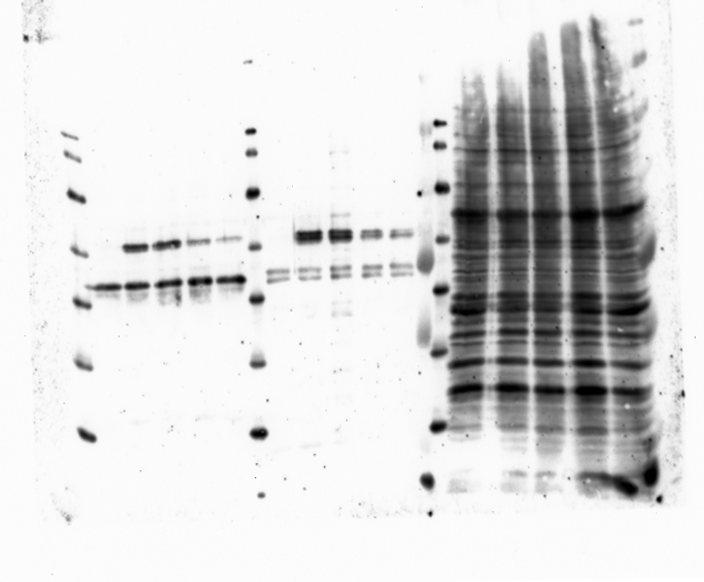

Supplement: Supplementary file 5 — Source Data for Appendix [file MSB-13-904-s013.zip › Source_Data_for_Appendix/Figure_S02/panel_B/BaF3_pAKTSer473.tif]

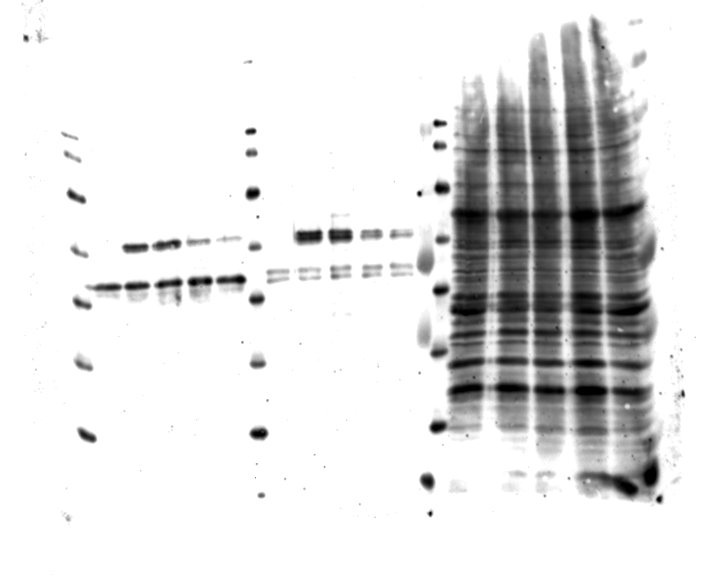

Supplement: Supplementary file 5 — Source Data for Appendix [file MSB-13-904-s013.zip › Source_Data_for_Appendix/Figure_S02/panel_B/BaF3_pAKTSer473.jpg]

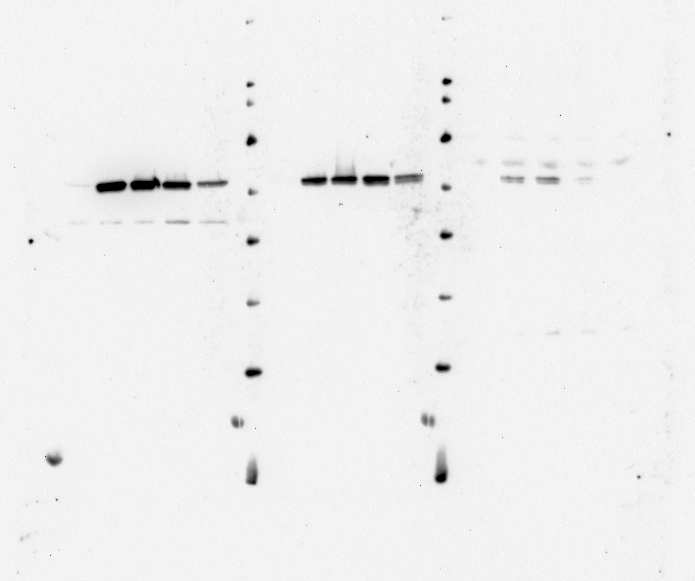

Supplement: Supplementary file 5 — Source Data for Appendix [file MSB-13-904-s013.zip › Source_Data_for_Appendix/Figure_S02/panel_B/CFUE_pAKTSer473.tif]

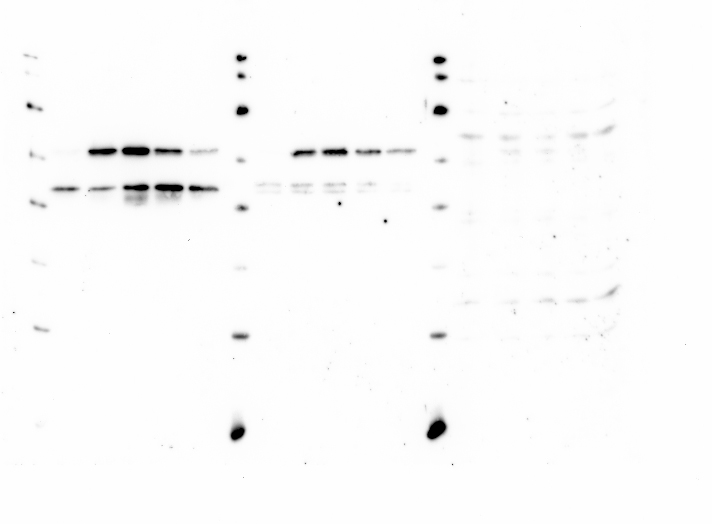

Supplement: Supplementary file 5 — Source Data for Appendix [file MSB-13-904-s013.zip › Source_Data_for_Appendix/Figure_S02/panel_B/CFUE_pAKTThr308.jpg]

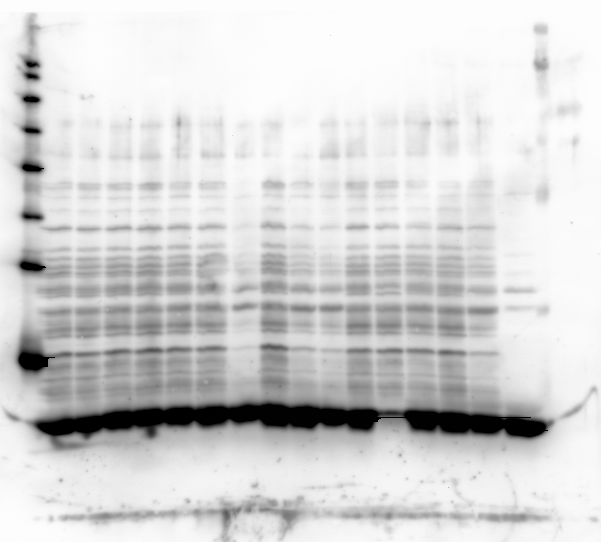

Supplement: Supplementary file 5 — Source Data for Appendix [file MSB-13-904-s013.zip › Source_Data_for_Appendix/Figure_S10/panel_A/LowerPanel_CFUE_GTPRas.tif]

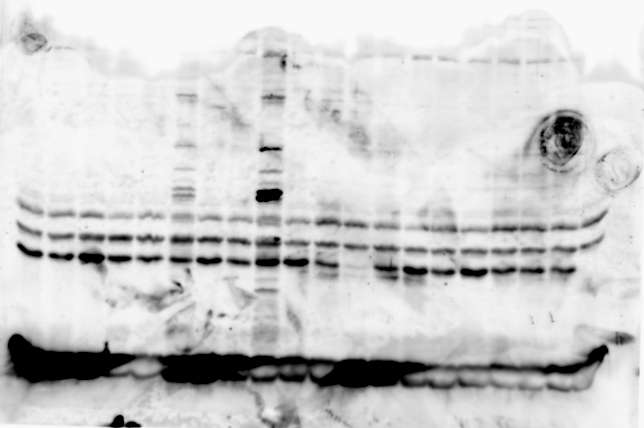

Supplement: Supplementary file 5 — Source Data for Appendix [file MSB-13-904-s013.zip › Source_Data_for_Appendix/Figure_S10/panel_A/LowerPanel_BaF3_GTPRas.jpg]

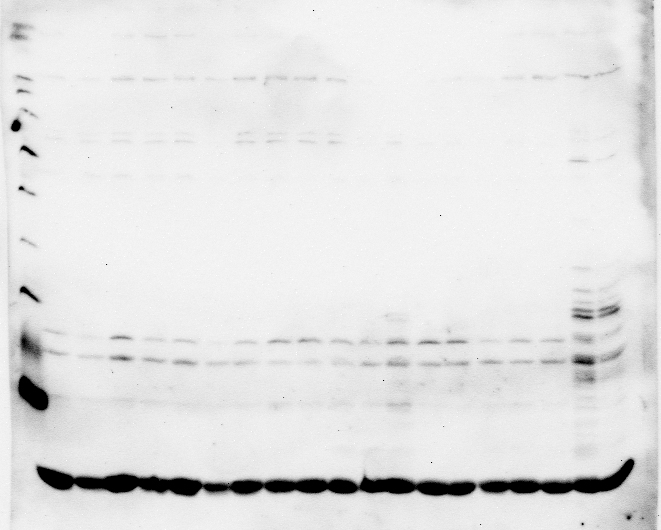

Supplement: Supplementary file 5 — Source Data for Appendix [file MSB-13-904-s013.zip › Source_Data_for_Appendix/Figure_S10/panel_A/UpperPanel_BaF3_RafRDB.jpg]

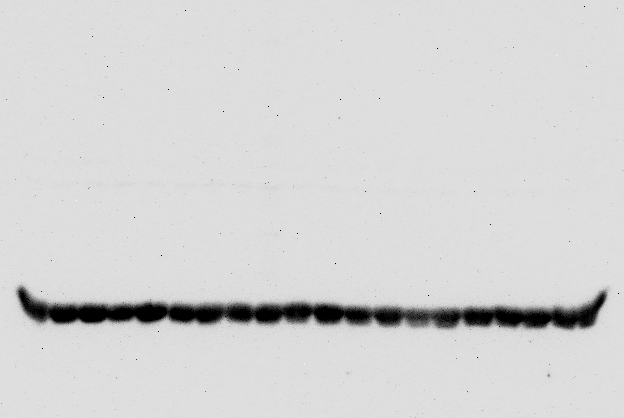

Supplement: Supplementary file 5 — Source Data for Appendix [file MSB-13-904-s013.zip › Source_Data_for_Appendix/Figure_S10/panel_A/LowerPanel_BaF3_RafRDB.jpg]

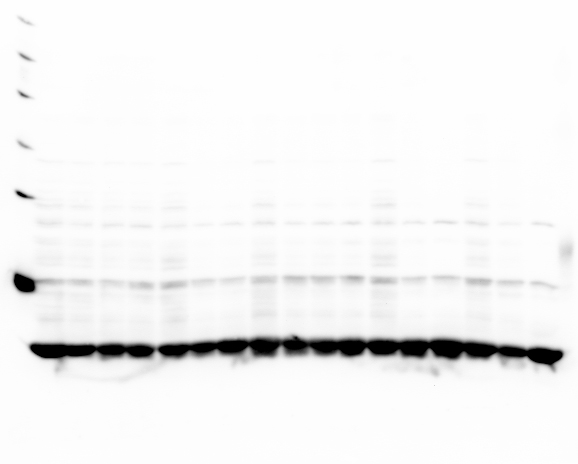

Supplement: Supplementary file 5 — Source Data for Appendix [file MSB-13-904-s013.zip › Source_Data_for_Appendix/Figure_S10/panel_A/UpperPanel_CFUE_RafRDB.jpg]

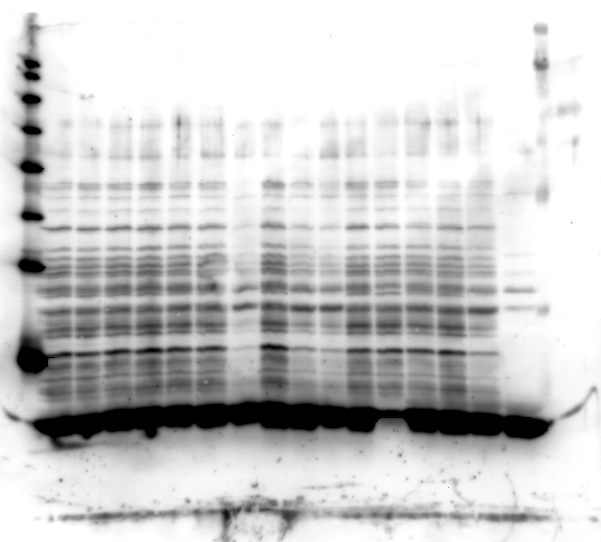

Supplement: Supplementary file 5 — Source Data for Appendix [file MSB-13-904-s013.zip › Source_Data_for_Appendix/Figure_S10/panel_A/LowerPanel_CFUE_GTPRas.jpg]

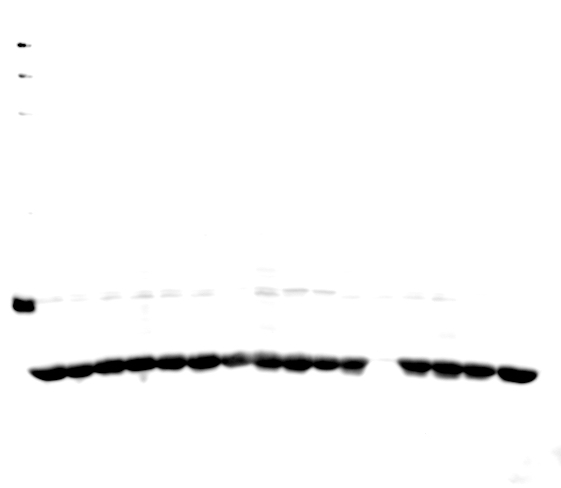

Supplement: Supplementary file 5 — Source Data for Appendix [file MSB-13-904-s013.zip › Source_Data_for_Appendix/Figure_S10/panel_A/LowerPanel_CFUE_RafRDB.jpg]

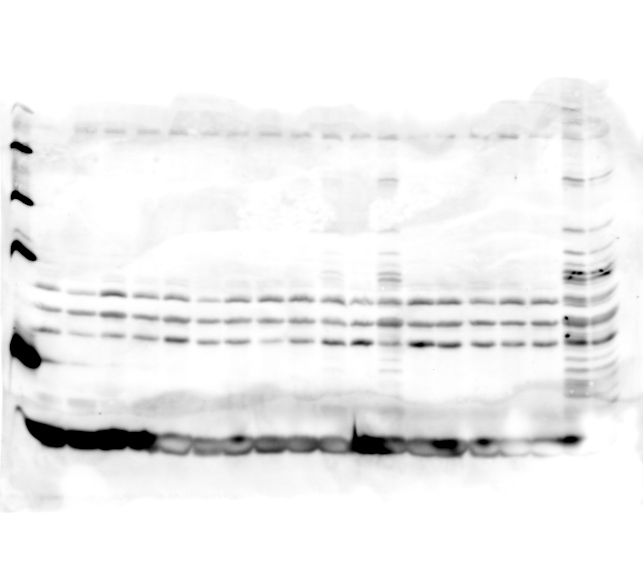

Supplement: Supplementary file 5 — Source Data for Appendix [file MSB-13-904-s013.zip › Source_Data_for_Appendix/Figure_S10/panel_A/UpperPanel_BaF3_GTPRas.jpg]

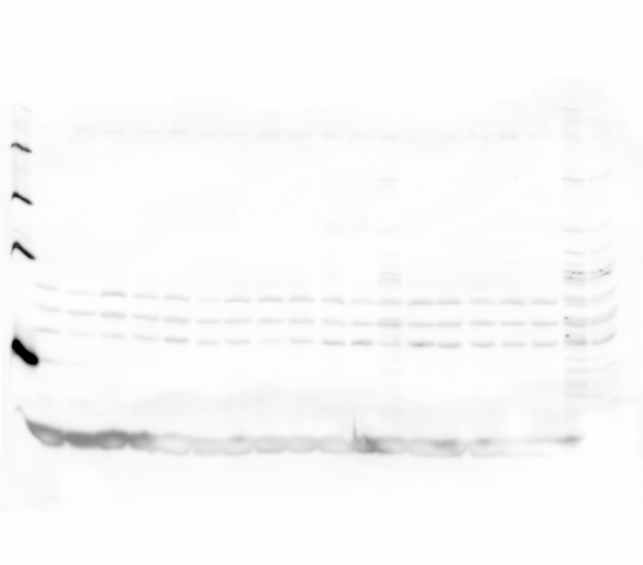

Supplement: Supplementary file 5 — Source Data for Appendix [file MSB-13-904-s013.zip › Source_Data_for_Appendix/Figure_S10/panel_A/UpperPanel_BaF3_GTPRas.tif]

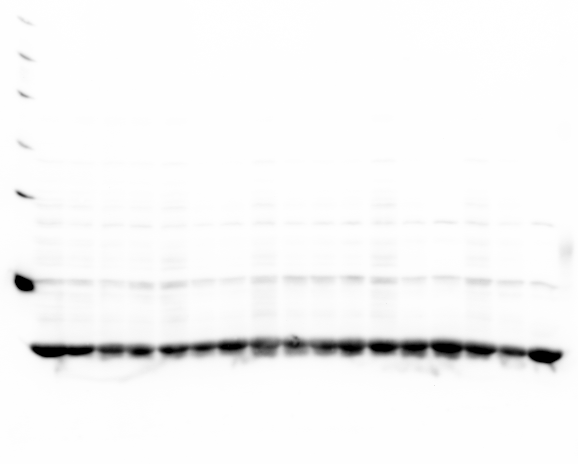

Supplement: Supplementary file 5 — Source Data for Appendix [file MSB-13-904-s013.zip › Source_Data_for_Appendix/Figure_S10/panel_A/UpperPanel_CFUE_RafRDB.tif]

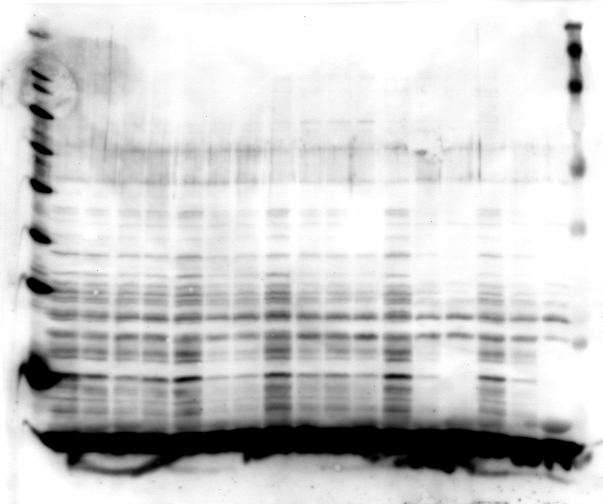

Supplement: Supplementary file 5 — Source Data for Appendix [file MSB-13-904-s013.zip › Source_Data_for_Appendix/Figure_S10/panel_A/UpperPanel_CFUE_GTPRas.jpg]

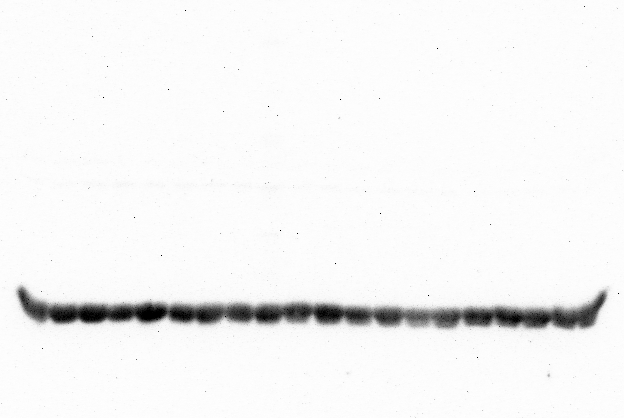

Supplement: Supplementary file 5 — Source Data for Appendix [file MSB-13-904-s013.zip › Source_Data_for_Appendix/Figure_S10/panel_A/LowerPanel_BaF3_RafRDB.tif]

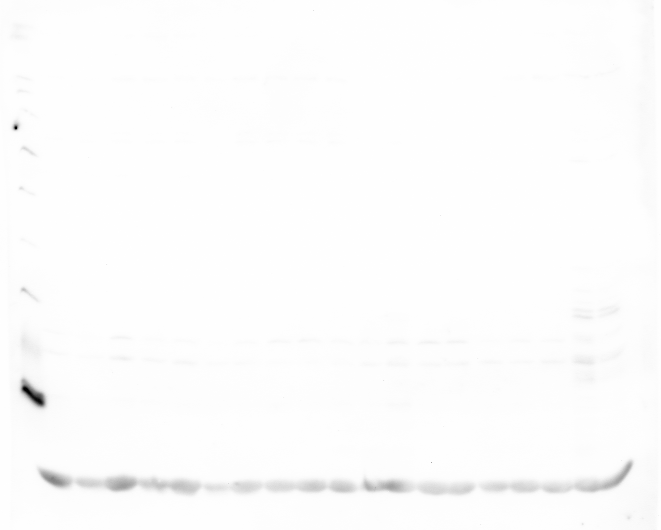

Supplement: Supplementary file 5 — Source Data for Appendix [file MSB-13-904-s013.zip › Source_Data_for_Appendix/Figure_S10/panel_A/UpperPanel_BaF3_RafRDB.tif]

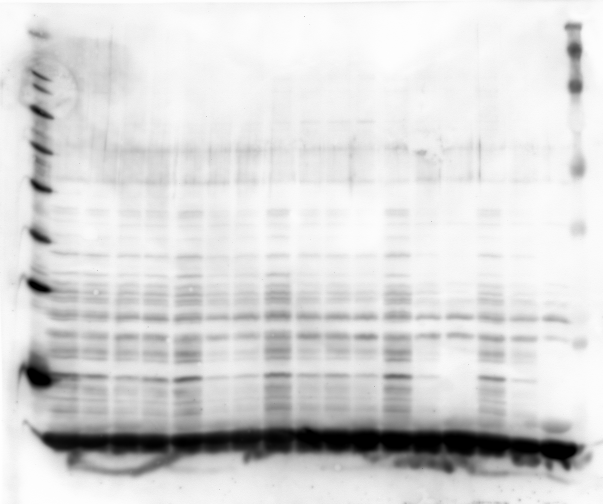

Supplement: Supplementary file 5 — Source Data for Appendix [file MSB-13-904-s013.zip › Source_Data_for_Appendix/Figure_S10/panel_A/UpperPanel_CFUE_GTPRas.tif]

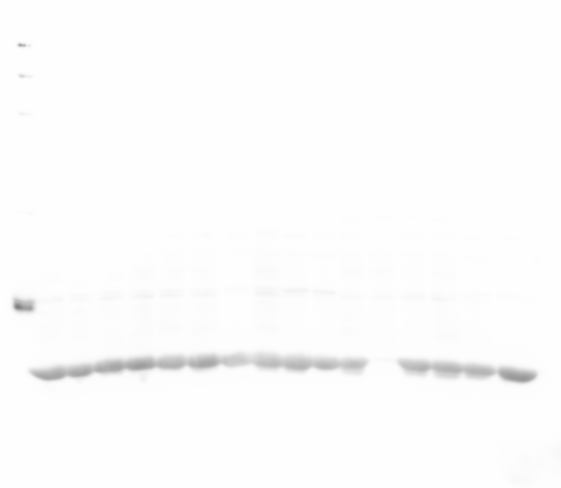

Supplement: Supplementary file 5 — Source Data for Appendix [file MSB-13-904-s013.zip › Source_Data_for_Appendix/Figure_S10/panel_A/LowerPanel_CFUE_RafRDB.tif]

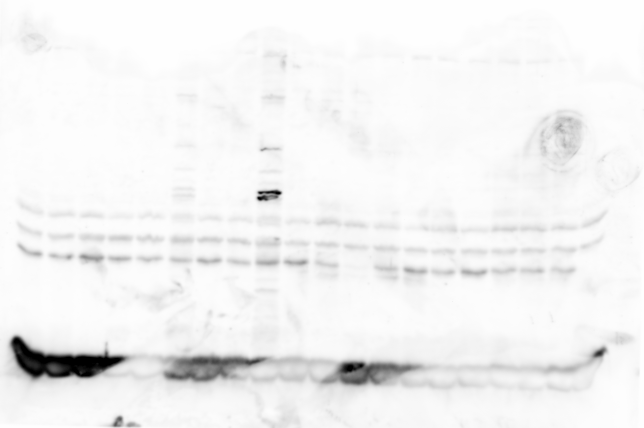

Supplement: Supplementary file 5 — Source Data for Appendix [file MSB-13-904-s013.zip › Source_Data_for_Appendix/Figure_S10/panel_A/LowerPanel_BaF3_GTPRas.tif]
