# Supplementary figures and images for: Protein abundance of AKT and ERK pathway components governs cell type‐specific regulation of proliferation (part 3 of 3)
Source: Mol Syst Biol. 2017 Jan 25;13(1):904. doi: 10.15252/msb.20167258 (PMC5293153; doi:10.15252/msb.20167258)

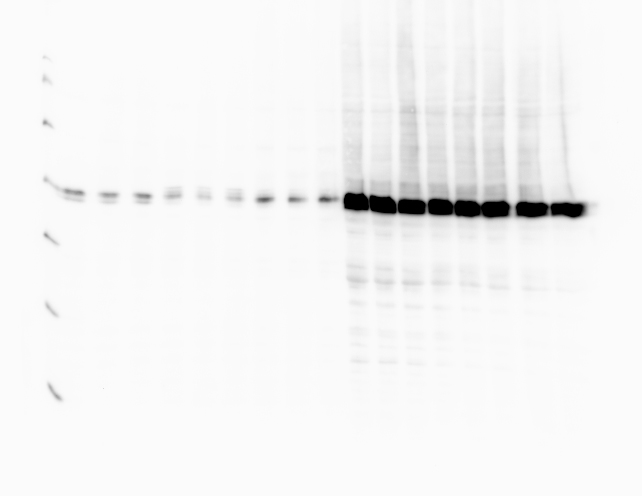

Supplement: Supplementary file 7 — Source Data for Figure 1 [file MSB-13-904-s005.zip › Source_Data_for_Figure_1/Figure01D/All_JPEG/Figure01D_AKT.jpg]

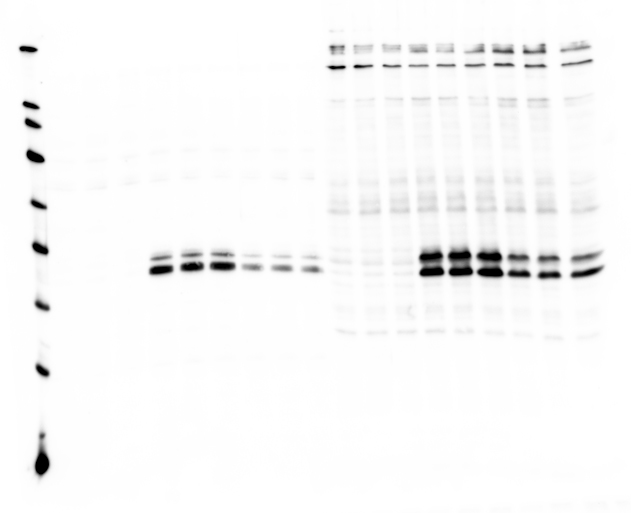

Supplement: Supplementary file 7 — Source Data for Figure 1 [file MSB-13-904-s005.zip › Source_Data_for_Figure_1/Figure01D/All_JPEG/Figure01D_ppERK.jpg]

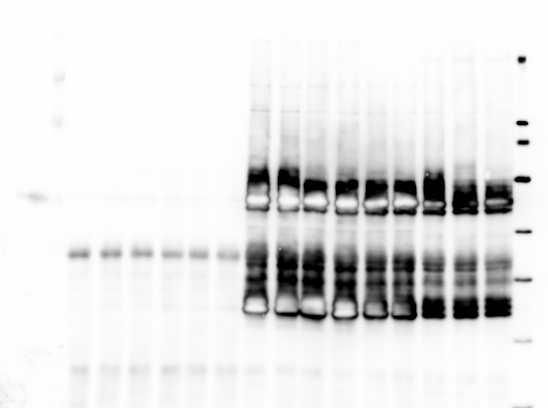

Supplement: Supplementary file 7 — Source Data for Figure 1 [file MSB-13-904-s005.zip › Source_Data_for_Figure_1/Figure01D/All_JPEG/Figure01D_EpoR.jpg]

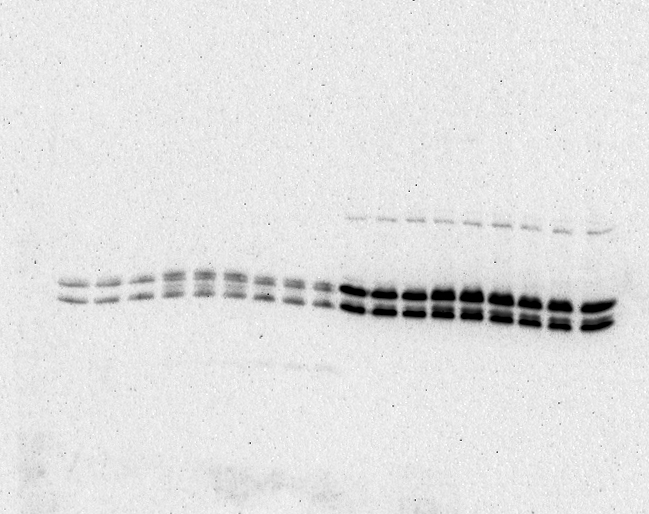

Supplement: Supplementary file 7 — Source Data for Figure 1 [file MSB-13-904-s005.zip › Source_Data_for_Figure_1/Figure01D/All_JPEG/Figure01D_ERK.jpg]

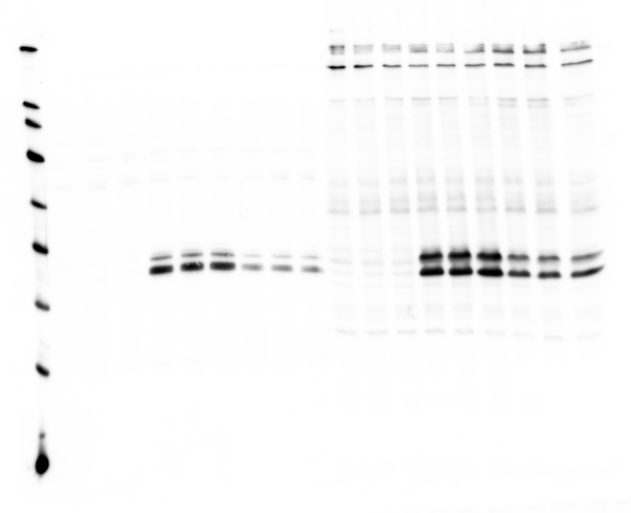

Supplement: Supplementary file 7 — Source Data for Figure 1 [file MSB-13-904-s005.zip › Source_Data_for_Figure_1/Figure01D/All_RAW/Figure01D_ppERK.tif]

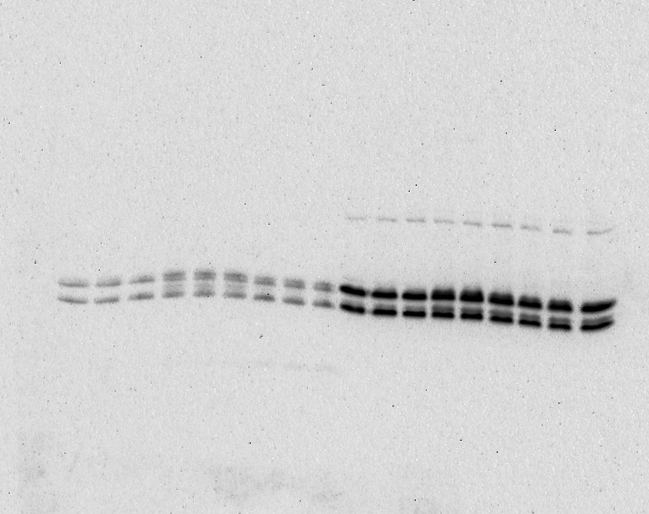

Supplement: Supplementary file 7 — Source Data for Figure 1 [file MSB-13-904-s005.zip › Source_Data_for_Figure_1/Figure01D/All_RAW/Figure01D_ERK.tif]

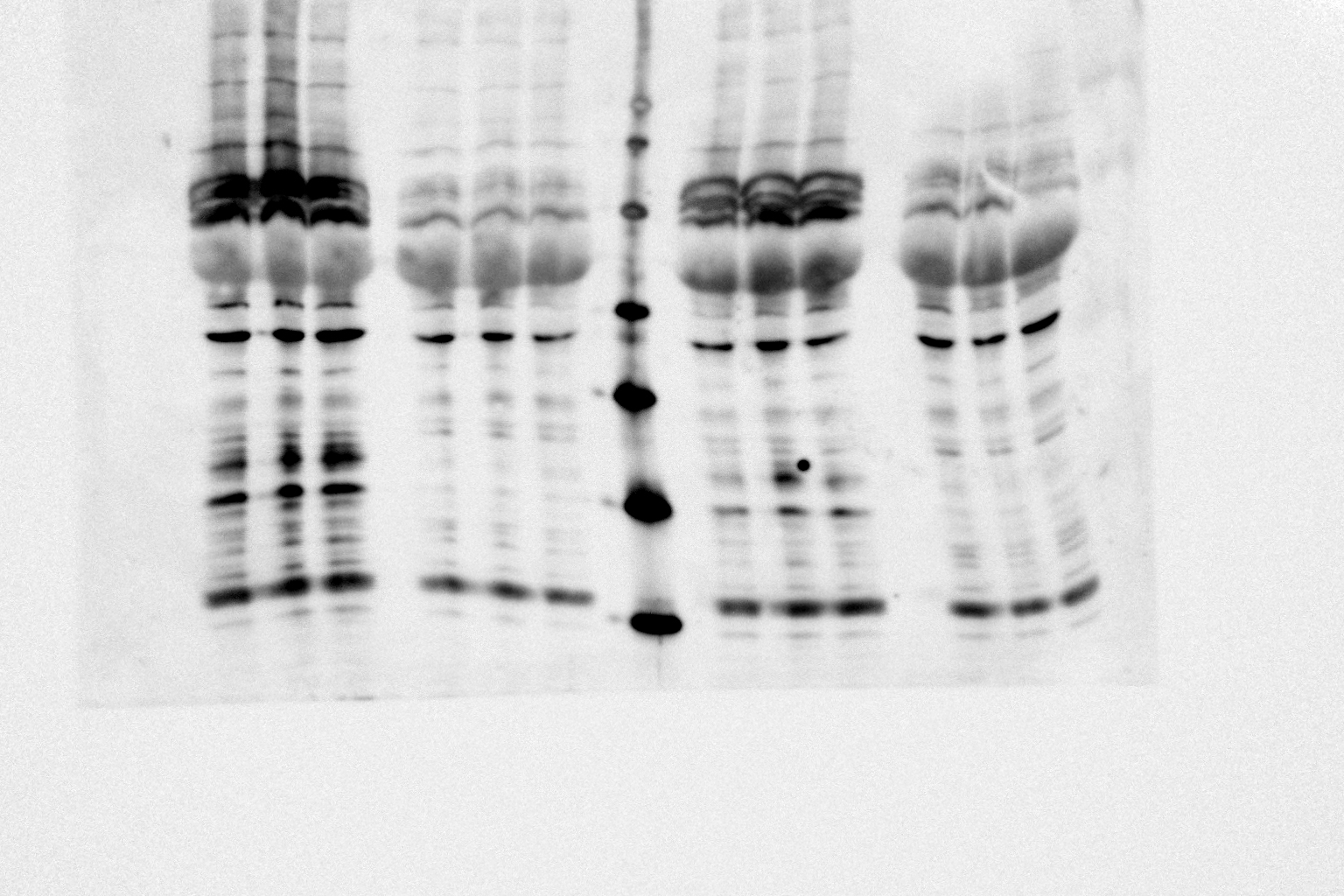

Supplement: Supplementary file 10 — Source Data for Figure 4 [file MSB-13-904-s008.zip › Source_Data_for_Figure_4/Figure04C/All_JPEG/Figure04C_PDI.jpg]

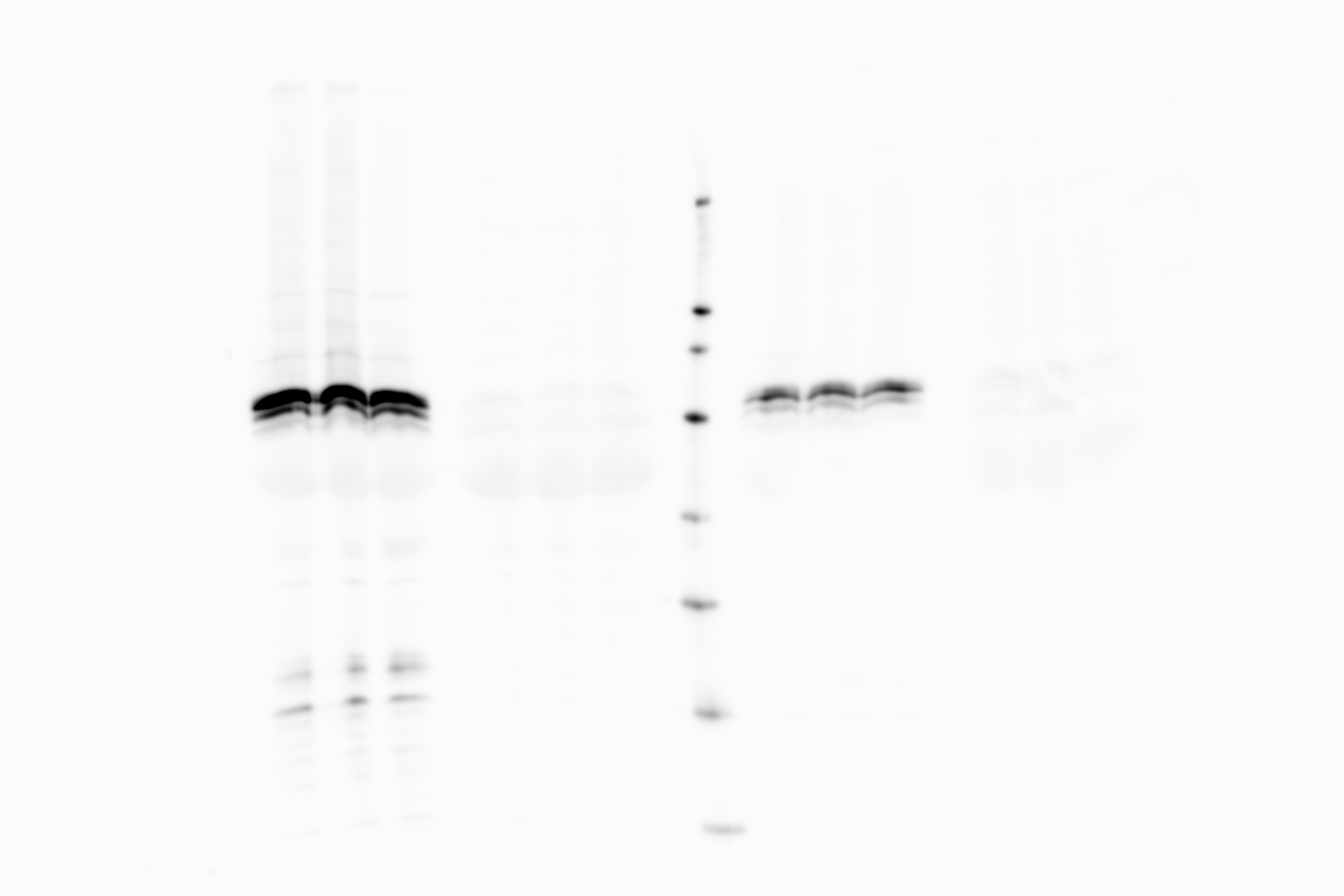

Supplement: Supplementary file 10 — Source Data for Figure 4 [file MSB-13-904-s008.zip › Source_Data_for_Figure_4/Figure04C/All_JPEG/Figure04C_RSK.jpg]

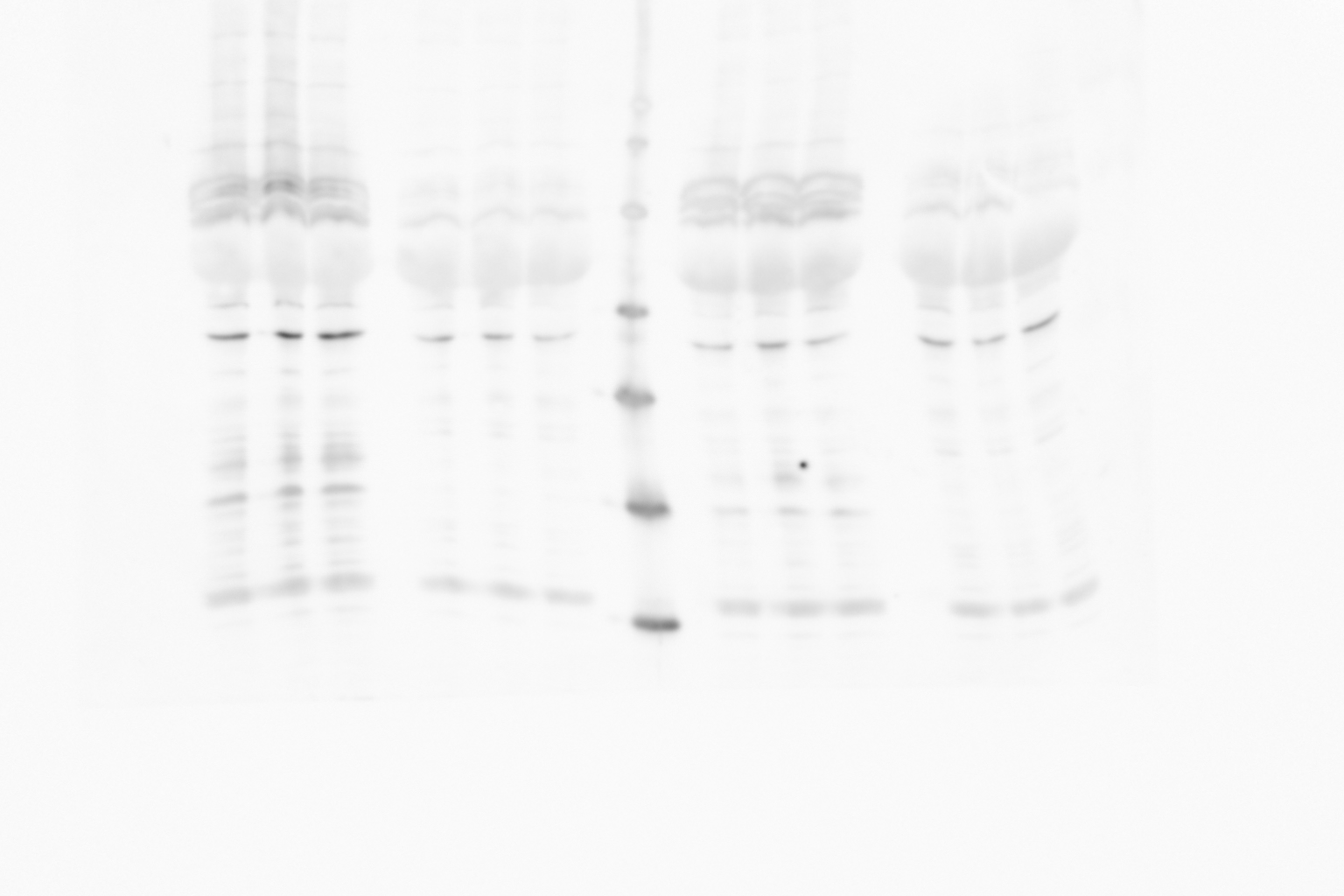

Supplement: Supplementary file 10 — Source Data for Figure 4 [file MSB-13-904-s008.zip › Source_Data_for_Figure_4/Figure04C/All_RAW/Figure04C_PDI.tif]

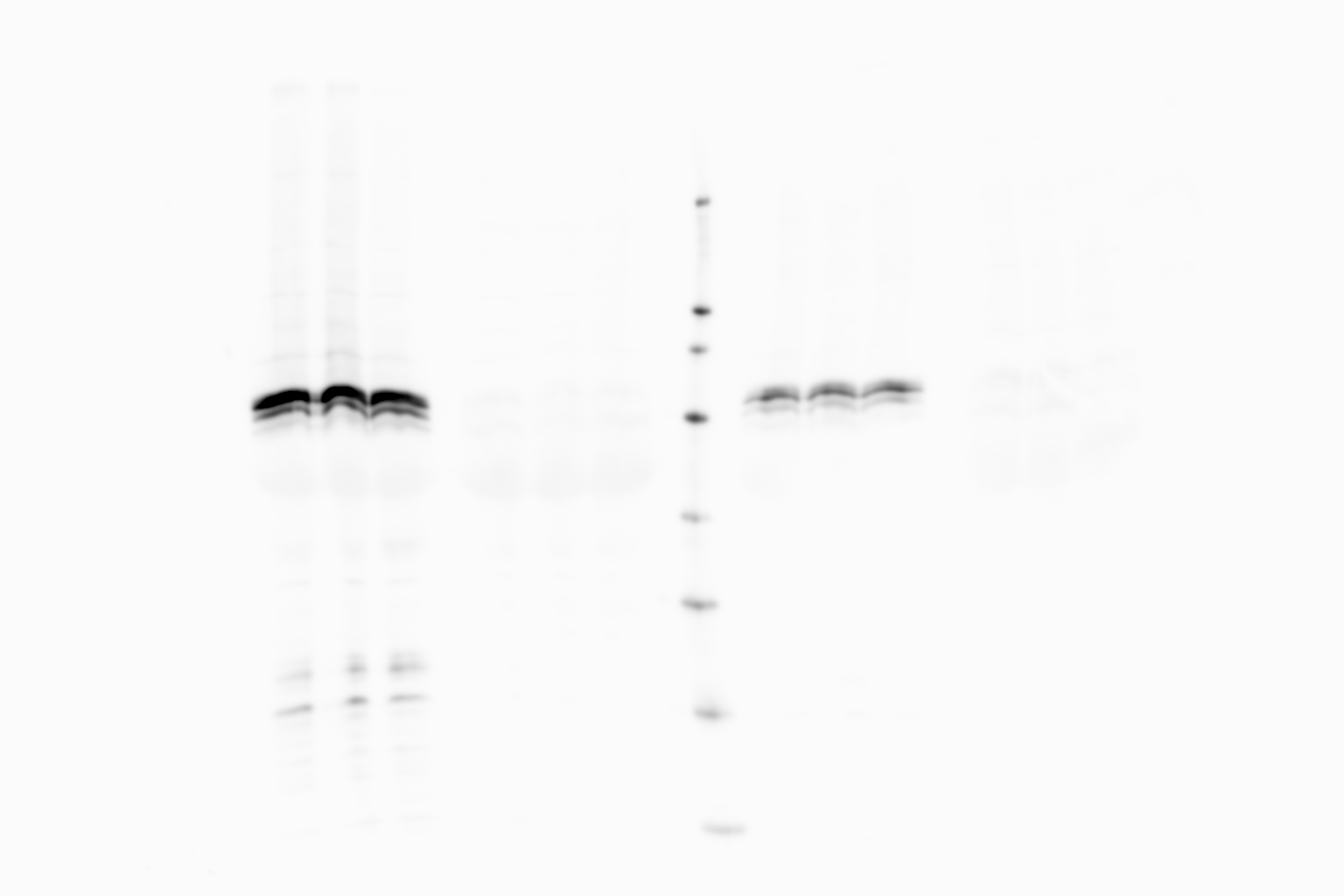

Supplement: Supplementary file 10 — Source Data for Figure 4 [file MSB-13-904-s008.zip › Source_Data_for_Figure_4/Figure04C/All_RAW/Figure04C_RSK.tif]

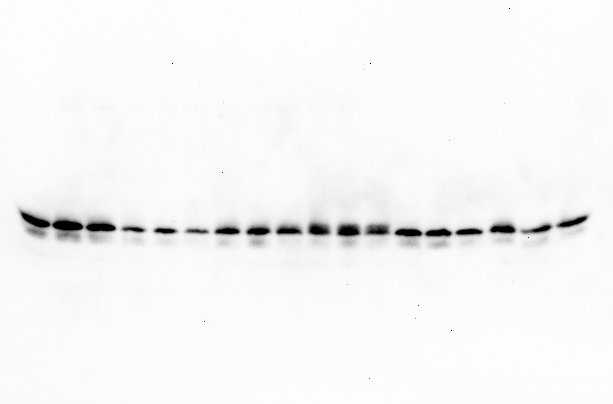

Supplement: Supplementary file 10 — Source Data for Figure 4 [file MSB-13-904-s008.zip › Source_Data_for_Figure_4/Figure04D/All_JPEG/Figure04D_CFUE_totalS6.jpg]

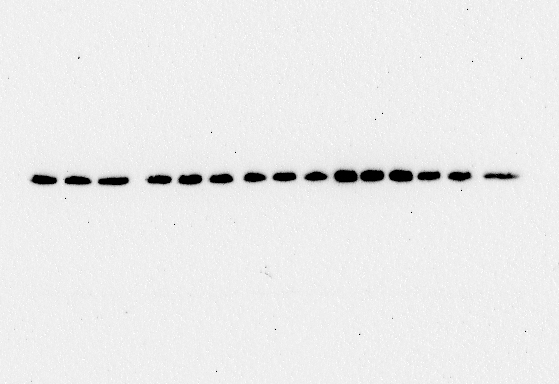

Supplement: Supplementary file 10 — Source Data for Figure 4 [file MSB-13-904-s008.zip › Source_Data_for_Figure_4/Figure04D/All_JPEG/Figure04D_BaF3_actin.jpg]

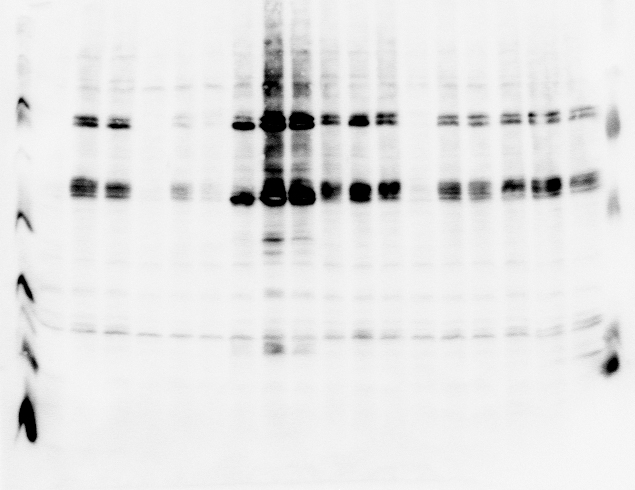

Supplement: Supplementary file 10 — Source Data for Figure 4 [file MSB-13-904-s008.zip › Source_Data_for_Figure_4/Figure04D/All_JPEG/Figure04D_CFUE_pAKT.jpg]

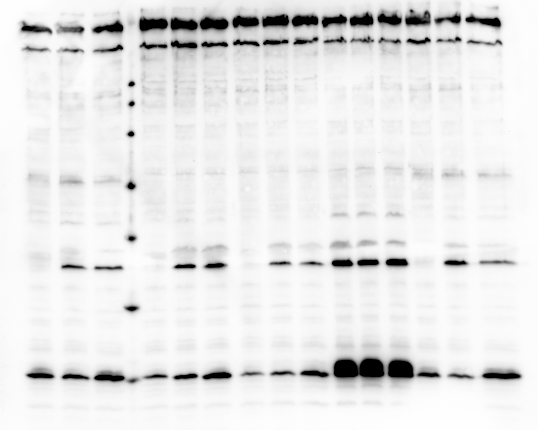

Supplement: Supplementary file 10 — Source Data for Figure 4 [file MSB-13-904-s008.zip › Source_Data_for_Figure_4/Figure04D/All_JPEG/Figure04D_BaF3_ppERK.jpg]

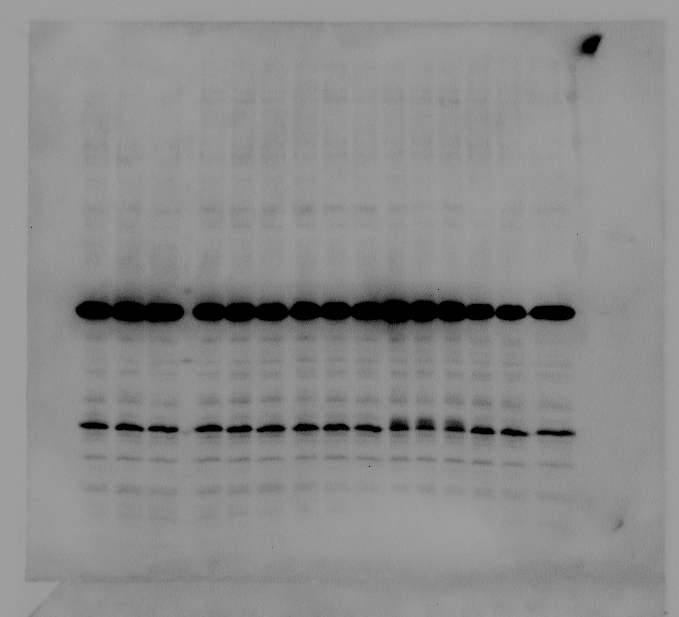

Supplement: Supplementary file 10 — Source Data for Figure 4 [file MSB-13-904-s008.zip › Source_Data_for_Figure_4/Figure04D/All_JPEG/Figure04D_BaF3_totalS6.jpg]

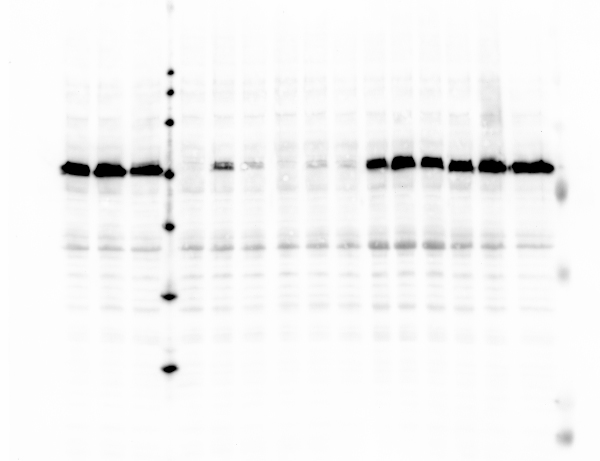

Supplement: Supplementary file 10 — Source Data for Figure 4 [file MSB-13-904-s008.zip › Source_Data_for_Figure_4/Figure04D/All_JPEG/Figure04D_BaF3_pAKT.jpg]

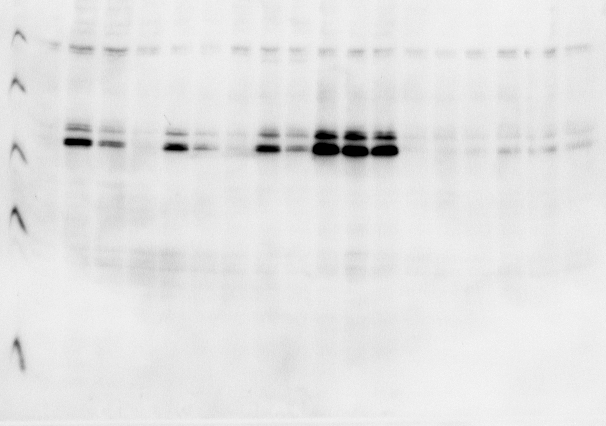

Supplement: Supplementary file 10 — Source Data for Figure 4 [file MSB-13-904-s008.zip › Source_Data_for_Figure_4/Figure04D/All_JPEG/Figure04D_CFUE_ppERK.jpg]

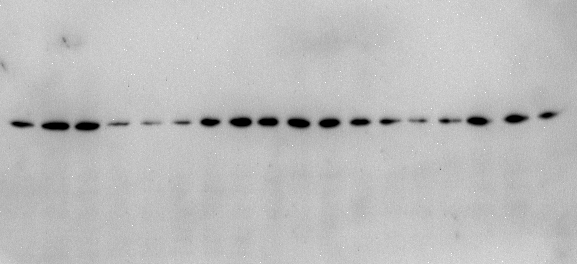

Supplement: Supplementary file 10 — Source Data for Figure 4 [file MSB-13-904-s008.zip › Source_Data_for_Figure_4/Figure04D/All_JPEG/Figure04D_CFUE_actin.jpg]

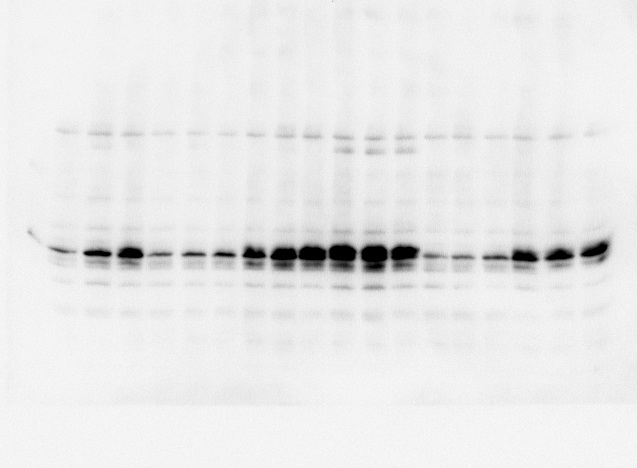

Supplement: Supplementary file 10 — Source Data for Figure 4 [file MSB-13-904-s008.zip › Source_Data_for_Figure_4/Figure04D/All_JPEG/Figure04D_CFUE_pS6.jpg]

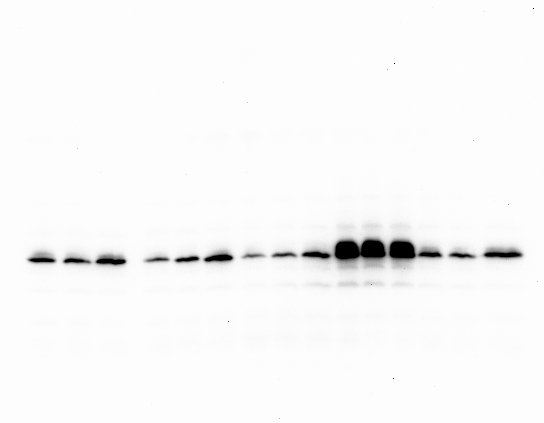

Supplement: Supplementary file 10 — Source Data for Figure 4 [file MSB-13-904-s008.zip › Source_Data_for_Figure_4/Figure04D/All_JPEG/Figure04D_BaF3_pS6.jpg]

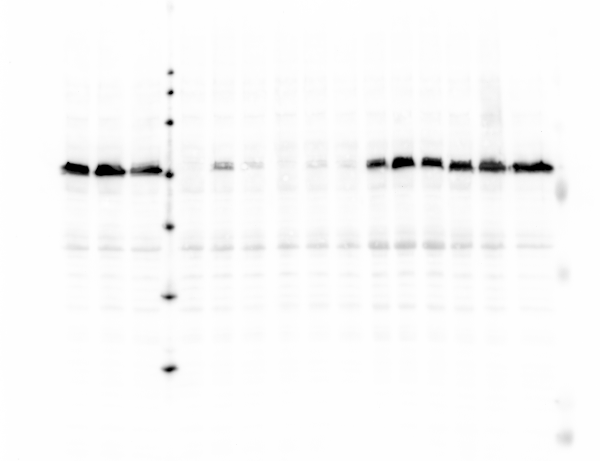

Supplement: Supplementary file 10 — Source Data for Figure 4 [file MSB-13-904-s008.zip › Source_Data_for_Figure_4/Figure04D/All_RAW/Figure04D_BaF3_pAKT.tif]

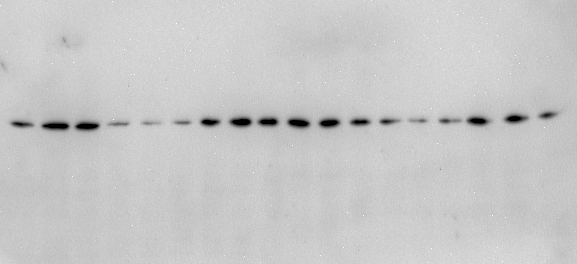

Supplement: Supplementary file 10 — Source Data for Figure 4 [file MSB-13-904-s008.zip › Source_Data_for_Figure_4/Figure04D/All_RAW/Figure04D_CFUE_actin.tif]

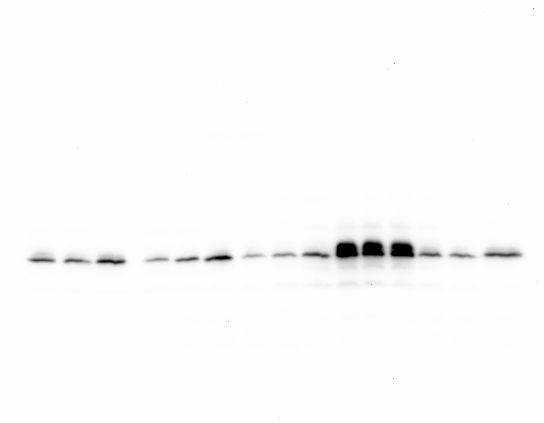

Supplement: Supplementary file 10 — Source Data for Figure 4 [file MSB-13-904-s008.zip › Source_Data_for_Figure_4/Figure04D/All_RAW/Figure04D_BaF3_pS6.tif]

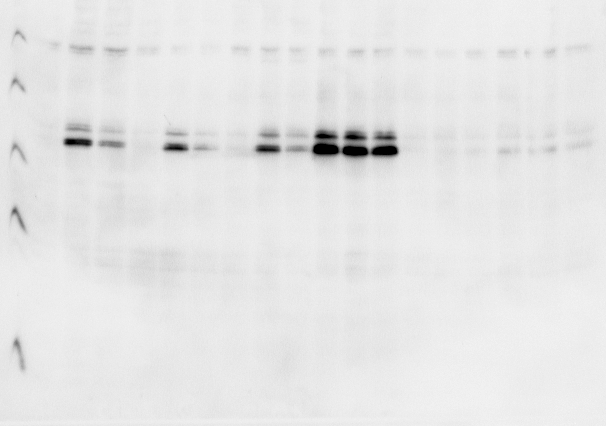

Supplement: Supplementary file 10 — Source Data for Figure 4 [file MSB-13-904-s008.zip › Source_Data_for_Figure_4/Figure04D/All_RAW/Figure04D_CFUE_ppERK.tif]

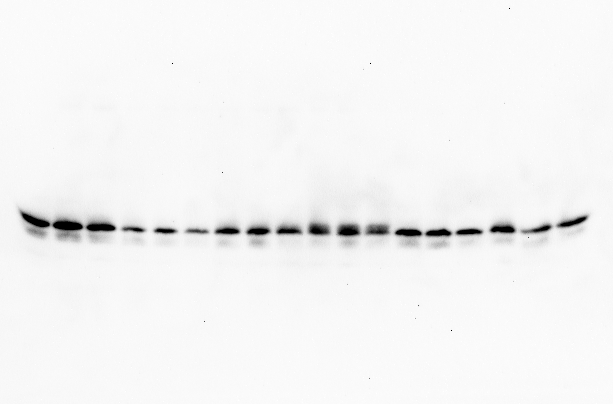

Supplement: Supplementary file 10 — Source Data for Figure 4 [file MSB-13-904-s008.zip › Source_Data_for_Figure_4/Figure04D/All_RAW/Figure04D_CFUE_totalS6.tif]

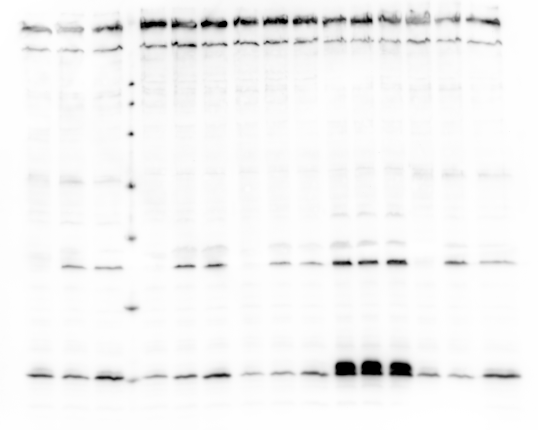

Supplement: Supplementary file 10 — Source Data for Figure 4 [file MSB-13-904-s008.zip › Source_Data_for_Figure_4/Figure04D/All_RAW/Figure04D_BaF3_ppERK.tif]

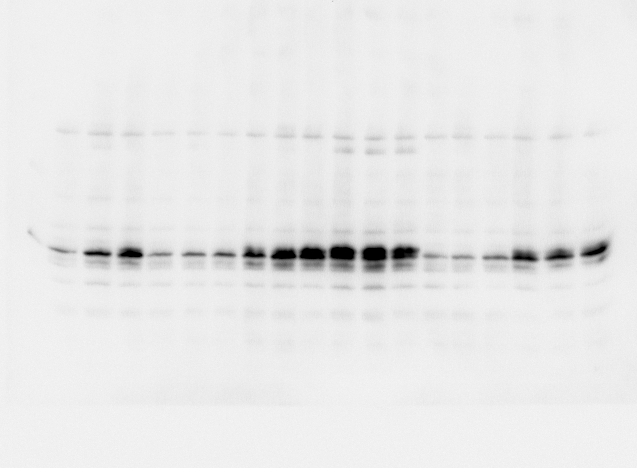

Supplement: Supplementary file 10 — Source Data for Figure 4 [file MSB-13-904-s008.zip › Source_Data_for_Figure_4/Figure04D/All_RAW/Figure04D_CFUE_pS6.tif]

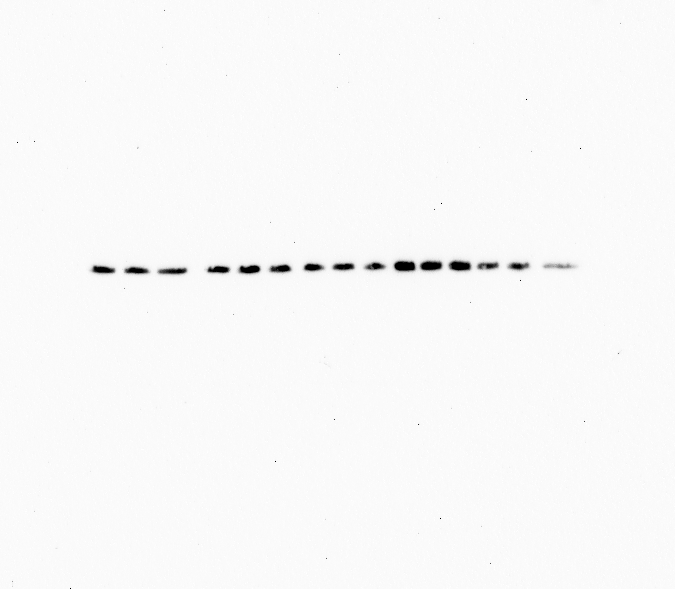

Supplement: Supplementary file 10 — Source Data for Figure 4 [file MSB-13-904-s008.zip › Source_Data_for_Figure_4/Figure04D/All_RAW/Figure04D_BaF3_actin.tif]

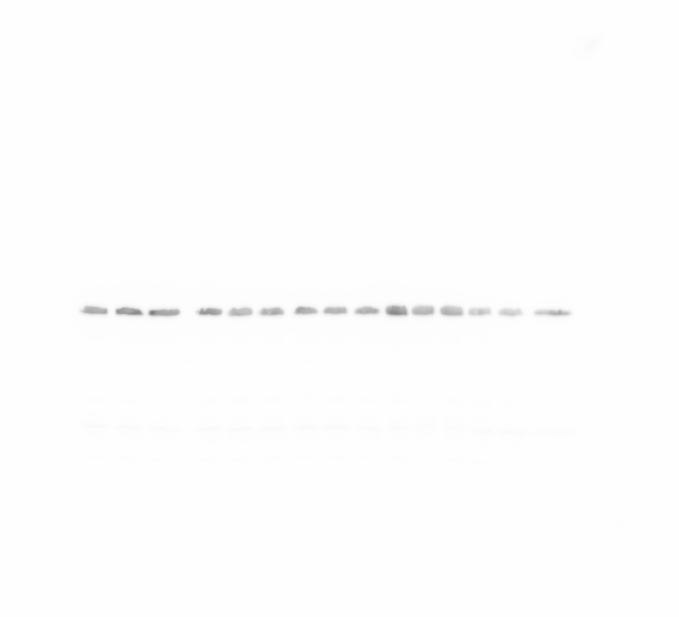

Supplement: Supplementary file 10 — Source Data for Figure 4 [file MSB-13-904-s008.zip › Source_Data_for_Figure_4/Figure04D/All_RAW/Figure04D_BaF3_totalS6.tif]

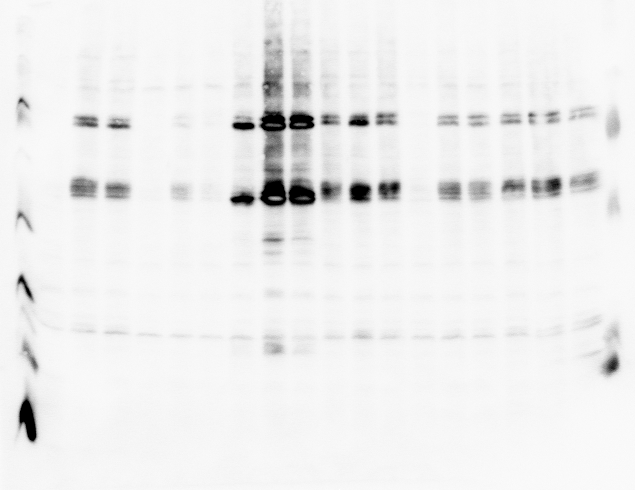

Supplement: Supplementary file 10 — Source Data for Figure 4 [file MSB-13-904-s008.zip › Source_Data_for_Figure_4/Figure04D/All_RAW/Figure04D_CFUE_pAKT.tif]

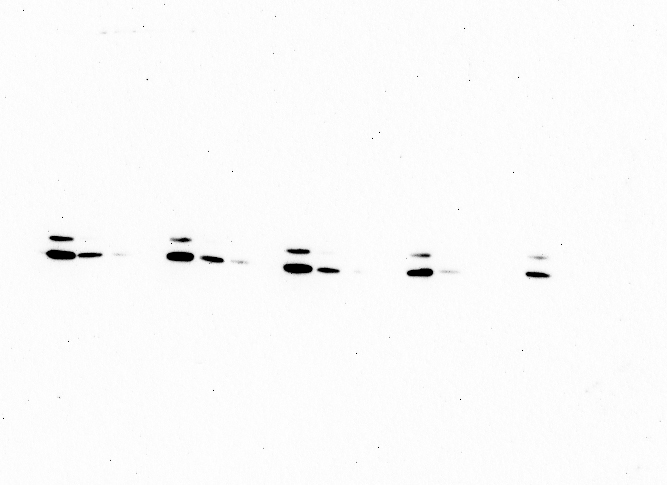

Supplement: Supplementary file 11 — Source Data for Figure 5 [file MSB-13-904-s009.zip › Source_Data_for_Figure_5/Figure05A/All_JPEG/Figure05A_CFUE_AKTVIII_ppERK.jpg]

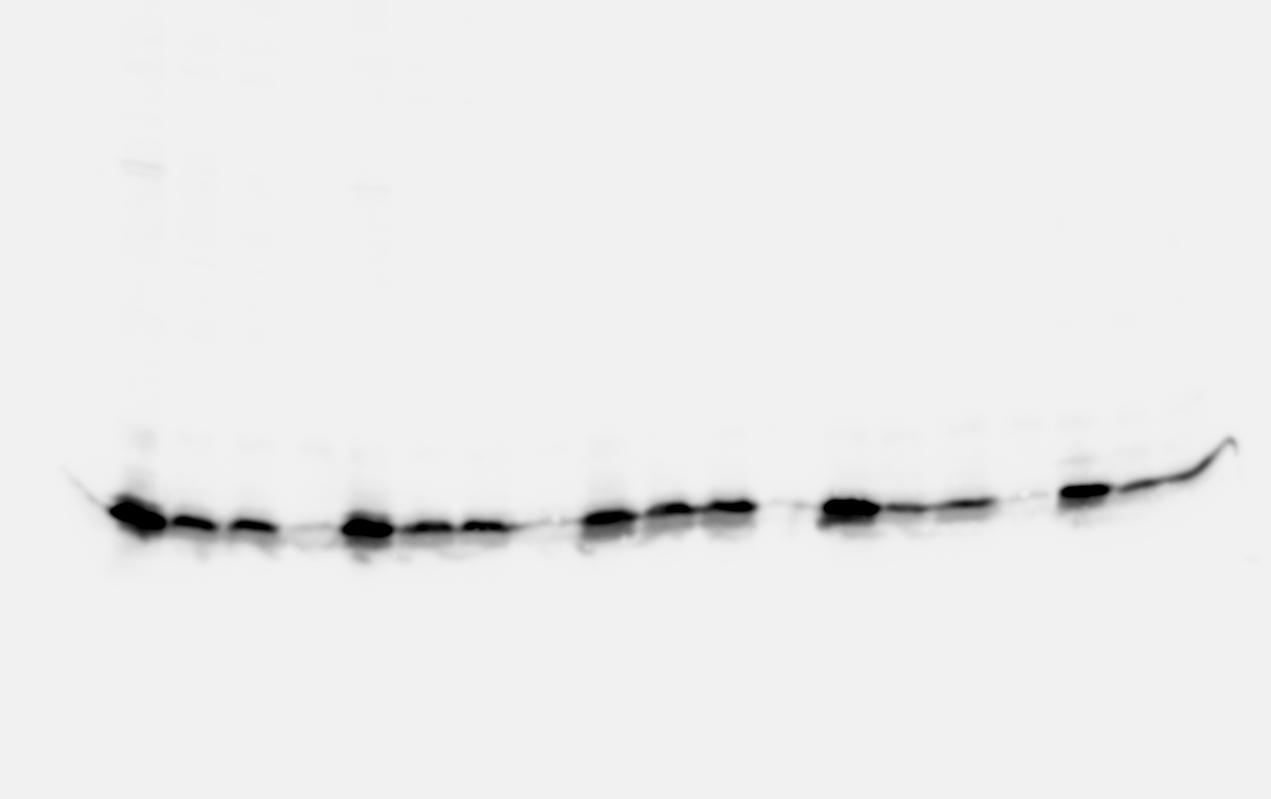

Supplement: Supplementary file 11 — Source Data for Figure 5 [file MSB-13-904-s009.zip › Source_Data_for_Figure_5/Figure05A/All_JPEG/Figure05A_32D_AKTVIII_pS6.jpg]

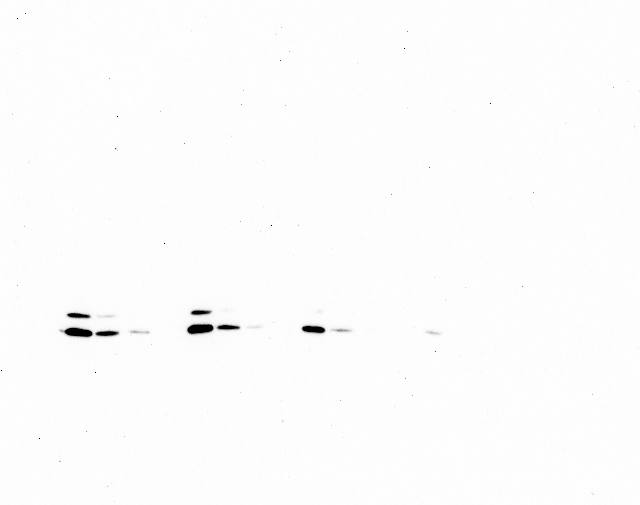

Supplement: Supplementary file 11 — Source Data for Figure 5 [file MSB-13-904-s009.zip › Source_Data_for_Figure_5/Figure05A/All_JPEG/Figure05A_CFUE_U0126_ppERK.jpg]

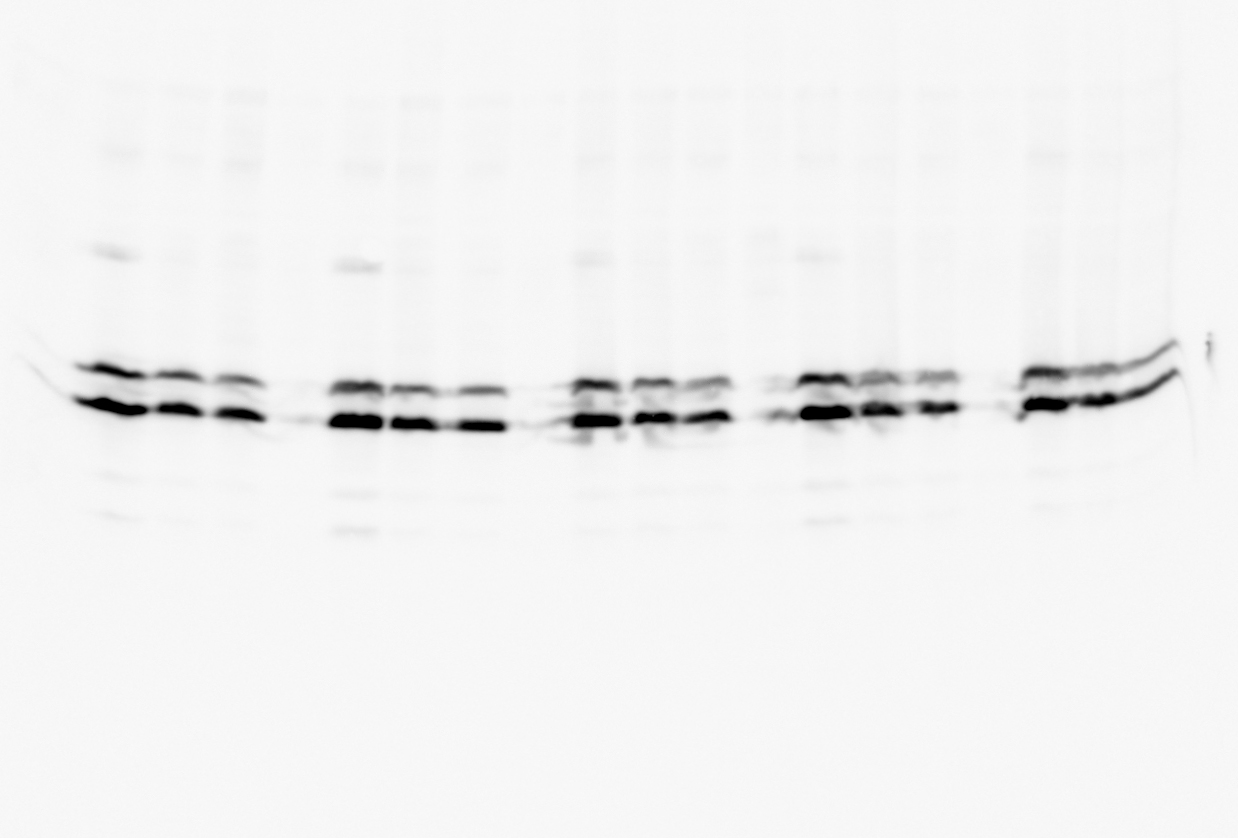

Supplement: Supplementary file 11 — Source Data for Figure 5 [file MSB-13-904-s009.zip › Source_Data_for_Figure_5/Figure05A/All_JPEG/Figure05A_32D_AKTVIII_ppERK.jpg]

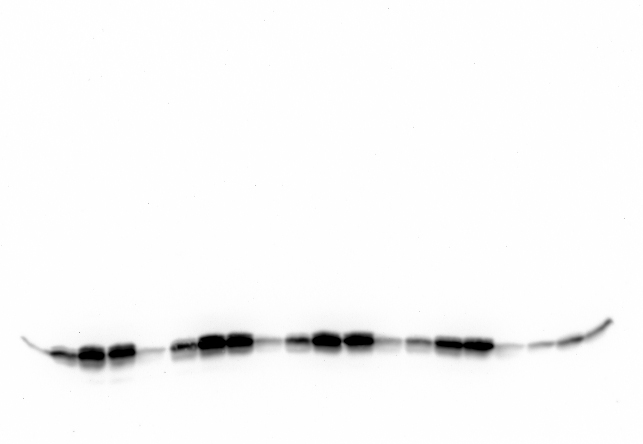

Supplement: Supplementary file 11 — Source Data for Figure 5 [file MSB-13-904-s009.zip › Source_Data_for_Figure_5/Figure05A/All_JPEG/Figure05A_CFUE_U0126_pS6.jpg]

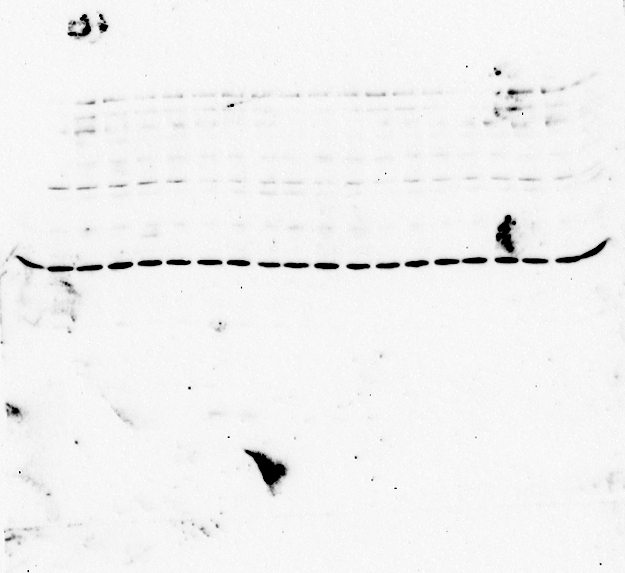

Supplement: Supplementary file 11 — Source Data for Figure 5 [file MSB-13-904-s009.zip › Source_Data_for_Figure_5/Figure05A/All_JPEG/Figure05A_CFUE_AKTVIII_PDI.jpg]

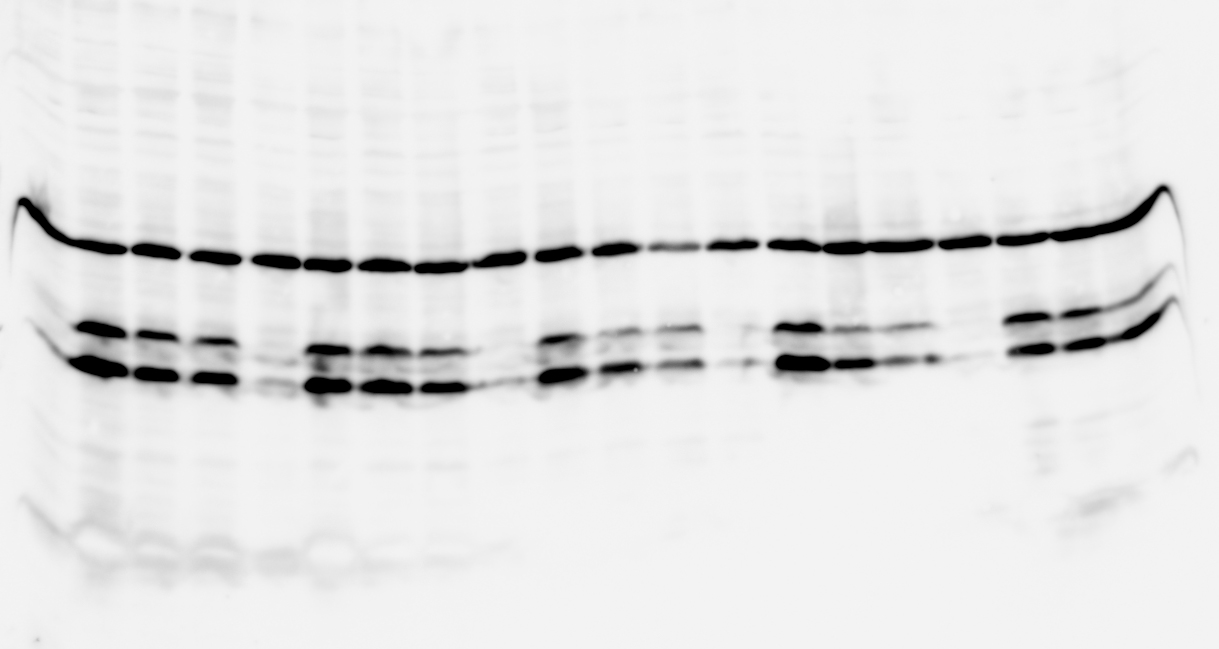

Supplement: Supplementary file 11 — Source Data for Figure 5 [file MSB-13-904-s009.zip › Source_Data_for_Figure_5/Figure05A/All_JPEG/Figure05A_32D_AKTVIII_PDI.jpg]

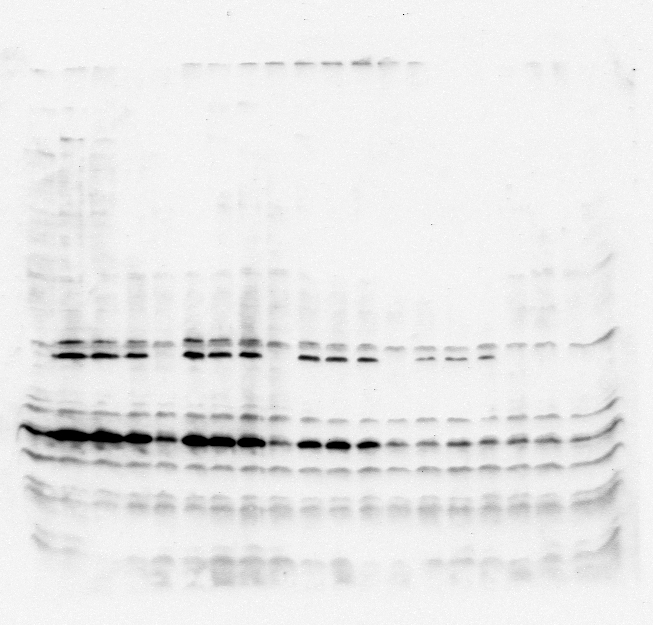

Supplement: Supplementary file 11 — Source Data for Figure 5 [file MSB-13-904-s009.zip › Source_Data_for_Figure_5/Figure05A/All_JPEG/Figure05A_BaF3_U0126_pS6.jpg]

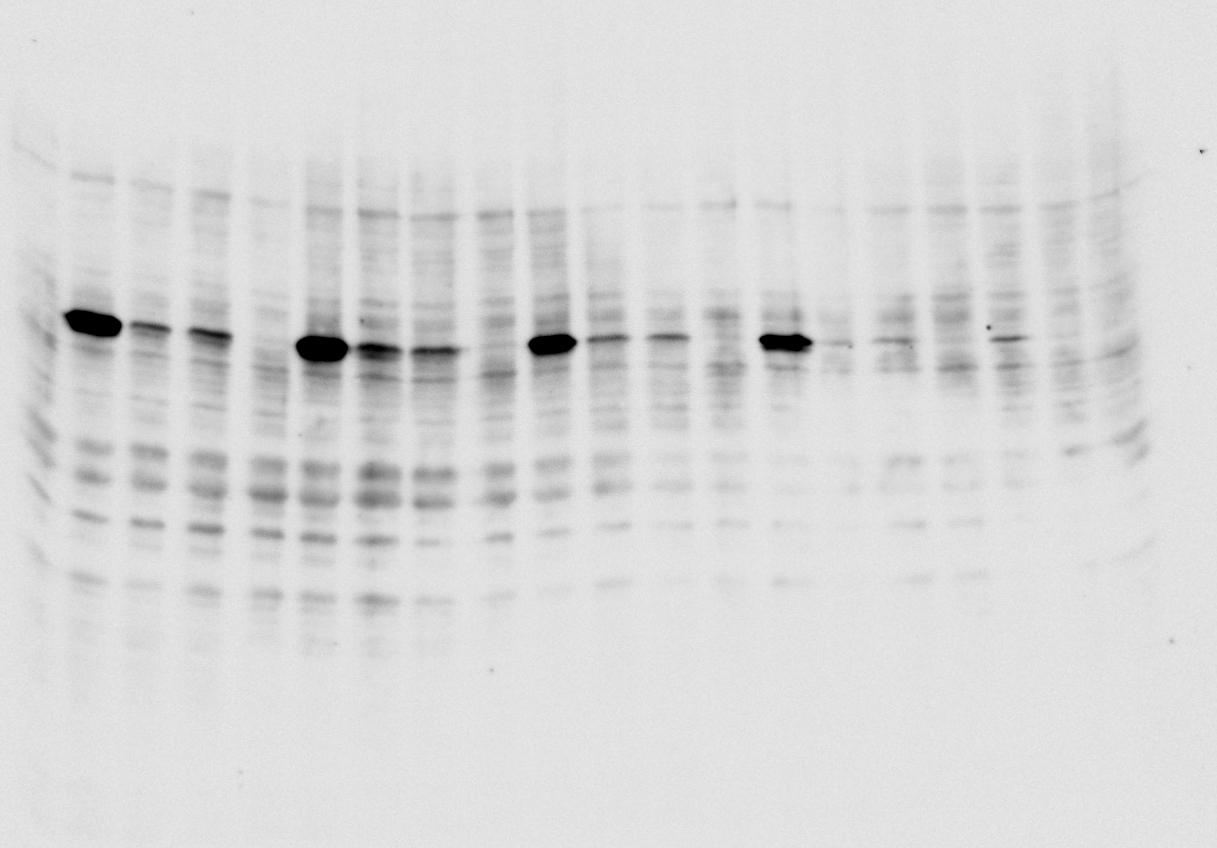

Supplement: Supplementary file 11 — Source Data for Figure 5 [file MSB-13-904-s009.zip › Source_Data_for_Figure_5/Figure05A/All_JPEG/Figure05A_32D_AKTVIII_pAKT.jpg]

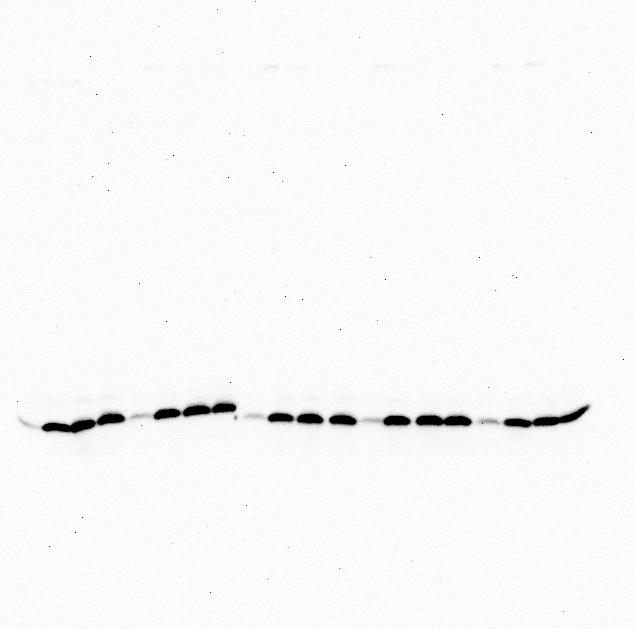

Supplement: Supplementary file 11 — Source Data for Figure 5 [file MSB-13-904-s009.zip › Source_Data_for_Figure_5/Figure05A/All_JPEG/Figure05A_BaF3_AKTVIII_pS6.jpg]

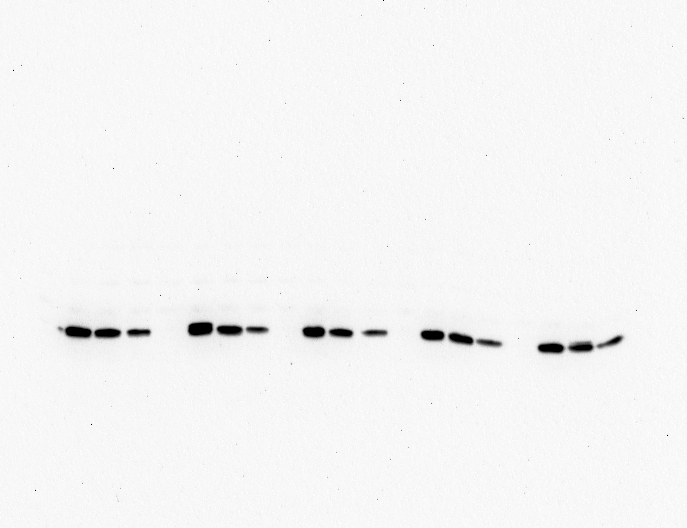

Supplement: Supplementary file 11 — Source Data for Figure 5 [file MSB-13-904-s009.zip › Source_Data_for_Figure_5/Figure05A/All_JPEG/Figure05A_CFUE_U0126_pAKT.jpg]

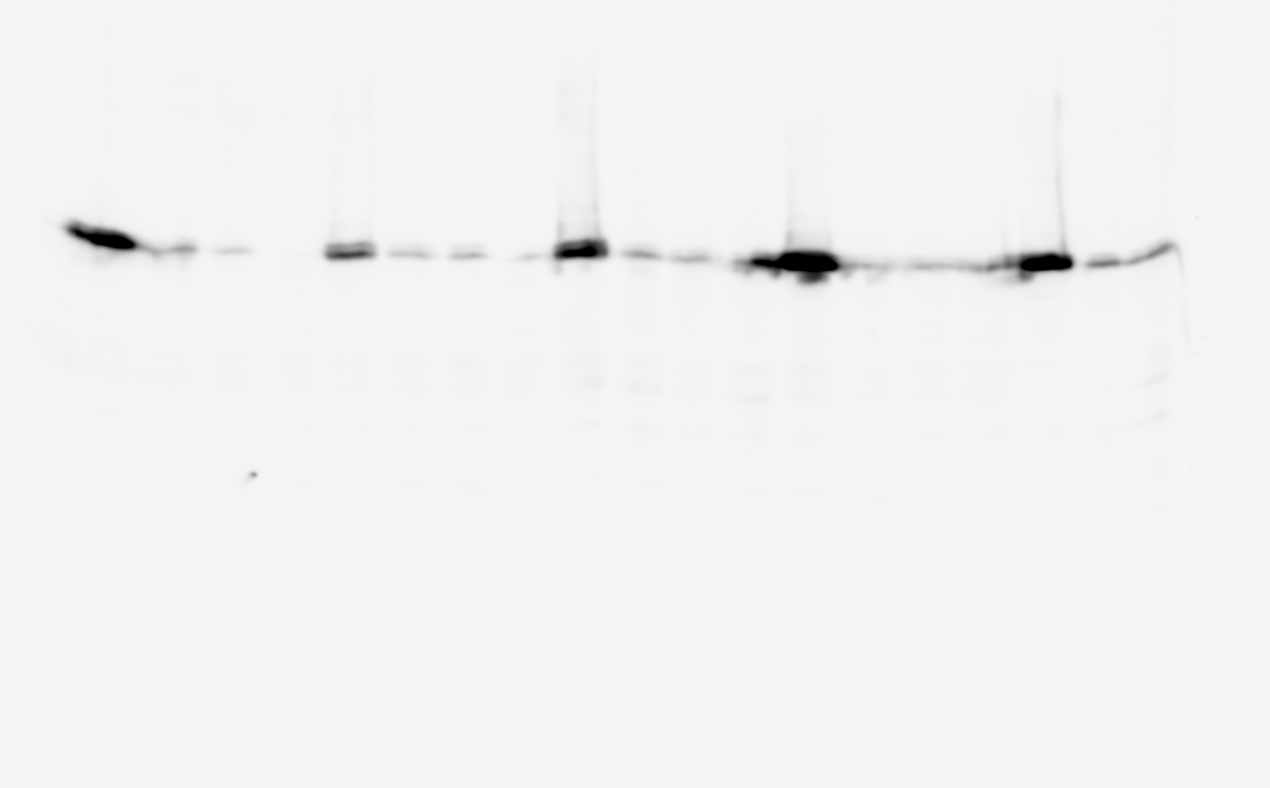

Supplement: Supplementary file 11 — Source Data for Figure 5 [file MSB-13-904-s009.zip › Source_Data_for_Figure_5/Figure05A/All_JPEG/Figure05A_32D_U0126_pAKT.jpg]

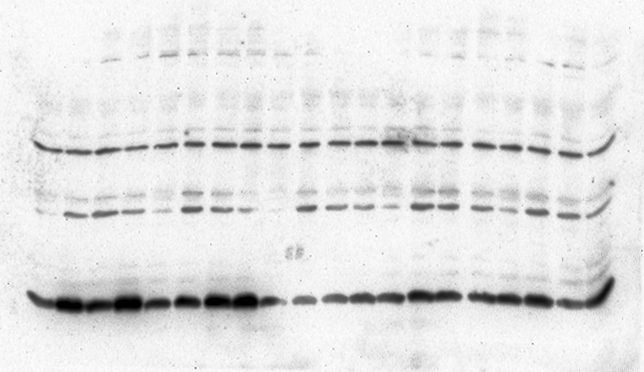

Supplement: Supplementary file 11 — Source Data for Figure 5 [file MSB-13-904-s009.zip › Source_Data_for_Figure_5/Figure05A/All_JPEG/Figure05A_BaF3_U0126_PDI.jpg]

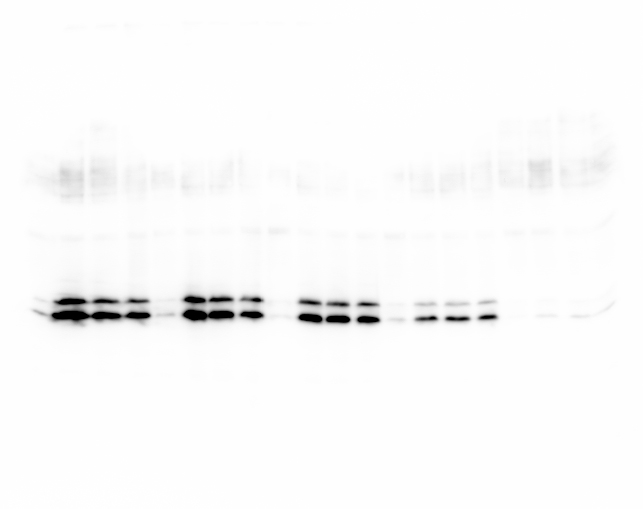

Supplement: Supplementary file 11 — Source Data for Figure 5 [file MSB-13-904-s009.zip › Source_Data_for_Figure_5/Figure05A/All_JPEG/Figure05A_BaF3_U0126_ppERK.jpg]

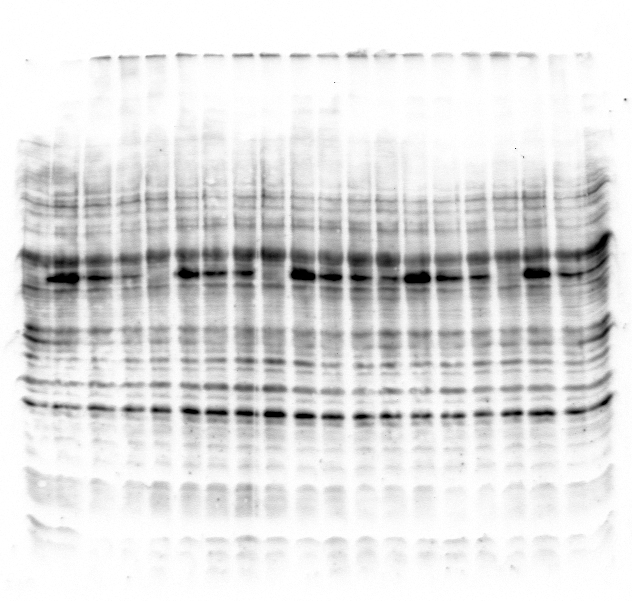

Supplement: Supplementary file 11 — Source Data for Figure 5 [file MSB-13-904-s009.zip › Source_Data_for_Figure_5/Figure05A/All_JPEG/Figure05A_BaF3_U0126_pAKT.jpg]

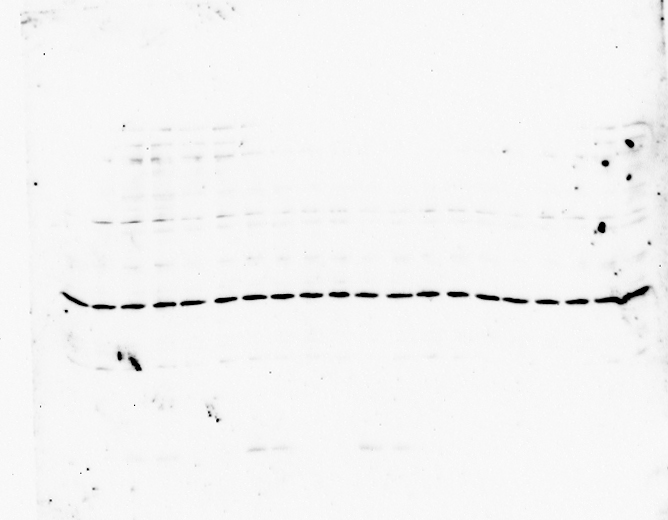

Supplement: Supplementary file 11 — Source Data for Figure 5 [file MSB-13-904-s009.zip › Source_Data_for_Figure_5/Figure05A/All_JPEG/Figure05A_CFUE_U0126_PDI.jpg]

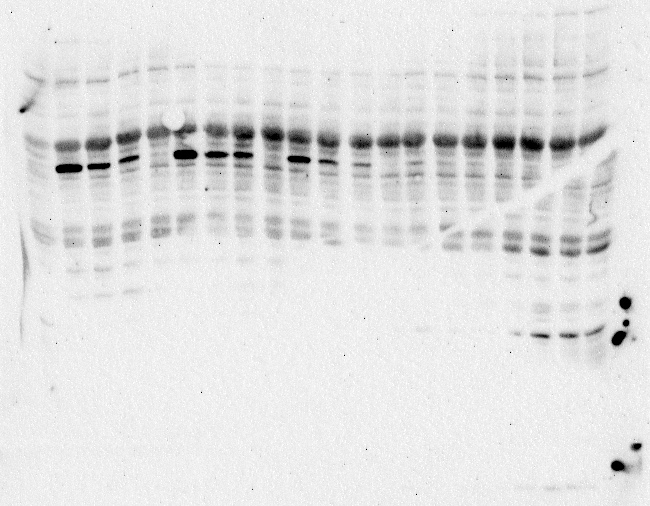

Supplement: Supplementary file 11 — Source Data for Figure 5 [file MSB-13-904-s009.zip › Source_Data_for_Figure_5/Figure05A/All_JPEG/Figure05A_BaF3_AKTVIII_pAKT.jpg]

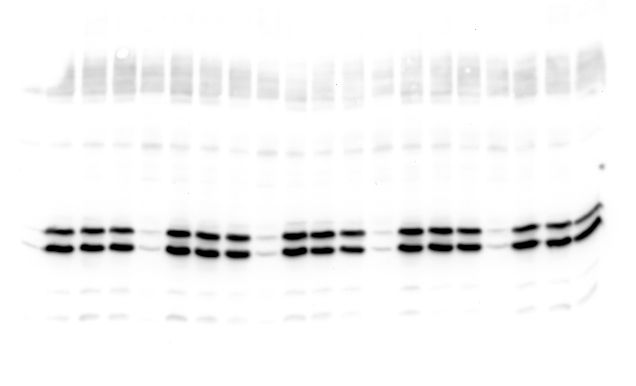

Supplement: Supplementary file 11 — Source Data for Figure 5 [file MSB-13-904-s009.zip › Source_Data_for_Figure_5/Figure05A/All_JPEG/Figure05A_BaF3_AKTVIII_ppERK.jpg]

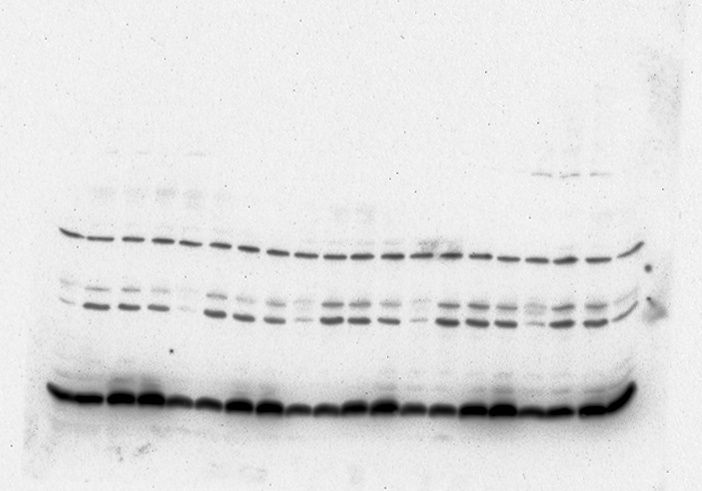

Supplement: Supplementary file 11 — Source Data for Figure 5 [file MSB-13-904-s009.zip › Source_Data_for_Figure_5/Figure05A/All_JPEG/Figure05A_BaF3_AKTVIII_PDI.jpg]

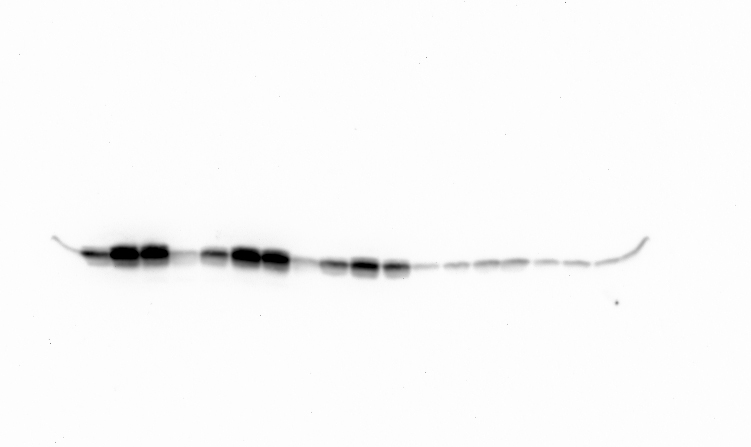

Supplement: Supplementary file 11 — Source Data for Figure 5 [file MSB-13-904-s009.zip › Source_Data_for_Figure_5/Figure05A/All_JPEG/Figure05A_CFUE_AKTVIII_pS6.jpg]

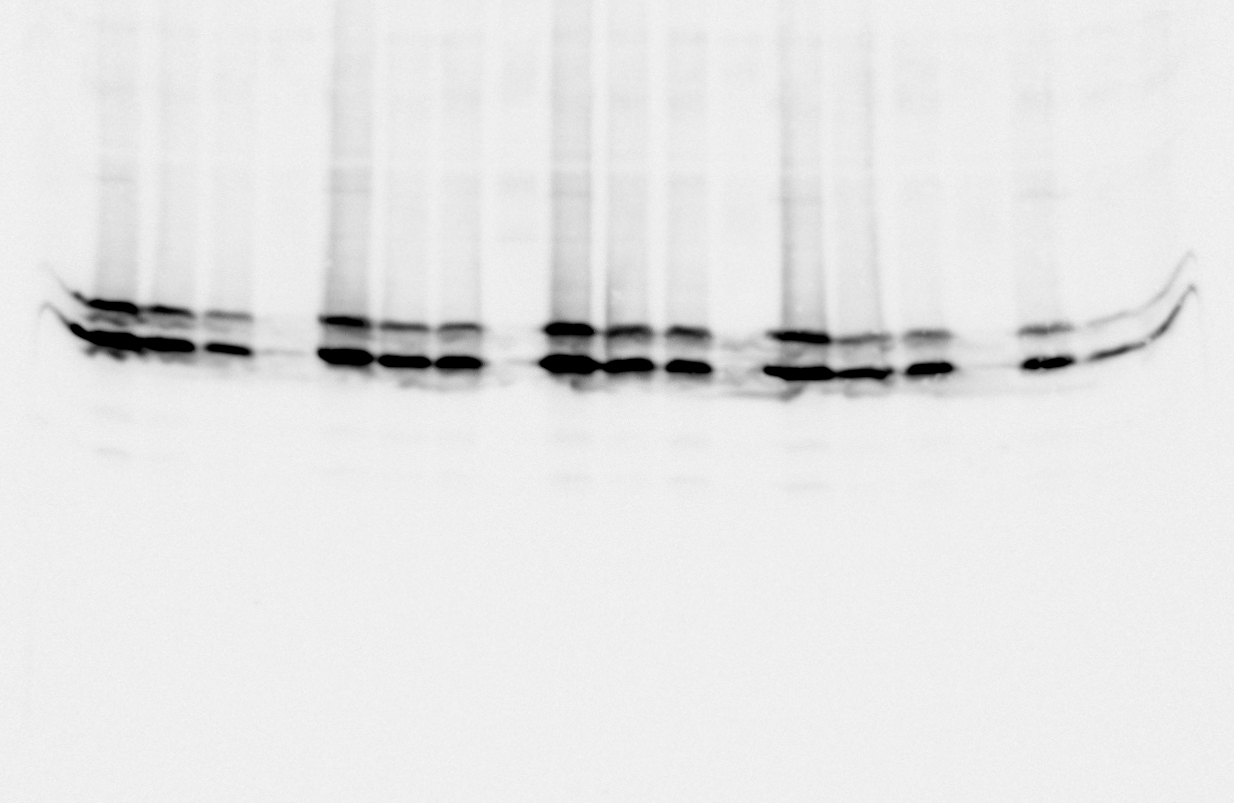

Supplement: Supplementary file 11 — Source Data for Figure 5 [file MSB-13-904-s009.zip › Source_Data_for_Figure_5/Figure05A/All_JPEG/Figure05A_32D_U0126_ppERK.jpg]

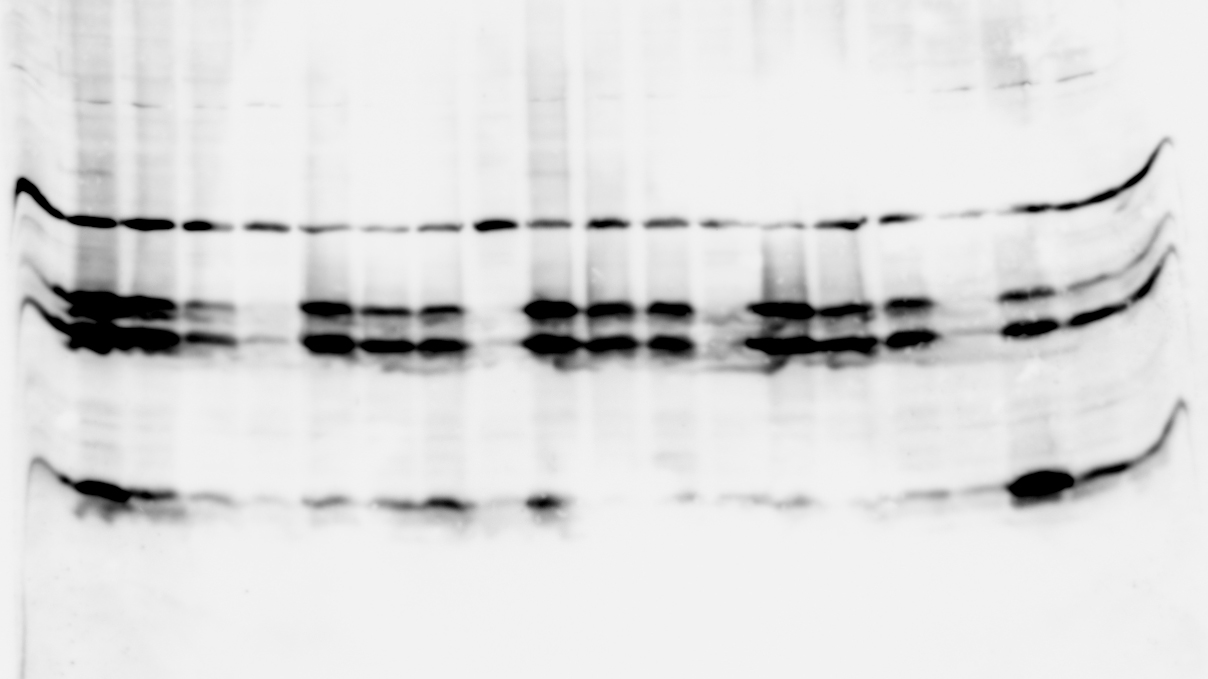

Supplement: Supplementary file 11 — Source Data for Figure 5 [file MSB-13-904-s009.zip › Source_Data_for_Figure_5/Figure05A/All_JPEG/Figure05A_32D_U0126_PDI.jpg]

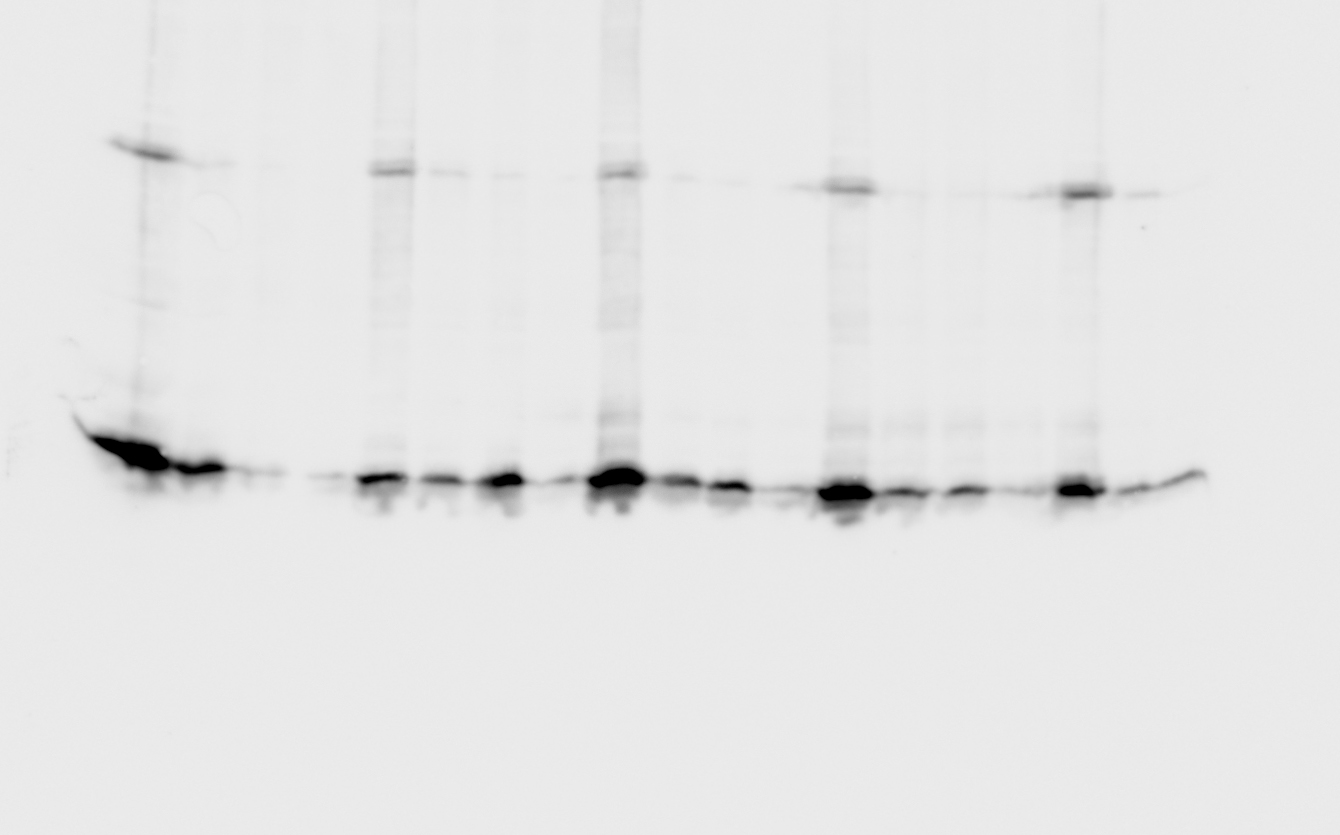

Supplement: Supplementary file 11 — Source Data for Figure 5 [file MSB-13-904-s009.zip › Source_Data_for_Figure_5/Figure05A/All_JPEG/Figure05A_32D_U0126_pS6.jpg]

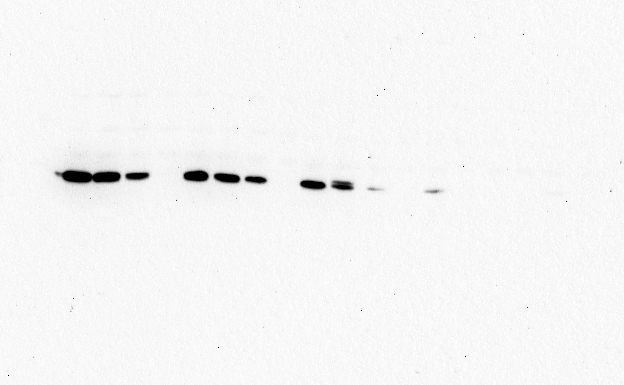

Supplement: Supplementary file 11 — Source Data for Figure 5 [file MSB-13-904-s009.zip › Source_Data_for_Figure_5/Figure05A/All_JPEG/Figure05A_CFUE_AKTVIII_pAKT.jpg]

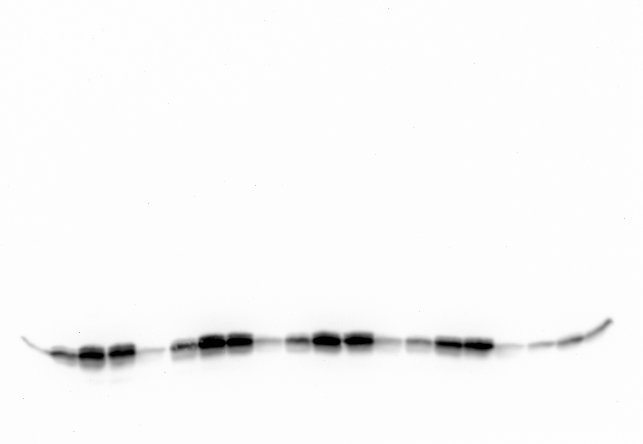

Supplement: Supplementary file 11 — Source Data for Figure 5 [file MSB-13-904-s009.zip › Source_Data_for_Figure_5/Figure05A/All_RAW/Figure05A_CFUE_U0126_pS6.tif]

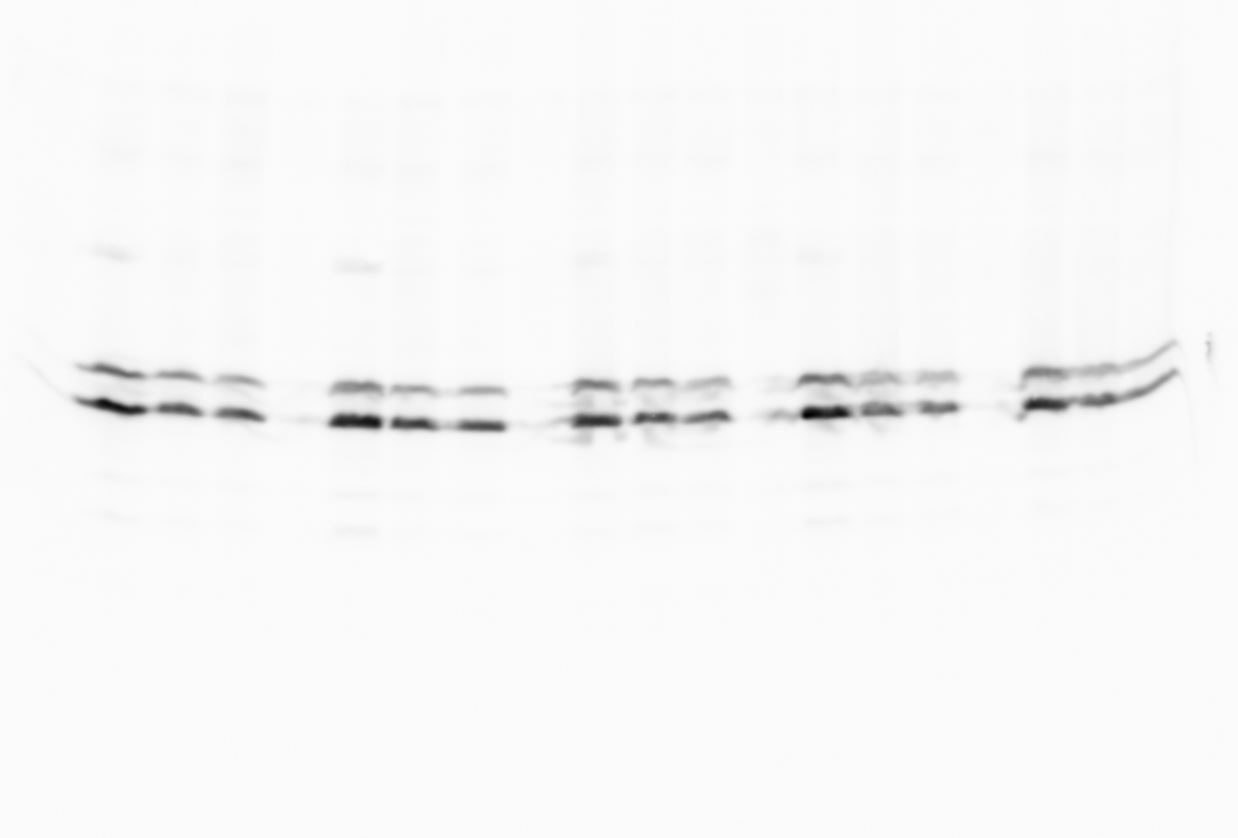

Supplement: Supplementary file 11 — Source Data for Figure 5 [file MSB-13-904-s009.zip › Source_Data_for_Figure_5/Figure05A/All_RAW/Figure05A_32D_AKTVIII_ppERK.tif]

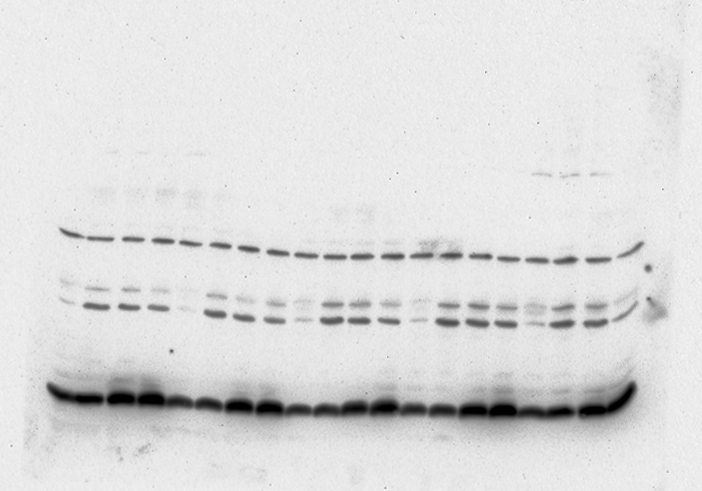

Supplement: Supplementary file 11 — Source Data for Figure 5 [file MSB-13-904-s009.zip › Source_Data_for_Figure_5/Figure05A/All_RAW/Figure05A_BaF3_AKTVIII_PDI.tif]

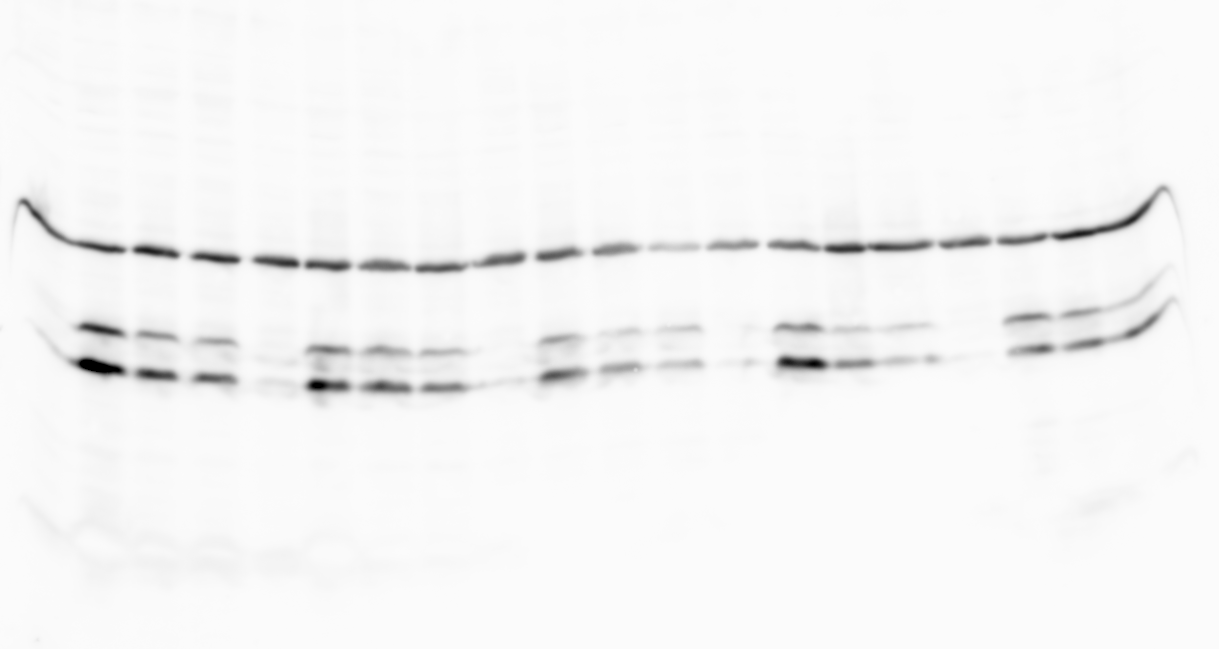

Supplement: Supplementary file 11 — Source Data for Figure 5 [file MSB-13-904-s009.zip › Source_Data_for_Figure_5/Figure05A/All_RAW/Figure05A_32D_AKTVIII_PDI.tif]

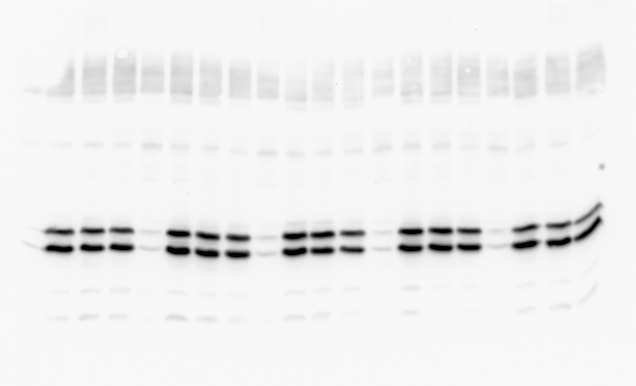

Supplement: Supplementary file 11 — Source Data for Figure 5 [file MSB-13-904-s009.zip › Source_Data_for_Figure_5/Figure05A/All_RAW/Figure05A_BaF3_AKTVIII_ppERK.tif]

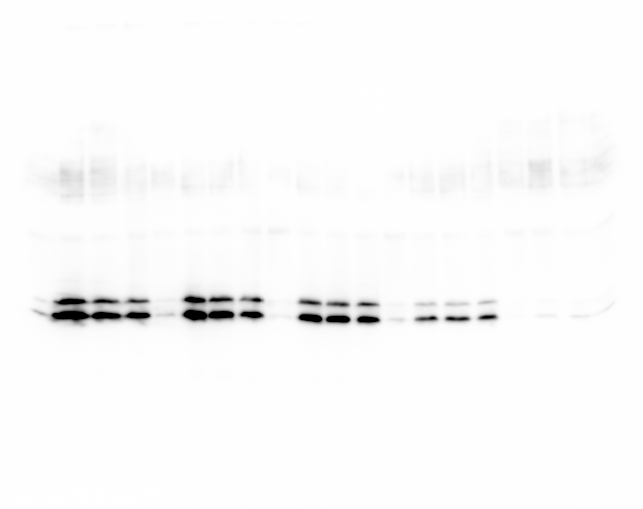

Supplement: Supplementary file 11 — Source Data for Figure 5 [file MSB-13-904-s009.zip › Source_Data_for_Figure_5/Figure05A/All_RAW/Figure05A_BaF3_U0126_ppERK.tif]

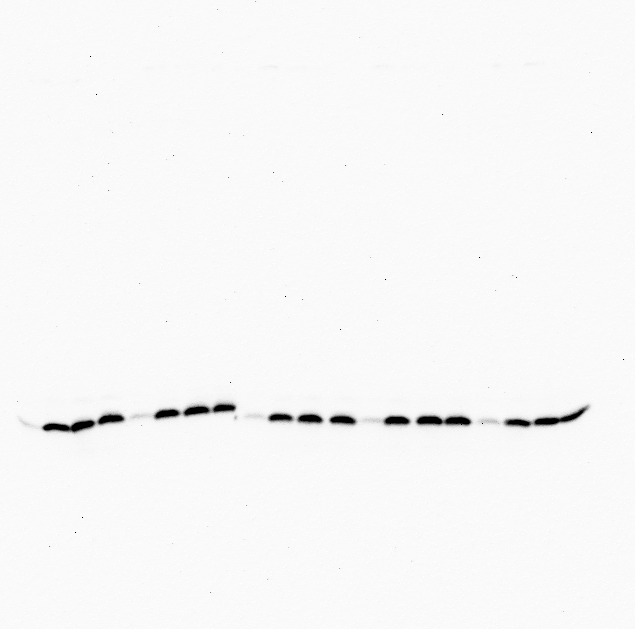

Supplement: Supplementary file 11 — Source Data for Figure 5 [file MSB-13-904-s009.zip › Source_Data_for_Figure_5/Figure05A/All_RAW/Figure05A_BaF3_AKTVIII_pS6.tif]

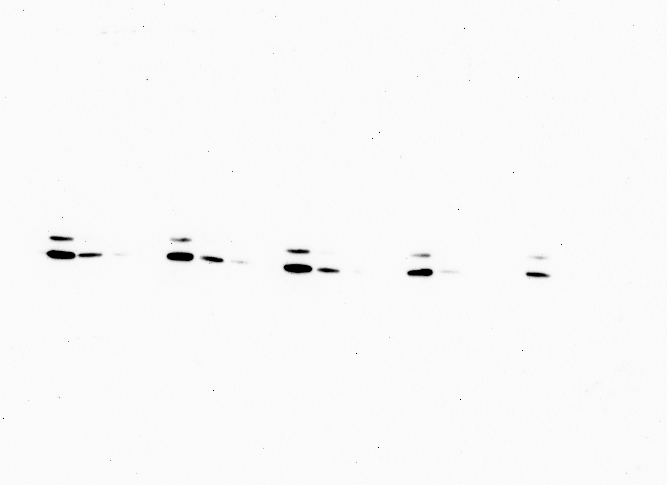

Supplement: Supplementary file 11 — Source Data for Figure 5 [file MSB-13-904-s009.zip › Source_Data_for_Figure_5/Figure05A/All_RAW/Figure05A_CFUE_AKTVIII_ppERK.tif]

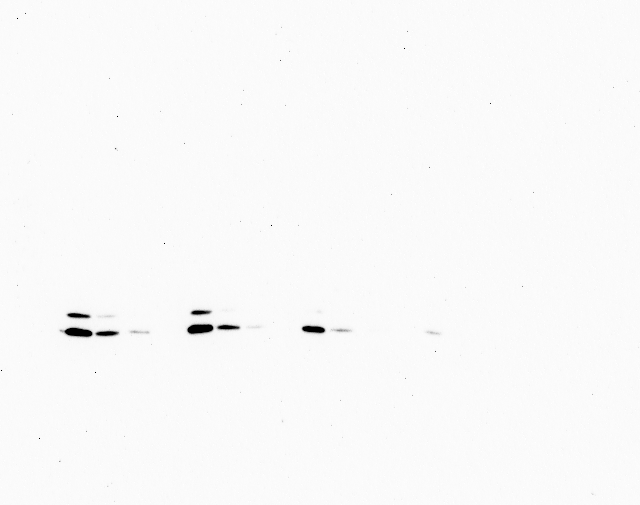

Supplement: Supplementary file 11 — Source Data for Figure 5 [file MSB-13-904-s009.zip › Source_Data_for_Figure_5/Figure05A/All_RAW/Figure05A_CFUE_U0126_ppERK.tif]

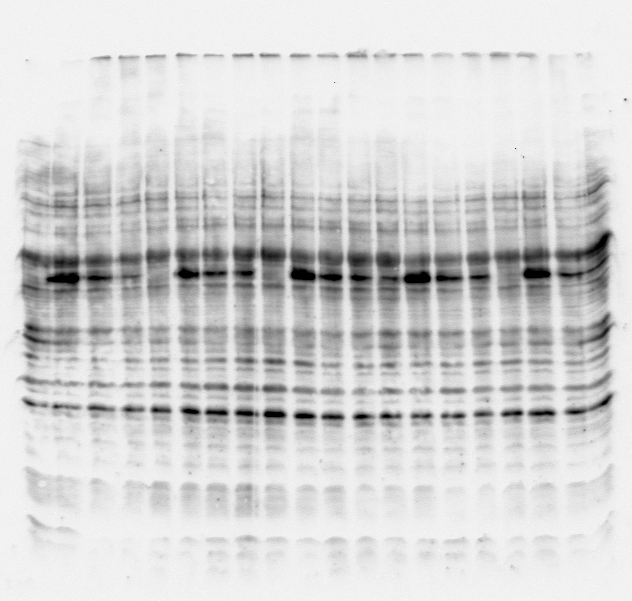

Supplement: Supplementary file 11 — Source Data for Figure 5 [file MSB-13-904-s009.zip › Source_Data_for_Figure_5/Figure05A/All_RAW/Figure05A_BaF3_U0126_pAKT.tif]

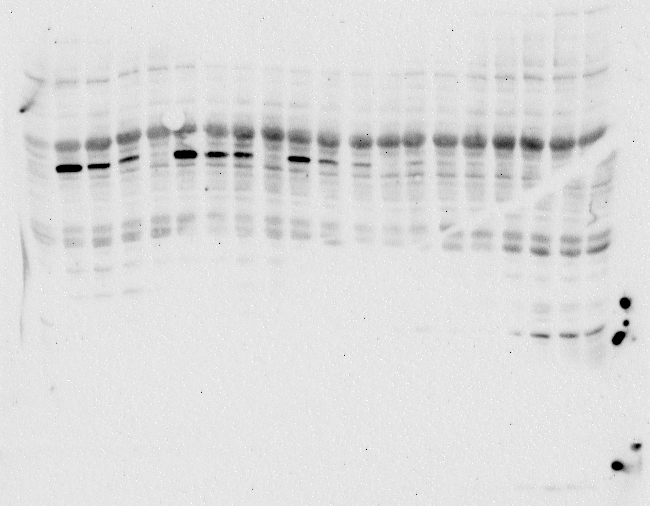

Supplement: Supplementary file 11 — Source Data for Figure 5 [file MSB-13-904-s009.zip › Source_Data_for_Figure_5/Figure05A/All_RAW/Figure05A_BaF3_AKTVIII_pAKT.tif]

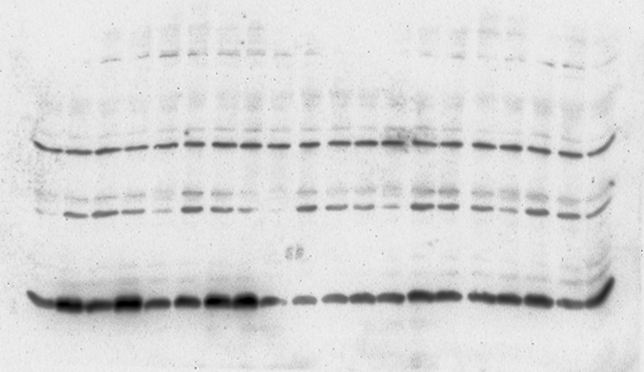

Supplement: Supplementary file 11 — Source Data for Figure 5 [file MSB-13-904-s009.zip › Source_Data_for_Figure_5/Figure05A/All_RAW/Figure05A_BaF3_U0126_PDI.tif]

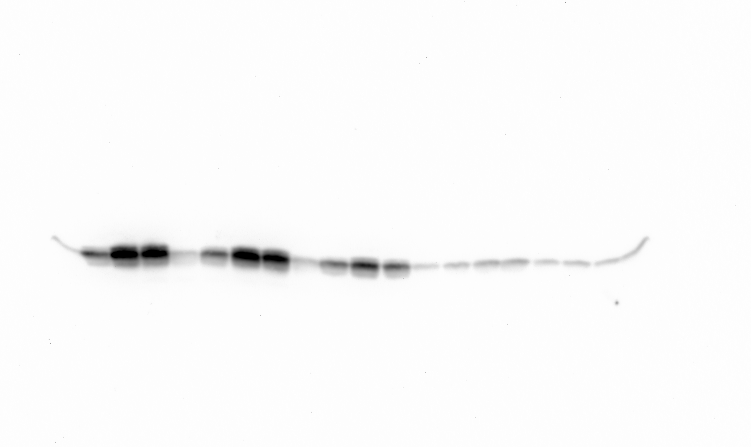

Supplement: Supplementary file 11 — Source Data for Figure 5 [file MSB-13-904-s009.zip › Source_Data_for_Figure_5/Figure05A/All_RAW/Figure05A_CFUE_AKTVIII_pS6.tif]

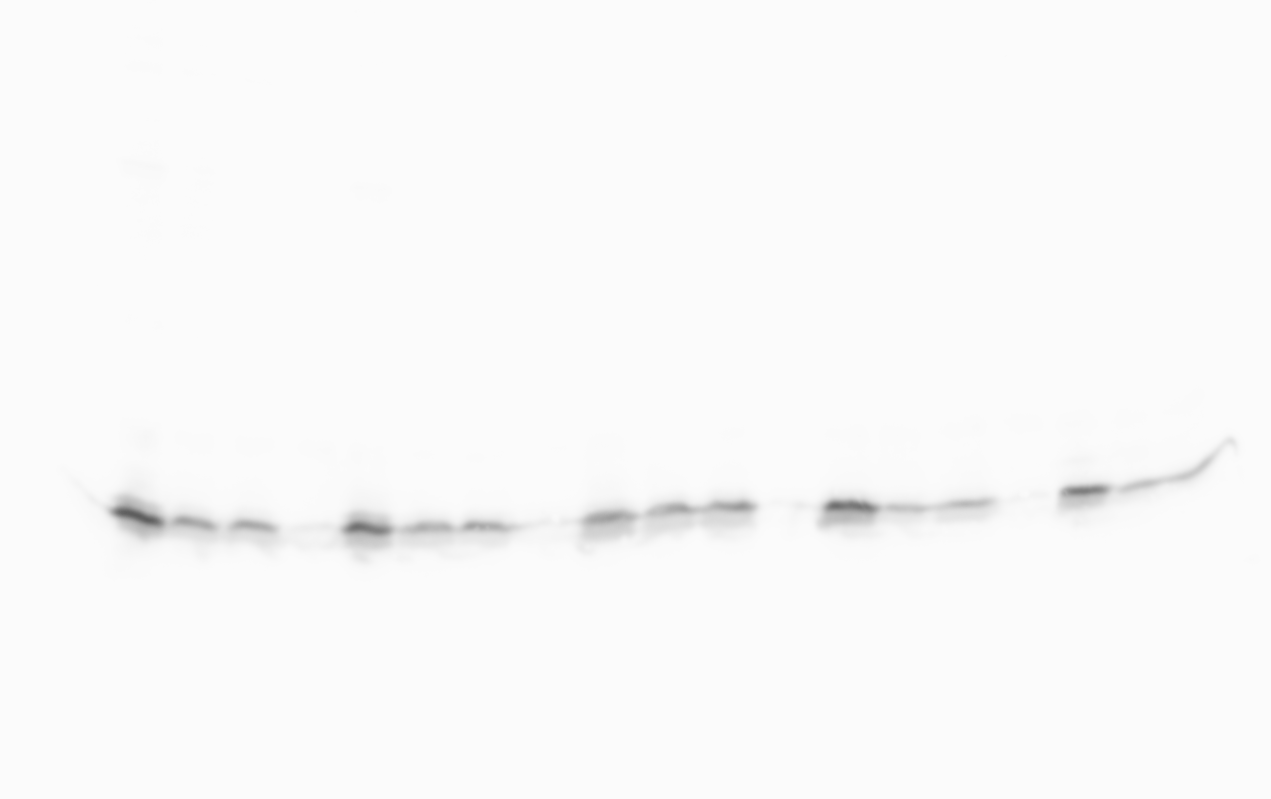

Supplement: Supplementary file 11 — Source Data for Figure 5 [file MSB-13-904-s009.zip › Source_Data_for_Figure_5/Figure05A/All_RAW/Figure05A_32D_AKTVIII_pS6.tif]

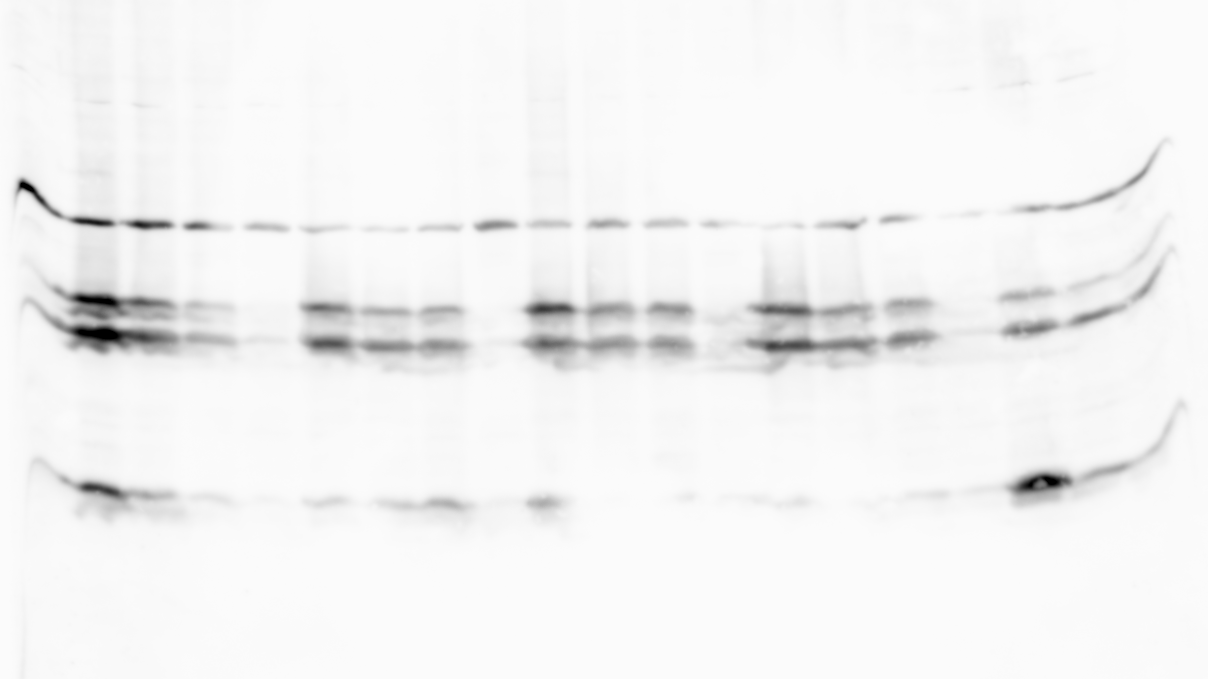

Supplement: Supplementary file 11 — Source Data for Figure 5 [file MSB-13-904-s009.zip › Source_Data_for_Figure_5/Figure05A/All_RAW/Figure05A_32D_U0126_PDI.tif]

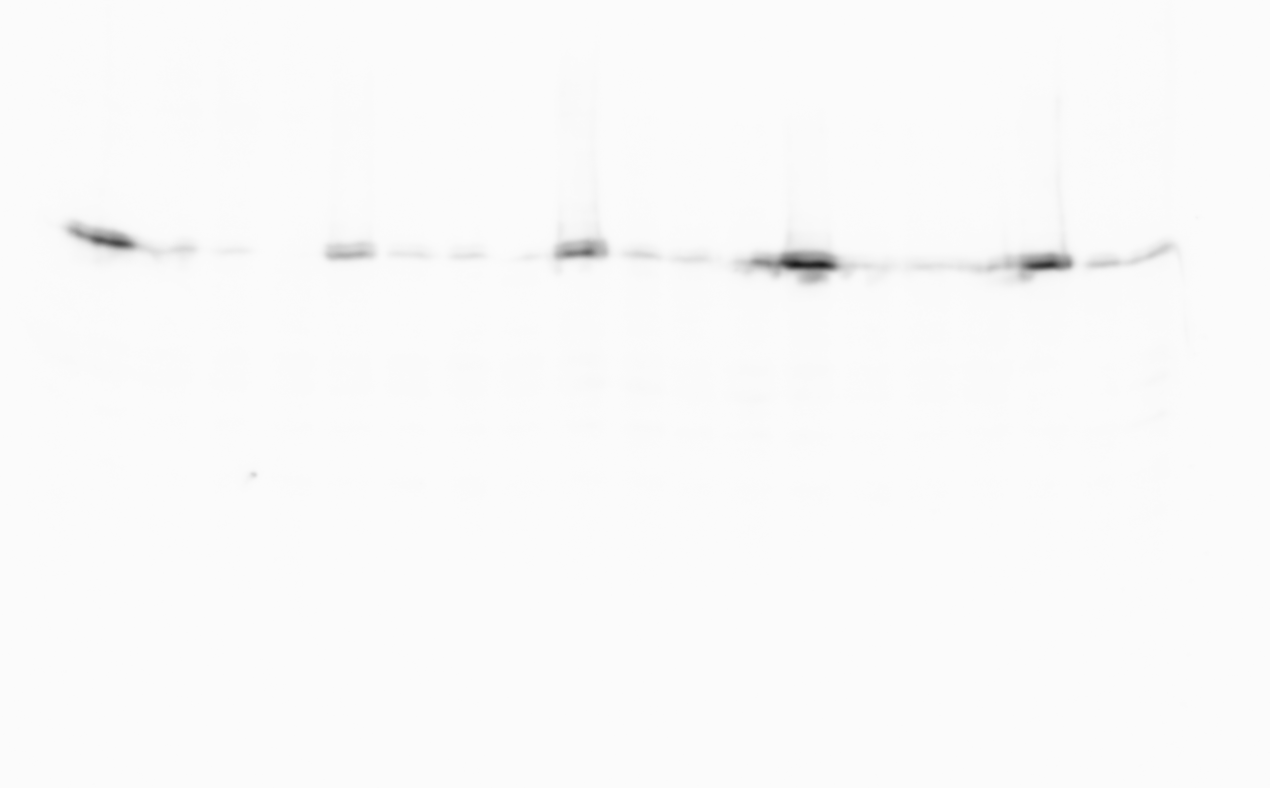

Supplement: Supplementary file 11 — Source Data for Figure 5 [file MSB-13-904-s009.zip › Source_Data_for_Figure_5/Figure05A/All_RAW/Figure05A_32D_U0126_pAKT.tif]

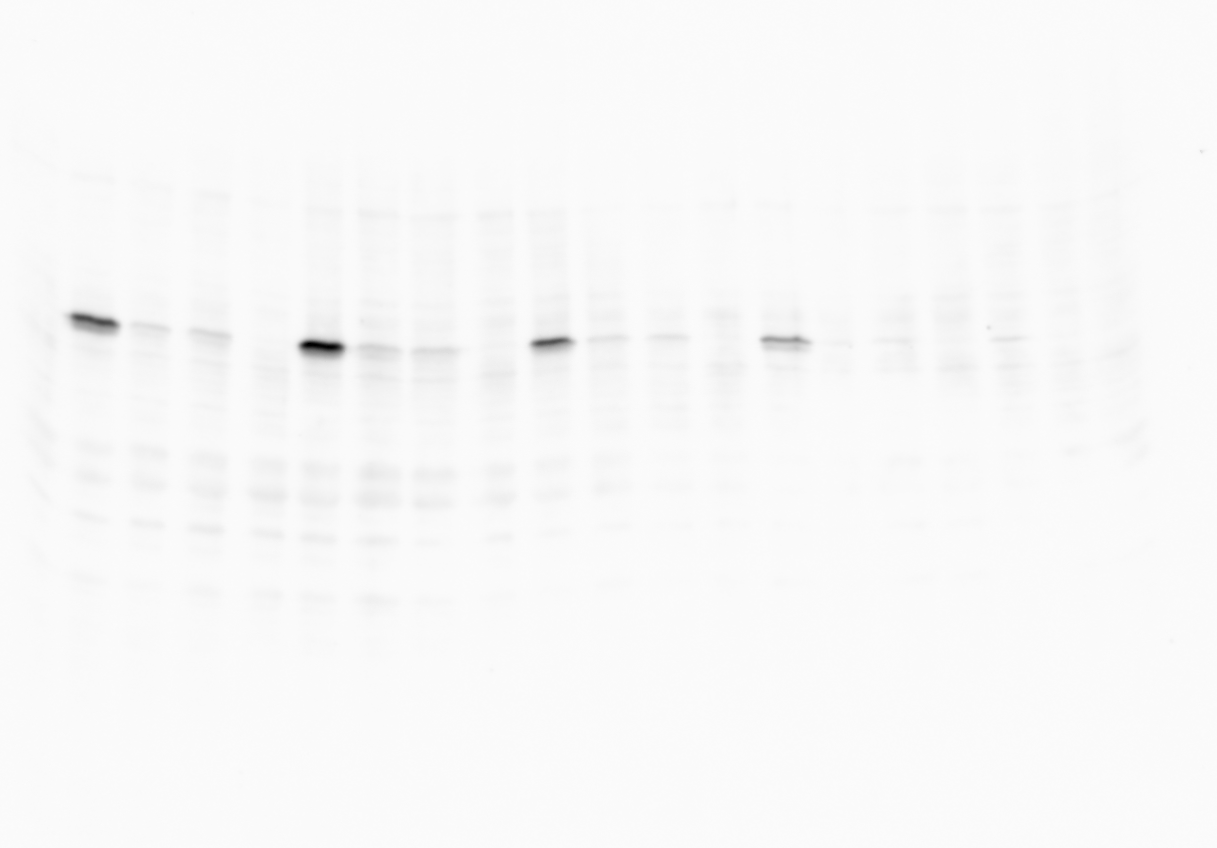

Supplement: Supplementary file 11 — Source Data for Figure 5 [file MSB-13-904-s009.zip › Source_Data_for_Figure_5/Figure05A/All_RAW/Figure05A_32D_AKTVIII_pAKT.tif]

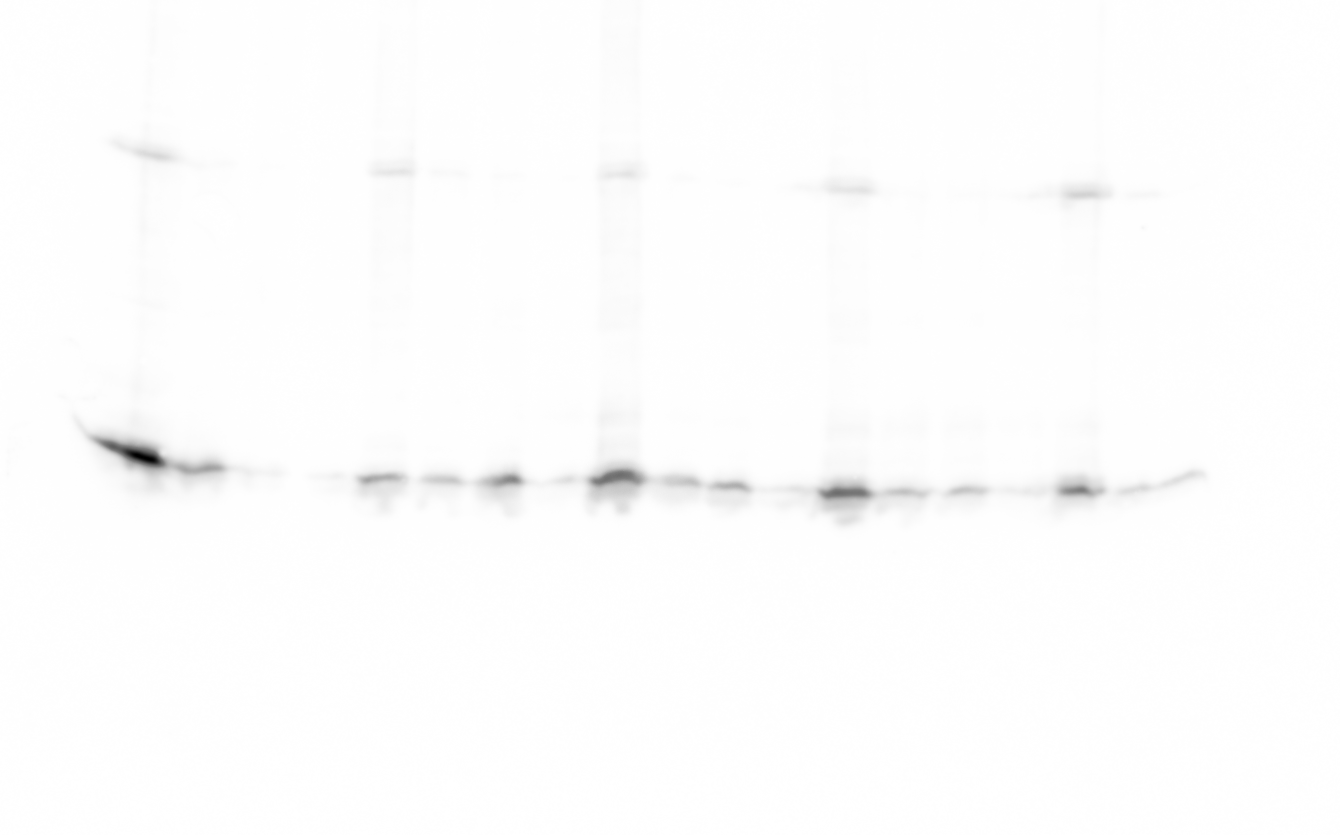

Supplement: Supplementary file 11 — Source Data for Figure 5 [file MSB-13-904-s009.zip › Source_Data_for_Figure_5/Figure05A/All_RAW/Figure05A_32D_U0126_pS6.tif]

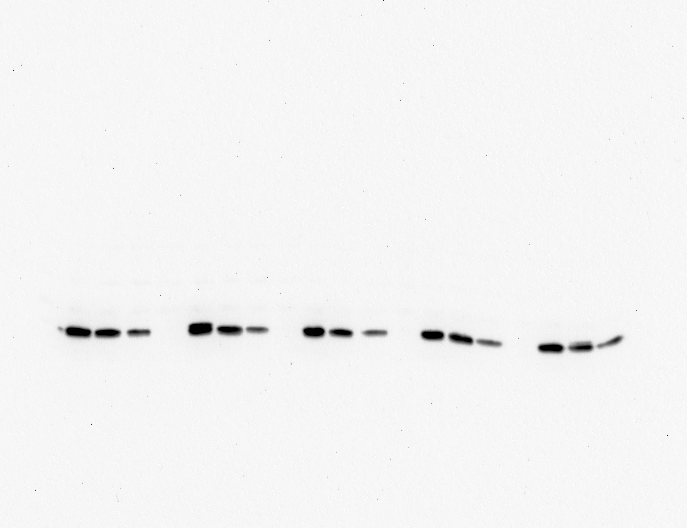

Supplement: Supplementary file 11 — Source Data for Figure 5 [file MSB-13-904-s009.zip › Source_Data_for_Figure_5/Figure05A/All_RAW/Figure05A_CFUE_U0126_pAKT.tif]

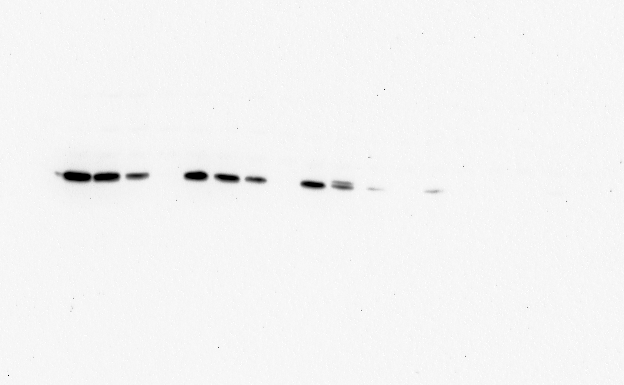

Supplement: Supplementary file 11 — Source Data for Figure 5 [file MSB-13-904-s009.zip › Source_Data_for_Figure_5/Figure05A/All_RAW/Figure05A_CFUE_AKTVIII_pAKT.tif]

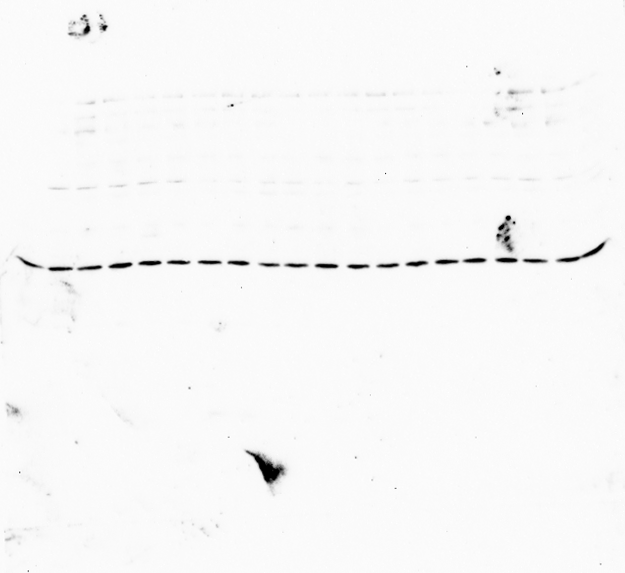

Supplement: Supplementary file 11 — Source Data for Figure 5 [file MSB-13-904-s009.zip › Source_Data_for_Figure_5/Figure05A/All_RAW/Figure05A_CFUE_AKTVIII_PDI.tif]

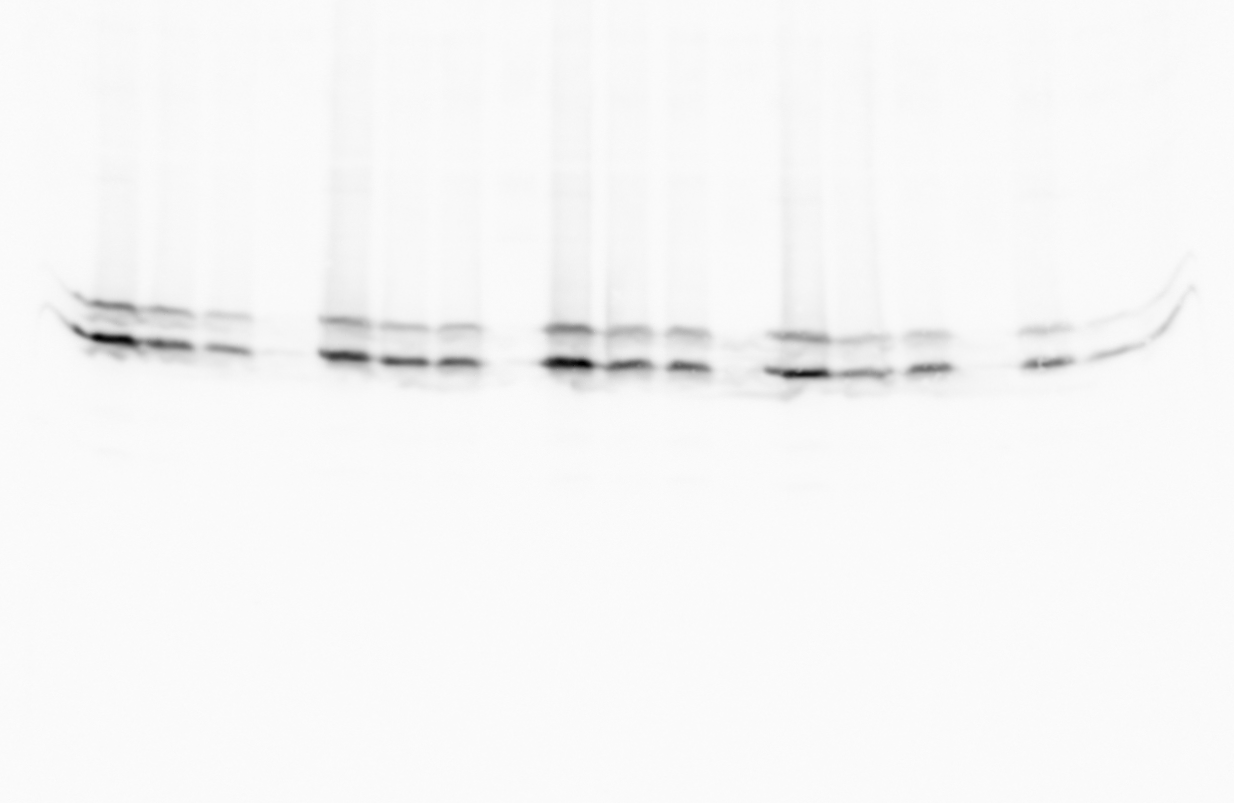

Supplement: Supplementary file 11 — Source Data for Figure 5 [file MSB-13-904-s009.zip › Source_Data_for_Figure_5/Figure05A/All_RAW/Figure05A_32D_U0126_ppERK.tif]

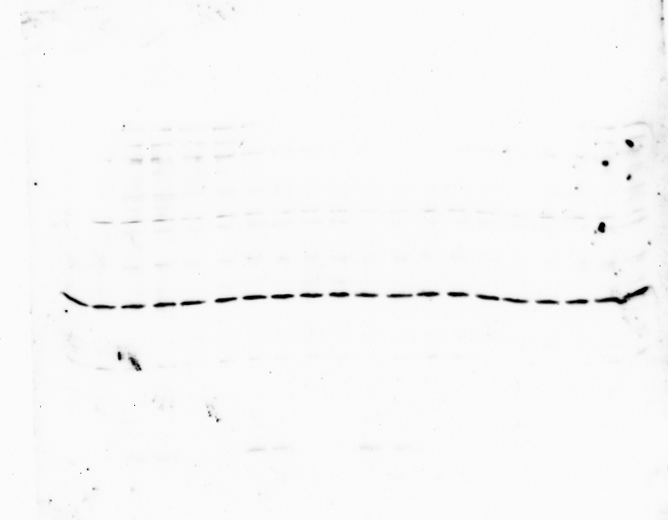

Supplement: Supplementary file 11 — Source Data for Figure 5 [file MSB-13-904-s009.zip › Source_Data_for_Figure_5/Figure05A/All_RAW/Figure05A_CFUE_U0126_PDI.tif]

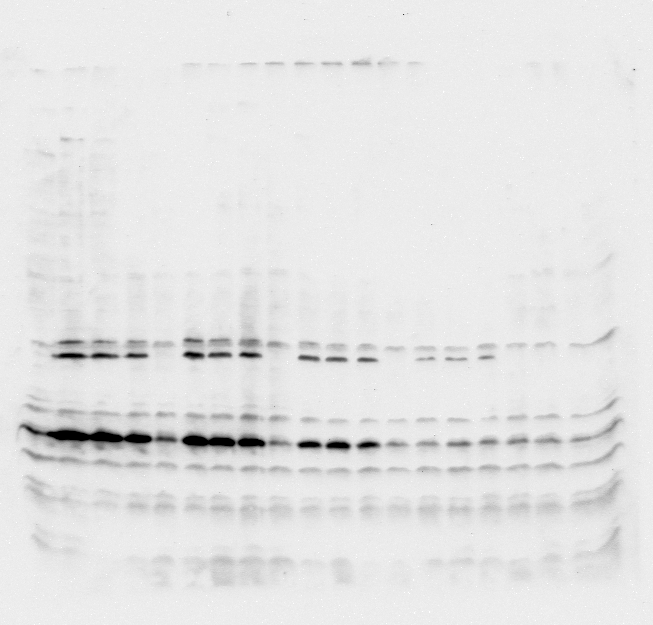

Supplement: Supplementary file 11 — Source Data for Figure 5 [file MSB-13-904-s009.zip › Source_Data_for_Figure_5/Figure05A/All_RAW/Figure05A_BaF3_U0126_pS6.tif]
